# Orthogonal Nanoparticle Catalysis with Organogermanes

**DOI:** 10.1002/anie.201910060

**Published:** 2019-10-23

**Authors:** Christoph Fricke, Grant J. Sherborne, Ignacio Funes‐Ardoiz, Erdem Senol, Sinem Guven, Franziska Schoenebeck

**Affiliations:** ^1^ Institute of Organic Chemistry RWTH Aachen University Landoltweg 1 52074 Aachen Germany

**Keywords:** catalysis, chemoselectivity, density functional calculations, germanium, nanoparticles

## Abstract

Although nanoparticles are widely used as catalysts, little is known about their potential ability to trigger privileged transformations as compared to homogeneous molecular or bulk heterogeneous catalysts. We herein demonstrate (and rationalize) that nanoparticles display orthogonal reactivity to molecular catalysts in the cross‐coupling of aryl halides with aryl germanes. While the aryl germanes are unreactive in L_*n*_Pd^0^/L_*n*_Pd^II^ catalysis and allow selective functionalization of established coupling partners in their presence, they display superior reactivity under Pd nanoparticle conditions, outcompeting established coupling partners (such as ArBPin and ArBMIDA) and allowing air‐tolerant, base‐free, and orthogonal access to valuable and challenging biaryl motifs. As opposed to the notoriously unstable polyfluoroaryl‐ and 2‐pyridylboronic acids, the corresponding germanes are highly stable and readily coupled. Our mechanistic and computational studies provide unambiguous support of nanoparticle catalysis and suggest that owing to the electron richness of aryl germanes, they preferentially react by electrophilic aromatic substitution, and in turn are preferentially activated by the more electrophilic nanoparticles.

## Graphical abstract

**Palladium nanoparticles** display orthogonal reactivity to molecular catalysts in the cross‐coupling of aryl halides with aryl germanes. While aryl germanes are unreactive under L_*n*_Pd^0^/L_*n*_Pd^II^ catalysis, they display superior reactivity under Pd nanoparticle conditions. Owing to their electron richness, aryl germanes preferentially react by electrophilic aromatic substitution, and in turn are preferentially activated by the more electrophilic nanoparticles.

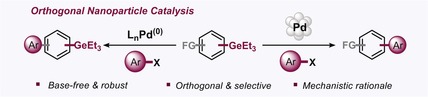

## Introduction

Over the past decade the nanotechnology industry has surged forward to reach a global market of greater than one trillion US dollars in 2018,^1^ with diverse applications ranging from materials for solar cells, photonics, cosmetics or biomedical applications, such as drug delivery, tissue engineering, and cancer therapy, to catalysis.^2^, ^3^, ^4^ While nanoparticle catalysts are generally more reactive than their bulk metal counterparts because of their greater surface area, they frequently need more forcing reaction conditions than homogeneous molecular metal catalysts to trigger the same transformations.^5^ However, leaching from molecular catalysts can cause the release of nanoparticles, and consequently their involvement as potentially “true” catalytic species in established catalytic transformations has also been subject to intense debates.^5^, ^6^, ^7^ As opposed to homogeneous molecular catalysis, lower loadings in metal are frequently required under nanoparticle catalysis conditions.^2^, ^5^, ^8^ This characteristic, paired with the low cost of preparation and the absence of sensitive ligands in nanoparticles, has led to immense interest academically as well as their implementation in large‐scale industrial processes.^9^

However, despite the many publications on nanoparticle catalysis, to date, there is no precedence of unambiguously unique and orthogonal reactivity of nanoparticles compared to homogeneous molecular or heterogeneous bulk catalysts in organic transformations. Such insights would be of utmost importance as there is a high demand for innovative and orthogonal synthetic strategies. Especially, a modular and straightforward access to richly functionalized biaryl motifs is in considerable demand, owing to their widespread abundance in drugs, materials, or privileged catalysts.^10^ In this context, a strategy that operates in an orthogonal fashion to the widely employed Pd‐catalyzed cross‐coupling technology^11^ would offer an additional dimension for structural diversification as well as potential to overcome existing synthetic challenges through orthogonal synthetic approaches.

**Figure 1:**
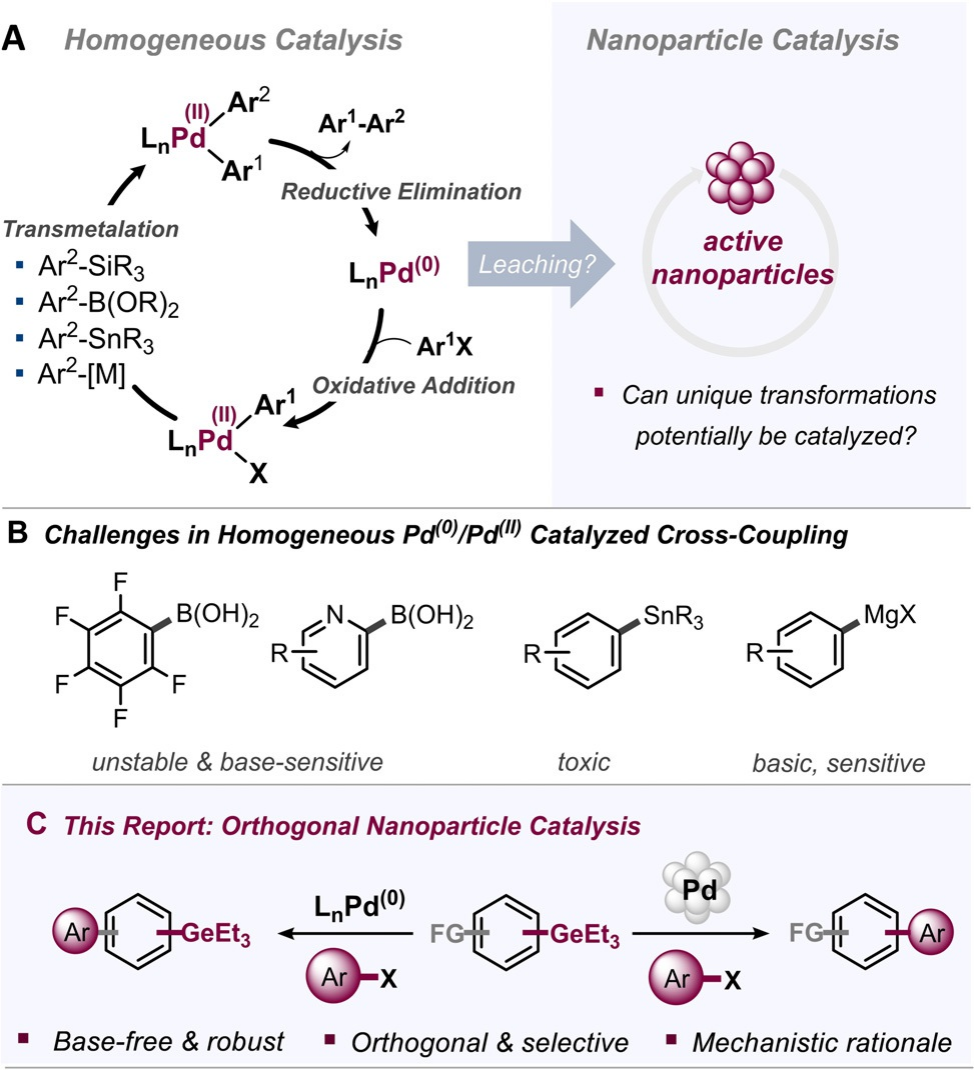
A) Homogeneous versus nanoparticle catalysis and current leaching assumptions. B) Challenges associated with established transmetalation reagents for C(sp^2^)−C(sp^2^) couplings. C) This work.

Since the advent of metal‐catalyzed cross‐coupling technology more than 40 years ago, the field has grown to be ever‐increasingly enabling owing to numerous efforts to push the frontiers of catalyst development and mechanistic understanding; yet the transmetalation step to date largely still relies on the original set of reagents.^11^ The Suzuki–Miyaura cross‐coupling of organoboron reagents with aryl halides is most widely and ubiquitously used among organic, medicinal, and materials chemists in academia and industry,^12^ as the established alternatives can be associated with basicity, instability (organomagnesium and ‐zinc reagents), toxicity (organotin), or lower reactivity. Despite its relative mildness, broad scope, and high reactivity, this popular coupling class is not free of challenges, however. These include, for example, the occasional instability of boronic acids, which is particularly pronounced in the case of 2‐pyridyl‐ and multifluoroarylboronic acids and further aggravated by the presence of (and need for) base.^13^ Ingenious masking strategies^14^, ^15^ or elegantly more reactive systems that make use of aryl diazonium salts as acceptors^16^ have been developed to balance the relative kinetics of deactivation versus productive cross‐coupling in these cases.^17^ Some toxicity concerns in conjunction with organoboron compounds and their derivatives have recently also been reported,^18^ which may create a need for alternative approaches in certain applications (Figure 1).

## Results and Discussion

**Figure 2:**
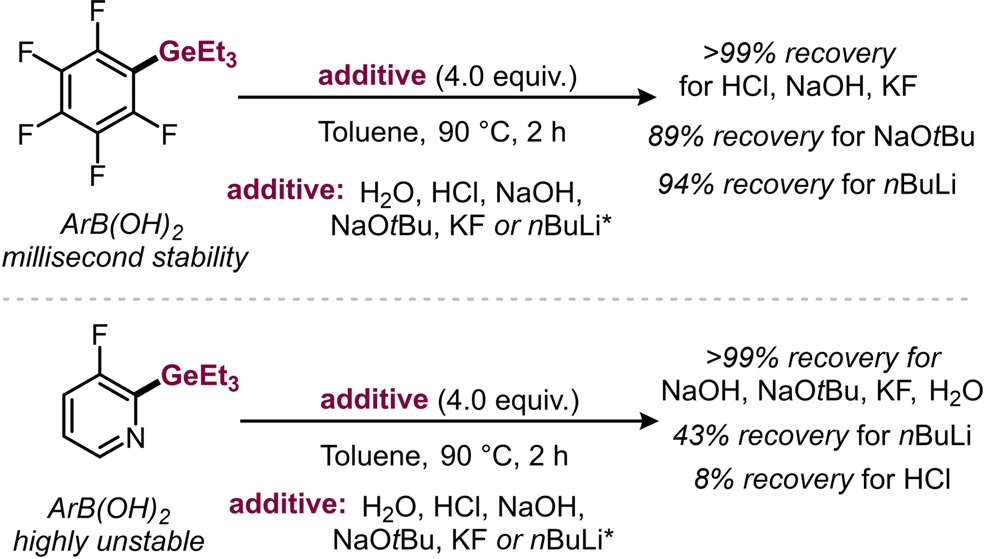
Stability tests with those ArGeEt_3_ reagents that are highly unstable as boronic acids. * *n*BuLi was used in 2.0–2.5 equiv.

We aspired to widen the conceptual coupling repertoire and focused on organogermanium compounds. Promisingly, no toxicity has been associated with this compound class,^19^ and our stability tests of a pentafluoroaryl germane (ArGeEt_3_, Ar=C_6_F_5_) indicated that as opposed to the corresponding boronic acid, which has a lifetime of milliseconds,^13^, ^16^ ArGeEt_3_ remains completely stable even upon subjection to acid (HCl) or base (NaOH, KF) for 2 h at 90 °C (Figure 2).^20^ Similarly the 2‐pyridyl derivative proved to be fairly stable under basic conditions but was sensitive to acid.

### Mechanistic Tests for Potential Reactivity with Pd^II^ and Mechanistic Support of Nanoparticle Reactivity

**Figure 3:**
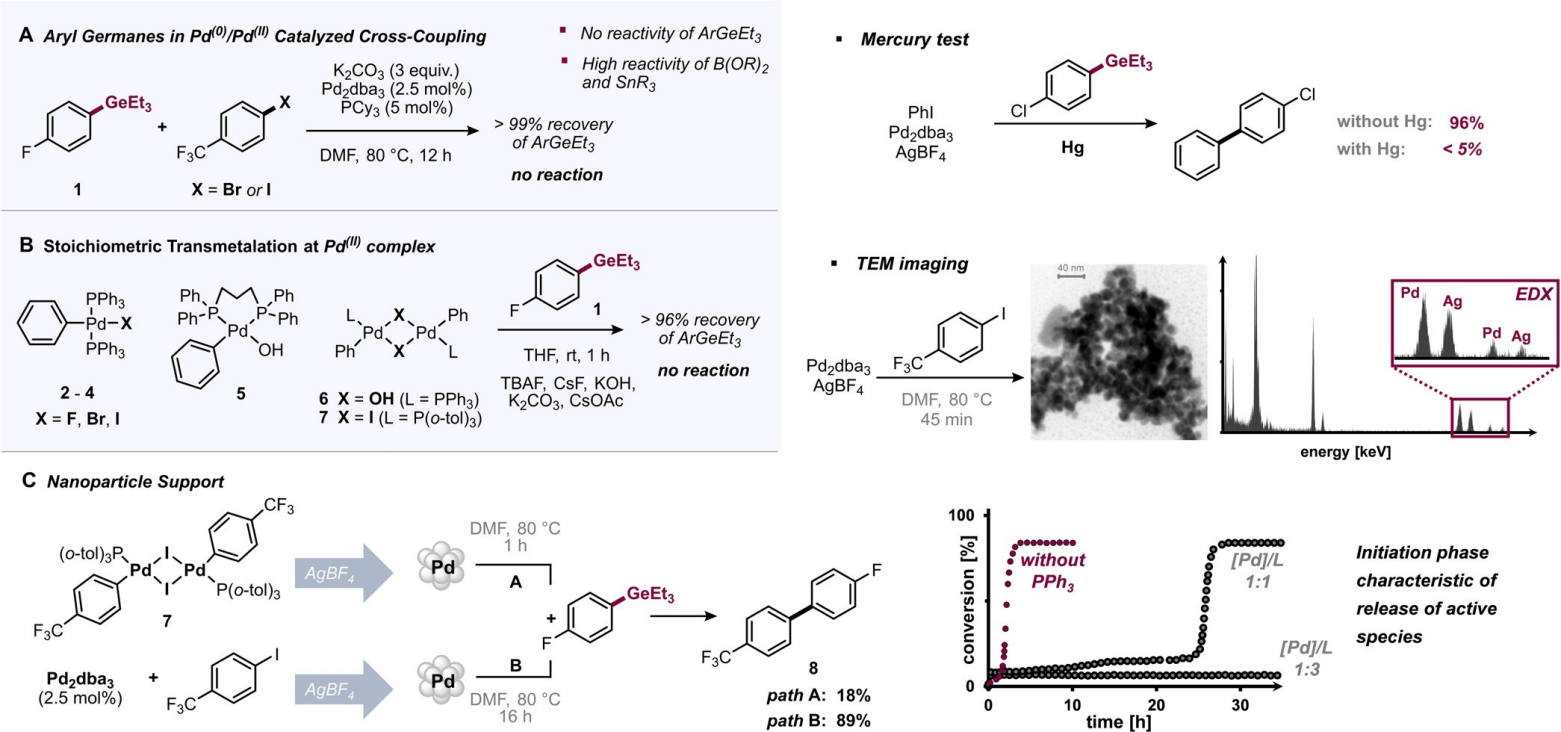
Mechanistic studies with aryl germanes and support of nanoparticle catalysis. A) Aryl germanes are unreactive under homogeneous catalysis. B) Aryl germanes are unreactive in the transmetalation of Pd^II^ complexes. C) Support of nanoparticle‐triggered reactivity (reactivity, imaging, mercury test, characteristic initiation phase in the reaction profile).

The few reported Pd‐catalyzed cross‐coupling reactions involving organogermanium compounds ascribed relatively low reactivity to the latter as compared to the established coupling partners, and coupling attempts exclusively applied basic and relatively harsh conditions without any detailed mechanistic interrogation.^21^, ^22^, ^23^, ^24^ We envisioned that a detailed investigation of the fundamental aspects of the coupling process involving organogermanium compounds might likely offer inspiration. In this context, we initially probed the potential of defined homogeneous Pd^0^/Pd^II^ coupling cycles and synthesized a variety of Pd^II^ complexes of the nature L_*n*_Pd^II^(X)(Ar), in which “X” was a halide or hydroxide. Upon subjection of phenyltriethylgermanium to the well‐established mono‐, bis‐, and bidentate phosphine‐coordinated Pd^II^ complexes **2**–**7** at room temperature or 80 °C, we saw no indication of transmetalation taking place, regardless of the coordinated halide (I, Br, F) or hydroxide, the employed solvent (THF, DMF, toluene), or additive (TBAF, CsF, KOH, or K_2_CO_3_) (Figure 3 B). In particular, Pd^II^−F complexes are usually privileged intermediates that typically undergo direct transmetalation with the established cross‐coupling partners ArSiR_3_, ArSnR_3_, or ArB(OH)_2_ without the need for additives.^12^, ^25^ Indeed, while the organogermane remained untouched (Figure 3), our comparative studies showed that organoboron reagents react with the same Pd^II^−F complex within seconds, and organosilane and organotin reagents within an hour at room temperature (see the Supporting Information, Table S1). These data clearly reinforce that typical Pd^0^/Pd^II^ reactivity modes are not readily amenable to organogermanium species.

Interestingly, although Pd^II^–iodide complex **7** did not give rise to any transformation of the organogermane, upon addition of one equivalent of AgBF_4_ at room temperature, the cross‐coupled product **8** was generated in 18 % yield (Figure 3 C). We next tested whether Pd^II^ complex **7** in conjunction with AgBF_4_ could also trigger the catalytic conversion of organogermanes. Using 2.5 mol % loading of **7** with AgBF_4_ indeed gave efficient catalytic transformation of 1‐iodo‐4‐(trifluoromethyl)benzene with 4‐fluorophenyl triethylgermane **1** to yield biaryl **8**. Given the rather labile nature of the Pd^II^ complex **7** as well as the visible metal precipitation upon AgBF_4_ addition, we speculated that palladium nanoparticles might be generated under these conditions and hence might also be involved in the coupling process. We therefore next adopted conditions that are known to generate nanoparticles and used Pd_2_dba_3_ along with AgBF_4_ (Figure 3 C).^26^ This resulted in efficient coupling of 1‐iodo‐4‐(trifluoromethyl)benzene with organogermane **1** under these conditions, and we isolated biaryl **8** in 89 % yield after 16 h at 80 °C.

Further support of nanoparticle catalysis was gained through the following experiments and observations: 1) Our analysis of the mixture through TEM imaging revealed the presence of spherical particles of approximately 5 nm diameter. EDX composition analysis showed the presence of both Pd and Ag in these nanoparticles. 2) The addition of mercury to this successful catalytic reaction resulted in complete inhibition; there was no significant product formation (≤5 %; Figure 3 C). These results are in accord with trapping and deactivation of the active nanoparticles.^27^ Moreover, 3) when we monitored the formation of 4‐methyl‐4′‐(trifluoromethyl)‐1,1′‐biphenyl as well as the consumption of the starting materials over time, we observed a brief induction period (of 90 min). This induction period was found to significantly prolong to 27 h in the presence of added ligand (5 mol % PPh_3_). These observations are common indicators that the true active species is phosphine‐free and is formed during the initial induction phase. In this context, our further experimentation revealed that the induction period and hence the formation of active species is independent of the aryl germane (see the Supporting Information for additional details).

### Exploration of Synthetic Potential

In light of these results, that is, the lack of reactivity of the organogermane with homogeneous Pd^II^ complexes, but high reactivity under [Pd] nanoparticle catalysis, we anticipated that there could be significant potential towards maximizing diversity in cross‐coupling and hence set out to explore the potential of catalytic cross‐coupling with organogermanes in greater detail.

**Figure 4:**
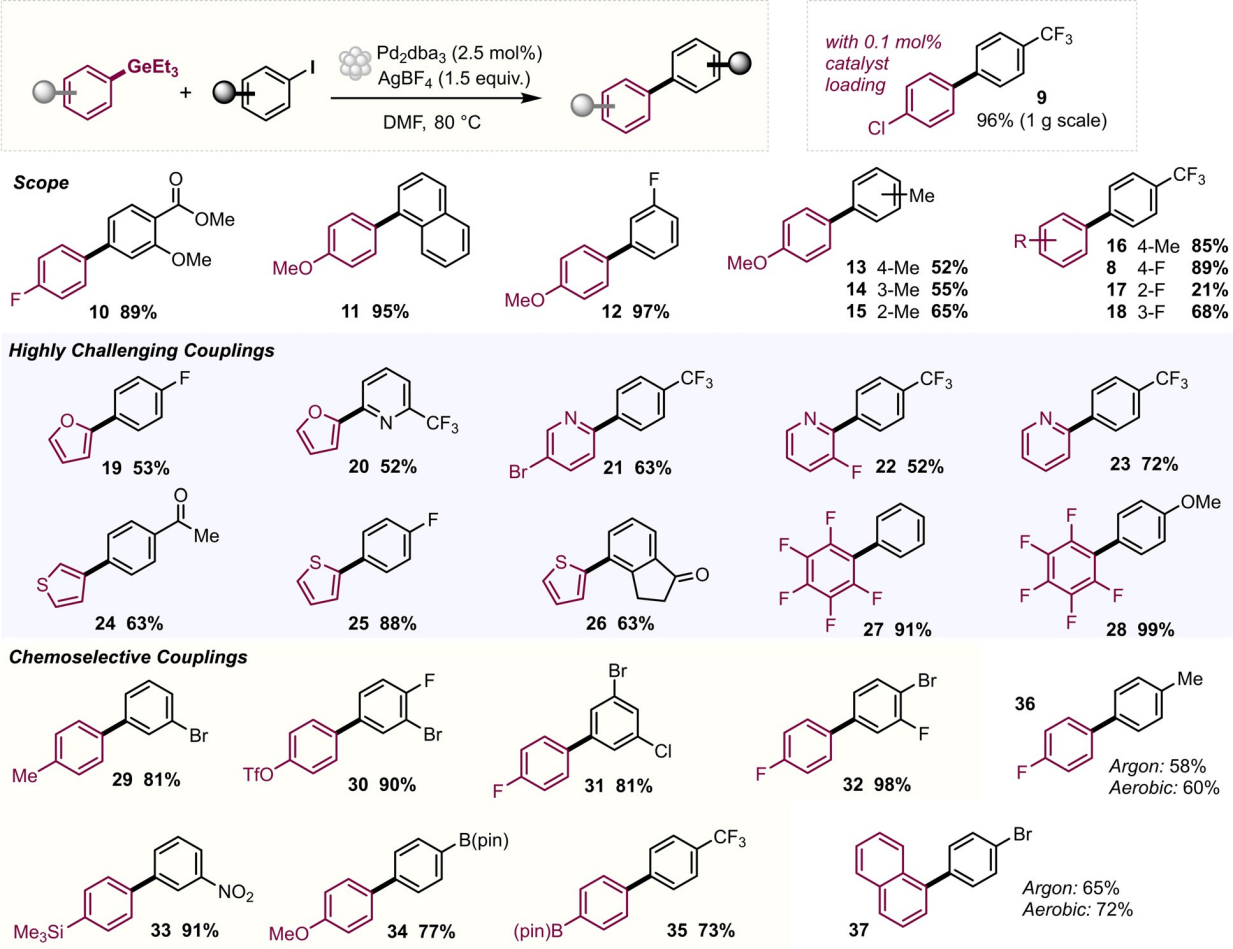
Scope of the C(sp^2^)−C(sp^2^) cross‐coupling reaction, including challenging and chemoselective couplings. Conditions: Aryl germane (1.0 equiv), aryl iodide (1.5 equiv) in DMF (0.3 m). The reactions were run for 16 h but can also be performed on much shorter timescales (see the main text).

Pleasingly, when we applied these nanoparticle conditions to both electron‐rich and electron‐poor aryl iodides with a variety of aryl germanes and stirred the mixture overnight, we obtained excellent yields of the corresponding biaryl products (**8**–**18**; Figure 4). Alkyl, ester, methoxy, or fluorinated groups were well tolerated, and all electronic combinations of biaryl (i.e., electron‐rich/‐rich, electron‐poor/electron‐rich, or electron‐poor/‐poor) could be prepared. Notably, the coupling was not affected by oxygen or moisture as the same coupling results were obtained under inert conditions or when the reaction was run in an open flask (see **36**, **37** in Figure 4).

### Major Coupling Challenges

With these successful conditions in hand, we subsequently set out to couple those moieties that are known to be tremendously challenging in the widely employed Suzuki cross‐coupling, that is, polyfluorinated arenes (**27** and **28**) and the medicinally and pharmaceutically relevant heterocycles thiophene, furan, and pyridine (**19**–**26**). The case of pentafluorophenylboronic acid has long been a challenge because of its propensity to undergo protodeboronation,^13^ typically requiring “designer” conditions.^16^

To our delight, when we performed the coupling of triethyl(pentafluorophenyl)germane with aryl iodides, we obtained near‐quantitative yields (**27**, **28**). Moreover, good yields were obtained also for heterocyclic variants, that is, the 2‐ and 3‐germylated thiophene (**24**–**26**) or furan (**19**, **20**) reagents. Even the most challenging substrates, 2‐pyridyl germanes, proved to be robust and stable and allowed for efficient cross‐couplings (**21**–**23**, **39**, **40**). The coupling of 2‐pyridylboronic acids has been a long‐standing challenge because of their inherent instability. Burke and co‐workers recently developed a solution through the use of MIDA boronate derivatives,^15^ which however requires additional protection/deprotection steps.

### Exploration of C−I versus C−Br/C−Cl Chemoselectivity

Another pertinent challenge in the cross‐coupling arena is site‐selective bond formation.^28^, ^29^ Chemoselective coupling strategies are of widespread interest as they provide access to densely functionalized biaryl motifs and enable the rapid creation of diversely substituted compound libraries. For poly(pseudo)halogenated arenes, typical L_*n*_Pd^0^/L_*n*_Pd^II^‐based coupling protocols generally suffer from low predictability of the favored coupling site and a pronounced substrate specificity. By utilizing Pd^I^ dimers or cationic Pd trimers, predictable site‐selective functionalizations were recently achieved with basic Grignard or organozinc reagents.^28^, ^29^ Pleasingly, our mild and base‐free conditions involving organogermanes allowed for C−I selective functionalization with multiply halogenated substrates, bearing bromide and chloride as well as the pseudo‐halogen OTf functionalities (**29**–**32**; Figure 4).

### Exploration of Practicability

**Figure 5:**
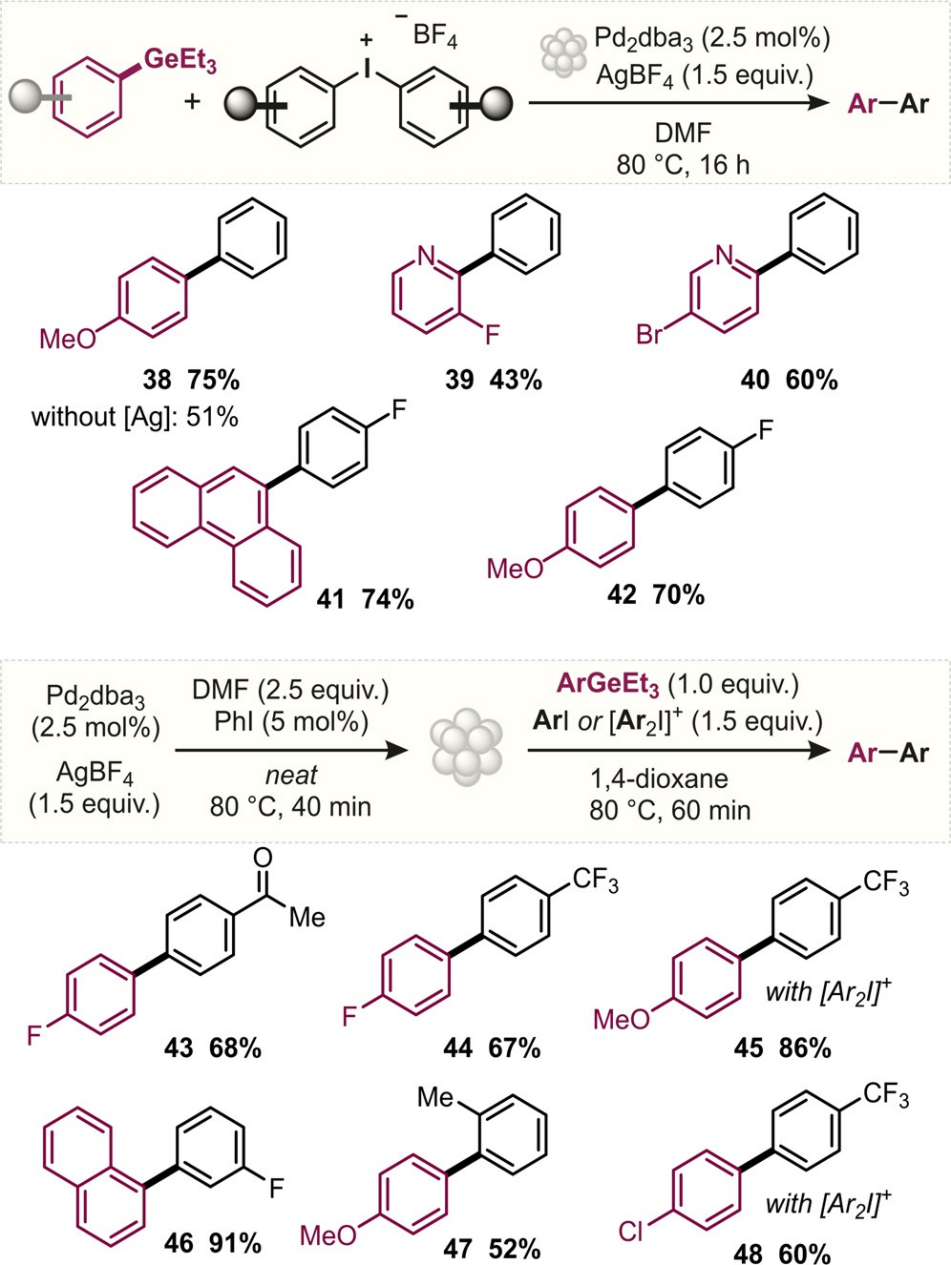
Scope of the C(sp^2^)−C(sp^2^) cross‐coupling with iodonium reagents and/or under preformed nanoparticle conditions.

With the exquisite synthetic potential of nanoparticle‐catalyzed couplings of aryl germanes showcased, we next assessed practical and sustainability features of the reaction for its wider applicability. While 2.5 mol % of the Pd source was employed in the above experiments, our tests indicated that the transformation also proceeds at a significantly lower loading of [Pd] and is scalable: Using 0.1 mol % of Pd_2_(dba)_3_, biaryl product **9** was efficiently prepared on a scale of about 1 g and 96 % yield. Aside from catalyst loading and scalability, the reaction medium and time will also influence wider applications, especially in an industrial context. To this end, a closer examination of the required reaction time revealed that much shorter times are sufficient and alternative solvents can be utilized. Only a small amount of DMF was found to be necessary for the formation of the active nanoparticle. As such, pre‐stirring of catalytic amounts of Pd_2_dba_3_ with (potentially sacrificial) iodobenzene (2.5 and 5 mol %, respectively), AgBF_4_ (1.5 equiv) with little DMF (2.5 equiv) for 40 min at 80 °C was found to be sufficient and then allowed the rapid coupling of an aryl germane with an aryl iodide within 1 h in dioxane in good yields (**43**–**48**; Figure 5).

### Tests of Silver‐Free Reactivity and Hypervalent Iodine Reagents

Our mechanistic data indicated that the primary role of silver was as an iodide scavenger. As such, we envisioned that hypervalent iodine compounds might also be effective in the coupling of organogermanium compounds as they are inherently more activated. Indeed, we found that diaryliodonium salts function as complementary electrophiles, even for the exceptionally challenging 2‐pyridyl substrates (**38**–**42**; Figure 5). Both the BF_4_ and PF_6_ iodonium salts were shown to be effective. Notably, the coupling of organogermanes with diaryliodonium salts is also effective in the absence of AgBF_4_. For example, when a 4‐methoxyphenylgermane was reacted with a diphenyliodonium salt in the presence of Pd_2_(dba)_3_ (2.5 mol %) in DMF for 2 h at 80 °C, 51 % of biaryl product **38** was isolated. In this context, we unambiguously confirmed the formation of nanoparticles under silver‐free reaction conditions with diaryliodonium salts (see the Supporting Information for further information and TEM images).^30^ The ability to conduct these reactions in a silver‐free fashion suggests that [Pd] instead of [Ag] is the key active component in the nanoparticles that allow for couplings of aryl germanes.

### Are Aryl Germanes Truly Privileged with Nanoparticles?

**Figure 6:**
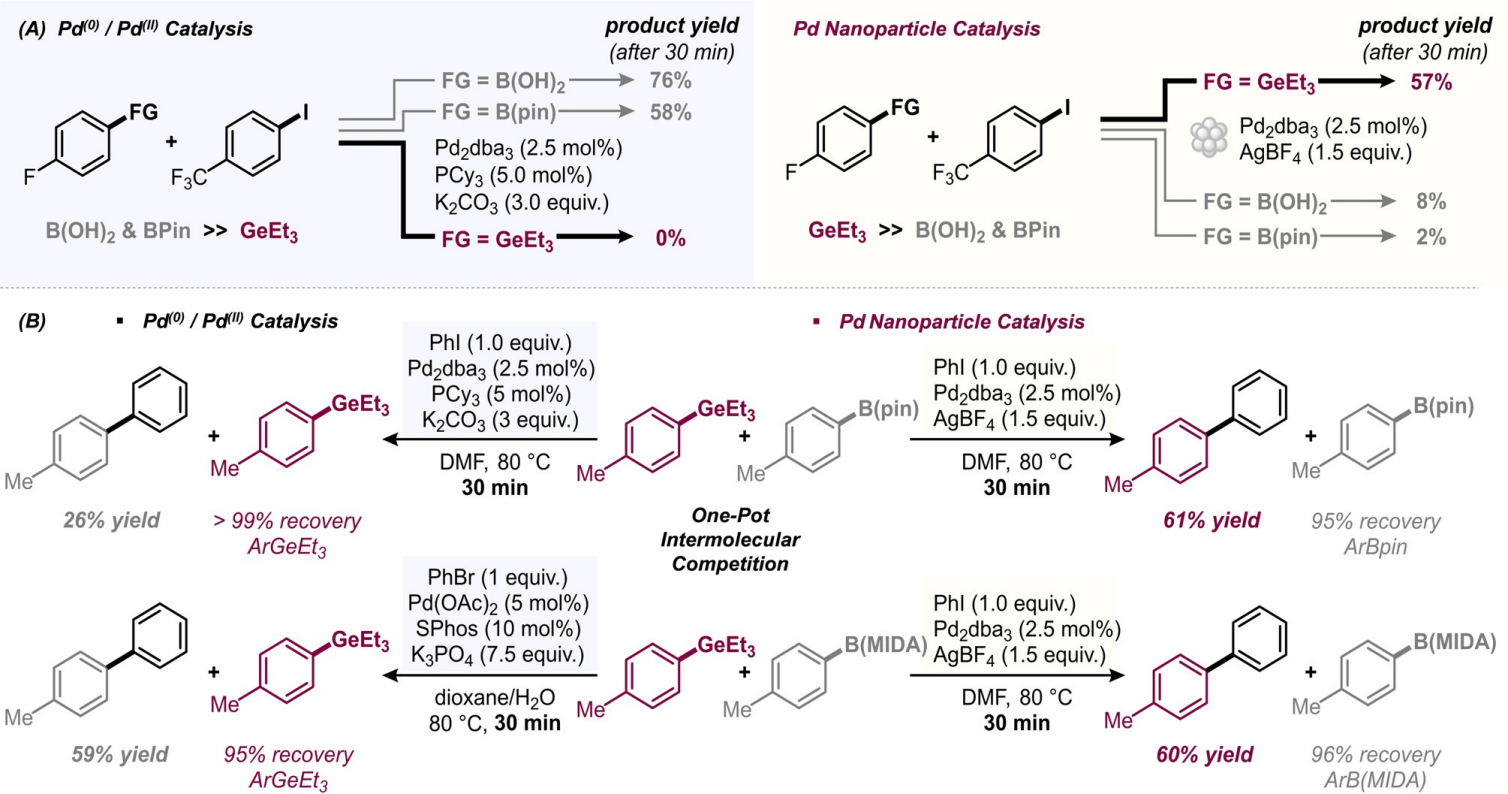
A) Performance of ArGeEt_3_ versus established coupling agents in couplings with PhI under Pd^0^/Pd^II^ catalysis (left) and nanoparticle catalysis (right). B) Intermolecular competitions in the coupling of PhI with ArGeEt_3_ versus ArB(Pin) or ArB(MIDA) under Pd^0^/Pd^II^ molecular catalysis (left) and nanoparticle catalysis (right). Reaction times: 30 min.

With the synthetic potential of the nanoparticle‐catalyzed coupling of aryl germanes with aryl iodides or hypervalent iodine reagents established, we next assessed whether the aryl germanes are truly privileged in these transformations. To this end, we subjected established transmetalation agents, that is, *para*‐fluorophenylboronic acid and the pinacol boronic ester thereof to the nanoparticle‐catalysis conditions and attempted to couple 1‐iodo‐4‐(trifluoromethyl)benzene (Figure 6 A, right). While the corresponding ArGeEt_3_ reagent delivered the coupling product **8** in 57 % yield after 30 min, the other transmetalating agents failed to deliver the coupling product in appreciable amounts and gave **8** in only 2–8 % yield.

As such, there is a profound selectivity reversal from traditional molecular Pd^0^/Pd^II^ versus [Pd] nanoparticle conditions. While the aryl germane proved to be the least reactive (=unreactive) under classical molecular L_*n*_Pd^0^/L_*n*_Pd^II^ cross‐coupling conditions as compared to the established cross‐coupling partner (Figure 6 A), it becomes the most reactive under nanoparticle conditions. Consequently, selective couplings should also have potential. Indeed, the intramolecular competition of silane‐ and Bpin‐substituted aryl germanes in the coupling with 4‐iodophenylboronic acid pinacol ester gave exclusive coupling at the C−Ge site (**33**–**35**, see Figure 4). Moreover, the intermolecular competition between ArGeEt_3_ versus ArB(Pin) or ArB(MIDA) in the coupling with iodobenzene also showed orthogonal selectivities (Figure 6 B), offering therefore an orthogonal tool for selective C(sp^2^)−C(sp^2^) coupling reactions and an additional mode to increase diversity.

### Computational Studies on the Origins of Reactivity

**Figure 7:**
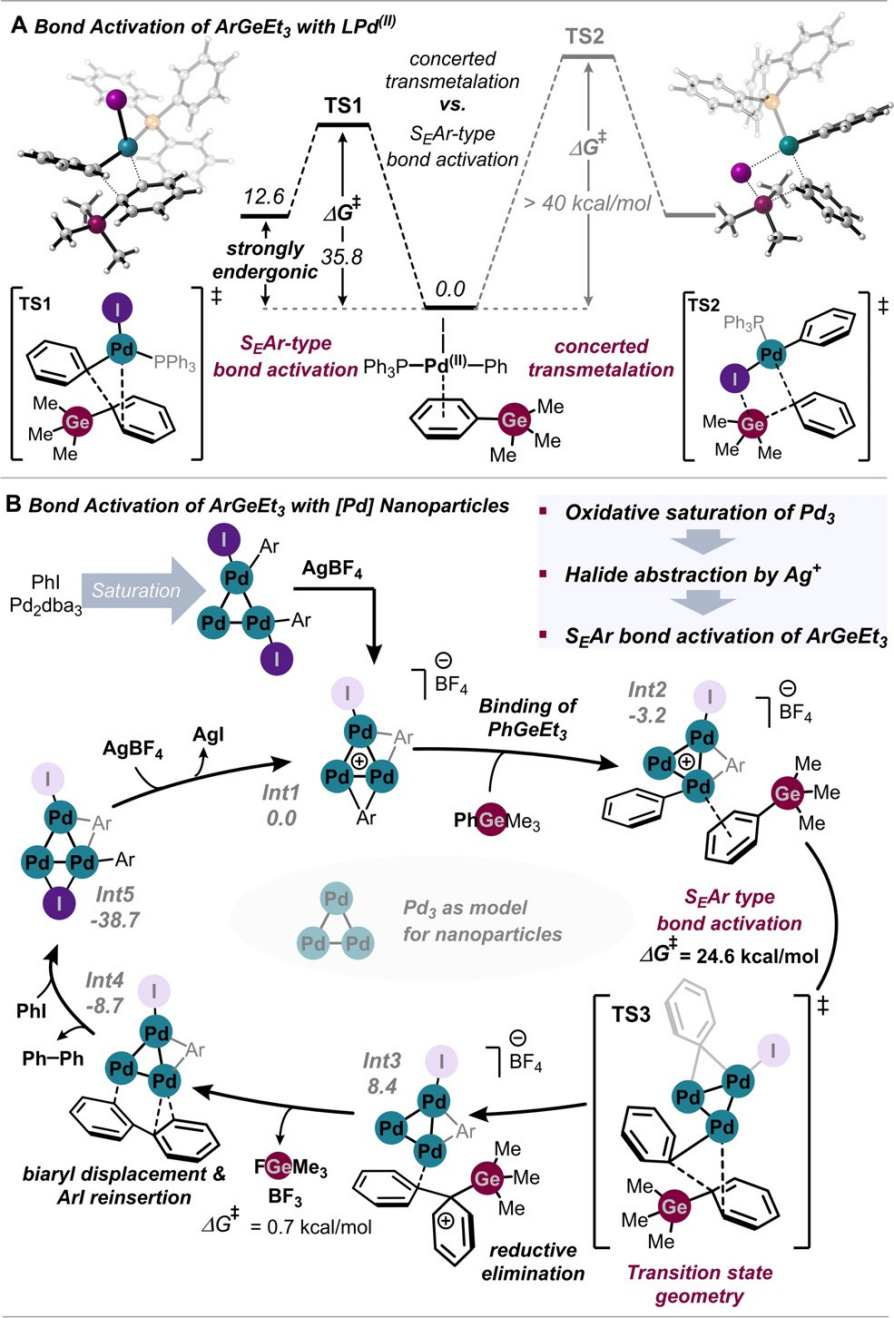
Computational study of the nanoparticle‐catalyzed cross‐coupling; free energy diagram in kcal mol^−1^, calculated at the CPCM (DMF) B3LYP‐D3/Def2TZVPP//B3LYP‐D3/Def2SVP level of theory.^33^

To gain insight into the origins of orthogonality, we undertook computational studies at the CPCM (DMF) B3LYP‐D3/Def2TZVPP//B3LYP‐D3/Def2SVP level of theory.^31^ We initially investigated why organogermanes are not reactive in the transmetalation of defined L_*n*_Pd^II^ complexes. Interestingly, we found that the generally assumed concerted four‐centered transmetalation of PhGeMe_3_ with [(PPh_3_)Pd^II^(X)(Ph)] with X=F or I is significantly disfavored (Δ*G* ^≠^>40 kcal mol^−1^; Figure 7 A), which appears to be due to a lack of driving force to form a [Ge]–halogen bond. Our search for alternative modes of activation revealed that electrophilic aromatic substitution (S_E_Ar) constitutes a lower‐energy pathway for transmetalation at Pd^II^ (see **TS2**, Figure 7 A), which is characterized by an activation free energy of Δ*G* ^≠^=35.8 kcal mol^−1^ for X=I. While this barrier is still rather high, these results indicate that organogermanes appear to be more prone to react as nucleophiles and hence should prefer more electrophilic and electron‐deficient metal species rather than ligand‐coordinated Pd^II^ complexes.

We next set out to study the molecular events under nanoparticle catalysis. Building on previous studies on the likely speciation of nanoparticles,^32^ we used a phosphine‐free Pd trimer as a representative model for the active nanoparticle. We studied the likely full catalytic cycle and investigated numerous possibilities, of which the favored pathway is featured in Figure 7 B.

In the experimentally observed initiation phase involving Pd, silver, and the aryl iodide, a palladium cluster is likely formed and stabilized through oxidative saturation of aryl iodide. Our computational data suggest that addition of two molecules of PhI to the Pd cluster is highly exergonic and favored over coordination of an aryl germane. The saturated Pd_3_ intermediate is likely activated by Ag^+^, forming a cationic cluster (**Int1**) and releasing AgI. Alternatively, **Int1** is formed directly with diaryliodonium salts (in the absence of silver salts). Subsequent coordination of aryl germane to **Int1** is now energetically favored over ArI. The key C−Ge bond activation then takes place from **Int2**, and was found to proceed by an S_E_Ar‐type mechanism via **TS3**. The activation free energy barrier for the C−Ge bond cleavage is 24.6 kcal mol^−1^ and as such significantly lower than that of S_E_Ar‐type transmetalation at a Pd^II^ complex.^33^ From **Int3** the release of [GeMe_3_]^+^ and formation of the biaryl is very facile.

Importantly, although we considered a trimer as the model for nanoparticles, our computational data suggest that these reactivity trends also hold for larger Pd clusters. As such, the computational studies suggest that bond activation under nanoparticle catalysis is reminiscent of an electrophilic aromatic substitution. Owing to its electron richness the aryl germane is a privileged reaction partner with electron‐deficient Pd species, which is the origin of its superior reactivity with electrophilic Pd nanoparticles and the lack of reaction under L_*n*_Pd^0^/L_*n*_Pd^II^ catalysis.

## Conclusion

We have developed a chemoselective coupling of aryl iodides (and diaryliodonium salts) with aryl germanes under nanoparticle catalysis in the presence of C−Br, C−Cl, C−BPin, C−BMIDA, and additional functional groups. The method is characterized by operational simplicity, air tolerance, and robustness and can be performed at low Pd loadings. The aryl germanes were shown to be highly stable. For example, a pentafluoroarylgermane tolerates strong acids or bases over extended times and at elevated temperature, whereas the corresponding boronic acid has a lifetime of milliseconds only. As such, highly challenging couplings can readily be performed with aryl germanes, including those involving 2‐pyridyl or polyfluoroaryl germanes. Mechanistic and computational data are presented that unambiguously demonstrate that while organogermanes are the least reactive functionality under Pd^0^/Pd^II^ homogeneous molecular catalysis as compared to established coupling partners, they are the most reactive group under nanoparticle conditions. The origin of this privileged reactivity was found to lie in the electron richness of the aryl germanes, which preferentially react by an electrophilic aromatic substitution type mechanism and as such are preferentially activated by more electrophilic nanoparticles. These features in turn allow to position organogermanes as an orthogonal coupling motif to the currently established and omnipresent cross‐coupling regimes, and showcase truly distinguished reactivity of nanoparticles as compared to homogeneous molecular metal catalysts.

## Conflict of interest

The authors declare no conflict of interest.

## Supporting information

As a service to our authors and readers, this journal provides supporting information supplied by the authors. Such materials are peer reviewed and may be re‐organized for online delivery, but are not copy‐edited or typeset. Technical support issues arising from supporting information (other than missing files) should be addressed to the authors.

Supplementary
