# Supplementary material for: Orthogonal Nanoparticle Catalysis with Organogermanes
Source: Angew Chem Int Ed Engl. 2019 Oct 23;58(49):17788–95. doi: 10.1002/anie.201910060 (PMC6899604; doi:10.1002/anie.201910060)
Supplement: Supplementary file 1 — Supplementary [file ANIE-58-17788-s001.pdf]

Angewandte Chemie
Eine Zeitschrift der Gesellschaft Deutscher Chemiker

Supporting Information

Orthogonal Nanoparticle Catalysis with Organogermanes

*Christoph Fricke[+], Grant J. Sherborne[+], Ignacio Funes-Ardoiz, Erdem Senol, Sinem Guven, and Franziska Schoenebeck**

anie_201910060_sm_miscellaneous_information.pdf

Table of Contents

1 Materials and Methods 4

2 Experimental Procedures 6

2.1 Synthesis of Aryl Germanes 6

2.1.1 General Procedures 6

2.2 Characterization of Aryl Germanes 7

2.3 Synthesis of Diaryliodonium Salts 15

2.4 Cross Coupling Reactions 17

2.4.3 Reaction with Diaryl Iododonium Salts 30

2.4.4 Gram Scale Reaction 33

2.4.5 Preformation of Nanoparticles/Cross Coupling 34

2.4.6 Intramolecular competition 36

3 Stoichiometric Bond Activation with $Pd^{(II)}$ 38

3.1 Preparation of $Pd^{(II)}$ Complexes 38

3.2 Stoichiometric Bond Activation of Classical Reagents 42

3.3 Stoichiometric Bond Activation of Aryl Germanes 43

3.3.1 $Pd^{(II)}$-Halide Complexes 43

3.3.2 $Pd^{(II)}$-Hydroxo Complexes 44

3.3.3 $Pd^{(II)}$-Iodo Dimer 45

4 Reaction Development 46

5 Mechanistic Investigations 47

5.1 Investigation of Induction Period with Various [Pd]/L Ratios 47

5.2 Investigation of Induction Period with and Without Premixing of Reagents 48

5.2.1 No Premixing of Reagents 48

5.2.2 Premixing of Aryl Iodide, $AgBF_4$ and Pd_2dba_3 48

5.3 Mercury Test 49

5.4 Stability Test 50

5.4.1 Stability of Triethyl(perfluorophenyl)germane 50

5.4.2 Stability of Triethyl(3-fluoropyridyl)germane 51

5.4.3 Stability of 4-Tolylboronic acid MIDA ester 52
5.5 TEM Analysis 53
5.5.1 Premixing of Aryl Iodide, $AgBF_4$ and Pd_2dba_3 53
5.5.2 Premixing of Diaryl Iodoniumsalt and Pd_2dba_3 (Silver-Free Conditions) 54
5.5.3 Standard Reaction conditions for Catalysis 55
6 $Pd^{(0)}/Pd^{(II)}$ *versus* Nanoparticle Catalysis 56
6.1 $Pd^{(0)}/Pd^{(II)}$ Molecular Catalysis 56
6.2 Intermolecular Competition – $Pd^{(0)}/Pd^{(II)}$ Molecular Catalysis 57
6.3 Nanoparticle Catalysis 58
6.4 Intermolecular Competition – Nanoparticle Catalysis 59
6.5 Compatibility of $ArGeEt_3$ to Orthogonal Coupling Reaction with ArB(MIDA) 60
7 Computational Details 61
7.1 General Computational Details 61
7.2 Oxidative Saturation of Pd_3 Nanocluster with Aryl Iodide 62
7.3 Homocoupling of Aryl Iodide *vs.* Organogermane S_EAr 62
7.4 Bond Activation of $ArGeEt_3$ with BF_4^- Counterion 63
7.5 XYZ Coordinates and Energies for Optimized Structures 64
8 NMR Spectra 85
9 References 115

1 Materials and Methods

Reagents and Solvents

Unless otherwise stated, all reagents and starting materials were commercially available and used as received. Anhydrous and degassed THF, diethyl ether (Et_2O), toluene and hexane were obtained using an Innovative Technology PS-MD-5 solvent purification system. Solvents used in work up and purification were distilled prior to use. Anhydrous and degassed dimethylformamide (DMF), acetone and benzene were purchased from Sigma Aldrich.

Experimental Techniques

All reactions involving air- or moisture-sensitive reagents or intermediates were carried out in dried glassware under an argon atmosphere and were performed either in an argon-filled glovebox or by using standard *Schlenk* techniques unless otherwise stated.

Column chromatography was carried out using silica gel (35–70 mesh; 60 Å). Thin layer chromatography (TLC) was performed on Merck silica gel 60 F254 aluminium plates; detection either under UV light or by dipping into a solution of $KMnO_4$ (1.5 g) and $NaHCO_3$ (5.0 g) in H_2O (400 mL) followed by heating. Preparative HPLC was performed on a Gilson-Abimed HPLC (employing UV detector model 117) using a Merck LiChrosorb Si60 column (porosity 7 µm, 250 x 25 mm).

All ^{1}H, $^{13}C\{^{1}H\}$ and $^{19}F\{^{1}H\}$ and ^{31}P NMR spectra were recorded at ambient temperature on a Varian V-NMRS 600 or a Varian V-NMRS 400 spectrometer. Chemical shifts (δ) are reported in parts per million (ppm) relative to $SiMe_4$ and referenced to either the residual solvent peak for ^{1}H NMR (7.26 ppm for $CDCl_3$) and ^{13}C NMR spectra (77.2 ppm for $CDCl_3$) or internally by the instrument after locking and shimming to the deuterated solvent (^{19}F and ^{31}P NMR spectra). Coupling constants (*J*) are given in Hertz (Hz).

High-resolution mass spectrometry (HRMS) was performed using a Thermo Scientific LTQ Orbitrap XL (ESI) or an Finnigan MAT 95 (EI, 70 eV). Low-resolution mass spectrometry was performed with an Agilent Technologies 5975 series MSD mass spectrometer under electron ionization (EI) mode coupled with an Agilent Technologies 7820A gas chromatograph employing an Agilent HP-5MS column (30 m × 0.25 mm inner diameter× 0.25 µm (5% phenyl)-methylpolysiloxane film) or an Agilent CP-Sil8-CB column (30 m × 0.25 mm inner diameter × 1.00 µm (5% phenyl)-methylpolysiloxane film) Operating with a constant He-flow of 1.2 mL min^{-1}, injector temperature 250 °C, detector-line temperature 280 °C.

IR spectra were recorded on a Spectrum 100 spectrometer with an UATR Diamond/KRS-5 crystal with attenuated total reflectance (ATR). Relative intensities are given in parentheses (w = weak, m = medium, s = strong).

2 Experimental Procedures

2.1 Synthesis of Aryl Germanes

2.1.1 General Procedures

General Procedure 1 (GP 1)

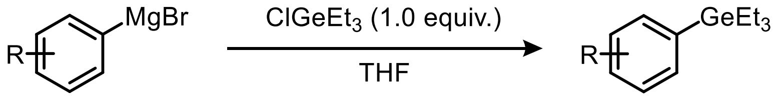

Triethylgermanium chloride (1.0 equiv.) and the corresponding aryl Grignard reagent (1.1 equiv.) were dissolved in anhydrous and degassed THF (0.2 M) under argon at 0 °C and stirred for 3 h, being allowed to warm to room temperature. The reaction was quenched by addition of aqueous solution of NH_4Cl (sat.), the organic phase was separated and the aqueous phase was extracted with DCM (3x). The combined organic phases were dried with $MgSO_4$, the solvent was removed under reduced pressure and the crude product mixture was purified by silica column chromatography.

General procedure 2 (GP 2)

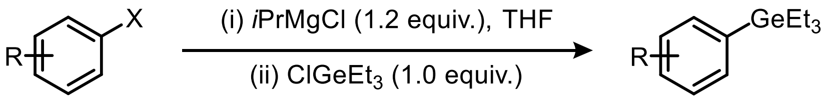

R = I *or* Br

Triethylgermanium chloride (1.05 equiv.) and the corresponding aryl iodide or aryl bromide (1.0 equiv.) were dissolved in THF (0.2 M) under argon, *i*PrMgCl (1.2 equiv.) was added slowly and the reaction was stirred for 3 h at room temperature (ArI) or for 12 h at 60 °C (ArBr). The reaction was quenched by addition of aqueous solution of NH_4Cl (sat.), the organic phase was separated and the aqueous phase was extracted with DCM (3x). The combined organic phases were dried with $MgSO_4$, the solvent was removed under reduced pressure and the crude product mixture was purified by silica column chromatography.

2.2 Characterization of Aryl Germanes

Triethyl(4-fluorophenyl)germane

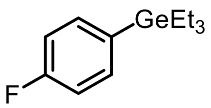

Prepared according to GP 1 using 4-fluorophenylmagnesium bromide (1.0 M in Et_2O, 3.3 mL, 3.3 mmol, 1.1 equiv.). The title product was obtained after purification by column chromatography (pentane) as a colorless oil (732 mg, 2.87 mmol, 96%).

R$_f$ = 0.85 (Pentane). **^{1}H NMR** (600 MHz, $CDCl_3$) δ/ ppm = 7.39 (dd, *J* = 7.9, 1.6 Hz, 2H), 7.05 (d, *J* = 8.8 Hz, 2H), 1.12 – 1.01 (m, 6H), 1.01 – 0.92 (m, 9H). **^{13}C NMR** (151 MHz, $CDCl_3$) δ/ ppm = 163.4 (d, *J* = 246.4 Hz), 135.7 (d, *J* = 6.9 Hz), 135.0 (d, *J* = 3.7 Hz), 115.1 (d, *J* = 19.1 Hz), 9.0, 4.4. **^{19}F NMR** (564 MHz, $CDCl_3$) δ/ ppm = -113.75 – -113.84 (m). **HRMS** (EI) calculated for $C_{12}H_{19}F^{74}Ge$: 256.0683 $[M]^+$, found: 256.0673. **IR** (neat): *ν*/ cm^{-1} = 3029 (w), 2949 (s), 2875 (s), 2332 (w), 2162 (w), 2092 (w), 1890 (w), 1752 (w), 1636 (w), 1584 (s), 1495 (s), 1459 (m), 1380 (w), 1304 (w), 1226 (s), 1160 (s), 1082 (m), 1014 (s), 965 (m), 816 (s), 698 (s). **Elemental analysis** calculated (%) for $C_{12}H_{19}FGe$: C 56.54, H 7.51; found: C 56.64, H 7.43.

Triethyl(4-chlorophenyl)germane

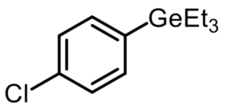

Prepared according to GP 1 using 4-chlorophenylmagnesium bromide (1.0 M in Et_2O, 11.0 mL, 11.0 mmol, 1.1 equiv.). The title product was obtained after purification by column chromatography (hexane) as a colorless oil (1.75 g, 6.45 mmol, 65%).

R$_f$ = 0.80 (Hexane). **^{1}H NMR** (600 MHz, $CDCl_3$) δ/ppm = 7.36 (d, *J* = 8.2 Hz, 2H), 7.31 (d, *J* = 8.2 Hz, 2H), 1.08 – 1.02 (m, 9H), 1.01 – 0.94 (m, 6H). **^{13}C NMR** (151 MHz, $CDCl_3$) δ/ppm = 138.2, 135.4, 134.5, 128.2, 9.0, 4.3. **HRMS** (EI) calculated for $C_{12}H_{19}{}^{35}Cl^{74}Ge$: 272.0387 $[M]^+$, found: 272.0385. **IR** (neat): *ν*/ cm^{-1} = 3070 (w), 2949 (s), 2873 (s), 2332 (w), 2150 (w), 2091 (w), 2030 (w), 1900 (w) 1637 (w), 1572 (m), 1468 (s), 1428 (m), 1378 (m), 1304 (w), 1229 (w), 1075 (s), 1012 (s), 966 (m), 806 (s), 699 (s). **Elemental analysis** calculated (%) for $C_{12}H_{19}GeCl$: C 53.11, H 7.06; found: C 53.74, H 7.06.

Triethyl(4-methoxyphenyl)germane

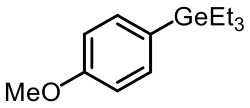

Prepared according to GP 1 using 4-methoxyphenylmagnesium bromide (1.0 M in Et_2O, 3.3 mL, 3.3 mmol, 1.1 equiv.). The title product was obtained after purification by column chromatography (pentane) as a colorless oil (785 mg, 2.94 mmol, 98%).

$\mathbf{R_f}$ = 0.42 (Pentane). **^{1}H NMR** (600 MHz, $CDCl_3$) δ/ ppm = 7.36 (d, *J* = 8.6 Hz, 2H), 6.91 (d, *J* = 8.6 Hz, 2H), 3.81 (s, 3H), 1.10 – 1.02 (m, 9H), 1.00 – 0.93 (m, 6H). **^{13}C NMR** (151 MHz, $CDCl_3$) δ/ ppm = 159.9, 135.2, 130.5, 113.8, 55.1, 9.1, 4.4. **HRMS** (EI) calculated for $C_{13}H_{22}{}^{74}GeO$: 268.0882 $[M]^+$, found: 268.0890. **IR** (neat): *ν*/ cm^{-1} = 2946 (s), 2326 (w), 2073 (w), 1887 (w), 1731 (w), 1590 (s), 1497 (s), 1457 (s), 1387 (w), 1276 (s), 1243 (s), 1178 (s), 1091 (s), 1024 (s), 964 (m), 810 (m), 697 (s). **Elemental analysis** calculated (%) for $C_{13}H_{22}GeO$: C 58.59, H 8.31; found: C 58.88, H 8.15.

Triethyl(*p*-tolyl)germane

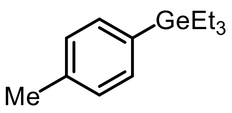

Prepared according to GP 2 using 4-methylphenylmagnesium bromide (1.0 M in THF, 3.3 mL, 3.3 mmol, 1.1 equiv.). The title product was obtained after purification by column chromatography (pentane) as a colorless oil (575 mg, 2.29 mmol, 76%).

$\mathbf{R_f}$ = 0.85 (Pentane). **^{1}H NMR** (600 MHz, $CDCl_3$) δ/ ppm = 7.35 (d, *J* = 7.6 Hz, 2H), 7.18 (d, *J* = 7.6 Hz, 2H), 2.36 (s, 3H), 1.11 – 1.03 (m, 9H), 1.01 – 0.95 (m, 6H). **^{13}C NMR** (151 MHz, $CDCl_3$) δ/ ppm = 137.8, 136.0, 133.9, 128.7, 21.4, 8.9, 4.2. **HRMS** (EI) calculated for $C_{13}H_{22}{}^{74}Ge$: 252.0933 $[M]^+$, found: 252.0926. **IR** (neat): *ν*/ cm^{-1} = 3016 (m), 2945 (s), 2732 (w), 2328 (m), 2087 (w), 2897 (m), 1738 (w), 1599 (w), 1455 (s), 1384 (m), 1228 (w), 1188 (w), 1086 (m), 1014 (s), 965 (m), 794 (s), 697 (s). **Elemental analysis** calculated (%) for $C_{13}H_{22}Ge$: C 62.22, H 8.84; found: C 61.32, H 8.50.

Triethyl(2-fluorophenyl)germane

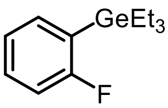

Prepared according to GP 2 using 2-fluoroiodobenzene (0.35 mL, 3.0 mmol, 1.00 equiv.). The title product was obtained after purification by column chromatography (pentane) as a colorless oil (527 mg, 2.07 mmol, 69%).

$\mathbf{R_f}$ = 0.48 (Pentane). **^{1}H NMR** (600 MHz, $CDCl_3$) δ/ ppm = 7.38 – 7.29 (m, 2H), 7.16 – 7.07 (m, 1H), 7.04 – 6.87 (m, 1H), 1.08 – 1.02 (m, 15H). **^{13}C NMR** (151 MHz, $CDCl_3$) δ/ ppm = 166.9 (d, *J* = 238.7 Hz), 135.6 (d, *J* = 12.7 Hz), 130.5 (d, *J* = 7.9 Hz), 125.3 (d, *J* = 35.8 Hz), 123.9 (d, *J* = 2.9 Hz), 114.7 (d, *J* = 26.5 Hz), 9.0, 4.7. **^{19}F NMR** (564 MHz, $CDCl_3$) δ/ ppm = -99.8 (q, *J* = 6.3 Hz, 1F). **HRMS** (EI) calculated for $C_{12}H_{19}F^{74}Ge$: 256.0677 $[M]^+$, found: 256.0675. **IR** (neat): *ν*/ cm^{-1} = 3066 (s), 2953 (m), 1580 (m), 1465 (w), 1436 (w), 1382 (s), 1256 (w), 1203 (s), 1110 (s), 1066 (s), 1017 (s), 970 (s), 816 (s), 757 (s), 704 (s) 579 (s) 542 (s). **Elemental analysis** calculated (%) for $C_{12}H_{19}FGe$: C 56.54, H 7.51; found: C 56.33, H 7.59.

Triethyl(3-fluorophenyl)germane

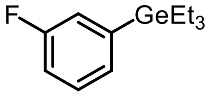

Prepared according to GP 2 using 3-fluoroiodobenzene (0.35 mL, 3.0 mmol, 1.00 equiv.). The title product was obtained after purification by column chromatography (pentane) as a colorless oil (726 mg, 2.85 mmol, 95%).

R_f = 0.59 (Pentane). **^{1}H NMR** (400 MHz, $CDCl_3$) δ/ ppm = 7.37 – 7.29 (m, 1H), 7.23 – 7.18 (m, 1H), 7.18 – 7.10 (m, 1H), 7.06 – 6.93 (m, 1H), 1.45 – 0.49 (m, 15H). **^{13}C NMR** (101 MHz, $CDCl_3$) δ/ ppm = 162.8 (d, *J* = 248.6 Hz), 143.1 (d, *J* = 3.6 Hz), 129.6 (d, *J* = 2.9 Hz), 129.5 (d, *J* = 6.8 Hz), 120.4 (d, *J* = 18.2 Hz), 115.1 (d, *J* = 21.1 Hz), 8.9, 4.2. **^{19}F NMR** (376 MHz, $CDCl_3$) δ/ ppm = -114.0 (q, *J* = 8.1 Hz, 1F). **HRMS** (EI) calculated for $C_{12}H_{19}F^{74}Ge$: 256.0677 $[M]^+$, found: 256.0677.**IR** (neat): *ν*/ cm^{-1} = 2908 (m), 2326 (w), 2091 (w), 1928 (w), 1575 (s), 1470 (s), 1412 (s), 1257 (s), 1211 (s), 1161 (s), 1095 (s), 1015 (s), 968 (s), 895 (s), 862 (s), 781 (s), 693 (s). **Elemental analysis** calculated (%) for $C_{12}H_{19}FGe$: C 56.54, H 7.51; found: C 56.45, H 7.64.

Triethyl(furan-2-yl)germane

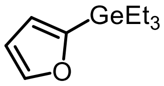

Furan (500 µl, 6.9 mmol, 1.4 equiv.) was dissolved into anhydrous Et_2O (2 mL) in a round-bottom flask under a flow of argon. The solution was cooled to -78 °C and *n*-BuLi (2.5 M in hexane, 1.96 mL, 4.9 mmol, 1.0 equiv.) was added dropwise and stirred at -78 °C for 30 minutes. The mixture was warmed to room temperature for a further 90 minutes before cooling back to -78 °C. Et_3GeCl (957 µl, 4.9 mmol, 1.0 equiv.) was added slowly and the reactions was gradually warmed to room temperature before stirring overnight. The reaction was quenched with 5 mL aqueous NH_4Cl (sat.), diluted with 20 mL H_2O and extracted with 3x30 mL Et_2O. The title product was obtained after column chromatography (pentane) as a colorless oil (823 mg, 3.6 mmol, 74%).

R_f = 0.43 (Hexane). **^{1}H NMR** (600 MHz, $CDCl_3$) δ/ ppm = 7.67 (d, *J* = 1.8 Hz, 1H), 6.54 (d, *J* = 3.3 Hz, 1H), 6.40 (dd, *J* = 3.3, 1.8 Hz, 1H), 1.10 – 1.06 (m, 9H), 1.02 – 0.96 (m, 6H). **^{13}C NMR** (151 MHz, $CDCl_3$) δ/ ppm = 159.1, 146.2, 118.7, 109.1, 77.2, 77.0, 76.8, 8.9, 4.6, 4.4. **HRMS** (EI) calculated for $C_8H_{13}{}^{74}GeO$: 199.0178 $[M-Et]^+$, found: 199.0171. **IR** (neat): *ν*/ cm^{-1} = 3469 (w), 3110 (w), 2954 (s), 2875 (s), 2736 (w), 2190 (w), 1779 (s), 1688 (m), 1608 (w), 1549 (w), 1459 (s), 1426 (m), 1379 (m), 1226 (m) 1147 (m), 1091 (m), 1008 (s), 740 (s), 702 (s), 548 (s). **Elemental analysis** calculated (%) for $C_{10}H_{18}GeO$: C 52.94, H 8.00; found: C 52.58, H 7.99.

5-Bromo-2-(triethylgermyl)pyridine

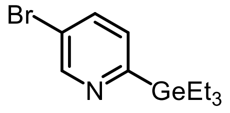

Prepared according to GP 2 using 4-bromo-2-iodopyridine (851.7 mg, 3.00 mmol, 1.0 equiv.). The title product was obtained following purification by column chromatography (hexane/EtOAc 14:1) as a pale yellow oil (76.0 mg, 0.218 mmol, 73%).

R$_f$ = 0.45 (Hexane/EtOAc, 14:1). **^{1}H NMR** (600 MHz, $CDCl_3$) δ/ ppm = 8.80 (d, *J* = 2.4 Hz, 1H), 7.68 (dd, *J* = 8.0, 2.4 Hz, 1H), 7.28 (d, *J* = 8.0 Hz, 1H), 1.05 (dd, *J* = 4.9, 3.3 Hz, 15H). **^{13}C NMR** (151 MHz, $CDCl_3$) δ/ ppm = 167.5, 151.3, 136.3, 130.5, 120.5, 8.9, 4.1. **HRMS** (ESI) calculated for $C_{11}H_{18}{}^{79}Br{}^{74}GeN$: 317.9907 $[M+H]^+$, found: 317.9902 **IR** (neat): *ν*/ cm^{-1} = 3432 (w), 3049 (w), 2950 (s), 2907 (s), 2872 (s), 2828 (m), 2733 (w), 2329 (m), 2161 (w), 2108 (w), 1916 (w), 1832 (w), 1646 (w), 1540 (m), 1446 (s), 1379 (m), 1347 (s), 1229 (m), 1196 (w), 1117 (m), 1081 (s), 1002 (s), 968 (s), 920 (w), 822 (s), 730 (s), 699 (s). **Elemental analysis** calculated (%) for $C_{12}H_{18}BrGeN$: C 41.70, H 5.73, N 4.42; found: C 41.44, H 5.70, N 4.79.

3-Fluoro-2-(triethylgermyl)pyridine

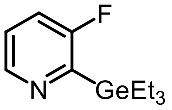

Prepared according to GP 2 using 3-fluoro-2-iodopyridine (669.0 mg, 3.00 mmol, 1.0 equiv.) The title product was obtained after purification by column chromatography (DCM) as a colorless oil (609 mg, 2.38 mmol, 79%).

R$_f$ = 0.76 (DCM). **^{1}H NMR** (600 MHz, $CDCl_3$) δ/ ppm = 8.63 – 8.55 (m, 1H), 7.27 – 7.22 (m, 1H), 7.22 – 7.16 (m, 1H), 1.17 – 1.10 (m, 6H), 1.10 – 1.05 (m, 9H). **^{13}C NMR** (151 MHz, $CDCl_3$) δ/ ppm = 164.6 (d, *J* = 247.6 Hz), 156.4 (d, *J* = 36.8 Hz), 146.6, 123.5 (d, *J* = 4.3 Hz), 120.7 (d, *J* = 19.7 Hz), 9.1, 4.5. **^{19}F NMR** (564 MHz, $CDCl_3$) δ/ ppm = -114.6. **HRMS** (ESI) calculated for $C_{11}H_{18}F{}^{74}GeN$: 258.0708 $[M+H]^+$, found: 258.0705. **IR** (neat): *ν*/ cm^{-1} = 3054 (w), 2951 (s), 2909 (s), 2873 (s), 2734 (w), 2325 (w), 2043 (w), 1584 (w), 1552 (w), 1485 (m), 1413 (s), 1245 (s), 1200 (s), 1114 (m), 1074 (m), 1016 (s), 970 (m), 830 (m), 799 (s), 701 (s).
Elemental analysis calculated (%) for $C_{11}H_{18}FGeN$: C 51.63, H 7.09, N 5.47; found: C 51.69, H 6.90, N 5.63.

2-(Triethylgermyl)pyridine

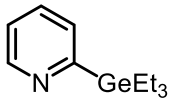

Prepared according to GP 2 using 2-iodopyridine (319 µL, 3.00 mmol, 1.0 equiv.). The title product was obtained after purification by column chromatography (DCM) as a colorless oil (690 g, 2.90 mmol, 97%).

R_f = 0.17 (DCM). **^{1}H NMR** (600 MHz, $CDCl_3$) δ/ ppm = 8.75 (d, *J* = 4.8 Hz, 1H), 7.54 (ddd, *J* = 7.6, 7.6, 1.8 Hz, 1H), 7.39 (d, *J* = 7.6 Hz, 1H), 7.19 – 7.12 (m, 1H), 1.12 – 1.01 (m, 15H). **^{13}C NMR** (151 MHz, $CDCl_3$) δ/ ppm = 169.3, 150.4, 133.9, 129.8, 122.4, 9.1, 4.1. **HRMS** (ESI) calculated for $C_{11}H_{19}{}^{74}GeN$: 240.0802 $[M+H]^+$, found: 240.0802. **IR** (neat): *ν*/ cm^{-1} = 3866 (w), 3060 (m), 2950 (s), 2907 (s), 2872 (s), 2829 (w), 2732 (w), 2689 (w), 2510 (w), 2326 (w), 2206 (w), 2162 (w), 2056 (w), 1993 (w), 1570 (s), 1455 (s), 1419 (s), 1377 (m), 1267 (w), 1228 (w), 1149 (w), 1123 (w), 1081 (w), 1016 (s), 986 (m), 750 (s), 700 (s). **Elemental analysis** calculated (%) for $C_{11}H_{19}GeN$: C 55.53, H 8.05, N 5.89; found: C 55.20, H 7.51, N 6.09.

Triethyl(thiophen-2-yl)germane

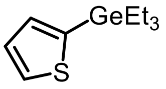

Prepared according to GP 1 using 2-Thienylmagnesium bromide (1.0 M in THF, 3.3 mL, 3.3 mmol). The title product was obtained after purification by column chromatography (hexane/EtOAc, 5:1) as a colorless oil (589 mg, 2.43 mmol, 81%).

R_f = 0.91 (Hexane/ EtOAc, 5:1). **^{1}H NMR** (400 MHz, $CDCl_3$) δ/ ppm = 7.59 (dd, *J* = 4.7, 0.9 Hz, 1H), 7.22 (dd, *J* = 4.7, 3.3 Hz, 1H), 7.18 (dd, *J* = 3.3, 0.9 Hz, 1H), 1.14 – 0.99 (m, 15H). **^{13}C NMR** (101 MHz, $CDCl_3$) δ/ ppm = 137.6, 133.4, 129.7, 127.9, 9.0, 5.7. **HRMS** (EI) calculated for $C_{10}H_{18}{}^{74}GeS$: 244.0336 $[M]^+$, found: 244.0342. **IR** (neat): *ν*/ cm^{-1} = 3070 (w), 2951 (s), 2907 (s), 2872 (s), 2330 (w), 2101 (w), 1593 (w), 1497 (w), 1458 (m), 1425 (m), 1404 (w), 1379 (w), 1323 (w), 1212 (m), 1978 (w), 1016 (s), 965 (s), 846 (m), 823 (m), 746 (w), 698 (s). **Elemental analysis** calculated (%) for $C_{10}H_{18}GeS$: C 49.44, H 7.47; found: C 49.70, H 7.26.

Triethyl(thiophen-3-yl)germane

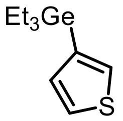

Prepared according to GP 2 using 3-bromothiophen (489 mg, 3.00 mmol, 1.0 equiv.). The title product was obtained after purification by column chromatography (DCM) as a colorless oil (625 mg, 2.57 mmol, 86%).

R_f = 0.95 (DCM). **^{1}H NMR** (400 MHz, $CDCl_3$) δ/ ppm = 7.41 (dd, *J* = 4.8, 2.6 Hz, 1H), 7.33 (dd, *J* = 2.6, 1.1 Hz, 1H), 7.14 (dd, *J* = 4.8, 1.1 Hz, 1H), 1.12 – 1.03 (m, 9H), 1.03 – 0.93 (m, 6H). **^{13}C NMR** (101 MHz, $CDCl_3$) δ/ ppm = 138.4, 131.7, 129.9, 125.2, 9.1, 5.0. **HRMS** (EI) calculated for $C_{10}H_{18}{}^{74}GeS$: 244.0336 $[M]^+$, found: 244.0335. **IR** (neat): *ν*/ cm^{-1} = 3063 (w), 2950 (s), 2907 (s), 2872 (s), 2829 (m), 2733 (w), 2327 (w), 2169 (w), 2099 (w), 1755 (w), 1570 (w), 1458 (s), 1426 (m), 1375 (m), 1337 (w), 1229 (w), 1199 (m), 1089 (s), 1015 (s), 967 (m), 846 (s), 798 (w), 765 (s), 695 (s). **Elemental analysis** calculated (%) for $C_{10}H_{18}GeS$: C 49.44, H 7.47; found: C 49.71, H 7.34.

Triethyl(perfluorophenyl)germane

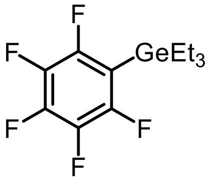

Prepared according to GP 2 using iodopentafluorobenzene (800 µL, 6.00 mmol, 1.0 equiv.). The title product was obtained after purification by column chromatography (pentane) as a colorless oil (1.92 g, 5.87 mmol, 98%).

R_f = 0.90 (Pentane). **^{1}H NMR** (600 MHz, $CDCl_3$) δ/ ppm = 1.15 (q, *J* = 8.5 Hz, 6H), 1.06 (t, *J* = 7.7 Hz, 9H). **^{13}C NMR** (151 MHz, $CDCl_3$) δ/ ppm = 148.9 (dm, *J* = 234.4 Hz), 141.4 (dm, *J* = 252.4 Hz), 137.2 (dm, *J* = 247.2 Hz), 110.1 (tm, *J* = 39.0 Hz), 8.9, 6.0. **^{19}F NMR** (564 MHz, $CDCl_3$) δ/ ppm = -126.30 – -126.42 (m, 2F), -152.93 (t, *J* = 20.2 Hz, 1F), -161.11 – -161.28 (m, 2F). **HRMS** (EI) calculated for $C_{12}H_{15}F_5{}^{74}Ge$: 328.0306 $[M]^+$, found: 328.0303. **IR** (neat): *ν*/ cm^{-1} = 2956 (m), 2159 (w), 1736 (w), 1639 (m), 1512 (m), 1458 (s), 1374 (m), 1276 (w), 1232 (w), 1076 (s), 1015 (m), 963 (s), 801 (w), 704 (s). **Elemental analysis** calculated (%) for $C_{12}H_{15}F_5Ge$: C 44.09, H 4.63; found: C 44.68, H 4.81.

4-(Triethylgermyl)phenyl trifluoromethanesulfonate

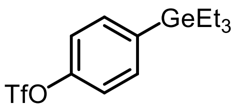

Prepared according to GP 2 using 4-iodophenyl trifluoromethanesulfonate (1056 mg, 3.00 mmol, 1.0 equiv.). The title product was obtained after purification by column chromatography (pentane) as a colorless oil (903 mg, 2.35 mmol, 78%).

R_f = 0.62 (Pentane). **^{1}H NMR** (600 MHz, $CDCl_3$) δ/ ppm = 7.50 (d, *J* = 8.5 Hz, 2H), 7.23 (d, *J* = 8.5 Hz, 2H), 1.08 – 1.03 (m, 9H), 1.03 – 0.97 (m, 6H). **^{13}C NMR** (151 MHz, $CDCl_3$) δ/ ppm = 150.1, 141.3, 135.9, 120.7, 118.9 (q, *J* = 320.6 Hz), 9.0, 4.3. **^{19}F NMR** (564 MHz, $CDCl_3$) δ/ ppm = -72.96. **HRMS** (EI) calculated for $C_{13}H_{19}F_3{}^{74}GeO_3S$: 386.0219 $[M]^+$, found 386.0213. **IR** (neat): *ν*/ cm^{-1} = 2952 (m), 2876 (m), 2329 (w), 2092 (w), 1903 (w), 1766 (w), 1643 (w), 1569 (w), 1489 (m), 1421 (s), 1304 (w), 1210 (s), 1136 (s), 1084 (w), 965 (w), 884 (s), 825 (m), 751 (w), 700 (s). **Elemental analysis** calculated (%) for $C_{13}H_{19}F_3GeO_3S$: C 40.56, H 4.97; found: C 40.55, H 5.26.

Triethyl(4-iodophenyl)germane

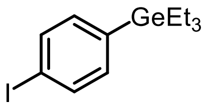

Prepared according to GP 2 using 1,4-diiodobenzene (990 mg, 3.00 mmol, 1.0 equiv.). The title product was obtained after purification by column chromatography (pentane) as a colorless oil (963 mg, 2.65 mmol, 88%).

R_f = 0.90 (Pentane). **^{1}H NMR** (600 MHz, $CDCl_3$) δ/ppm = 7.67 (d, *J* = 8.0 Hz, 2H), 7.16 (d, *J* = 8.0 Hz, 2H), 1.09 – 1.01 (m, 9H), 1.01 – 0.93 (m, 6H). **^{13}C NMR** (151 MHz, $CDCl_3$) δ/ppm = 139.4, 137.0, 135.9, 95.0, 9.0, 4.2. **HRMS** (EI) calculated for $C_{12}H_{19}{}^{74}GeI$: 363.9738 $[M]^+$, found: 363.9744.

IR (neat): *ν*/ cm^{-1} = 3060 (w), 2948 (s), 2872 (s), 2734 (w), 2329 (w), 2097 (w), 1900 (w), 1741 (w), 1631 (w), 1561 (m), 1463 (m), 1426 (m), 1372 (m), 1228 (w), 1051 (w), 1014 (s), 966 (m), 797 (s), 696 (s). **Elemental analysis** calculated (%) for $C_{12}H_{19}GeI$: C 39.73, H 5.28; found: C 41.65, H 6.06.

Trimethyl(4-(triethylgermyl)phenyl)silane

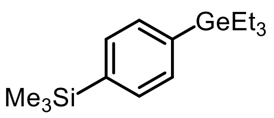

Triethyl(4-iodophenyl)germane (1.09 g, 3.0 mmol, 1.0 equiv.) was added to a round bottom flask and dissolved in anhydrous THF (20 mL). *i*PrMgCl (2.0 M in THF, 1.8 mL, 3.6 mmol, 1.2 equiv.) was added dropwise at 0 °C and the reaction was stirred for 30 min. Tetramethyl orthosilicate (899 µL, 6.0 mmol, 2.0 equiv.) was added and the mixture was stirred at room temperature for 12 h. The reaction was quenched by addition of aqueous solution of NH_4Cl (sat.), the organic phase was separated and the aqueous phase was extracted with DCM (3x20 mL). The combined organic phases were dried with $MgSO_4$ and the solvent was removed under reduced pressure. The title product was obtained after purification by column chromatography (hexane) as a colorless oil (576 mg, 1.86 mmol, 62%).

R_f = 0.89 (Hexane). **^{1}H NMR** (600 MHz, $CDCl_3$) δ/ppm = 7.49 (d, *J* = 8.0 Hz, 2H), 7.42 (d, *J* = 8.0 Hz, 2H), 1.10 – 1.03 (m, 9H), 1.02 – 0.93 (m, 6H), 0.54 (s, 3H). **^{13}C NMR** (151 MHz, $CDCl_3$) δ/ppm = 141.1, 137.9, 133.6, 133.5, 9.1, 4.3, -2.3. **HRMS** (EI) calculated for $C_{15}H_{28}{}^{74}GeSi$: 310.1172 $[M]^+$, found: 310.1180. **IR** (neat): *ν*/ cm^{-1} = 3048 (w), 2951 (s), 2873 (m), 2328 (w), 2113 (w), 1991 (w), 1914 (w), 1584 (w), 1458 (m), 1426 (w), 1377 (m), 1247 (s), 1122 (m), 1014 (m), 967 (m), 839 (s), 800 (m), 754 (m), 697 (s). **Elemental analysis** calculated (%) for $C_{15}H_{28}GeSi$: C 58.29, H 9.13; found: C 58.56, H 9.12.

Triethyl(4-(4,4,5,5-tetramethyl-1,3,2-dioxaborolan-2-yl)phenyl)germane

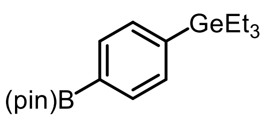

Triethyl(4-iodophenyl)germane (1.09 g, 3.0 mmol, 1.0 equiv.) was added to a round bottom flask and dissolved in anhydrous THF (20 mL). *i*PrMgCl (2.0 M in THF, 1.8 mL, 3.6 mmol, 1.2 equiv.) was added dropwise at 0 °C and the reaction was stirred for 30 min. Tris *iso*-propylborate (1.0 M in THF, 3.3 mL, 3.3 mmol, 1.1 equiv.) was added and the reaction was stirred at room temperature for 12 h. The solvent was removed *in vacuo* and anhydrous toluene (20 mL) and pinacol (1.42 g, 12.0 mmol, 4.0 equiv.) were added. The reaction was stirred for 8 h under reflux. It was quenched by addition of aqueous solution of NH_4Cl (sat.), the organic phase was separated and the aqueous phase was extracted with DCM (3x20 mL). The combined organic phases were dried with $MgSO_4$ and the solvent was removed under reduced pressure. The title product was obtained after purification by column chromatography (hexane/ EtOAc, 50:1) as a white solid (496 mg, 1.34 mmol, 45%).

R_f = 0.47 (Hexane/ EtOAc, 50:1). **M.p.** = 55.6 °C. **^{1}H NMR** (600 MHz, $CDCl_3$) δ/ ppm = 7.77 (d, *J* = 7.7 Hz, 2H), 7.45 (d, *J* = 7.7 Hz, 2H), 1.34 (s, 12H), 1.08 – 1.02 (m, 9H), 1.01 – 0.95 (m, 6H). **^{13}C NMR** (151 MHz, $CDCl_3$) δ/ ppm = 144.1, 136.4, 134.0, 133.5, 83.8, 25.0, 9.1, 4.2.
HRMS (EI) calculated for $C_{18}H_{31}B^{74}GeO_2$: 364.1623 $[M]^+$, found: 364.1636. **IR** (neat): *ν*/ cm^{-1} = 3057 (w), 2953 (m), 2875 (m), 1597 (m), 1503 (w), 1459 (w), 1355 (s), 1298 (m), 1270 (w), 1212 (w), 1143 (s), 1107 (w), 1064 (s), 1017 (m), 962 (m), 857 (s), 818 (m), 736 (w), 696 (s), 659 (m). **Elemental analysis** calculated (%) for $C_{18}H_{31}BGeO_2$: C 59.58, H 8.61; found: C 59.10, H 8.60.

Triethyl(naphthalen-1-yl)germane

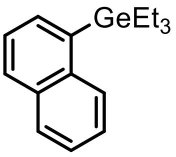

Prepared according to GP 2 using 1-iodonaphthalene (438 µL, 3.00 mmol, 1.0 equiv.). The title product was obtained after purification by column chromatography (hexane) as a colorless oil (743 mg, 2.59 mmol, 86%).

R_f = 0.88 (Hexane). **^{1}H NMR** (400 MHz, $CDCl_3$) δ/ppm = 8.00 – 7.95 (m, 1H), 7.89 – 7.80 (m, 2H), 7.60 (dd, *J* = 6.7, 1.3 Hz, 1H), 7.53 – 7.41 (m, 3H), 1.25 – 1.14 (m, 6H), 1.13 – 1.02 (m, 9H). **^{13}C NMR** (101 MHz, $CDCl_3$) δ/ppm = 138.5, 137.6, 133.7, 133.3, 129.1, 128.9, 128.3, 125.7, 125.4, 125.3, 9.3, 5.7. **HRMS** (EI) calculated for $C_{16}H_{22}{}^{74}Ge$: 288.0928 $[M]^+$, found: 288.0931. **IR** (neat): *ν*/ cm^{-1} = 3850 (w), 3052 (m), 2948 (s), 2872 (s), 2731 (w), 2660 (w), 2325 (m), 2103 (w), 1994 (w), 1930 (w), 1809 (w), 1588 (w), 1504 (m), 1457 (m), 1379 (m), 1322 (w), 1221 (w), 1138 (m), 1013 (s), 967 (s), 855 (w), 785 (s), 701 (s). **Elemental analysis** calculated (%) for $C_{16}H_{22}Ge$: C 66.96, H 7.73; found: C 67.21, H 7.95.

Triethyl(phenanthren-9-yl)germane

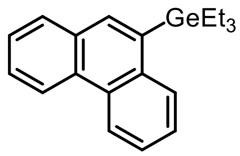

Prepared according to GP 2 using 9-bromophenanthene (771.4 mg, 3.0 mmol, 1.0 equiv.). The title product was obtained after purification by column chromatography (hexane/EtOAc) as a colorless oil (550 mg, 1.63 mmol, 54%).

R_f = 0.50 (Hexane/EtOAc 95:5). **^{1}H NMR** (400 MHz, $CDCl_3$) δ/ppm = 8.75 (dd, *J* = 8.0, 1.6 Hz, 1H), 8.69 (d, *J* = 8.0 Hz, 1H), 8.03 (dd, *J* = 8.0, 1.5 Hz, 1H), 7.88 (dd, *J* = 7.6, 1.7 Hz, 1H), 7.85 (s, 1H), 7.70 – 7.56 (m, 4H), 1.31 – 1.20 (m, 6H), 1.15 – 1.06 (m, 9H). **^{13}C NMR** (151 MHz, $CDCl_3$) δ/ppm = 136.8, 135.6, 135.0, 131.3, 130.6, 130.1, 128.8, 128.5, 126.7, 126.5, 126.3, 126.0, 123.2, 122.5, 9.1, 5.5. **HRMS** (EI) calculated for $C_{20}H_{24}{}^{74}Ge$: 338.1084 $[M]^+$, found: 338.1087. **IR** (neat): *ν*/ cm^{-1} = 3470 (w), 3069 (m), 2951 (s), 2871 (s), 2733 (w), 2184 (w), 1945 (w), 1916 (w), 1697 (w), 1582 (w), 1525 (w), 1488 (m), 1453 (s), 1378 (m), 1244 (m), 1144 (w), 1100 (w), 1014 (s), 968 (m), 894 (m), 851 (w), 745 (s), 715 (s), 615 (w), 574 (s), 506 (w), 464 (w). **Elemental analysis** calculated (%) for $C_{20}H_{24}Ge$: C 71.27, H 7.18; found: C 71.30, H 7.15.

2.3 Synthesis of Diaryliodonium Salts

2.3.1 General Procedure 3 (GP 3)

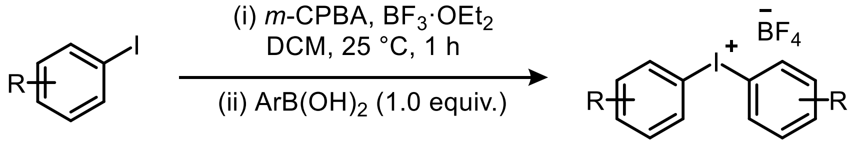

Under a flow of argon *m*-CPBA (≥77 % active oxidant, 1.35 g, 6.0 mmol, 1.1 equiv.) was dissolved in anhydrous DCM (20 mL). Whilst stirring, aryl iodide (5.4 mmol, 1.0 equiv.) was added by syringe and $BF_3{\cdot}OEt_2$ added dropwise (1.7 mL, 13.5 mL). After 45 minutes, the reaction was cooled to 0 °C before the aryl boronic acid (1.1 equiv.) was added in portions over 10 minutes. The reaction was allowed to warm to room temperature, then stir for a further 45 minutes. At the end of the reaction, the solution was filtered through 12 g silica with 120 mL DCM then further with 250 mL DCM/MeOH (95:5). The second elution was concentrated under reduced pressure and the resulting diaryliodonium salt precipitated with Et_2O. The solids were washed 3 times with Et_2O before drying under high vacuum to yield the pure diaryliodonium salts.

2.3.2 Compound Characterization

Diphenyliodonium tetrafluoroborate

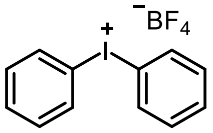

Prepared according to GP 3 using iodobenzene (602 µL, 5.4 mmol, 1.0 equiv.) and phenylboronic acid (732 mg, 6.0 mmol, 1.1 equiv.). Diphenyl iodonium tetrafluoroborate was purified by crystallization and isolated as a white solid (1.65 g, 4.50 mmol, 83%).

^{1}H NMR (300 MHz, DMSO-d_6) δ/ ppm = 8.29 – 8.18 (d, J = 7.9 Hz, 4H), 7.71 – 7.59 (dd, J = 7.4, 7.4 Hz, 2H), 7.57 – 7.46 (dd, J = 7.4, 7.4 Hz, 4H). **^{19}F NMR** (282 MHz, DMSO-d_6) δ/ ppm = -148.22, -148.27. **HRMS** (ESI) calculated for $C_{12}H_{10}IBF_4$: 280.9822 $[M\text{-}BF_4]^+$, found: 280.9810.
These data are in agreement with those reported previously in the literature.[1]

Bis(4-fluorophenyl)iodonium tetrafluoroborate

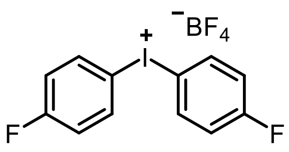

Prepared according to GP 3 using 4-fluoroiodobenzene (623 µL, 5.4 mmol, 1.0 equiv.) and 4-fluorophenylboronic acid (840 mg, 6.0 mmol, 1.1 equiv.). Diphenyl iodonium tetrafluoroborate was purified by crystallization and isolated as a white solid (1.75 g, 4.32 mmol, 80%).

^{1}H NMR (300 MHz, DMSO-d_6) δ/ ppm = 8.37 – 8.20 (m, 4H), 7.49 – 7.30 (m, 4H). **^{19}F NMR** (282 MHz, DMSO-d_6) δ/ ppm = -106.55 – -106.72 (m), -148.21, -148.26 [BF_4]. **HRMS** (ESI) calculated for $C_{12}H_8IBF_6$: 316.9633 $[M\text{-}BF_4]^+$, found: 316.9635. These data are in agreement with those reported previously in the literature.[2]

Bis(4-trifluoromethyl)phenyl iodonium tetrafluoroborate

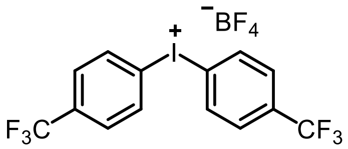

Prepared according to GP 3 using 4-iodobenzotrifluoride (790 µL, 5.4 mmol, 1.0 equiv.) and 4-trifluoromethyl phenylboronic acid (1.14 g, 6.0 mmol, 1.1 equiv.). Diphenyl iodonium tetrafluoroborate was purified by crystallization and isolated as a white solid (1.81 g, 3.60 mmol, 67%).

^{1}H NMR (300 MHz, DMSO-d_6) δ/ ppm = 8.53 – 8.43 (m, 4H), 7.97 – 7.87 (m, 4H). **^{19}F NMR** (282 MHz, DMSO-d_6) δ/ ppm = -61.72, -148.24, -148.29 [BF_4]. **HRMS** (ESI) calculated for $C_{14}H_8IBF_{10}$: 416.957 $[M\text{-}BF_4]^+$, found: 416.9569.
These data are in agreement with those reported previously in the literature.[1]

2.4 Cross Coupling Reactions

2.4.1 General Procedures

General Procedure 4 (GP 4)

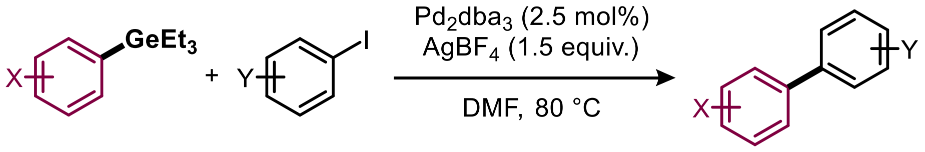

Aryl germane (1.0 equiv.), aryl iodide (1.5 equiv.), $AgBF_4$ (1.5 equiv.) and Pd_2dba_3 (2.5 mol%) were added to a reaction vial, dissolved in DMF (0.3 M) and stirred at 80 °C for 16 h. The reaction was quenched by addition of aqueous solution of NH_4Cl (sat.), the organic phase was separated and the aqueous phase was extracted with DCM (3x). The combined organic phases were dried with $MgSO_4$, the solvent was removed under reduced pressure and the crude product mixture was purified by silica column chromatography.

General Procedure 5 (GP 5)

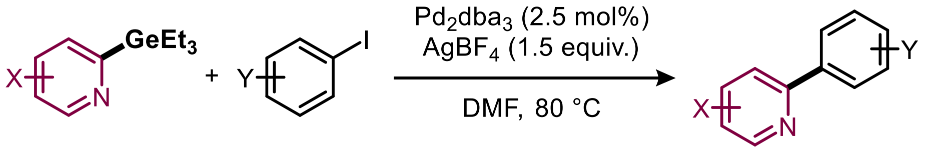

Aryl germane (1.2 equiv.), aryl iodide (1.0 equiv.), $AgBF_4$ (1.5 equiv.) and Pd_2dba_3 (2.5 mol%) were added to a reaction vial, dissolved in DMF (0.3 M) and stirred at 80 °C for the stated reaction time. The reaction was quenched by addition of aqueous solution of NH_4Cl (sat.), the organic phase was separated and the aqueous phase was extracted with DCM (3x). The combined organic phases were dried with $MgSO_4$, the solvent was removed under reduced pressure and the crude product mixture was purified by silica column chromatography.

General Procedure 6 (GP 6)

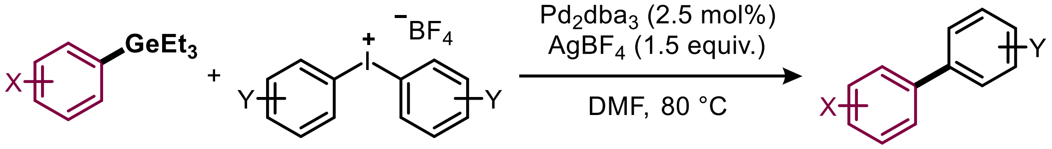

Aryl germane (1.0 equiv.), diaryliodonium tetrafluoroborate salt (1.5 equiv.), $AgBF_4$ (1.5 equiv.) and Pd_2dba_3 (2.5 mol%) were added to a reaction vial and dissolved in DMF (0.3 M) and stirred at 80 °C for 16 hours. The reaction was quenched by addition of aqueous solution of NH_4Cl (sat.), the organic phase was separated and the aqueous phase was extracted with DCM (3x). The combined organic phases were dried with $MgSO_4$, the solvent was removed under reduced pressure and the crude product mixture was purified by silica column chromatography.

General Procedure 7 (GP 7)

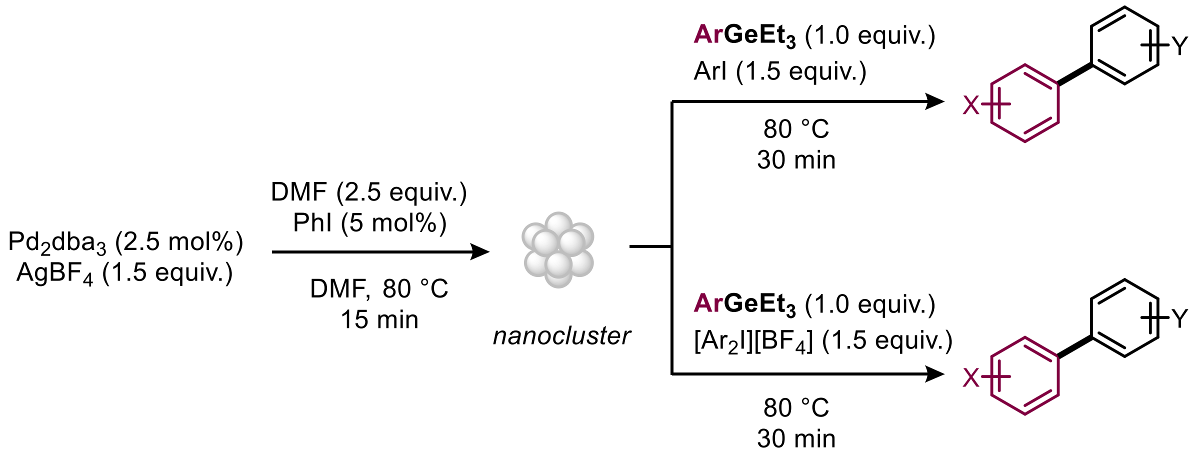

$AgBF_4$ (1.5 equiv.) and Pd_2dba_3 (2.5 mol%) were stirred with DMF (2.5 equiv.) and iodobenzene (5 mol%) at 80 °C for 20 minutes in an open flask. A solution of aryl germane (1.0 equiv., 0.3 M in 1,4-dioxane) and either aryl iodide (1.5 equiv.) or diaryliodonium salt (1.5 equiv.) were added and the reaction stirred for a further 60 minutes at 80 °C under air. The reaction was quenched by addition of aqueous solution of NH_4Cl (sat.), the organic phase was separated and the aqueous phase was extracted with DCM (3x). The combined organic phases were dried with $MgSO_4$, the solvent was removed under reduced pressure and the crude product mixture was purified by silica column chromatography.

2.4.2 Compound Characterization

Methyl 4'-fluoro-3-methoxy-[1,1'-biphenyl]-4-carboxylate

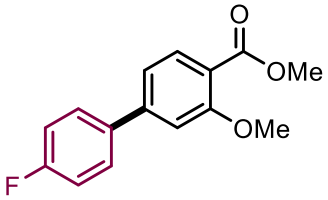

According to GP 4 the cross coupling was performed using triethyl(4-fluorophenyl)germane (76.5 mg, 0.3 mmol, 1.0 equiv.) and methyl 4-iodo-2-methoxybenzoate (131 mg, 0.45 mmol, 1.5 equiv.). The title product was obtained after purification by column chromatography (pentane) as a colorless oil (69.4 mg, 0.266 mmol, 89%).

R$_f$ = 0.64 (Pentane/EtOAc 95:5). **^{1}H NMR** (600 MHz, $CDCl_3$) δ/ ppm = 7.98 – 7.79 (m, 1H), 7.60 – 7.51 (m, 2H), 7.17 – 7.13 (m, 3H), 7.12 – 7.09 (m, 1H), 3.97 (s, 3H), 3.91 (s, 3H). **^{13}C NMR** (101 MHz, $CDCl_3$) δ/ ppm = 166.4, 163.8, 162.1, 159.6, 145.7, 136.3 (d, *J* = 3.5 Hz), 132.4, 128.9 (d, *J* = 8.5 Hz), 118.9, 115.8 (d, *J* = 21.5 Hz), 110.7, 56.1, 52.1. **^{19}F NMR** (564 MHz, $CDCl_3$) δ/ ppm = -114.0 (m, 1F). **HRMS** (ESI) calculated for $C_{15}H_{13}O_3FNa$: 283.0746 $[M+Na]^+$, found: 283.0745.

These data are in agreement with those reported previously in the literature.[3]

1-(4-Methoxyphenyl)naphthalene

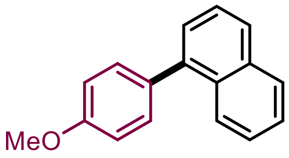

According to GP 4 the cross coupling was performed using triethyl(naphthalen-1-yl)germane (86.1 mg, 0.3 mmol, 1.0 equiv.) and 4-iodoanisole (105 mg, 0.45 mmol, 1.5 equiv.). The title product was obtained after purification by column chromatography (hexane/EtOAc 15:1) as a white solid. (67.0 mg, 0.286 mmol, 95%).

R$_f$ = 0.32 (Hexane/EtOAc 10:1). **^{1}H NMR** (600 MHz, $CDCl_3$) δ/ ppm = 7.95 (d, *J* = 8.4 Hz, 1H), 7.92 (d, *J* = 8.2 Hz, 1H), 7.86 (d, *J* = 8.2 Hz, 1H), 7.55 – 7.47 (m, 2H), 7.47 – 7.39 (m, 4H), 7.06 (d, *J* = 8.4 Hz, 2H), 3.91 (s, 3H). **^{13}C NMR** (151 MHz, $CDCl_3$) δ/ ppm = 159.1, 140.0, 134.0, 133.3, 132.0, 131.3, 128.4, 127.5, 127.0, 126.2, 126.1, 125.8, 125.5, 113.8, 55.5. **HRMS** (EI) calculated for $C_{17}H_{14}O$: 234.1046 $[M]^+$, found: 234.1039.

These data are in agreement with those reported previously in the literature.[4]

3-Fluoro-4'-methoxy-1,1'-biphenyl

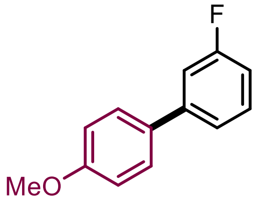

According to GP 4 the cross coupling was performed using triethyl(4-methoxyphenyl)germane (80.1 mg, 0.3 mmol, 1.0 equiv.) and 3-fluoroiodobenzene (100 mg, 0.45 mmol, 1.5 equiv.). The title product was obtained after purification by column chromatography (hexane) as a white solid (58.6 mg, 0.290 mmol, 97%).

R_f = 0.24 (Hexane). **^{1}H NMR** (600 MHz, $CDCl_3$) δ/ ppm = 7.52 (d, *J* = 8.8 Hz, 2H), 7.44 – 7.30 (m, 2H), 7.30 – 7.22 (m, 1H), 6.98 (m, 3H), 3.85 (s, 3H). **^{13}C NMR** (151 MHz, $CDCl_3$) δ/ ppm = 163.4 (d, *J* = 244.9 Hz), 159.7, 143.2 (d, *J* = 7.8 Hz), 132.5 (d, *J* = 2.3 Hz), 130.3 (d, *J* = 8.5 Hz), 128.3, 127.9, 122.4 (d, *J* = 2.9 Hz), 113.6 (d, *J* = 21.5), 113.5 (d, *J* = 21.3), 55.47. **^{19}F NMR** (564 MHz, $CDCl_3$) δ/ ppm = -111.62 – -114.74 (m). **HRMS** (EI) calculated for $C_{13}H_{10}FO$: 202.0788 $[M]^+$, found: 202.0786. These data are in agreement with those reported previously in the literature.[5]

4-Methoxy-4'-methylbiphenyl

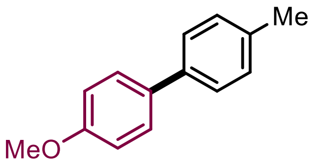

According to GP 4 the cross coupling was performed using triethyl(4-methoxyphenyl)germane (80.1 mg, 0.3 mmol, 1.0 equiv.) and 4-iodotoluene (98.1 mg, 0.45 mmol, 1.5 equiv.). The title product was obtained after purification by column chromatography (pentane/Et_2O 100:1) as a light yellow solid (30.7 mg, 0.155 mmol, 52%)

R_f = 0.24 (Pentane/Et_2O 100:1). **^{1}H NMR** (400 MHz, $CDCl_3$) δ/ ppm = 7.55 – 7.49 (m, 2H), 7.49 – 7.42 (m, 2H), 7.25 – 7.20 (m, 2H), 7.01 – 6.94 (m, 2H), 3.85 (s, 3H), 2.39 (s, 3H). **^{13}C NMR** (101 MHz, $CDCl_3$) δ/ ppm = 159.1, 138.1, 136.5, 133.9, 129.6, 128.1, 126.7, 114.3, 55.5, 21.2. **MS** (EI): *m/z* (%): 198 (100) $[M]^+$, 183 (56), 155 (40), 128 (14), 89 (3), 77 (5).

These data are in agreement with those reported previously in the literature.[6]

4-Methoxy-3'-methylbiphenyl

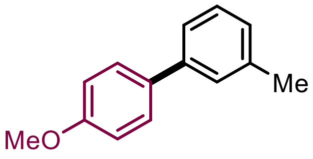

According to GP 4 the cross coupling was performed using triethyl(4-methoxyphenyl)germane (80.1 mg, 0.3 mmol, 1.0 equiv.) and 3-iodotoluene (98.1 mg, 0.45 mmol, 1.5 equiv.). The title product was obtained after purification by column chromatography (pentane/Et_2O 100:1) as a light yellow solid (32.6 mg, 0.20 mmol, 55%).

R_f = 0.14 (Pentane/Et_2O 100:1). **^{1}H NMR** (400 MHz, $CDCl_3$) δ/ ppm = 7.57 – 7.48 (m, 2H), 7.39 – 7.35 (m, 2H), 7.32 (ddd, *J* = 7.4, 7.4, 0.8 Hz, 1H), 7.14 (m, 1H), 7.02 – 6.95 (m, 2H), 3.86 (s, 3H), 2.43 (s,

3H). **^{13}C NMR** (101 MHz, $CDCl_3$) δ/ ppm = 159.2, 141.0, 138.4, 134.1, 128.8, 128.3, 127.7, 127.6, 124.0, 114.3, 55.5, 21.7. **MS** (EI): *m/z* (%): 198 (100) $[M]^+$, 183 (52), 155 (41), 128 (16), 89 (4), 63 (6). These data are in agreement with those reported previously in the literature.[6]

4-Methoxy-2'-methylbiphenyl

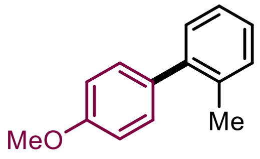

According to GP 4 the cross coupling was performed using triethyl(4-methoxyphenyl)germane (80.1 mg, 0.3 mmol, 1.0 equiv.) and 2-iodotoluene (98.1 mg, 0.45 mmol, 1.5 equiv.). The title product was obtained after purification by column chromatography (pentane/Et_2O 200:1 to 100:1) as a light yellow oil (38.7 mg, 0.20 mmol, 65%).

R_f = 0.14 (Pentane/Et_2O 100:1). **^{1}H NMR** (400 MHz, $CDCl_3$) δ/ ppm = 7.29 – 7.18 (m, 6H), 6.97 – 6.92 (m, 2H), 3.85 (s, 3H), 2.27 (s, 3H). **^{13}C NMR** (101 MHz, $CDCl_3$) δ/ ppm = 158.7, 141.7, 135.6, 134.5, 130.4, 130.4, 130.0, 127.1, 125.9, 113.6, 55.4, 20.7. **MS** (70 eV, EI): *m/z* (%): 198 (100, M^+), 183 (26), 163 (2), 153 (32), 128 (22), 115 (17), 77 (6).
These data are in agreement with those reported previously in the literature.[7]

4-Methyl-4'-(trifluoromethyl)-1,1'-biphenyl

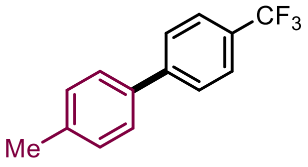

According to GP 4 the cross coupling was performed using triethyl(*p*-tolyl)germane (75.3 mg, 0.3 mmol, 1.0 equiv.) and 1-iodo-4-(trifluoromethyl)benzene (122.4 mg, 0.45 mmol, 1.5 equiv.). The title product was obtained after purification by column chromatography (hexane), and further purification by prep-HPLC (hexane) as a white solid (60.5 mg, 0.256 mmol, 85%).

R_f = 0.67 (Hexane). **^{1}H NMR** (600 MHz, $CDCl_3$) δ/ ppm = 7.68 (s, 4H), 7.51 (d, *J* = 8.0 Hz, 2H), 7.29 (d, *J* = 8.0 Hz, 2H), 2.42 (s, 3H). **^{13}C NMR** (151 MHz, $CDCl_3$) δ/ ppm = 144.8, 138.3, 137.0, 129.9, 129.2 (q, *J* = 32.3 Hz), 127.3, 127.2, 125.8 (q, *J* = 3.8 Hz), 124.5 (q, *J* = 272.1 Hz), 21.3. **^{19}F NMR** (564 MHz, $CDCl_3$) δ/ ppm = -62.34. **HRMS** (EI) calculated for $C_{14}H_{11}F_3$: 236.0807 $[M]^+$, found: 236.0810.
These data are in agreement with those reported previously in the literature.[8]

4-Fluoro-4'-(trifluoromethyl)-1,1'-biphenyl

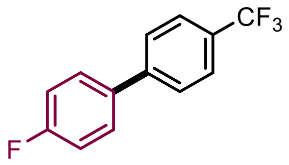

According to GP 4 the cross coupling was performed using triethyl(4-fluorophenyl)germane (76.5 mg, 0.30 mmol, 1.0 equiv.) and 4-iodobenzotrifluoride (122.4 mg, 0.45 mmol, 1.5 equiv.). The title product was obtained after purification by column chromatography (pentane) as a colorless oil (64.1 mg, 0.267 mmol, 89%).

R$_f$ = 0.43 (pentane). **^{1}H NMR** (400 MHz, $CDCl_3$) δ/ ppm = 7.71 – 7.67 (m, 2H), 7.67 – 7.62 (m, 2H), 7.59 – 7.49 (m, 2H), 7.20 – 7.14 (m, 2H). **^{13}C NMR** (101 MHz, $CDCl_3$) δ/ ppm = 163.1 (d, *J* = 248.0 Hz), 143.9, 136.0 (d, *J* = 3.3 Hz), 129.5 (q, *J* = 32.5 Hz), 129.1 (d, *J* = 8.2 Hz), 127.4, 125.9 (q, *J* = 3.9 Hz), 124.4 (q, *J* = 271.8 Hz), 116.1 (d, *J* = 21.4 Hz). **^{19}F NMR** (376 MHz, $CDCl_3$) δ/ ppm = -62.5 (s, 3F), -114.2 (m, 1F). **HRMS** (EI) calculated for $C_{13}H_8F_4$: 240.0557 $[M]^+$, found: 240.0564.
These data are in agreement with those reported previously in the literature.[9]

2-Fluoro-4'-(trifluoromethyl)-1,1'-biphenyl

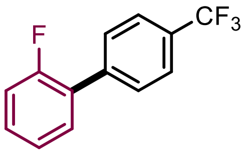

According to GP 4 the cross coupling was performed using triethyl(2-fluorophenyl)germane (76.5 mg, 0.30 mmol, 1.0 equiv.) and 4-iodobenzotrifluoride (122.4 mg, 0.45 mmol, 1.5 equiv.). The title product was obtained after purification by column chromatography (pentane) as a colorless oil (15.0 mg, 0.063 mmol, 21%).

R$_f$ = 0.48 (pentane). **^{1}H NMR** (400 MHz, $CDCl_3$) δ/ ppm = 7.72 – 7.64 (m, 4H), 7.48 – 7.42 (m, 1H), 7.41 – 7.34 (m, 1H), 7.27 – 7.22 (m, 1H), 7.21 – 7.16 (m, 1H). **^{13}C NMR** (101 MHz, $CDCl_3$) δ/ ppm = 159.8 (d, *J* = 248.3 Hz), 139.5, 130.8 (d, *J* = 2.7 Hz), 130.1 (d, *J* = 8.2 Hz), 129.9 (q, *J* = 32.5 Hz), 128.5 (d, *J* = 3.0 Hz), 127.8 (d, *J* = 13.1 Hz), 125.5 (d, *J* = 3.8 Hz), 124.7 (d, *J* = 3.7 Hz), 124.3 (q, *J* = 272.3 Hz), 116.5 (d, *J* = 22.8 Hz). **^{19}F NMR** (376 MHz, $CDCl_3$) δ/ ppm = -62.6 (s, 3F), -117.9 (m, 1F). **HRMS** (EI) calculated for $C_{13}H_8F_4$: 240.0557 $[M]^+$, found: 240.0565.
These data are in agreement with those reported previously in the literature.[9]

3-Fluoro-4'-(trifluoromethyl)-1,1'-biphenyl

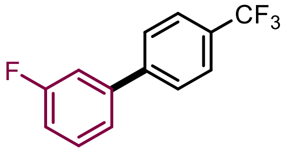

According to GP 4 the cross coupling was performed using triethyl(3-fluorophenyl)germane (76.5 mg, 0.30 mmol, 1.0 equiv.), 4-iodobenzotrifluoride (122.4 mg, 0.45 mmol, 1.5 equiv.). The title product was obtained after purification by column chromatography (pentane) as a colorless oil (48.9 mg, 0.204 mmol, 68%).

R_f = 0.49 (pentane). **^{1}H NMR** (600 MHz, $CDCl_3$) δ/ ppm = 7.73 – 7.65 (m, 4H), 7.48 – 7.41 (m, 1H), 7.40 – 7.36 (m, 1H), 7.31 – 7.28 (m, 1H), 7.13 – 7.07 (m, 1H). **^{13}C NMR** (151 MHz, $CDCl_3$) δ/ ppm = 163.4 (d, *J* = 246.8 Hz), 143.5, 142.1 (d, *J* = 7.6 Hz), 130.7 (d, *J* = 8.5 Hz), 130.1 (q, *J* = 32.8 Hz), 127.6, 126.0 (q, *J* = 3.8 Hz), 124.3 (q, *J* = 272.1 Hz), 123.1 (d, *J* = 2.9 Hz), 115.2 (d, *J* = 21.2 Hz), 114.4 (d, *J* = 22.3 Hz). **^{19}F NMR** (564 MHz, $CDCl_3$) δ/ ppm = -62.5 (s, 3F), -112.5 (m, 1F). **HRMS** (EI) calculated for $C_{13}H_8F_4$: 240.0557 $[M]^+$, found: 240.0557.
These data are in agreement with those reported previously in the literature.[9]

2-(4-Fluorophenyl)furan

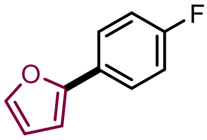

According to GP 4 the cross coupling was performed using 2-triethylgermaniumfuran (81.7 mg, 0.36 mmol, 1.2 equiv.) and 4-fluoroiodobenzene (66.6 mg, 0.45 mmol, 1.0 equiv.). The title product was obtained after purification by column chromatography (hexane) as a light yellow oil (26 mg, 0.16 mmol, 53%).

R_f = 0.26 (Hexane). **^{1}H NMR** (600 MHz, $CDCl_3$) δ/ ppm = 7.67 – 7.60 (m, 2H), 7.45 (d, *J* = 2.0 Hz, 1H), 7.11 – 7.04 (m, 2H), 6.58 (d, *J* = 3.4 Hz, 1H), 6.46 (dd, *J* = 3.4, 1.8 Hz, 1H). **^{13}C NMR** (151 MHz, $CDCl_3$) δ/ ppm = 162.1 (d, *J* = 246.7 Hz), 153.1, 142.0, 127.3 (d, *J* = 3.0 Hz), 125.5 (d, *J* = 7.9 Hz), 115.7 (d, *J* = 22.0 Hz), 111.7, 104.6. **^{19}F NMR** (564 MHz, $CDCl_3$) δ/ ppm = -114.46 (ddd, *J* = 13.5, 8.5, 5.2 Hz). **MS** (EI): *m/z* (%): 162.1 (67) $[M]^+$, 134.1 (20), 133.1 (100), 107.0 (10).
These data are in agreement with those reported previously in the literature.[10]

2-(Furan-2-yl)-6-(trifluoromethyl)pyridine

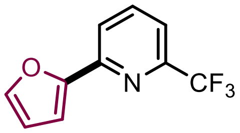

According to GP 5 the cross coupling was performed using 2-triethylgermaniumfuran (81.7 mg, 0.36 mmol, 1.2 equiv.) and 2-iodo-6-trifluoromethyl pyridine (81.9 mg, 0.3 mmol, 1.0 equiv.). The title product was obtained after purification by column chromatography (hexane/ EtOAc 7:1) as a yellow oil (32 mg, 0.15 mmol, 50%).

R$_f$ = 0.40 (Hexane/EtOAc 7:1). **^{1}H NMR** (400 MHz, $CDCl_3$) δ/ ppm = 7.93 – 7.79 (m, 2H), 7.56 (dd, *J* = 1.8, 0.9 Hz, 1H), 7.51 (dd, *J* = 7.0, 1.8 Hz, 1H), 7.20 (dd, *J* = 3.4, 0.9 Hz, 1H), 6.56 (dd, *J* = 3.4, 1.8 Hz, 1H). **^{13}C NMR** (151 MHz, $CDCl_3$) δ/ ppm = 152.6, 149.9, 148.3 (q, *J* = 34.8 Hz), 144.2, 138.1, 121.6 (d, *J* = 274.5 Hz), 121.0, 118.2 (d, *J* = 3.0 Hz), 112.5, 110.6.
^{19}F NMR (376 MHz, $CDCl_3$) δ/ ppm = -68.28. **HRMS** (ESI) calculated for $C_{10}H_6ONF_3Na$: 236.0294 $[M+Na]^+$: found: 236.0293.

5-Bromo-2-(4-(trifluoromethyl)phenyl)pyridine

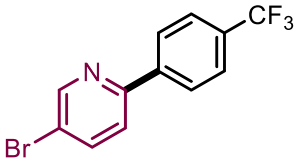

According to GP 5 (72 h) the cross coupling was performed using 4-bromo-2-(triethylgermyl)pyridine (114.0 mg, 0.36 mmol, 1.2 equiv.) and 4-iodobenzotrifluoride (44.1 µL, 0.3 mmol, 1.0 equiv.). The title product was obtained after purification by column chromatography (DCM) as a light yellow oil (57 mg, 0.190 mmol, 63%).

R$_f$ = 0.32 (DCM). **^{1}H NMR** (600 MHz, $CDCl_3$) δ/ ppm = 8.77 (d, *J* = 2.4 Hz, 1H), 8.09 (d, *J* = 8.2 Hz, 2H), 7.92 (dd, *J* = 8.4, 2.4 Hz, 1H), 7.73 (d, *J* = 8.2 Hz, 2H), 7.66 (d, *J* = 8.4 Hz, 1H). **^{13}C NMR** (151 MHz, $CDCl_3$) δ/ ppm = 154.3, 151.0, 141.5, 139.6, 131.1 (d, *J* = 33.0 Hz), 127.0, 125.8 (q, *J* = 3.8 Hz), 121.9, 120.4. **^{19}F NMR** (564 MHz, $CDCl_3$) δ/ ppm = -62.67. **HRMS** (ESI) calculated for $C_{13}H_8{}^{79}BrF_3N$: 301.9787 $[M+H]^+$, found: 301.9783.
These data are in agreement with those reported previously in the literature.[11]

3-Fluoro-2-(4-(trifluoromethyl)phenyl)pyridine

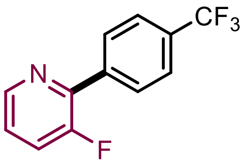

According to GP 5 (72 h) the cross coupling was performed using 3-fluoro-2-(triethylgermyl)pyridine (92.1 mg, 0.36 mmol, 1.2 equiv.) and 4-iodobenzotrifluoride (44.1 µL, 0.3 mmol, 1.0 equiv.). The title product was obtained by column chromatography (DCM) as a pale yellow solid (37.8 mg, 0.156 mmol, 52%).

R$_f$ = 0.40 (DCM). **^{1}H NMR** (400 MHz, $CDCl_3$) δ/ ppm = 8.54 (ddd, *J* = 4.6, 4.6, 1.6 Hz, 1H), 8.13 – 8.06 (m, 2H), 7.76 – 7.69 (m, 2H), 7.51 (ddd, *J* = 11.0, 8.3, 1.4 Hz, 1H), 7.32 (ddd, *J* = 8.3, 4.6, 3.7 Hz, 1H). **^{13}C NMR** (151 MHz, $CDCl_3$) δ/ ppm = 157.8 (d, *J* = 261.5 Hz), 145.6 (d, *J* = 5.0 Hz), 144.6 (d, *J* = 10.4 Hz), 138.7 (d, *J* = 5.4 Hz), 131.0 (q, *J* = 32.4 Hz), 129.1 (d, *J* = 6.1 Hz), 125.4 (q, *J* = 3.7 Hz), 124.5, 124.4 – 124.3 (m), 124.1 (q, *J* = 272.3 Hz). **HRMS** (EI) calculated for $C_{12}H_7F_4N$: 241.0515 $[M]^+$, found: 241.0510.
These data are in agreement with those reported previously in the literature.[12]

2-(4-(Trifluoromethyl)phenyl)pyridine

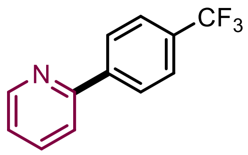

According to GP 5 (72 h) the cross coupling was performed using 2-(triethylgermyl)pyridine (86.1 mg, 0.36 mmol, 1.2 equiv.) and 4-iodobenzotrifluoride (44.1 µL, 0.3 mmol, 1.0 equiv.). The title product was obtained by column chromatography (DCM) as a white solid (48.2 mg, 0.216 mmol, 72%).

R$_f$ = 0.30 (DCM). **^{1}H NMR** (600 MHz, $CDCl_3$) δ/ ppm = 8.80 – 8.59 (m, 1H), 8.11 (d, *J* = 8.1 Hz, 2H), 7.82 – 7.75 (m, 2H), 7.73 (d, *J* = 8.1 Hz, 2H), 7.30 (ddd, *J* = 6.7, 4.8, 1.4 Hz, 1H). **^{13}C NMR** (151 MHz, $CDCl_3$) δ/ ppm = 155.9, 149.9, 142.7, 137.0, 130.8 (q, *J* = 32.2 Hz), 127.2, 125.7 (q, *J* = 3.9 Hz), 123.0, 120.9. *CF_3 quartet not fully visible.* **^{19}F NMR** (564 MHz, $CDCl_3$) δ/ ppm = -62.58. **HRMS** (EI) calculated for $C_{12}H_9F_3N$: 224.0682 $[M+H]^+$, found: 224.0682.
These data are in agreement with those reported previously in the literature.[13]

2-(4-Fluorophenyl)thiophene

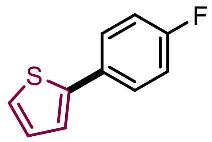

According to GP 4 the cross coupling was performed using triethyl(thiophen-2-yl)germane (72.9 mg, 0.30 mmol, 1.0 equiv.) and 1-fluoro-4-iodobenzene (100 mg, 0.45 mmol, 1.5 equiv.). The title product was obtained after purification by column chromatography (hexane) as a white solid (47.0 mg, 0.264 mmol, 88%).

R$_f$ = 0.60 (Hexane). **^{1}H NMR** (600 MHz, $CDCl_3$) δ/ ppm = 7.61 – 7.55 (m, 2H), 7.28 (d, *J* = 5.1 Hz, 1H), 7.25 (d, *J* = 3.5 Hz, 1H), 7.13 – 7.05 (m, 3H). **^{13}C NMR** (151 MHz, $CDCl_3$) δ/ ppm = 162.4 (d, *J* = 246.9 Hz), 143.4, 130.8 (d, *J* = 3.0 Hz), 128.2, 127.7 (d, *J* = 7.7 Hz), 124.9, 123.2, 116.0 (d, *J* = 21.4 Hz). **^{19}F NMR** (564 MHz, $CDCl_3$) δ/ ppm = -113.79 – -115.70 (m). **HRMS** (EI) calculated for $C_{10}H_7FS$: 178.0247 $[M]^+$, found: 178.0246.
These data are in agreement with those reported previously in the literature.[14]

1-(4-(Thiophen-3-yl)phenyl)ethan-1-one

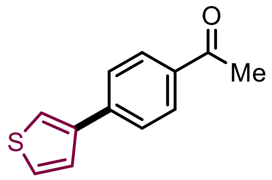

According to GP 4 the cross coupling was performed using triethyl(thiophen-3-yl)germane (72.9 mg, 0.30 mmol, 1.0 equiv.) and 1-(4-iodophenyl)ethan-1-one (110 mg, 0.45 mmol, 1.5 equiv.). The title product was obtained after purification by column chromatography (hexane/EtOAc, 10:1) as a white solid (38.4 mg, 0.190 mmol, 63%).

R$_f$ = 0.23 (Hexane/EtOAc, 10:1). **^{1}H NMR** (600 MHz, $CDCl_3$) δ/ ppm = 8.00 (d, *J* = 8.3 Hz, 2H), 7.69 (d, *J* = 8.3 Hz, 2H), 7.60 – 7.57 (m, 1H), 7.46 – 7.42 (m, 2H), 2.63 (s, 3H). **^{13}C NMR** (151 MHz, $CDCl_3$) δ/ ppm = 197.7, 141.2, 140.3, 135.7, 129.2, 126.9, 126.5, 126.3, 122.2, 26.8. **HRMS** (ESI) calculated for $C_{12}H_{11}OS$: 202.0452 $[M+H]^+$, found: 203.0525.

These data are in agreement with those reported previously in the literature.[15]

4-(Thiophen-2-yl)-2,3-dihydro-1*H*-inden-1-one

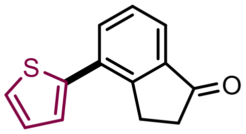

According to GP 4 the cross coupling was performed using triethyl(thiophen-2-yl)germane (72.9 mg, 0.30 mmol, 1.0 equiv.) and 4-iodo-2,3-dihydro-1*H*-inden-1-one (116 mg, 0.45 mmol, 1.5 equiv.). The title product was obtained after purification by column chromatography (hexane/EtOAc, 20:1) as a colorless oil (40.4 mg, 0.189 mmol, 63%).

R$_f$ = 0.21 (Hexane/EtOAc, 20:1). **^{1}H NMR** (600 MHz, $CDCl_3$) δ/ ppm = 7.82 (d, *J* = 7.6 Hz, 1H), 7.74 (d, *J* = 7.6 Hz, 1H), 7.47 – 7.40 (m, 2H), 7.34 (d, *J* = 3.7 Hz, 1H), 7.19 – 7.14 (m, 1H), 3.33 (t, *J* = 5.7 Hz, 2H), 2.76 (t, *J* = 5.7 Hz, 2H). **^{13}C NMR** (151 MHz, $CDCl_3$) δ/ ppm = 207.0, 151.8, 141.1, 138.2, 133.8, 133.2, 128.2, 127.9, 126.1, 126.0, 123.0, 36.4, 26.9. **HRMS** (ESI) calculated for $C_{13}H_{11}OS$: 237.0345 $[M+H]^+$, found: 237.0344.

2,3,4,5,6-Pentafluoro-1,1'-biphenyl

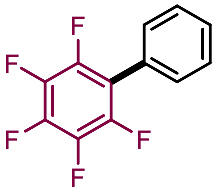

According to GP 4 the cross coupling was performed using triethyl(perfluorophenyl)germane (98.1 mg, 0.30 mmol, 1.0 equiv.), iodobenzene (50.0 µL, 0.45 mmol, 1.5 equiv.), Ag_2CO_3 (128.1 mg, 0.45 mmol, 1.5 equiv.) and $Pd(PPh_3)_4$ (8.7 mg, 0.0075 mmol, 2.5 mol%). The title product was obtained after purification by column chromatography (hexane) as a white solid (66.7 mg, 0.273 mmol, 91%).

R$_f$ = 0.76 (Hexane). **^{1}H NMR** (600 MHz, $CDCl_3$) δ/ ppm = 7.85 – 7.34 (m, 5H). **^{13}C NMR** (151 MHz, $CDCl_3$) δ/ ppm = 144.3 (d, *J* = 247.6 Hz), 140.5 (d, *J* = 253.6 Hz), 138.0 (d, *J* = 251.4 Hz), 130.3, 129.5, 128.9, 116.2 – 115.9 (m). **^{19}F NMR** (564 MHz, $CDCl_3$) δ/ = -142.7 – -143.5 (m, 2F), -155.1 – -156.0 (m, 1F), -161.7 – -162.7 (m, 2F). **HRMS** (EI) calculated for $C_{12}H_5F_5$: 244.0311 $[M]^+$, found: 244.0315.

These data are in agreement with those reported previously in the literature.[16]

2,3,4,5,6-Pentafluoro-4'-methoxy-1,1'-biphenyl

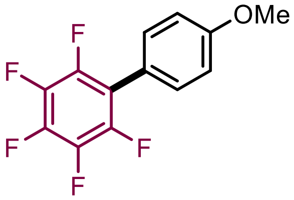

According to GP 4 the cross coupling was performed using triethyl(perfluorophenyl)germane (98.1 mg, 0.30 mmol, 1.0 equiv.) and 1-iodo-4-methoxybenzene (105 mg, 0.45 mmol, 1.5 equiv.). The title product was obtained after purification by column chromatography (pentane) as a white solid (73.4 mg, 0.297 mmol, 99%).

R$_f$ = 0.49 (Pentane). **^{1}H NMR** (600 MHz, $CDCl_3$) δ/ ppm = 7.38 – 7.33 (m, 2H), 7.05 – 6.99 (m, 2H), 3.87 (s, 3H). **^{13}C NMR** (151 MHz, $CDCl_3$) δ/ ppm = 160.4, 144.3 (d, *J* = 246.2 Hz), 141.0, 138.0 (d, *J* = 248.4 Hz), 131.6, 118.5, 115.9 (d, *J* = 19.3 Hz), 114.4, 55.5. **^{19}F NMR** (564 MHz, $CDCl_3$) δ/ ppm = -143.7 (m, 2F), -156.5 (m, 1F), -162.5 (m, 2F). **HRMS** (EI) calculated for $C_{13}H_7OF_5$: 274.0412 $[M]^+$, found: 274.0416.

These data are in agreement with those reported previously in the literature.[17]

3-Bromo-4'-methyl-1,1'-biphenyl

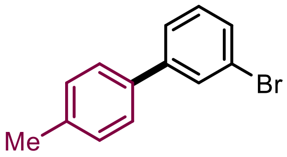

According to GP 4 the cross coupling was performed using triethyl(*p*-tolyl)germane (75.3 mg, 0.3 mmol, 1.0 equiv.) and 1-bromo-3-iodobenzene (127 mg, 0.45 mmol, 1.5 equiv.). The title product was obtained after purification by column chromatography (hexane), and further purification by prep-HPLC (hexane). *The mixture contains 1-bromo-3-iodobenzene as unconsumed and inseparable starting material. The yield was determined by quantitative ^{1}H NMR.*

R$_f$ = 0.77 (Hexane). **^{1}H NMR** (600 MHz, $CDCl_3$) δ/ppm = 7.73 – 7.71 (m, 1H), 7.52 – 7.48 (m, 1H), 7.48 – 7.43 (m, 3H), 7.34 – 7.23 (m, 4H), 2.40 (s, 3H). **^{13}C NMR** (151 MHz, $CDCl_3$) δ/ppm = 143.4, 137.9, 137.0, 130.4, 130.1, 130.0, 129.8, 127.1, 125.7, 123.0, 21.3. **MS** (70eV, EI): *m/z* (%): 249.0 (13), 248.0 (93) $[M]^+$ (^{81}Br), 247.0 (23), 246.0 (94) $[M]^+$ (^{79}Br), 245.0 (10), 167.1 (57), 166.1 (39), 165.1 (100), 164.1 (15), 152.1 (62), 151.1 (12), 139.1 (22), 115.1 (18), 89.0 (10), 82.3 (17), 74.1 (12), 63.1 (17), 50.1 (13).

These data are in agreement with those reported previously in the literature.[18]

3'-Bromo-4'-fluoro-[1,1'-biphenyl]-4-yl trifluoromethanesulfonate

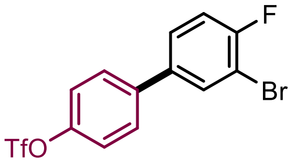

According to GP 4 the cross coupling was performed using 4-(triethylgermyl)phenyl trifluoromethanesulfonate (116 mg, 0.3 mmol, 1.0 equiv.) and 2-bromo-1-fluoro-4-iodobenzene (135 mg, 0.45 mmol, 1.5 equiv.). The title product was obtained after purification by column chromatography (hexane) as an orange oil (53.8 mg, 0.135 mmol, 90%).

$\mathbf{R_f}$ = 0.34 (Hexane). **^{1}H NMR** (600 MHz, $CDCl_3$) δ/ ppm = 7.71 – 7.59 (m, 3H), 7.37 (d, *J* = 8.8 Hz, 2H), 7.32 (dd, *J* = 9.5, 2.1 Hz, 1H), 7.23 (dd, *J* = 8.3, 2.1 Hz, 1H). **^{13}C NMR** (151 MHz, $CDCl_3$) δ/ ppm = 159.5 (d, *J* = 247.5 Hz), 149.5, 140.7 (d, *J* = 6.9 Hz), 139.5, 134.2, 128.9, 124.1 (d, *J* = 3.4 Hz), 122.1, 118.9 (q, *J* = 320.9 Hz), 115.3 (d, *J* = 23.0 Hz), 109.1 (d, *J* = 20.8 Hz). **^{19}F NMR** (564 MHz, $CDCl_3$) δ/ ppm = -72.74, -106.40 (dd, *J* = 9.5, 7.1 Hz). **HRMS** (EI) calculated for $C_{13}H_7{}^{79}BrF_4O_3S$: 397.9230 $[M]^+$, found: 397.9230.

3-Bromo-5-chloro-4'-fluorobiphenyl

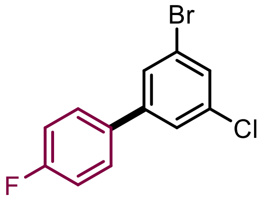

According to GP 4 the cross coupling was performed using triethyl(4-fluorophenyl)germane (76.5 mg, 0.3 mmol, 1.0 equiv.) and 1-bromo-3-chloro-5-iodobenzene (143 mg, 0.45 mmol, 1.5 equiv.). The title product was obtained after purification by column chromatography (pentane) as a white solid (69.0 mg, 0.242 mmol, 81%).

$\mathbf{R_f}$ = 0.70 (Pentane). **^{1}H NMR** (400 MHz, $CDCl_3$) δ/ ppm = 7.56 – 7.42 (m, 5H), 7.18 – 7.08 (m, 2H). **^{13}C NMR** (101 MHz, $CDCl_3$) δ/ ppm = 163.2 (d, *J* = 248.7 Hz), 143.6, 135.6, 134.7 (d, *J* = 2.8 Hz), 130.1, 128.9 (d, *J* = 8.2 Hz), 128.6, 126.1, 123.3, 116.2 (d, *J* = 21.6 Hz).
^{19}F NMR (376 MHz, $CDCl_3$) δ/ ppm = -113.6. **HRMS** (EI) calculated for $C_{12}H_{17}{}^{79}Br^{35}ClF$: 283.9398 $[M]^+$, found: 283.9403. These data are in agreement with those reported previously in the literature.[19]

4-Bromo-3,4'-difluoro-1,1'-biphenyl

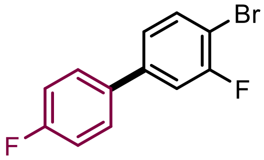

According to GP 4 the cross coupling was performed using triethyl(4-fluorophenyl)germane (76.5 mg, 0.3 mmol, 1.0 equiv.) and 1-bromo-2-fluoro-4-iodobenzene (135.4 mg, 0.45 mmol, 1.5 equiv.). The title product was obtained after purification by column chromatography (Hexane) as a white solid as an inseparable mixture of the desired product (0.270 mmol, 90%) and 4,4'-dibromo-3,3'-difluoro-1,1'-biphenyl as sideproduct (0.024 mmol, 8%).
NMR yields with 1,4-difluorobiphenyl as internal standard.

$\mathbf{R_f}$ = 0.83 (Hexane). **^{1}H NMR** (600 MHz, $CDCl_3$) δ/ ppm = 7.59 (dd, *J* = 7.8 Hz, 1H), 7.56 – 7.47 (m, 2H), 7.29 (dd, *J* = 9.8, 2.1 Hz, 1H), 7.20 (dd, *J* = 8.2, 2.1 Hz, 1H), 7.14 (dd, *J* = 8.6 Hz, 2H). **^{13}C NMR** (151 MHz, $CDCl_3$) δ/ ppm = 163.0 (d, *J* = 248.1 Hz), 159.5 (d, *J* = 247.3 Hz), 141.8 (d, *J* = 7.2 Hz), 134.9 (d, *J* = 153.1 Hz), 133.9, 128.73 (d, *J* = 8.1 Hz), 123.9 (d, *J* = 3.5 Hz), 116.1 (d, *J* = 21.5 Hz), 115.1 (d, *J* = 22.8 Hz), 108.0 (d, *J* = 21.0 Hz). **^{19}F NMR** (282 MHz, $CDCl_3$) δ/ ppm = -106.90 – -107.25 (m, 1F), -113.92 – -114.19 (m, 1F).

1-(4-Bromophenyl)naphthalene

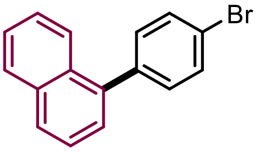

According to GP 4 the cross coupling was performed using triethyl(naphthalen-1-yl)germane (86.1 mg, 0.3 mmol, 1.0 equiv.) and 1-bromo-4-iodobenzene (127 mg, 0.45 mmol, 1.5 equiv.). The reaction was performed under anaerobic conditions (argon atmosphere) and under aerobic conditions. The title product was obtained after purification by column chromatography (hexane) as a white solid.

Anaerobic conditions: 65% yield (55.0 mg, 0.194 mmol)
Aerobic conditions: 72% yield (61.0 mg, 0.215 mmol)

R_f = 0.72 (Hexane). **^{1}H NMR** (600 MHz, $CDCl_3$) δ/ ppm = 7.97 – 7.83 (m, 3H), 7.64 (d, *J* = 8.2 Hz, 2H), 7.59 – 7.50 (m, 2H), 7.46 (ddd, *J* = 8.2, 6.7, 1.4 Hz, 1H), 7.43 – 7.35 (m, 3H). **^{13}C NMR** (151 MHz, $CDCl_3$) δ/ ppm = 139.8, 139.0, 133.9, 131.8, 131.6, 131.5, 128.5, 128.2, 127.0, 126.4, 126.1, 125.8, 125.5, 121.6. **HRMS** (EI) calculated for $C_{16}H_{11}{}^{79}Br$: 282.0039 $[M]^+$, found: 282.0034. These data are in agreement with those reported previously in the literature.[20]

4-Fluoro-4’-methylbiphenyl

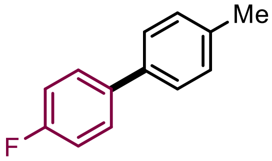

According to GP 4 the cross coupling was performed using triethyl(4-fluorophenyl)germane (76.5 mg, 0.3 mmol, 1.0 equiv.) and 4-iodotoluene (98.1 mg, 0.45 mmol, 1.5 equiv.). The reaction was performed under anaerobic conditions (argon atmosphere) and under aerobic conditions. The title product was obtained after purification by column chromatography (hexane) as a white solid.

Anaerobic conditions: 58% yield (32.2 mg, 0.174 mmol)
Aerobic conditions: 60% yield (33.5 mg, 0.180 mmol)

R_f = 0.31 (Hexane). **^{1}H NMR** (600 MHz, $CDCl_3$) δ/ ppm = 7.56 – 7.49 (m, 2H), 7.46 – 7.41 (m, 2H), 7.24 (d, *J* = 8.0 Hz, 2H), 7.14 – 7.07 (m, 2H), 2.39 (d, *J* = 2.7 Hz, 3H). **^{13}C NMR** (151 MHz, $CDCl_3$) δ/ ppm = 162.3 (d, *J* = 245.8 Hz), 137.4, 137.3 (d, *J* = 4.6 Hz), 137.0, 129.5, 128.5 (d, *J* = 7.7 Hz), 126.8, 115.6 (d, *J* = 21.1), 21.1. **HRMS** (EI): calculated for $C_{13}H_{11}F$: 186.0845 [M]+, found: 186.0840. These data are in agreement with those reported previously in the literature.[21]

2.4.3 Reaction with Diaryl Iododonium Salts

4-Fluoro-4'-methoxy-1,1'-biphenyl

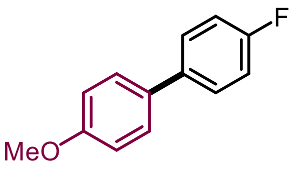

According to GP 6 the cross coupling was performed using triethyl(4-methoxyphenyl)germane (80.7 mg, 0.3 mmol, 1.0 equiv.) and bis(4-fluorophenyl)iodonium tetrafluoroborate (182 mg, 0.45 mmol, 1.5 equiv.). The title product was obtained after purification by column chromatography (hexane) as a white solid (42.5 mg, 0.210 mmol, 70%).

R$_f$ = 0.30 (Hexane/EtOAc 95:5). **^{1}H NMR** (400 MHz, $CDCl_3$) δ/ ppm = 7.54 – 7.43 (m, 4H), 7.15 – 7.05 (m, 2H), 7.03 – 6.93 (m, 2H), 3.85 (s, 3H). **^{13}C NMR** (101 MHz, $CDCl_3$) δ/ ppm = 163.3, 160.9, 159.1, 132.8, 128.2 (d, *J* = 7.8 Hz), 128.0, 115.5 (d, J = 21.2 Hz), 114.2, 55.3. **^{19}F NMR** (376 MHz, $CDCl_3$) δ/ ppm = -116.79 (tt, *J* = 8.9, 5.3 Hz). **HRMS** (EI) calculated for $C_{13}H_{11}FO$: 202.0794 $[M]^+$, found: 202.0794.

These data are in agreement with those reported previously in the literature.[22]

9-(4-Fluorophenyl)phenanthrene

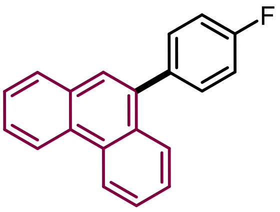

According to GP 6 the cross coupling was performed using triethyl(phenanthren-9-yl)germane (101 mg, 0.3 mmol, 1.0 equiv.) and bis(4-fluorophenyl)iodonium tetrafluoroborate (182 mg, 0.45 mmol, 1.5 equiv.). The title product was obtained after purification by column chromatography (hexane/DCM) as a colorless oil (60.3 mg, 0.222 mmol, 74%).

R$_f$ = 0.25 (Hexane/DCM 15:1). **^{1}H NMR** (600 MHz, $CDCl_3$) δ/ ppm = 8.79 (d, *J* = 8.3 Hz, 1H), 8.73 (d, *J* = 8.3 Hz, 1H), 7.90 (d, *J* = 7.7 Hz, 1H), 7.87 (d, *J* = 7.7 Hz, 1H), 7.70 – 7.66 (m, 3H), 7.63 (ddd, *J* = 8.0, 7.0, 1.3 Hz, 1H), 7.55 (ddd, *J* = 8.0, 7.0, 1.2 Hz, 1H), 7.53 – 7.49 (m, 2H), 7.24 – 7.19 (m, 2H). **^{13}C NMR** (151 MHz, $CDCl_3$) δ/ ppm = 162.3 (d, *J* = 246.0 Hz), 137.7, 136.7 (d, *J* = 3.7 Hz), 131.6 (d, *J* = 8.1 Hz), 131.5, 131.1, 130.6, 123.0, 128.6, 127.7, 126.9, 126.7, 126.7, 126.6, 126.5, 123.0, 122.5, 115.2 (d, *J* = 21.2 Hz). **^{19}F NMR** (376 MHz, $CDCl_3$) δ/ ppm = -115.26 (td, *J* = 8.8, 4.4 Hz). **MS** (70 eV, EI): *m/z* (%): 272 (100) $[M]^+$, 271 (57), 270 (43), 273 (21), 135 (13), 268 (11).

These data are in agreement with those reported previously in the literature.[23]

4-Methoxybiphenyl

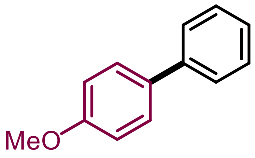

According to GP 6 the cross coupling was performed using triethyl(4-methoxyphenyl)germane (80.7 mg, 0.3 mmol, 1.0 equiv.) and diphenyliodonium tetrafluoroborate (166 mg, 0.45 mmol, 1.0 equiv.). The title product was obtained after purification by column chromatography (hexane/EtOAc 20:1) as a white solid (41.1 mg, 0.23 mmol, 75%).

R$_f$ = 0.33 (Hexane/EtOAc 20:1). **^{1}H NMR** (400 MHz, $CDCl_3$) δ/ ppm = 7.60 – 7.50 (m, 4H), 7.47 – 7.37 (m, 2H), 7.35 – 7.26 (m, 1H), 7.03 – 6.95 (m, 2H), 3.86 (s, 3H). **^{13}C NMR** (101 MHz, $CDCl_3$) δ/ ppm = 159.1, 140.8, 133.8, 128.7, 128.1, 126.7, 126.6, 114.2, 55.3. **HRMS** (EI) calculated for $C_{13}H_{12}O$: 184.0888 $[M]^+$, found: 184.0882.

These data are in agreement with those reported previously in the literature.[6]

3-Fluoro-2-phenylpyridine

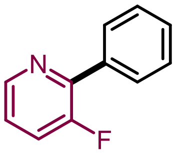

According to GP 6 the cross coupling was performed using 3-fluoro-2-(triethylgermyl)pyridine (76.8 mg, 0.3 mmol, 1.0 equiv.) and diphenyliodonium tetrafluoroborate (166 mg, 0.45 mmol, 1.5 equiv.). The title product was obtained after purification by column chromatography (DCM) as a yellow oil (22.3 mg, 0.129 mmol, 43% yield).

R$_f$ = 0.36 (DCM). **^{1}H NMR** (600 MHz, $CDCl_3$) δ/ ppm = 8.53 (d, *J* = 4.5 Hz, 1H), 8.02 – 7.91 (m, 2H), 7.52 – 7.40 (m, 4H), 7.29 – 7.24 (m, 1H). **^{13}C NMR** (151 MHz, $CDCl_3$) δ/ ppm = 157.5 (d, *J* = 260.4 Hz), 145.4 (d, *J* = 5.2 Hz), 135.3 (d, *J* = 5.1 Hz), 129.2, 128.8 (d, *J* = 5.8 Hz), 128.5, 124.2, 124.0, 123.4 (d, *J* = 4.0 Hz). **^{19}F NMR** (564 MHz, $CDCl_3$) δ/ ppm = -123.00 (d, *J* = 11.3 Hz). **HRMS** (ESI) calculated for $C_{11}H_8FN$: 174.0714 $[M+H]^+$, found: 174.0710

These data are in agreement with those reported previously in the literature.[24]

5-Bromo-2-phenylpyridine

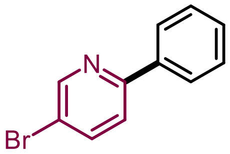

According to GP 6 the cross coupling was performed using 5-bromo-2-(triethylgermyl)pyridine (95.1 mg, 0.3 mmol, 1.0 equiv.) and diphenyliodonium tetrafluoroborate (166 mg, 0.45 mmol, 1.0 equiv.). The title product was obtained by purification by column chromatography (hexane/EtOAc 7:1), and further purification by prep-HPLC (hexane/EtOAc, 4:1) as an off-white solid (42.2 mg, 0.18 mmol, 60%).

R$_f$ = 0.45 (Hexane/EtOAc 7:1) **M.p.** 72-73 °C.**^{1}H NMR** (600 MHz, $CDCl_3$) δ/ ppm = 8.74 (dd, *J* = 2.4, 0.8 Hz, 1H), 7.99 – 7.94 (m, 2H), 7.87 (dd, *J* = 8.5, 2.4 Hz, 1H), 7.63 (dd, *J* = 8.5, 0.8 Hz, 1H), 7.50 –

7.46 (m, 2H), 7.45 – 7.43 (m, 1H). **^{13}C NMR** (151 MHz, $CDCl_3$) δ/ ppm = 156.1, 150.8, 139.4, 138.4, 129.5, 129.0, 126.9, 121.8, 119.5. **HRMS** (ESI) calculated for $C_{11}H_8{}^{79}BrN$: 233.9913 $[M+H]^+$, found: 233.9912.

These data are in agreement with those reported previously in the literature.[25]

2.4.4 Gram Scale Reaction

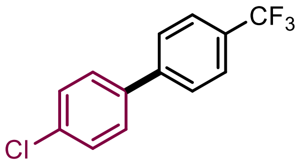

The cross coupling was performed using triethyl(4-chloro)germane (1.00 g, 3.68 mmol, 1.0 equiv.), 4-benzotrifluoride (811.2 µL, 5.52 mmol, 1.5 equiv.) with Pd_2dba_3 (1.68 mg, 0.00184 mmol, 0.1 mol% [Pd]) and $AgBF_4$ (1.074 g, 5.52 mmol, 1.5 equiv.) in 10 mL DMF. After 72 hours, the product was purified by column chromatography (hexane) to give a white solid (0.909 g, 3.54 mmol, 96%).

R_f = 0.35 (Hexane). **^{1}H NMR** (600 MHz, $CDCl_3$) δ/ ppm = 7.70 (d, *J* = 8.3 Hz, 2H), 7.65 (d, *J* = 8.2 Hz, 2H), 7.56 – 7.51 (m, 2H), 7.47 – 7.42 (m, 2H). **^{13}C NMR** (151 MHz, $CDCl_3$) δ/ ppm = 143.5, 138.2, 134.4, 129.7 (q, *J* = 32.6 Hz), 129.2, 128.5, 127.3, 125.8 (q, *J* = 3.7 Hz), 124.2 (q, *J* = 271.9 Hz). **^{19}F NMR** (376 MHz, $CDCl_3$) δ/ ppm = -62.47. **HRMS** (EI) calculated for $C_{13}H_8F_3{}^{35}Cl$: 256.0267 $[M]^+$, found: 256.0252

These data are in agreement with those reported previously in the literature

2.4.5 Preformation of Nanoparticles/Cross Coupling

4'-Methoxy-2-methyl-1,1'-biphenyl

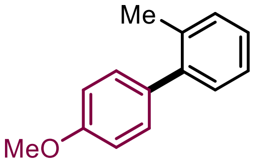

According to GP 7 the cross coupling was performed using triethyl(4-methoxy)germane (80.7 mg, 0.3 mmol, 1.0 equiv.) and 2-iodotoluene (57.3 µL, 0.45 mmol, 1.5 equiv.). The title product was obtained after purification by column chromatography (hexane/ EtOAc 95:5) as a yellow oil (30.5 mg, 0.156 mmol, 52%).

R$_f$ = 0.45 (Hexane/EtOAc 95:5). **^{1}H NMR** (400 MHz, $CDCl_3$) δ/ ppm = 7.25 – 7.20 (m, 6H), 6.98 – 6.90 (m, 2H), 3.84 (s, 3H), 2.26 (s, 3H). **^{13}C NMR** (151 MHz, $CDCl_3$) δ/ ppm = 158.5, 141.6, 135.5, 134.4, 130.3, 130.2, 129.9, 127.0, 125.7, 113.5, 55.3, 20.5. **HRMS** (EI) calculated for $C_{14}H_{14}O$: 198.1045 $[M]^+$, found: 198.1037.

These data are in agreement with those reported previously in the literature.[26]

4-Acetyl-4'-fluoro-biphenyl

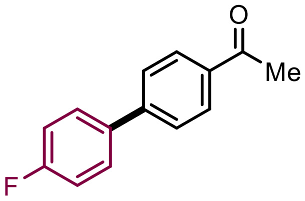

According to GP 7 the cross coupling was performed using triethyl(4-fluorophenyl)germane (76.5 mg, 0.3 mmol, 1.0 equiv.) and 4'-iodoacetophenone (110.7 mg, 0.45 mmol, 1.5 equiv.). The title product was obtained after purification by column chromatography (hexane/ EtOAc 7:1) as a white solid (43.7 mg, 0.204 mmol, 68%).

R$_f$ = 0.25 (Hexane/EtOAc 7:1). **^{1}H NMR** (400 MHz, $CDCl_3$) δ/ ppm = 8.16 – 7.93 (m, 2H), 7.66 – 7.62 (m, 2H), 7.62 – 7.57 (m, 2H), 7.22 – 7.10 (m, 2H), 2.64 (s, 3H). **^{13}C NMR** (151 MHz, $CDCl_3$) δ/ ppm = 197.7, 162.0 (d, J = 248.0 Hz), 144.7, 136.0 (d, J = 3.1 Hz), 135.8, 129.0 – 128.8 (m), 127.1, 115.9 (d, J = 21.9 Hz), 26.7. **^{19}F NMR** (376 MHz, $CDCl_3$) δ/ ppm = -114.08 (tt, J = 9.1, 5.4 Hz). **HRMS** (ESI) calculated for $C_{14}H_{11}FNaO$: 237.0686 $[M+Na]^+$, found: 237.0682

These data are in agreement with those reported previously in the literature.[27]

1-(3-Fluorophenyl)naphthalene

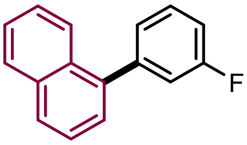

According to GP 7 using triethyl(naphthalen-1-yl)germane (86.1 mg, 0.3 mmol, 1.0 equiv.) and 3-fluoro iodobenzene (52.9 µL, 0.45 mmol, 1.5 equiv.). The title product was obtained after purification by column chromatography (hexane) as a colorless oil (61.0 mg, 0.273 mmol, 91%).

R$_f$ = 0.22 (Hexane). **^{1}H NMR** (600 MHz, $CDCl_3$) δ/ ppm = 7.94 – 7.91 (m, 1H), 7.91 – 7.87 (m, 2H), 7.57 – 7.49 (m, 2H), 7.50 – 7.43 (m, 2H), 7.45 – 7.40 (m, 1H), 7.32 – 7.26 (m, 1H), 7.22 (ddd, J = 9.8,

2.0, 2.0 Hz, 1H), 7.14 (ddd, J = 8.5, 8.5, 2.6 Hz, 1H). **^{13}C NMR** (151 MHz, $CDCl_3$) δ/ ppm = 162.7 (d, J = 246.1 Hz), 143.0 (d, J = 7.5 Hz), 138.9, 133.8, 131.3, 129.7 (d, J = 8.5 Hz), 128.4, 128.1, 126.9, 126.3, 125.9, 125.8 (d, J = 3.0 Hz), 125.7, 125.3, 117.0 (d, J = 21.5 Hz), 114.1 (d, J = 20.9 Hz). **^{19}F NMR** (282 MHz, $CDCl_3$) δ/ ppm = -113.53 (td, J = 9.0, 5.6 Hz). **HRMS** (EI) calculated for $C_{16}H_{11}F$: 222.0845 $[M]^+$, found: 222.0843.

4-Methoxy-4'-(trifluoromethyl)-1,1'-biphenyl

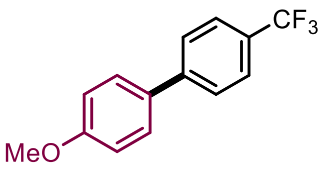

According to GP 7 the cross coupling was performed using triethyl(4-methoxy)germane (80.7 mg, 0.3 mmol, 1.0 equiv.) and bis(4-trifluoromethyl)phenyl iodonium tetrafluoroborate (227 mg, 0.45 mmol, 1.5 equiv.). The title product was obtained after purification by column chromatography (hexane/EtOAc) as a white solid (45.1 mg, 0.179 mmol, 60%).

R_f = 0.50 (Hexane/EtOAc 95:5). **^{1}H NMR** (400 MHz, $CDCl_3$) δ/ ppm = 7.70 – 7.61 (m, 4H), 7.55 (d, J = 8.8 Hz, 2H), 7.01 (d, J = 8.8 Hz, 2H), 3.87 (s, 3H). **^{13}C NMR** (101 MHz, $CDCl_3$) δ/ ppm = 159.8, 144.3, 132.2, 128.7 (d, J = 32.7 Hz), 128.4, 126.9, 125.7 (q, J = 3.8 Hz), 114.4, 55.4. *CF_3 quartet not fully visible.* **^{19}F NMR** (376 MHz, $CDCl_3$) δ/ ppm = -62.36. **HRMS** (EI) calculated for $C_{10}H_{11}F_3O$: 252.0762 $[M]^+$, found: 252.0751.
These data are in agreement with those reported previously in the literature.[28]

4-Chloro-4'-(trifluoromethyl)-1,1'-biphenyl

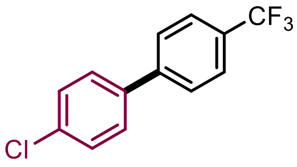

According to GP 7 the cross coupling was performed using triethyl(4-chloro)germane (81.4 mg, 0.3 mmol, 1.0 equiv.) and bis(4-trifluoromethyl)phenyl iodonium tetrafluoroborate (227 mg, 0.45 mmol, 1.5 equiv.). The title product was obtained after purification by column chromatography (hexane) as a white solid (66.4 mg, 0.259 mmol, 86%).

The analytical data matches the ones previously reported in this manuscript.

2.4.6 Intramolecular competition

Trimethyl(3'-nitro-[1,1'-biphenyl]-4-yl)silane

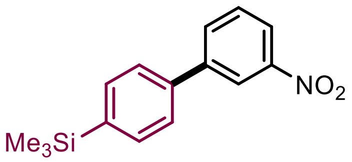

According to GP 4 the cross coupling was performed using trimethyl(4-(triethylgermyl)phenyl)silane (92.7 mg, 0.30 mmol, 1.0 equiv.) and 1-iodo-3-nitrobenzene (74.7 mg, 0.3 mmol, 1.0 equiv.). The title product was obtained after purification by column chromatography (hexane) as a white solid (74.2 mg, 0.273 mmol, 91%).

R$_f$ = 0.16 (Hexane). **M.p.** = 72.9 °C. **^{1}H NMR** (600 MHz, $CDCl_3$) δ/ ppm = 8.47 (dd, *J* = 2,0, 2.0 Hz, 1H), 8.23 – 8.18 (m, 1H), 7.92 (d, *J* = 7.7 Hz, 1H), 7.69 – 7.64 (m, 2H), 7.64 – 7.61 (m, 2H), 0.32 (s, 9H). **^{13}C NMR** (151 MHz, $CDCl_3$) δ/ ppm = 148.9, 143.0, 141.2, 139.1, 134.3, 133.2, 129.9, 126.6, 122.2, 122.1, -1.0. **HRMS** (EI) calculated for $C_{15}H_{17}NO_2Si$: 271.1029 $[M]^+$, found: 271.1023.

2-(4'-Methoxy-[1,1'-biphenyl]-4-yl)-4,4,5,5-tetramethyl-1,3,2-dioxaborolane

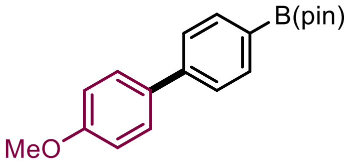

According to GP 4 the cross coupling was performed using triethyl(4-methoxy)germane (80.7 mg, 0.3 mmol, 1.0 equiv.) and 2-(4-iodophenyl)- 4,4,5,5-tetramethyl- 1,3,2-dioxaborolane (99.0 mg, 0.3 mmol, 1.0 equiv.). The title product was obtained following purification by column chromatography (hexane/EtOAc 20:1) as a white solid (76.0 mg, 0.218 mmol, 73%).

R$_f$ = 0.59 (DCM). **^{1}H NMR** (600 MHz, $CDCl_3$) δ/ ppm = 7.86 (d, *J* = 8.2 Hz, 2H), 7.60 – 7.54 (m, 4H), 6.98 (d, *J* = 8.8 Hz, 2H), 3.86 (s, 3H), 1.36 (s, 12H). **^{13}C NMR** (151 MHz, $CDCl_3$) δ/ ppm = 159.4, 143.5, 135.3, 133.5, 128.3, 126.0, 114.2, 83.8, 55.4, 55.3, 24.9. **HRMS** (EI) calculated for $C_{19}H_{23}O_3B$: 310.1740 $[M]^+$, found: 310.1737.
These data are in agreement with those reported previously in the literature.[29]

4,4,5,5-Tetramethyl-2-(4'-(trifluoromethyl)-[1,1'-biphenyl]-4-yl)-1,3,2-dioxaborolane

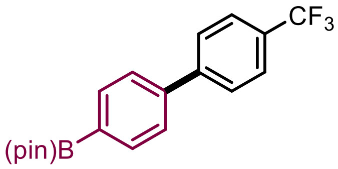

According to GP 4 the cross coupling was performed using triethyl (4-(4,4,5,5-tetramethyl- 1,3,2-dioxaborolan-2-yl) phenyl)germane (109 mg, 0.30 mmol, 1.0 equiv.) and 1-iodo-4-(trifluoromethyl)benzene (81.6 mg, 0.3 mmol, 1.0 equiv.). The title product was obtained after purification by column chromatography (hexane/EtOAc 20:1) as a white solid (76.0 mg, 0.218 mmol, 73%).

R_f = 0.39 (Hexane/EtOAc, 20:1). **^{1}H NMR** (600 MHz, $CDCl_3$) δ/ ppm = 7.91 (d, *J* = 8.1 Hz, 2H), 7.75 – 7.69 (m, 4H), 7.61 (d, *J* = 8.1 Hz, 2H), 1.37 (s, 12H). **^{13}C NMR** (151 MHz, $CDCl_3$) δ/ ppm = 144.7, 142.5, 135.6, 129.7 (q, *J* = 32.4 Hz), 127.7, 127.1, 126.7, 125.9 (q, *J* = 3.8 Hz), 124.4 (q, *J* = 272.2 Hz), 84.1, 25.0. **^{19}F NMR** (564 MHz, $CDCl_3$) δ/ ppm = -62.4. **HRMS** (ESI) calculated for $C_{19}H_{20}BF_3O_2$: 348.1508 $[M]^+$, found: 348.1512.

These data are in agreement with those reported previously in the literature.[30]

3 Stoichiometric Bond Activation with Pd$^{(II)}$

3.1 Preparation of Pd$^{(II)}$ Complexes

***trans*-Iodo(phenyl)bis(triphenylphosphine)palladium(II) [$(Ph_3P)_2Pd(C_6H_5)I$]**

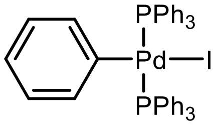

Under Argon atmosphere, $Pd(PPh_3)_4$ (1.16 g, 1.00 mmol, 1.0 equiv.) and iodobenzene (0.31 g, 1.50 mmol, 1.5 equiv.) were added to an oven-dried 50 mL flask. The solids were dissolved in benzene (15 mL) and the reaction was allowed to stir at room temperature for 3 h. The mixture was then diluted with hexane (15 mL). The formed precipitate was filtered and washed with hexane (3 x 10 mL) and dried under vacuum to give the pure product as an off-white solid (0.79 g, 0.94 mmol, 94%).

^{1}H NMR (400 MHz, CD_2Cl_2) δ/ ppm = 7.58 – 7.44 (m, 12H), 7.43 – 7.30 (m, 6H), 7.32 – 7.21 (m, 12H), 6.69 – 6.54 (m, 2H), 6.46 – 6.28 (m, 1H), 6.28 – 6.18 (m, 2H). **^{31}P NMR** (121 MHz, CD_2Cl_2) δ/ ppm = 22.8 (s, 2P).

These data are in agreement with those previously reported in the literature.[31]

***trans*-Fluoro(phenyl)bis(triphenylphosphine)palladium(II) [$(Ph_3P)_2Pd(C_6H_5)F$]**

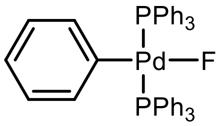

Under Argon atmosphere, [$(Ph_3P)_2Pd(C_6H_5)I$] (450 mg, 0.54 mmol) and silver fluoride (126 mg, 1.00 mmol, 1.85 equiv.) were suspended in benzene (10 mL) and iodobenzene (10 mg) was added in a flat-bottomed brown vial. The vial was sealed and brought out of the glove-box. The reaction was sonicated for 150 min at 18 °C – 20 °C and then brought back inside the glove-box. The reaction mixture was filtered through a pad of celite, washed with toluene (5 mL) and concentrated to 2 mL under vacuum. After addition of hexane (5 mL) the formed precipitate was filtered, washed with hexane (2 x 5 mL) and dried under vacuum to give the pure product as an off-white solid (292 mg, 0.40 mmol, 75%).

^{1}H NMR (600 MHz, $CDCl_3$) δ/ ppm = 7.52 – 7.39 (m, 12H), 7.25 – 7.19 (m, 6H), 7.19 – 7.07 (m, 12H), 6.55 – 6.43 (m, 2H), 6.35 – 6.16 (m, 1H), 6.17 – 6.04 (m, 2H). **^{19}F NMR** (564 MHz, $CDCl_3$) δ/ ppm = -274.0 (brs, 1F). **^{31}P NMR** (243 MHz, $CDCl_3$) δ/ ppm = 19.5 (brs, 2P).

These data are in agreement with those reported previously in the literature.[32]

***trans*-Bromo(phenyl)bis(triphenylphosphine)palladium(II) [$(Ph_3P)_2Pd(C_6H_5)Br$]**

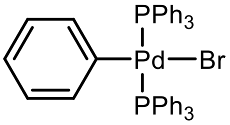

In the glove-box, $Pd(PPh_3)_4$ (0.55 g, 0.475 mmol, 1.0 equiv.) and bromobenzene (0.25 g, 1.60 mmol, 3.4 equiv.) were added to an oven-dried 50 mL flask. The solids were dissolved in benzene (10 mL) and the reaction was allowed to stir at 80 °C for 16 h. The mixture was then cooled to -30 °C for 3 h. The formed precipitate was filtered and washed with Et_2O (3 x 10 mL) and dried under vacuum to give the pure product as a white solid (0.35 g, 0.45 mmol, 95%).

^{1}H NMR (600 MHz, CD_2Cl_2) δ/ ppm = 7.55 – 7.44 (m, 12H), 7.39 – 7.30 (m, 6H), 7.31 – 7.17 (m, 12H), 6.69 – 6.55 (m, 2H), 6.38 – 6.30 (m, 1H), 6.27 – 6.16 (m, 2H). **^{13}C NMR** (151 MHz, CD_2Cl_2) δ/ ppm = 156.6 (t, *J* = 3.3 Hz), 136.7 (t, *J* = 5.0 Hz), 135.2 (t, *J* = 6.2 Hz), 132.1 (t, *J* = 22.8 Hz), 130.3, 128.4 (t, *J* = 5.1 Hz), 128.3, 122.4. **^{31}P NMR** (243 MHz, CD_2Cl_2) δ/ ppm = 23.6 (s, 2P). **HRMS** (ESI) calculated for $C_{42}H_{35}P_2{}^{106}Pd$: 707.1243 $[M-Br]^+$, found: 707.1245.
These data are in agreement with those previously reported in the literature.[33]

***trans*-Chloro[(4-trifluoromethyl)phenyl]bis(triphenylphosphine)palladium(II) [$(Ph_3P)_2Pd($*p*-$CF_3C_6H_4)Cl$]**

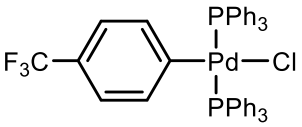

In the glove-box, $Pd(PPh_3)_4$ (0.55 g, 0.475 mmol, 1.0 equiv.) and 4-chlorobenzotrifluoride (0.25 g, 1.38 mmol, 2.9 equiv.) were added to an oven-dried pressure tube. The solids were dissolved in benzene (10 mL) and the reaction was allowed to stir at 135°C for 16 h. The mixture was then cooled down to room temperature, diluted with Et_2O (15 mL) and cooled to -30 °C for 4 h. The formed precipitate was filtered and washed with Et_2O (3 x 10 mL) and dried under vacuum to give the pure product as a yellow solid (0.32 g, 0.39 mmol, 82%).

^{1}H NMR (600 MHz, CD_2Cl_2) δ/ ppm 7.61 – 7.48 (m, 12H), 7.41 – 7.34 (m, 6H), 7.33 – 7.23 (m, 12H), 6.85 – 6.57 (m, 2H), 6.46 – 6.28 (m, 2H). **^{13}C NMR** (151 MHz, CD_2Cl_2) δ/ ppm = 164.1 (t, *J* = 5.3 Hz), 136.7 (t, *J* = 5.1 Hz), 135.1 (t, *J* = 6.3 Hz), 131.3 (t, *J* = 22.8 Hz), 130.7 130.4, 128.6 (t, *J* = 5. Hz), 128.5 (t, *J* = 5.1 Hz), 123.6 (d, *J* = 4.2 Hz). **^{19}F NMR** (564 MHz, CD_2Cl_2) δ/ ppm = -62.4 (s, 3F). **^{31}P NMR** (243 MHz, CD_2Cl_2) δ/ ppm = 24.0 (s, 2P). **HRMS** (ESI) calculated for $C_{43}H_{34}F_3P_2{}^{106}Pd$: 775.1117 $[M-Cl]^+$, found: 775.1125
These data are in agreement with those previously reported in the literature.[33]

***cis/trans*-[(Ph$_3$P)Pd(Ph)(μ-OH)]$_2$**

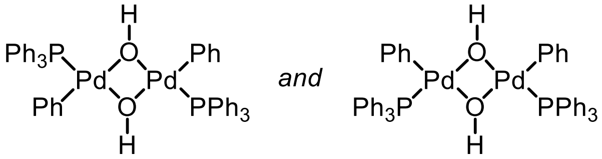

Under argon atmosphere, [(Ph$_3$P)$_2$PdCl$_2$] (1.00 g, 1.4 mmol), iodobenzene (0.62 g, 3.0 mmol, 2.14 equiv.) and KOH (4.00 g) were added to an oven-dried 50 mL flask. The solids were dissolved in benzene (20 mL) and degassed H_2O (4 mL) and the reaction stirred under reflux for 3 h. The hot organic-layer was separated and the aqueous phase was extracted with benzene (3 x 5 mL). The combined organic solution was filtered through cotton and evaporated. The solid was washed thoroughly with acetone (3 x 5 mL) and dried under vacuum to give the pure product as a green solid (520 mg, 0.526 mmol, 75%).

^{1}H NMR (300 MHz, CDCl$_3$) δ/ ppm = 7.52 – 7.20 (m, 15H), 7.11 – 7.00 (m, 2H), 6.73 – 6.57 (m, 3H), -0.25 (s, 0.03H, *OH-cis*), -1.50 (d, *J* = 3.0 Hz, 0.3H), -3.40 (m, 0.03H, *OH-cis*). **^{31}P NMR** (121 MHz, CDCl$_3$ δ/ ppm = 33.9 (s, 2P, *trans*), 33.2 (s, 2P, *cis*). These data are in agreement with those reported previously in the literature.[34]

***cis*-[(dppp)Pd(Ph)(OH)]**

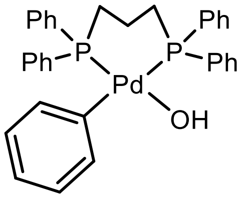

Under argon atmosphere, [(Ph$_3$P)Pd(Ph)(μ-OH)]$_2$ (460 mg, 0.50 mmol, 1.0 equiv.) and 1,3-bis(diphenylphosphino)propane (410 mg, 1.0 mmol, 2.0 equiv.) were added to an oven-dried 25 mL flask. The solids were dissolved in benzene (4 mL) and the reaction was allowed to stir at room temperature for 0.5 h. The solution was evaporated to dryness and the residue was washed with toluene (3 x 10 mL) and hexane (3 x 10 mL) and dried under vacuum to give the pure product as a white solid (490 mg, 0.80 mmol, 80%).

^{1}H NMR (300 MHz, CD$_2$Cl$_2$) δ/ ppm = 8.05 – 7.91 (m, 4H), 7.52 – 7.25 (m, 12H), 7.23 – 7.12 (m, 4H), 7.07 – 6.96 (m, 2H), 6.69 – 6.58 (m, 3H), 2.50 – 2.37 (m, 4H), 1.95 – 1.70 (m, 2H), 0.13 – 0.02 (m, 1H). **^{31}P NMR** (121 MHz, CDCl$_3$) δ/ ppm = 17.3 (d, *J* = 47.6 Hz), -7.8 (d, *J* = 47.6 Hz).
These data are in agreement with those reported previously in the literature.[35]

[(o-tol)$_3$P)Pd(*p*-CF$_3$C$_6$H$_4$)(μ-I)]$_2$

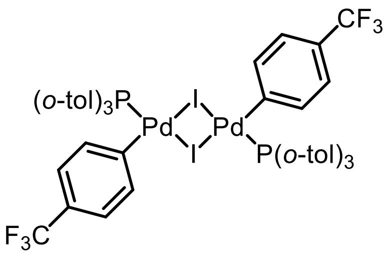

Under an argon atmosphere, Pd$_2$dba$_3$ (1.00 g, 1.08 mmol, 0.5 equiv.), tri-*o*-tolylphosphine (1.32 g, 4.4 mmol, 2.0 equiv.) and 4-iodobenzotrifluoride (1.59 mL, 10.8 mmol, 5.0 equiv.) were stirred at room temperature in 60 mL benzene. After 2 hours, the mixture was filtered through celite and evaporated to approximately 20 mL. 200 mL of anhydrous Et$_2$O was added and the complex allowed to crash out overnight. A small amount of pentane was added and the resulting solid was filtered and washed further with pentane. After drying, the Pd$^{(II)}$ dimer was obtained as a pale orange solid (0.77 g, 0.56 mmol, 52%).

^{1}H NMR (400 MHz, 55 °C, CD$_2$Cl$_2$) δ/ ppm = ppm 7.30 (br), 7.10 (br), 6.72 (br), 2.12 (br). **^{19}F NMR** (376 MHz, 55 °C, CD$_2$Cl$_2$) δ/ ppm = ppm -62.18(s). **^{31}P NMR** (162 MHz, 55 °C, CD$_2$Cl$_2$) δ/ ppm = 24.1. **HRMS** (ESI) calculated for C$_{28}$H$_{25}$P^{106}PdI (monomer): 681.9720 [M]$^+$, found: 681.9607.

3.2 Stoichiometric Bond Activation of Classical Reagents

Transmetalation experiments were performed under an inert atmosphere. The transmetalating agents (1.0 equiv.) were stirred with the palladium complex (1.1 equiv.) at the given time period at room temperature unless otherwise stated. Analysis of the reaction was performed by quantitative ^{19}F NMR.

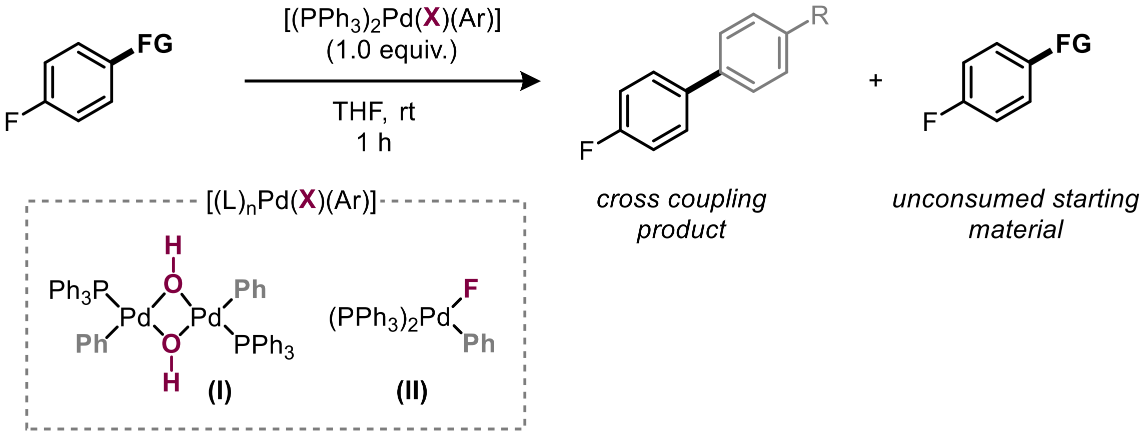

Table S1 – Ar-FG in stoichiometric transmetalation with $Pd^{(II)}$-hydroxo complexes.

entry	FG	Ar–Ar[a]
**1**	Bpin	49%
**4**	$Si(OEt)_3$	85%
**5**	$SnBu_3$	64%
**6**	$B(OH)_2$	64%

[a]Determined by quantitative ^{19}F NMR.

Table S2 – Aryl boronic acid derivatives in stoichiometric transmetalation with $Pd^{(II)}$-fluoride complexes.

entry	FG	Ar–Ar[a]
**2**	Bpin	69%
**3**	B(MIDA)	32%
**7**	$B(OH)_2$[a]	74%

[a]Determined by quantitative ^{19}F NMR. [b]Reaction performed with THF/H_2O (50:1)

3.3 Stoichiometric Bond Activation of Aryl Germanes

Transmetalation experiments were performed under an inert atmosphere. The aryl germane (1.0 equiv.) was stirred with the palladium complex (1.1 equiv.) at the given time period at room temperature unless otherwise stated. Analysis of the reaction was performed by quantitative ^{19}F NMR.

3.3.1 Pd$^{(II)}$-Halide Complexes

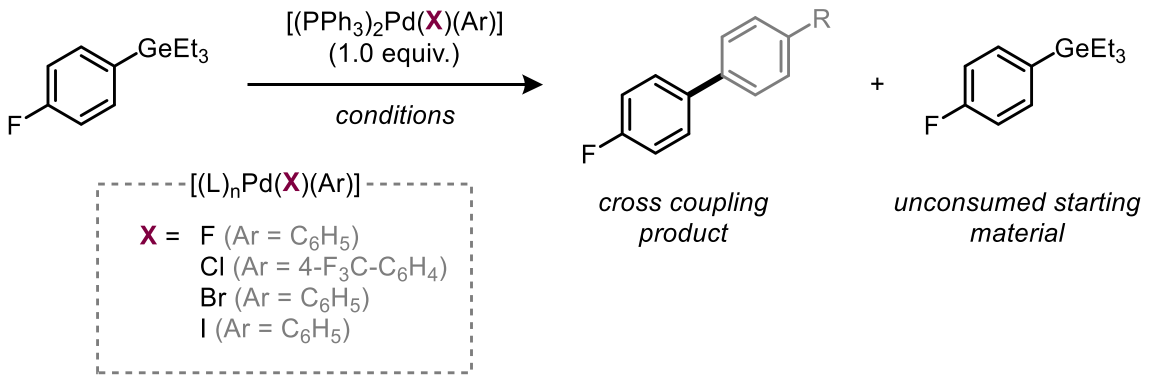

Table S3 – $ArGeEt_3$ in stoichiometric transmetalation with Pd$^{(II)}$-halide complexes.

entry	X	solvent	temp. / °C	Ar–Ar[a]	$ArGeEt_3$ recovered[a]
**1**	**F**	THF	25	0%	> 99%
**2**	**F**	DMF	25	0%	> 99%
**3**	**F**	DMF	80	0%	> 99%
**4**	**F**	toluene	25	0%	> 99%
**5**	**I**	DMF	80	0%	> 99%
**6**	**Br**	DMF	25	0%	> 99%
**7**	**Cl**	DMF	25	0%	> 99%

[a]Determined by quantitative ^{19}F NMR.

3.3.2 Pd$^{(II)}$-Hydroxo Complexes

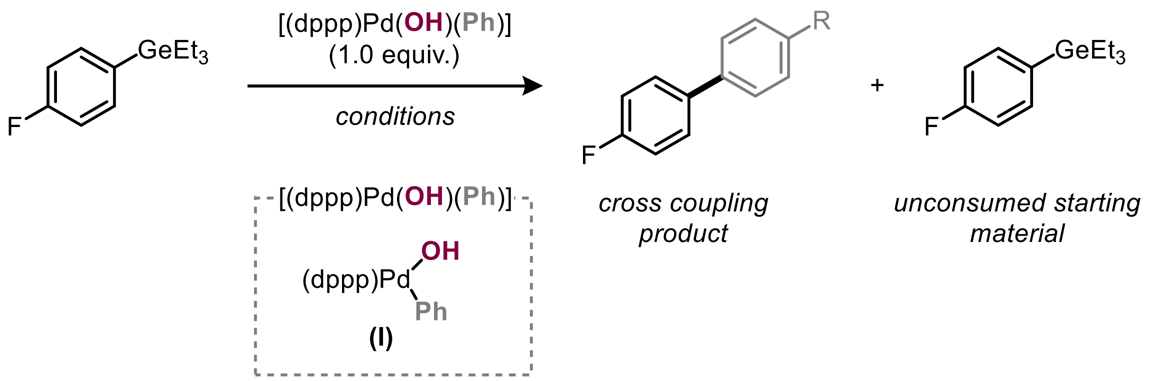

Table S4 – $ArGeEt_3$ in stoichiometric transmetalation with Pd$^{(II)}$-hydroxo complexes.

entry	solvent	additive[a]	temp. / °C	Ar–Ar[b]	$ArGeEt_3$ recovered[b]
**1**	THF	-	25	0%	97%
**2**	DMF	-	25	0%	97%
**3**	DMF	-	80	0%	96%
**4**	DMF	TBAF	25	0%	> 99%
**5**	DMF	CsF	25	0%	> 99%
**6**	DMF	KOH	25	0%	> 99%
**7**	DMF	K_2CO_3	25	0%	> 99%
**8**	DMF	CsOAc	25	0%	> 99%

[a]2.0 equiv. [b]Determined by quantitative ^{19}F NMR.

3.3.3 Pd(II)-Iodo Dimer

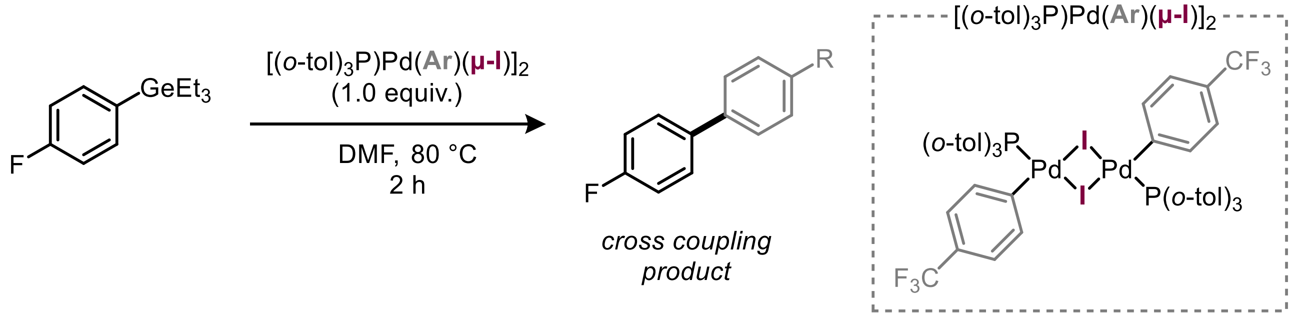

Table S5 – $ArGeEt_3$ in stoichiometric transmetalation with Pd(II)-iodo dimer complexes.

entry	additive[a]	Ar–Ar[b]
**1**	-	0%
**2**	$AgBF_4$	18%

[a]2.0 equiv. [b]Determined by quantitative ^{19}F NMR.

4 Reaction Development

Reactions were carried out as detailed in GP 4. Triethyl(4-fluorophenyl)germane (15.4 mg, 0.06 mmol, 1.0 equiv.) and 4-iodobenzotrifluoride (13.2 µL, 0.09 mmol, 1.5 equiv.) were added to a septum sealed vial. To the vial, the palladium source (5 mol%) and solid additives (0.09 mmol) were added, followed by anhydrous DMF (200 µL). The reaction was heated at 80 °C for 16 hours and the product formation was determined by calibrated GC/MS of the crude reaction mixture.

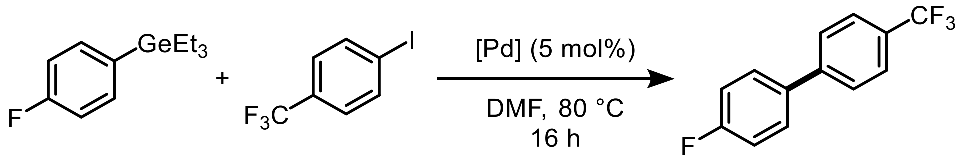

Table S6 – Reaction development

entry	[Pd]	additive[a]	temp. / °C	Ar–Ar[b]
**1**	Pd(II)-iodo dimer	-	80	-
**2**	Pd(II)-iodo dimer	$AgBF_4$	80	85%
**3**	Pd_2dba_3	$AgBF_4$	25	-
**4**	Pd_2dba_3	$AgBF_4$	80	95%
**5**	$Pd(PPh_3)_4$	$AgBF_4$	80	92%

[a]2.0 equiv. [b]Determined by quantitative ^{19}F NMR.

5 Mechanistic Investigations

5.1 Investigation of Induction Period with Various [Pd]/L Ratios

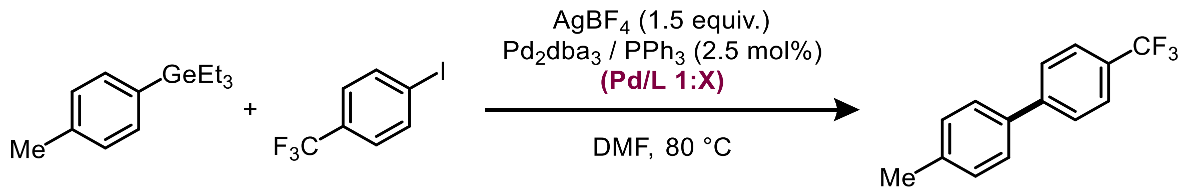

According to GP 4 the cross coupling was performed using triethyl(4-tolyl)germane (26.7 mg, 0.10 mmol, 1.0 equiv.), 1-iodo-4-(trifluoromethyl)benzene (22 µL, 0.15 mmol, 1.5 equiv.), $AgBF_4$ (29.2 mg, 0.15 mmol, 1.5 equiv.) and Pd_2dba_3 (2.3 mg, 0.0025 mmol, 2.5 mol%) in DMF (1 mL). Varying amounts of PPh_3 were added (5 mol%, Pd/L = 1:1; 15 mol%, Pd/L = 1:3). The reaction was monitored by FT-IR using a Mettler Toledo *ReactIR® 15* equipped with a 6.3 mm probe. The relative absorption data over time was normalized to yields obtained by calibrated GC-MS.

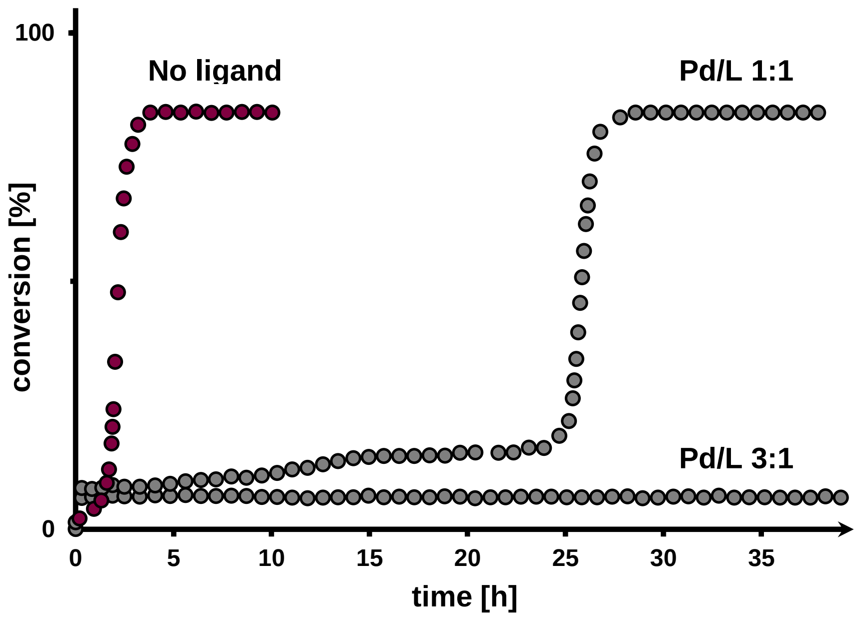

Figure S1 – Reaction profiles with varying amounts of phosphine ligand.

5.2 Investigation of Induction Period with and Without Premixing of Reagents

5.2.1 No Premixing of Reagents

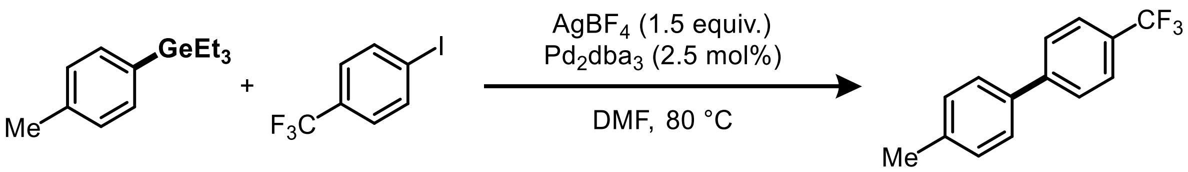

According to GP 4 the cross coupling was performed using triethyl(4-tolyl)germane (26.7 mg, 0.10 mmol, 1.0 equiv.), 1-iodo-4-(trifluoromethyl)benzene (22 µL, 0.15 mmol, 1.5 equiv.), $AgBF_4$ (29.2 mg, 0.15 mmol, 1.5 equiv.) and Pd_2dba_3 (2.3 mg, 0.0025 mmol, 2.5 mol%) in DMF (1 mL). The reaction was monitored by FT-IR using a Mettler Toledo *ReactIR*® *15* equipped with a 6.3 mm probe. The relative absorption data over time was normalized to yields obtained by calibrated GC-MS.

5.2.2 Premixing of Aryl Iodide, $AgBF_4$ and Pd_2dba_3

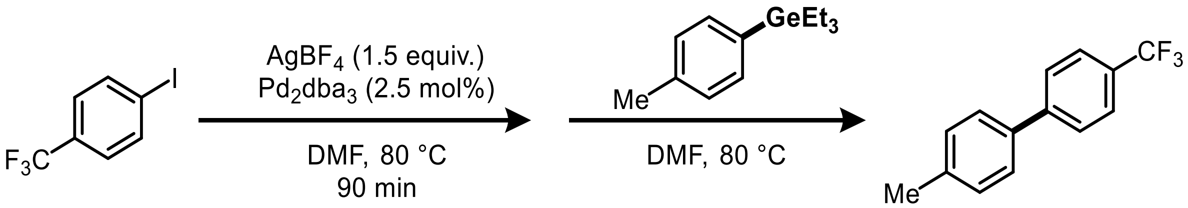

For *in situ* formation of [Pd] nanoparticles, 1-iodo-4-(trifluoromethyl)benzene (22 µL, 0.15 mmol, 1.5 equiv.), $AgBF_4$ (29.2 mg, 0.15 mmol, 1.5 equiv.) and Pd_2dba_3 (2.3 mg, 0.0025 mmol, 2.5 mol%) were dissolved in DMF (1 mL) and stirred for 90 min at 80 °C. Triethyl(4-tolyl)germane (26.7 mg, 0.10 mmol, 1.0 equiv.) was added. The reaction was monitored as previously described.

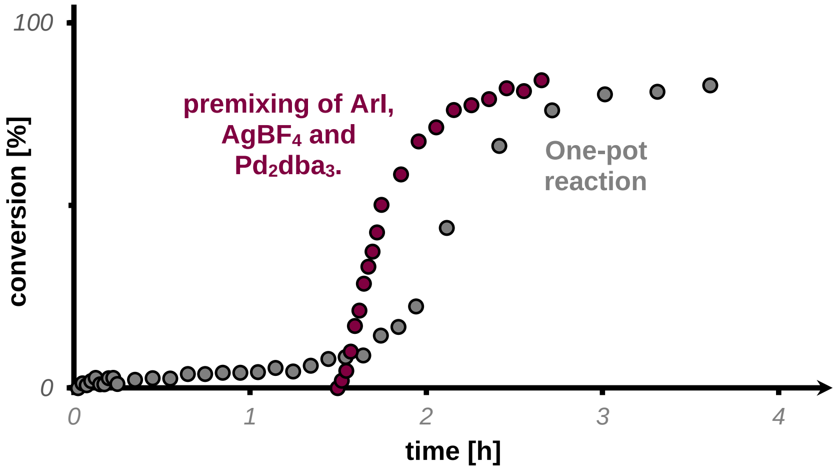

Figure S2 – Kinetic profile of nanoparticle catalyzed cross coupling of aryl germanes with and without premixing.

5.3 Mercury Test

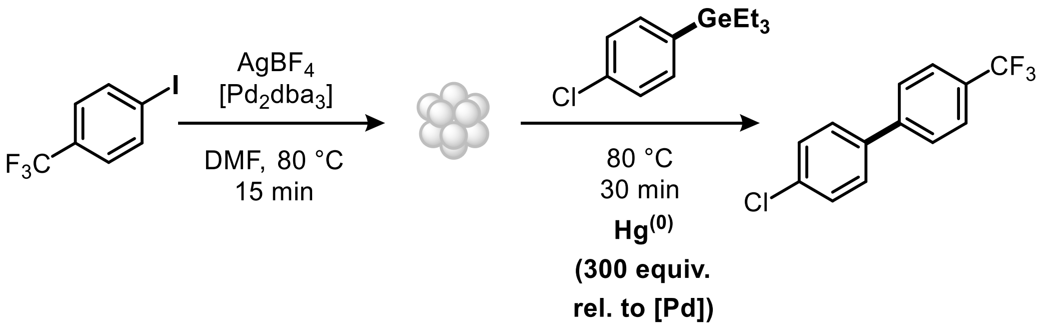

In a septum seal vial, sealed under argon, 4-iodobenzotrifluoride (22.0 µL, 0.15 mmol, 1.5 equiv.) was added to a 4 mL glass vial, to this Pd_2dba_3 (0.46 mg, 0.0005 mmol, 0.5 mol%) and $AgBF_4$ (29.2 mg, 0.15 mmol, 1.5 equiv.) were added followed by 330 µL anhydrous DMF. The reactions were heated at 80 °C for 15 minutes before the addition of triethyl(4-chlorophenyl)germane (27.1 mg, 0.1 mmol, 1.0 equiv.) followed immediately by metallic mercury (60.2 mg, 0.3 mmol, 3.0 equiv.). After 40 minutes, an aliquot was taken, filtered through a short plug of silica and analyzed by GC/MS. The resulting chromatogram showed trace quantities (< 5 %) of the cross coupled product.

5.4 Stability Test

5.4.1 Stability of Triethyl(perfluorophenyl)germane

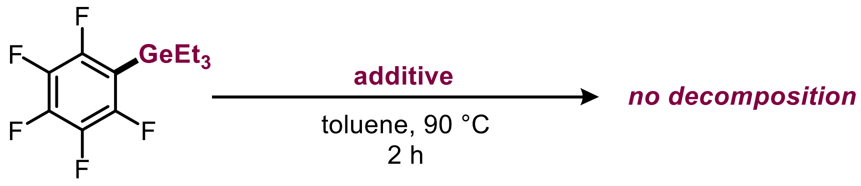

In a septum seal vial, triethyl(perfluorophenyl)germane (16.3 mg, 0.05 mmol, 1.0 equiv.) was added to a 4 mL glass vial, 150 µL toluene-d_8 was added, followed by the corresponding additive (2.0 – 4.0 eq). The reactions were heated at 90 °C for 2 h. The reactions were cooled to room temperature, a further 600 µL toluene-d_8 added and the samples analyzed by quantitative ^{19}F NMR analysis using 1,4-difluorobenzene as an external standard.

Table S7 – Stability test with triethyl(pentafluorophenyl)germane.

entry	Additive	Ar–[M] recovered[a]
**1**	**H_2O** (4 equiv.)	> 99%
**2**	**HCl** (4 M in 1,4-dioxane, 4 equiv.)	> 99%
**3**	**NaOH** (4 equiv.)	> 99%
**4**	**NaO*t*Bu** (4 equiv.)	89%
**5**	**KF** (hydrous, 4 equiv.)	> 99%
**6**	***n*BuLi** (2.5 M in hexane, 2 equiv.)	94%

[a]Determined by quantitative ^{19}F NMR using 1,4-difluorobenzene as internal standard.

5.4.2 Stability of Triethyl(3-fluoropyridyl)germane

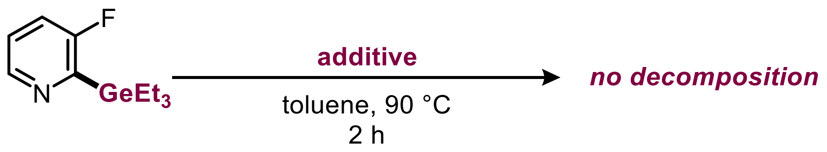

In a septum seal vial, triethyl(3-fluoropyridyl)germane (11.3 mg, 0.05 mmol, 1.0 equiv.) was added to a 4 mL glass vial, 150 µL toluene-d_8 was added, followed by the corresponding additive (2.0 – 4.0 eq). The reactions were heated at 90 °C for 2 h. The reactions were cooled to room temperature, a further 600 µL toluene-d_8 added and the samples analyzed by quantitative ^{19}F NMR analysis using 1,4-difluorobenzene as an external standard.

Table S8 – Stability test with triethyl(pentafluorophenyl)germane.

entry	Additive	Ar–[M] recovered[a]
**1**	**H_2O** (4 equiv.)	> 99%
**2**	**HCl** (4 M in 1,4-dioxane, 4 equiv.)	8%
**3**	**NaOH** (4 equiv.)	> 99%
**4**	**NaO*t*Bu** (4 equiv.)	89%
**5**	**KF** (hydrous, 4 equiv.)	> 99%
**6**	***n*BuLi** (2.5 M in hexane, 2 equiv.)	43%

[a]Determined by quantitative ^{19}F NMR using 1,4-difluorobenzene as internal standard.

5.4.3 Stability of 4-Tolylboronic acid MIDA ester

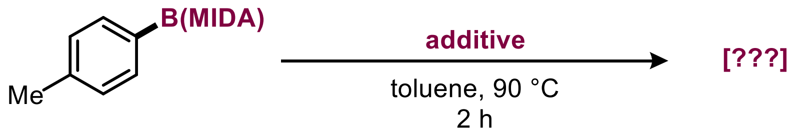

In a septum seal vial, 4-tolylboronic acid MIDA ester (12.4 mg, 0.05 mmol, 1.0 equiv.) was added to a 4 mL glass vial, 150 µL toluene-d_8 was added, followed by the corresponding additive (2.0 – 4.0 eq). The reactions were heated at 90 °C for 2 h. The reactions were cooled to room temperature, 600 µL toluene-d_8/MeCN-d_3 (3:1) added and the samples analyzed by quantitative ^{1}H NMR analysis using mesitylene as an internal standard..

Table S9 – Stability test with 4-tolylboronic acid MIDA ester.

entry	Additive	Ar–[M] recovered[a]
**1**	**H_2O** (4 equiv.)	> 99%
**2**	**HCl** (4 M in 1,4-dioxane, 4 equiv.)	86%
**3**	**NaOH** (4 equiv.)	0%
**4**	**NaO*t*Bu** (4 equiv.)	0%
**5**	**KF** (hydrous, 4 equiv.)	89%
**6**	***n*BuLi** (2.5 M in hexane, 2 equiv.)	0%

[a]Determined by quantitative ^{1}H NMR using mesitylene as internal standard.

5.5 TEM Analysis

Scanning transmission electron microscopy (STEM) was performed on a FEI Tecnai F20. Before measurements, samples were mounted on a 3 mm copper grid and fixed in the grid holder. Images were acquired in bright-field (BF), dark-field (DF) and high-angle annular dark-field (HAADF) imaging. The results are shown in Fig. S3 to Fig S5.

5.5.1 Premixing of Aryl Iodide, $AgBF_4$ and Pd_2dba_3

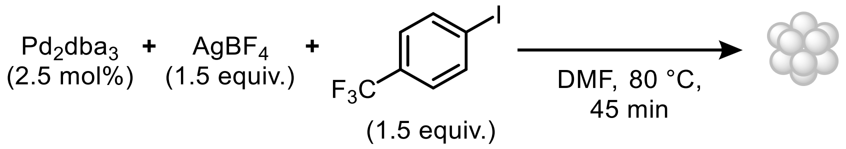

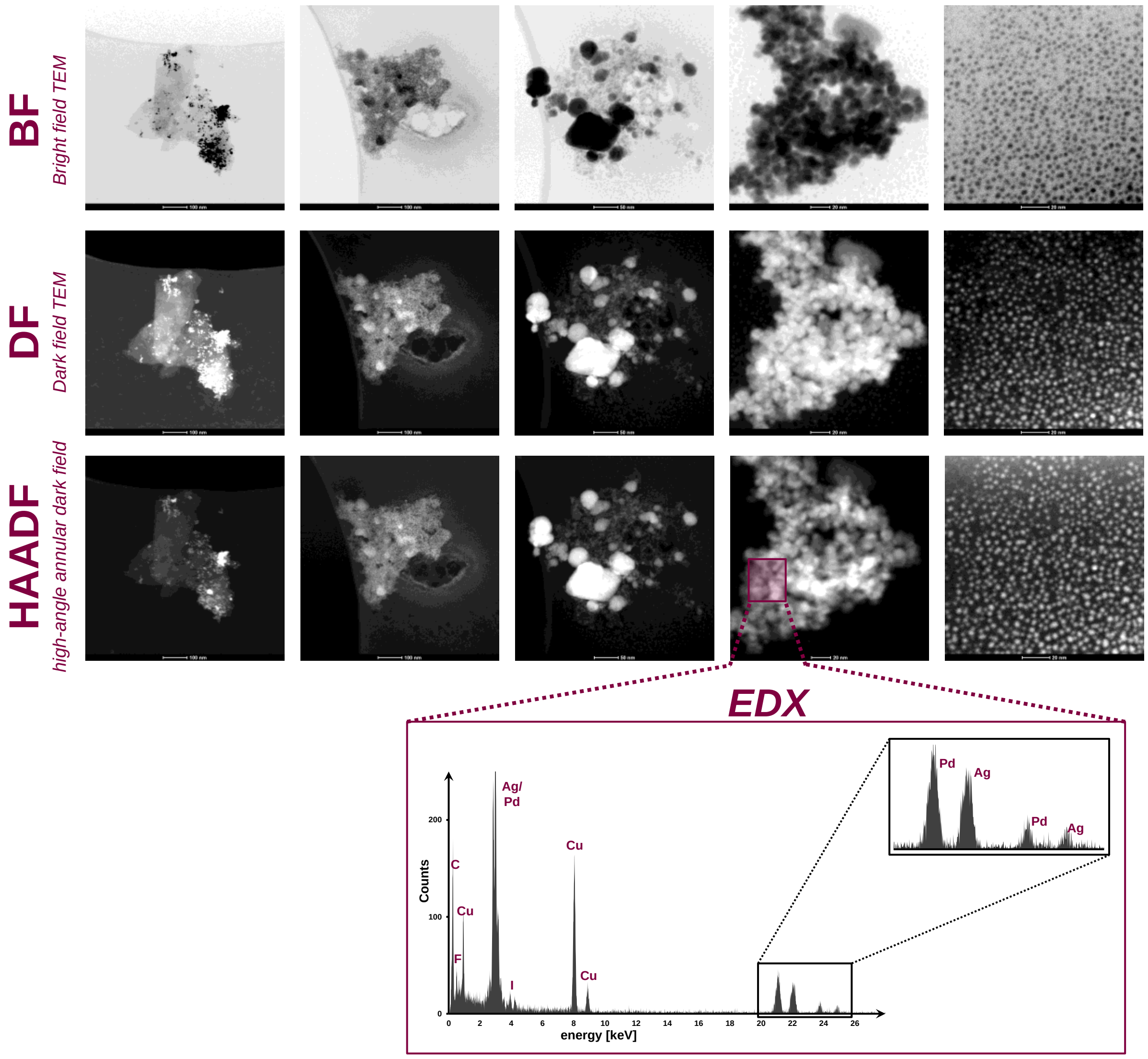

Figure S3 – TEM analysis of formation of nanoparticles. Comparison of bright-field, dark-field and HAADF mode of imaging.

5.5.2 Premixing of Diaryl Iodoniumsalt and Pd_2dba_3 (Silver-Free Conditions)

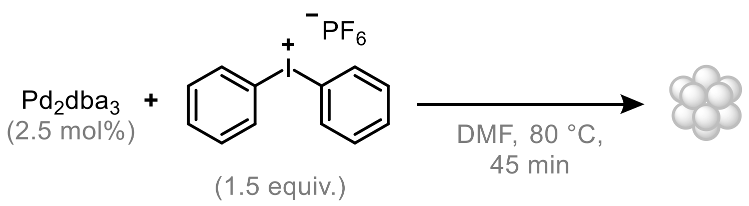

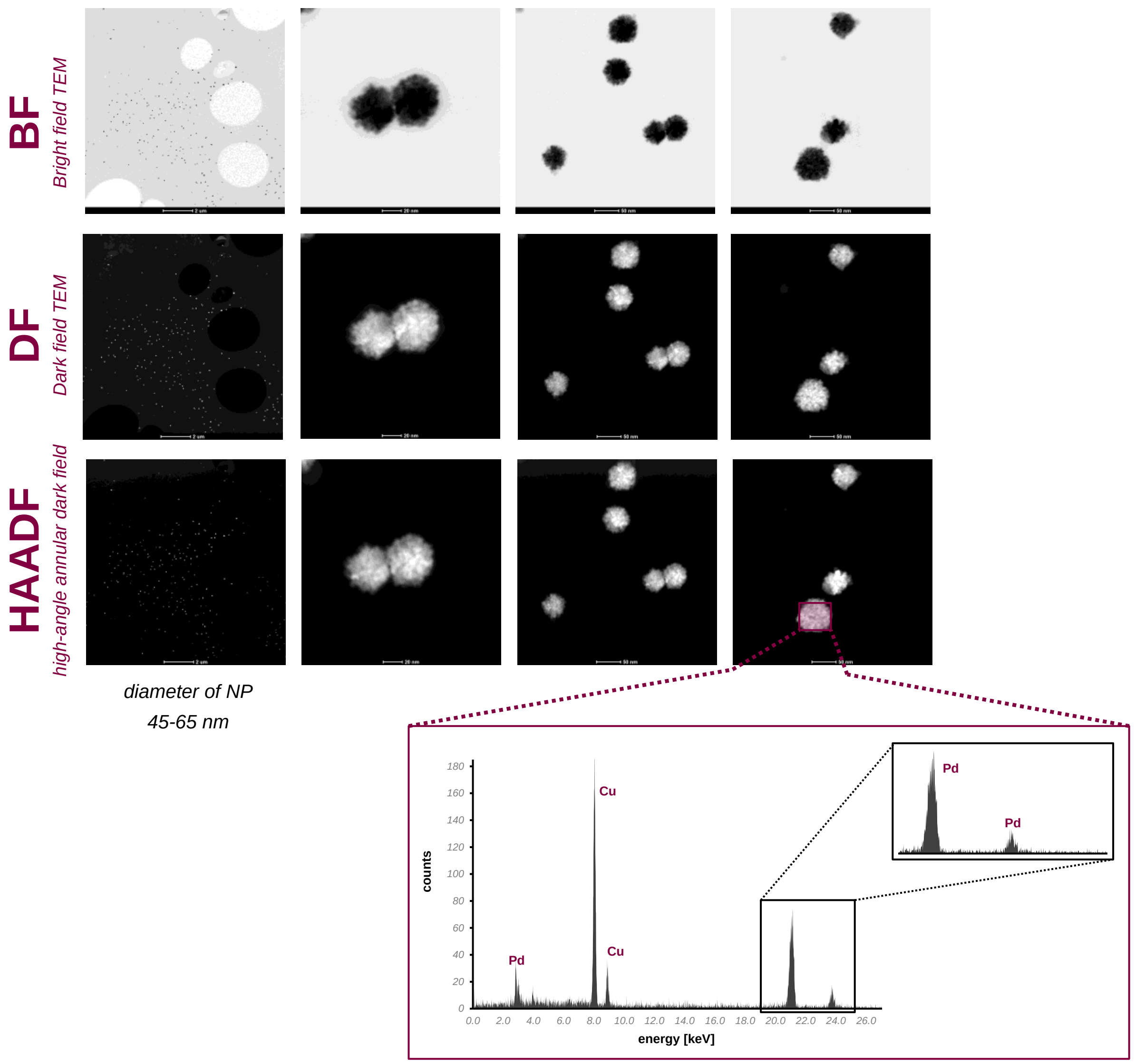

Figure S4 – TEM analysis of formation of palladium nanoparticles under silver-free conditions. Comparison of bright-field, dark-field and HAADF mode of imaging.

5.5.3 Standard Reaction conditions for Catalysis

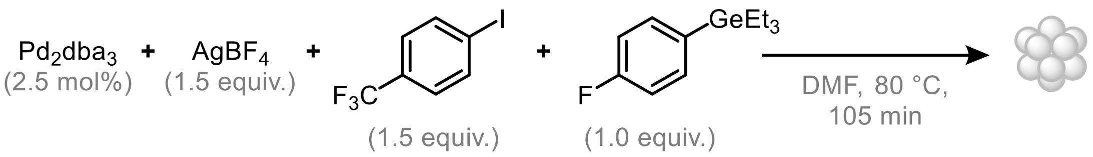

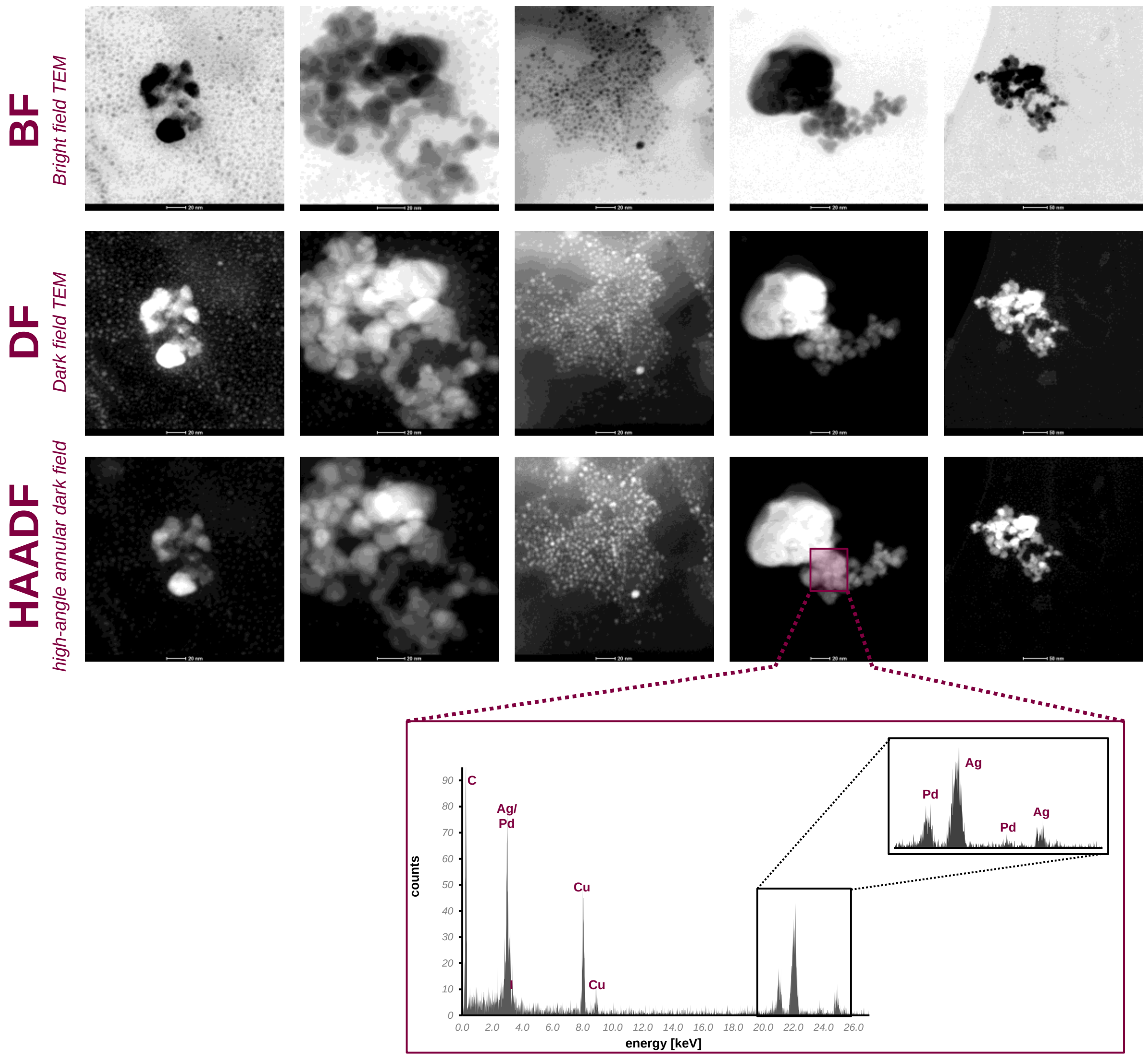

Figure S5 – TEM analysis of in situ formation of nanoparticles in cross coupling reactions with aryl germanes. Comparison of bright-field, dark-field and HAADF mode of imaging.

6 Pd$^{(0)}$/Pd$^{(II)}$ *versus* Nanoparticle Catalysis

6.1 Pd$^{(0)}$/Pd$^{(II)}$ Molecular Catalysis

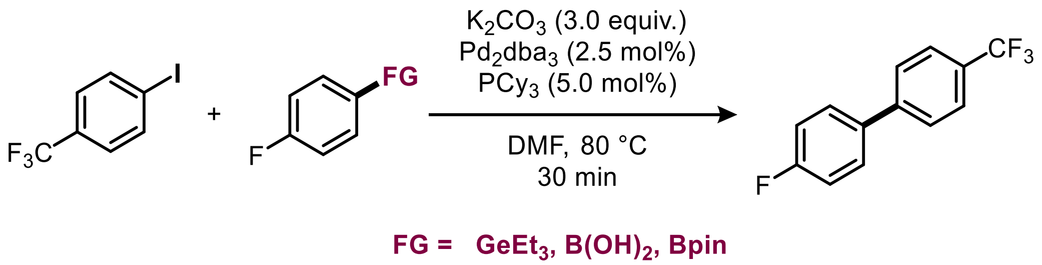

In an inert atmosphere, 4-bromobenzotrifluoride (20.3 mg, 0.09 mmol, 1.5 equiv.) and the desired transmetalating agent (0.06 mmol, 1.0 equiv.) were added to a 4 mL glass vial, to this Pd_2dba_3 (1.3 mg, 0.0015 mmol, 2.5 mol%), PCy_3 and K_2CO_3 (17.5 mg, 0.09 mmol, 1.5 equiv.) were added followed by anhydrous DMF (0.6 mL). The reactions were heated at 80 °C for 12 h. The reactions were cooled to room temperature and 400 µL 1,4-difluorobenzene (stock solution in DMF-d_7, 0.3 M) added as an external standard. The solutions were filtered through a short silica plug and analyzed by quantitative ^{19}F NMR spectroscopy.

Table S10 – Product formation and consumption of starting material on Pd$^{(II)}$ catalysis after 30 min reaction time

entry	FG	Ar–Ar[a]	Ar–[M] recovered[a]
**1**	**$GeEt_3$**	0%	> 99%
**2**	**$B(OH)_2$**	76%	0%
**3**	**B(pin)**	58%	0%

[a]Determined by quantitative ^{19}F NMR.

6.2 Intermolecular Competition – $Pd^{(0)}/Pd^{(II)}$ Molecular Catalysis

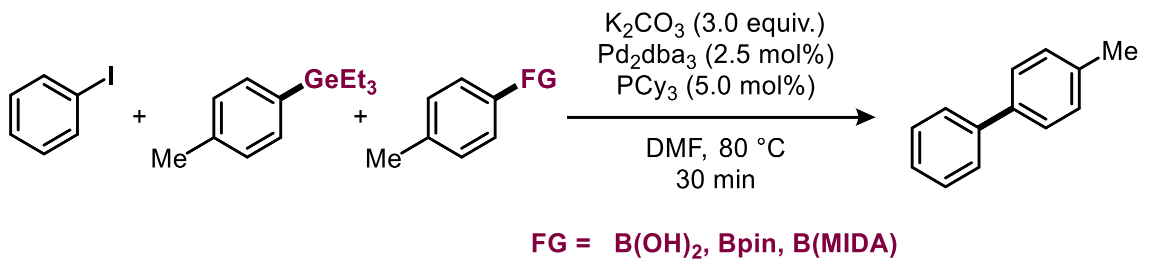

In an inert atmosphere, iodobenzene (3.4 µL, 0.03 mmol, 1.0 equiv.), triethyl(*p*-tolyl)germane (7.5 mg, 0.03 mmol, 1.0 equiv.) **and** the desired transmetalating agent (0.03 mmol, 1.0 equiv.) were added to a 4 mL glass vial, to this a stock solution of Pd_2dba_3 (0.69 mg, 0.00075 mmol, 2.5 mol%) and PCy_3 (0.42 mg, 0.0015 mmol, 5.0 mol%) in DMF (0.3 mL) and K_2CO_3 (20.7 mg, 0.09 mmol, 3.0 equiv.) were added. The reactions were heated at 80 °C for 12 h. The reactions were cooled to room temperature and analyzed by calibrated GC-MS analysis using mesitylene as internal standard.

Table S11 – Product formation and consumption of starting material on $Pd^{(II)}$ catalysis after 30 min reaction time

entry	FG	Ar–Ar[a]	Ar–GeEt3 recovered[a]	Ar–FG recovered[a]
**1**	**B(OH)$_2$**	30%	98%	*n.d.*[b]
**2**	**B(pin)**	26%	>99%	59%
**3**	**B(MIDA)**	0%	>99%	77%[c]

[a]Determined by calibrated GC-MS analysis using mesitylene as internal standard. [b]Not detected by GC-MS. [c]Determined by quantitative ^{1}H NMR using mesitylene as internal standard.

6.3 Nanoparticle Catalysis

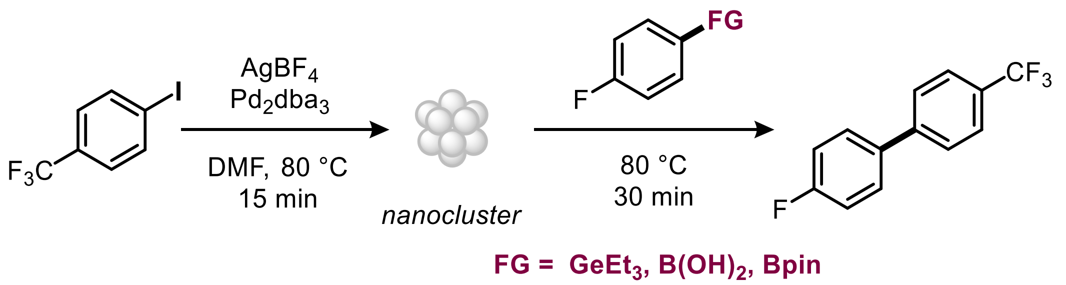

In an inert atmosphere, 4-iodobenzotrifluoride (8.8 µL, 0.06 mmol, 1.5 equiv.) was added to a 4 mL glass vial, to this Pd_2dba_3 (1.3 mg, 0.0015 mmol, 2.5 mol%) and $AgBF_4$ (17.5 mg, 0.09 mmol, 1.5 equiv.) were added followed by 200 µL anhydrous DMF. The reactions were heated at 80 °C for 15 minutes before the addition of the desired transmetalating agent (0.06 mmol, 1.0 equiv.). After 30 minutes further reaction at 80 °C, the reactions were cooled to room temperature and 400 µL 1,4-difluorobenzene (stock solution in DMF-d_7, 0.3 M) added as an external standard. The solutions were filtered through a short silica plug and analyzed by quantitative ^{19}F NMR spectroscopy.

Table S12 – Product formation and recovery of starting material in nanoparticle catalysis after 30 min reaction time

entry	FG	Ar–Ar[a]	Ar–FG recovered[a]
**1**	**$GeEt_3$**	57%	40%
**2**	**$B(OH)_2$**	8%	42%
**3**	**B(pin)**	2%	84%

[a]Determined by quantitative ^{19}F NMR.

6.4 Intermolecular Competition – Nanoparticle Catalysis

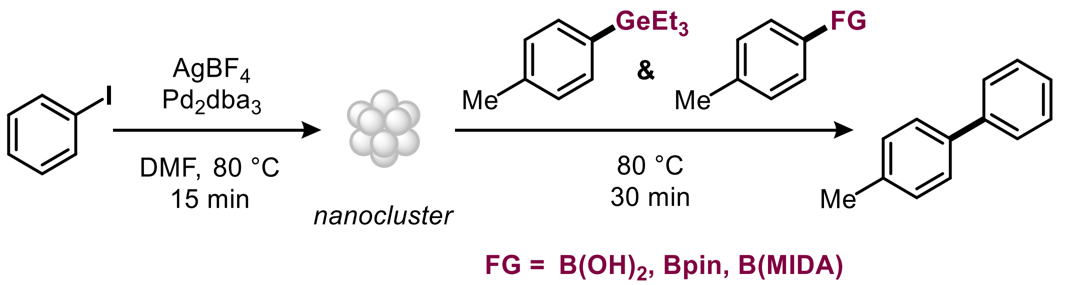

In an inert atmosphere, iodobenzene (3.4 µL, 0.03 mmol, 1.0 equiv.) was added to a 4 mL glass vial, to this a stock solution of Pd_2dba_3 (0.69 mg, 0.00075 mmol, 2.5 mol in DMF (0.3 mL) and $AgBF_4$ (8.8 mg, 0.045 mmol, 1.5 equiv.) were added. The reactions were heated at 80 °C for 45 minutes before the addition of triethyl(*p*-tolyl)germane (7.5 mg, 0.03 mmol, 1.0 equiv.) **and** the desired transmetalating agent (0.06 mmol, 1.0 equiv.). After 30 minutes further reaction at 80 °C, the reactions were cooled to room temperature and analyzed by calibrated GC-MS analysis using mesitylene as internal standard.

Table S13 – Product formation and consumption of starting material on Pd$^{(II)}$ catalysis after 30 min reaction time

entry	FG	Ar–Ar[a]	Ar–GeEt3 recovered[a]	Ar–FG recovered[a]
**1**	**B(OH)$_2$**	65%	71%	*n.d.*[b]
**2**	**B(pin)**	61%	12%	95%
**3**	**B(MIDA)**	60%	3%	96%[c]

[a]Determined by calibrated GC-MS analysis using mesitylene as internal standard. [b]Not detected by GC-MS. [c]Determined by quantitative ^{1}H NMR using mesitylene as internal standard.

6.5 Compatibility of $ArGeEt_3$ to Orthogonal Coupling Reaction with ArB(MIDA)

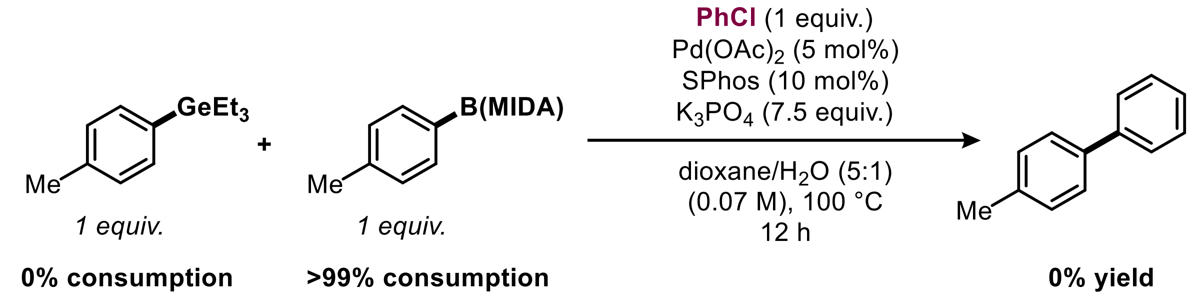

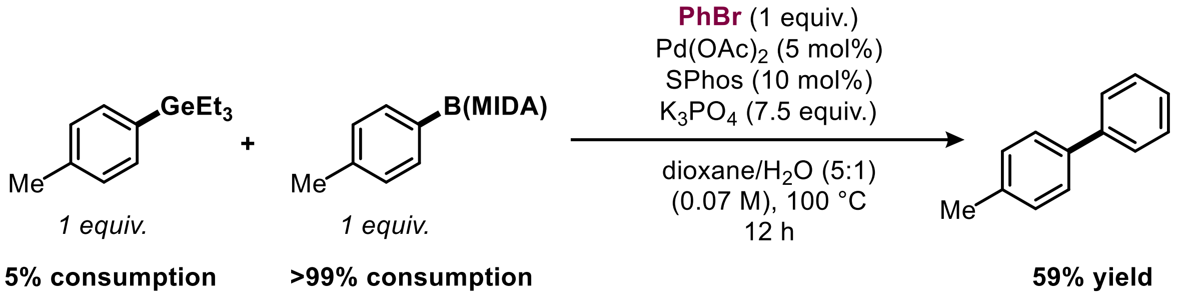

In an inert atmosphere, chloro- or bromobenzene (0.05 mmol, 1.0 equiv.), triethyl(*p*-tolyl)germane (12.5 mg, 0.05 mmol, 1.0 equiv.) **and** 4-tolylboronic acid MIDA ester (12.4 mg, 0.05 mmol, 1.0 equiv.) were added to a 4 mL glass vial, to this a stock solution of $Pd(OAc)_2$ (0.0025 mmol, 5 mol%) and SPhos (0.005 mmol, 1.0 mol%) in dioxane (0.58 mL) and K_3PO_4 (79.6 mg, 0.375 mmol, 7.5 equiv.) were added. The reactions were heated at 80 °C for 12 h. The reactions were cooled to room temperature and analyzed by calibrated GC-MS analysis using mesitylene as internal standard.

7 Computational Details

7.1 General Computational Details

DFT calculations were carried out in Gaussian 16 (A.03) program package.[36] Geometry optimizations and frequency analyses were calculated with B3LYP-D3/Def2SVP (and the associated pseudopotential for Pd and I) theoretical method, including solvation through the CPCM implicit solvent model (solvent = N,N-dimethylformamide). Frequencies were used to characterize the nature of the stationary point as minima (no imaginary frequency) or transition state (one imaginary frequency). Additionally, relaxation of transition states towards reactants and products and IRC analysis were used to verify the connectivity of the transition states. Electronic energies were further refined using the same functional with a bigger basis set (Def2TZVPP and the corresponding pseudopotential for Pd and I). All the energies showed in the manuscript are calculated at the sum of the high-basis set electronic energies plus the thermochemistry corrections at standard conditions (298.15K and 1 atm). All 3D representations were created using the CYLview software.[37]

7.2 Oxidative Saturation of Pd_3 Nanocluster with Aryl Iodide

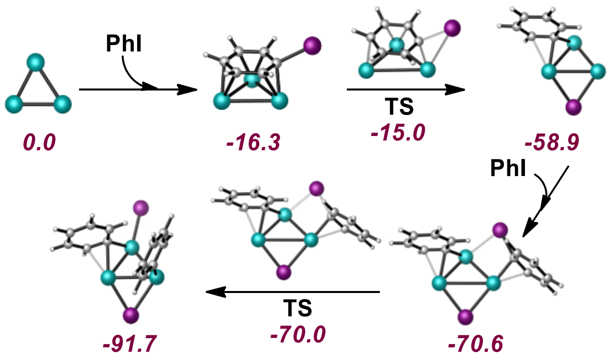

Figure S6 – Oxidative saturation of Pd_3 nanocluster with PhI. Energies in kcal mol^{-1}.

7.3 Homocoupling of Aryl Iodide *vs.* Organogermane S_EAr

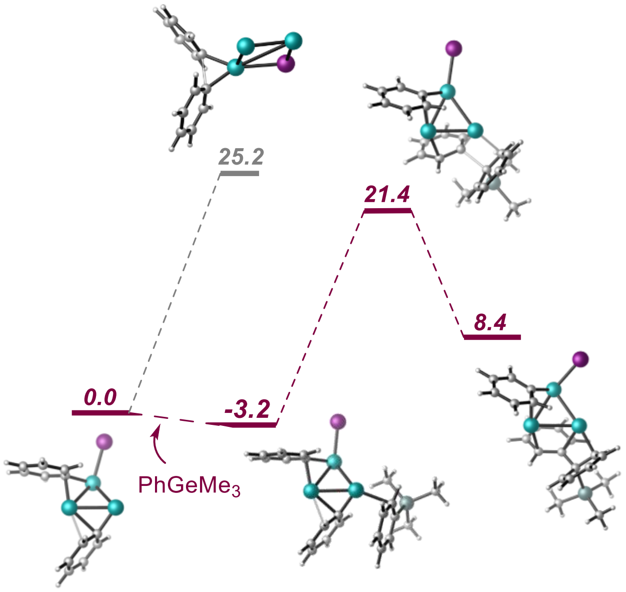

Figure S7 – Free energy profile for the competition between intramolecular reductive elimination and electrophilic aromatic substitution of organogermane. Energies in kcal mol^{-1}.

7.4 Bond Activation of $ArGeEt_3$ with BF_4^- Counterion

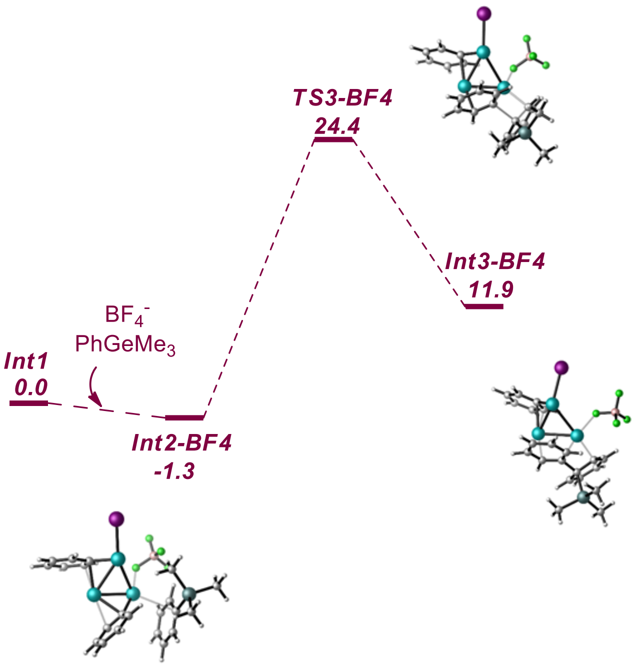

Figure S8 – Free energy profile of the C-C bond formation on Pd_3 nanocluster assisted by BF_4^- as the ion pair. Energies in kcal mol^{-1}.

7.5 XYZ Coordinates and Energies for Optimized Structures

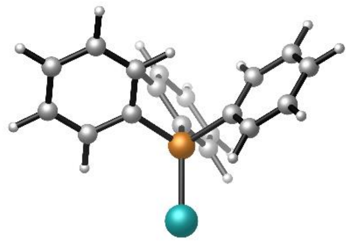

Pd	2.05358500	-0.45880800	-0.22845300
P	4.23483600	-0.52669100	-0.08861900
C	5.10444400	1.11276300	-0.09268800
C	6.22719200	1.39212100	0.70387400
C	4.61816600	2.11079700	-0.95614000
C	6.85062100	2.64358100	0.63507100
H	6.62262600	0.63256000	1.38122300
C	5.24822800	3.35520500	-1.03303100
H	3.73315400	1.90724600	-1.56663000
C	6.36609900	3.62597500	-0.23418100
H	7.72114500	2.84888500	1.26354900
H	4.86062900	4.11939400	-1.71170200
H	6.85459600	4.60231500	-0.28632600
C	4.92958500	-1.33274200	1.43168200
C	6.06022300	-2.16601500	1.41536400
C	4.29370100	-1.07371400	2.65925500
C	6.54459500	-2.72590300	2.60351700
H	6.57056600	-2.38108400	0.47443600
C	4.78522200	-1.62265500	3.84599000
H	3.40173400	-0.44018700	2.67837500
C	5.91193500	-2.45404900	3.82050100
H	7.42297000	-3.37599900	2.57559000
H	4.28240200	-1.40879400	4.79269400
H	6.29202600	-2.89209400	4.74691000
C	5.11218200	-1.43674000	-1.44739800
C	6.32175300	-0.99977400	-2.01214700
C	4.53781500	-2.63330000	-1.91280400
C	6.94355200	-1.74644800	-3.01984700
H	6.78650100	-0.07434000	-1.66638400
C	5.16532000	-3.38414400	-2.90973500
H	3.58658300	-2.97155500	-1.49075700
C	6.37024900	-2.94023000	-3.46865500
H	7.88251200	-1.39229700	-3.45331500
H	4.70849500	-4.31399700	-3.25832300
H	6.85766300	-3.52170600	-4.25535900

Zero-point correction = 0.274798 (Hartree/Particle)

Thermal correction to Energy = 0.292613

Thermal correction to Enthalpy = 0.293557

Thermal correction to Gibbs Free Energy = 0.224932

Sum of electronic and zero-point Energies = -1163.441676

Sum of electronic and thermal Energies = -1163.423861

Sum of electronic and thermal Enthalpies = -1163.422917

Sum of electronic and thermal Free Energies = -1163.491542

E(B3LYPD3/Def2TZVPP) = -1164.624703

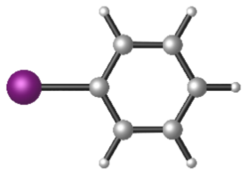

C	1.51886400	2.55626300	-0.06713200
C	2.91875900	2.56524300	-0.06672300
C	3.62048200	3.77519100	-0.06731700
C	2.91888100	4.98523600	-0.06828100
C	1.51901300	4.99438300	-0.06868600
C	0.83221000	3.77534900	-0.06811500
H	0.97672100	1.60901400	-0.06668000
H	3.45899700	1.61512400	-0.06595900
H	4.71296900	3.77515300	-0.06699900
H	3.45926500	5.93527400	-0.06873500
H	0.97694000	5.94167200	-0.06944300
I	-1.30312300	3.77550700	-0.06871600

Zero-point correction = 0.090041 (Hartree/Particle)

Thermal correction to Energy = 0.095902

Thermal correction to Enthalpy = 0.096846

Thermal correction to Gibbs Free Energy = 0.058298

Sum of electronic and zero-point Energies = -529.192812

Sum of electronic and thermal Energies = -529.186951

Sum of electronic and thermal Enthalpies = -529.186007

Sum of electronic and thermal Free Energies = -529.224555

E(B3LYPD3/Def2TZVPP) = -529.5465435

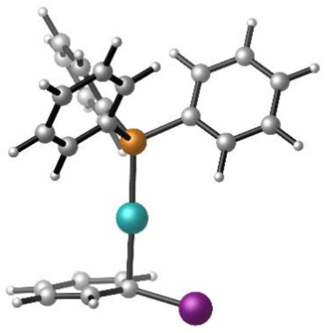

C	-4.36395200	-1.12236600	1.01999600
C	-4.71729700	0.03024600	1.73670400
C	-4.64902900	1.29375700	1.13695800
C	-4.18958500	1.42062500	-0.17871400
C	-3.82863200	0.28706600	-0.92388400
C	-3.93811600	-0.99927000	-0.33015700

H	-4.48574800	-2.10853100	1.47104900
H	-5.05933600	-0.07219400	2.76956000
H	-4.94549500	2.18177800	1.69975100
H	-4.11931600	2.40593800	-0.64613800
H	-3.54604100	0.38804800	-1.97305000
I	-3.99641900	-2.76188200	-1.62430200
Pd	-1.86010700	-1.03804300	0.17522300
P	0.35145700	-1.03736300	0.76236100
C	1.47331100	-1.86029400	-0.45689900
C	0.98595600	-2.99844800	-1.12441900
C	2.77586100	-1.41336200	-0.73323900
C	1.79024800	-3.68490000	-2.03765300
H	-0.03421900	-3.34318100	-0.93052200
C	3.57678500	-2.09670600	-1.65531600
H	3.17187900	-0.52928800	-0.22929500
C	3.08820200	-3.23401600	-2.30635000
H	1.39961400	-4.56927100	-2.54743300
H	4.58777600	-1.73743100	-1.86388900
H	3.71537800	-3.76571200	-3.02637900
C	0.74677300	-1.90856500	2.34549100
C	-0.17771000	-1.79812300	3.39951900
C	1.92019500	-2.65514600	2.54271000
C	0.07325200	-2.40870700	4.63103000
H	-1.10332100	-1.23496900	3.24751600
C	2.16555400	-3.27367700	3.77377500
H	2.64873100	-2.75713700	1.73569200
C	1.24594700	-3.14965400	4.82032500
H	-0.65268600	-2.31300100	5.44246900
H	3.08085500	-3.85435600	3.91415600
H	1.43946900	-3.63428800	5.78063200
C	1.12937600	0.62437600	0.99350100
C	2.12520500	0.88415200	1.94950700
C	0.70091800	1.66697100	0.15288800
C	2.68484100	2.16226800	2.05713900
H	2.46883100	0.08960000	2.61514100
C	1.26778000	2.94000700	0.25491100
H	-0.08883400	1.47771700	-0.58027900
C	2.26068500	3.19085500	1.20954200
H	3.45661800	2.35305100	2.80722600
H	0.92713200	3.74077600	-0.40638100
H	2.69877900	4.18839400	1.29607900

Zero-point correction = 0.364889 (Hartree/Particle)

Thermal correction to Energy = 0.390803

Thermal correction to Enthalpy = 0.391747

Thermal correction to Gibbs Free Energy= 0.301201

Sum of electronic and zero-point Energies = -1692.663638

Sum of electronic and thermal Energies = -1692.637725

Sum of electronic and thermal Enthalpies = -1692.636781

Sum of electronic and thermal Free Energies = -1692.727327

E(B3LYPD3/Def2TZVPP) = -1694.196009

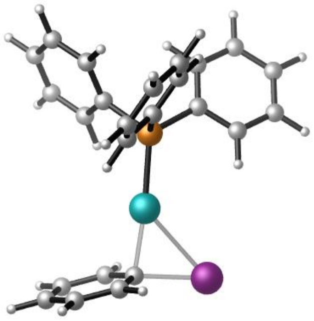

C	-4.41013300	-1.09611300	1.01742400
C	-4.99586500	-0.04741300	1.73692900
C	-5.05332300	1.24330800	1.19697100
C	-4.50965600	1.50007300	-0.06767600
C	-3.91674100	0.47241700	-0.81127600
C	-3.85623900	-0.83092200	-0.25934300
H	-4.40850200	-2.11006100	1.42110600
H	-5.41697800	-0.24951600	2.72519500
H	-5.52415200	2.05078300	1.76246100
H	-4.55073400	2.50753000	-0.48979500
H	-3.53451300	0.66603000	-1.81496700
I	-3.51666200	-2.57574900	-1.66613000
Pd	-1.81400000	-1.00639500	0.14748400
P	0.41514900	-1.04534700	0.73458000
C	1.53496800	-1.89389700	-0.46794900
C	1.02494100	-3.00827400	-1.15756300
C	2.85790500	-1.48804300	-0.70994000
C	1.82739100	-3.71319400	-2.05861300
H	-0.01123100	-3.31811700	-0.99266900
C	3.65697000	-2.18972800	-1.61939600
H	3.27086400	-0.62175400	-0.18887500
C	3.14564800	-3.30429300	-2.29241300
H	1.41925800	-4.57890500	-2.58642800
H	4.68398700	-1.86289500	-1.80128900
H	3.77142200	-3.85046700	-3.00277500
C	0.78077500	-1.90385500	2.33057500
C	-0.14905300	-1.75833000	3.37583000
C	1.93536300	-2.67394900	2.54695200
C	0.07884200	-2.35740500	4.61737300
H	-1.06052200	-1.17617000	3.21016600
C	2.15760300	-3.28051800	3.78835800
H	2.66733000	-2.80370900	1.74713900
C	1.23333300	-3.12141300	4.82593300
H	-0.65077900	-2.23426500	5.42175400
H	3.05862900	-3.87938100	3.94378500
H	1.40893800	-3.59651500	5.79440100
C	1.21010200	0.60919900	0.95377500
C	2.19754600	0.87072300	1.91791700
C	0.80028700	1.64624600	0.09688600

C	2.76776000	2.14482300	2.01740200
H	2.52582800	0.08053400	2.59646800
C	1.37741100	2.91524400	0.19126700
H	0.01738000	1.45694100	-0.64372300
C	2.36223900	3.16761800	1.15384300
H	3.53300200	2.33710400	2.77376300
H	1.05127200	3.71171200	-0.48240200
H	2.80850700	4.16205200	1.23403800

Zero-point correction = 0.364760 (Hartree/Particle)

Thermal correction to Energy = 0.389863

Thermal correction to Enthalpy = 0.390807

Thermal correction to Gibbs Free Energy= 0.302801

Sum of electronic and zero-point Energies = -1692.663204

Sum of electronic and thermal Energies = -1692.638101

Sum of electronic and thermal Enthalpies = -1692.637157

Sum of electronic and thermal Free Energies = -1692.725164

E(B3LYPD3/Def2TZVPP) = -1694.195784

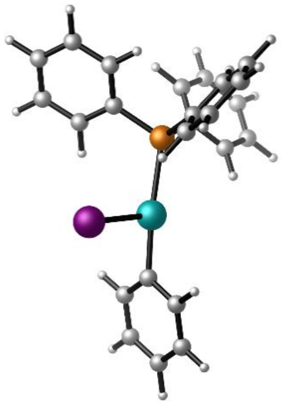

C	-4.43174800	0.33103000	1.47957400
C	-5.64215800	0.99596500	1.23792200
C	-6.14794100	1.08795800	-0.06421400
C	-5.44344300	0.51101400	-1.12761900
C	-4.23236300	-0.15531400	-0.89337100
C	-3.70805200	-0.21473200	0.40756400
H	-4.05586100	0.24869300	2.50377600
H	-6.19514500	1.43473500	2.07348100
H	-7.09696400	1.59819500	-0.24851600
H	-5.84100700	0.57001900	-2.14494100
H	-3.70043700	-0.61894600	-1.72934200
I	-2.66949700	-3.17761000	1.07795200
Pd	-1.80825700	-0.71648100	0.67933600
P	0.59920700	-1.03027200	0.97994500
C	1.48524300	-1.77521000	-0.44958700
C	0.85422700	-2.82620200	-1.13935600
C	2.75669500	-1.34461900	-0.86370700
C	1.49237500	-3.44277300	-2.21890400
H	-0.13837200	-3.16224800	-0.82799300
C	3.38881300	-1.96057900	-1.94890000
H	3.25715200	-0.52718300	-0.34015100
C	2.75985000	-3.01048400	-2.62646100
H	0.99505600	-4.25999800	-2.74731800
H	4.37744800	-1.61812400	-2.26469100
H	3.25562900	-3.48965300	-3.47441200
C	1.10070000	-2.05073900	2.42169900
C	0.31245300	-1.96990000	3.58291700
C	2.23114600	-2.88329500	2.41266600
C	0.65931000	-2.69890600	4.72295100
H	-0.58094400	-1.33963600	3.58980000
C	2.57080100	-3.61914900	3.55304200
H	2.84994300	-2.95993200	1.51604000
C	1.78851100	-3.52643100	4.70895000
H	0.04139100	-2.62836800	5.62149400
H	3.45116800	-4.26646900	3.53695700
H	2.05567400	-4.10275200	5.59826000
C	1.44511900	0.58305800	1.24274300
C	2.47066800	0.77131100	2.18320700
C	1.02521400	1.67147800	0.45547400
C	3.06986900	2.02723800	2.32878500
H	2.80560300	-0.06230200	2.80390800
C	1.62990900	2.92261400	0.59926500
H	0.22342100	1.54131300	-0.27792800
C	2.65312100	3.10279900	1.53772200
H	3.86719300	2.16371900	3.06363000
H	1.29798000	3.76026500	-0.01905900
H	3.12286300	4.08264500	1.65396500

Zero-point correction = 0.365650 (Hartree/Particle)

Thermal correction to Energy = 0.391452

Thermal correction to Enthalpy = 0.392396

Thermal correction to Gibbs Free Energy= 0.301994

Sum of electronic and zero-point Energies = -1692.686352

Sum of electronic and thermal Energies = -1692.660549

Sum of electronic and thermal Enthalpies = -1692.659605

Sum of electronic and thermal Free Energies = -1692.750007

E(B3LYPD3/Def2TZVPP) = -1694.219985

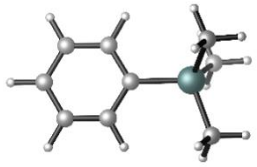

C	-2.30190100	0.38865800	0.36802700
C	-0.90129100	0.37690200	0.38535700
C	-0.15950400	1.51004400	0.00548300
C	-0.86822600	2.65967900	-0.39302100
C	-2.26725200	2.67847500	-0.41258300
C	-2.98815400	1.54037300	-0.03135500
H	-2.85836700	-0.50390800	0.66752400
H	-0.38531000	-0.53447600	0.70184400

H	-0.32532000	3.56167000	-0.69484700
H	-2.79707500	3.58253400	-0.72559000
H	-4.08131700	1.55209600	-0.04557000
Ge	1.81367600	1.51297500	0.01691000
C	2.45944100	-0.21466700	0.70342200
H	2.11895200	-1.04177800	0.06110000
H	3.56034400	-0.22132900	0.72384800
H	2.09101800	-0.39047000	1.72612800
C	2.43456100	2.98958700	1.16533600
H	3.53406100	3.05255800	1.14746900
H	2.02463700	3.94830600	0.81080800
H	2.10733200	2.83289400	2.20502000
C	2.45483700	1.80399300	-1.82435200
H	3.55447000	1.86506500	-1.84268200
H	2.13701400	0.97637000	-2.47753900
H	2.04592600	2.74383900	-2.22715900

Zero-point correction = 0.199077 (Hartree/Particle)

Thermal correction to Energy = 0.212031

Thermal correction to Enthalpy = 0.212975

Thermal correction to Gibbs Free Energy= 0.159003

Sum of electronic and zero-point Energies = -2427.766028

Sum of electronic and thermal Energies = -2427.753074

Sum of electronic and thermal Enthalpies = -2427.752130

Sum of electronic and thermal Free Energies = -2427.806103

Sum of electronic and thermal Free Energies = -1692.750007

E(B3LYPD3/Def2TZVPP) = -2428.621333

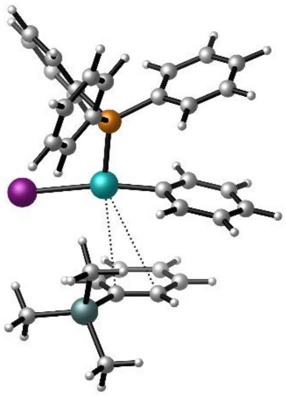

C	-4.56503600	-0.24834200	-0.01525700
C	-5.90277100	-0.25014300	-0.43136300
C	-6.30408900	-1.04169100	-1.51418600
C	-5.35514400	-1.82226600	-2.18242300
C	-4.01746400	-1.82428000	-1.76206300
C	-3.61719000	-1.05646700	-0.65970700
H	-4.27246900	0.38392900	0.82584700
H	-6.63262900	0.37281700	0.09436200
H	-7.34753400	-1.04180200	-1.84043100
H	-5.65287600	-2.43537400	-3.03831800
H	-3.29125800	-2.44062900	-2.29812000
P	0.63873000	-1.27495900	0.78808600
C	1.77051500	-1.33464400	-0.66387300
C	1.36143600	-2.10988600	-1.76456600
C	2.99309200	-0.64897800	-0.72864400
C	2.17284500	-2.21867700	-2.89642200
H	0.39668000	-2.62333300	-1.74188300
C	3.79698700	-0.74761600	-1.86954700
H	3.32324800	-0.02564500	0.10349400
C	3.39294400	-1.53537900	-2.95196000
H	1.84495900	-2.82883800	-3.74155400
H	4.74402500	-0.20373000	-1.91020400
H	4.02407300	-1.61129300	-3.84088800
C	1.13464700	-2.74515300	1.79501100
C	0.86093300	-2.75339600	3.17654600
C	1.66118900	-3.90543900	1.20331800
C	1.10555000	-3.89452000	3.94394900
H	0.46021100	-1.86111800	3.66360400
C	1.90570100	-5.04679900	1.97492600
H	1.88718500	-3.92615600	0.13553400
C	1.62687800	-5.04679800	3.34481100
H	0.88799100	-3.88095800	5.01476100
H	2.31899700	-5.93942700	1.49888100
H	1.81789100	-5.93972900	3.94489700
C	1.19764700	0.12856500	1.83796500
C	2.41672600	0.08354800	2.54164100
C	0.37733400	1.26108600	1.96597300
C	2.81104000	1.15747100	3.34251500
H	3.05632000	-0.79933800	2.47524400
C	0.77349700	2.33412600	2.77141200
H	-0.57468300	1.30452200	1.43607300
C	1.99007800	2.28601100	3.45785800
H	3.76013700	1.11110000	3.88209400
H	0.12353600	3.20753000	2.86485400
H	2.29755500	3.12388800	4.08860600
C	-4.41627900	-4.23437900	0.37450000
C	-4.68552400	-3.36482100	1.44489800
C	-3.65090900	-2.72364000	2.13083800
C	-2.32018400	-2.97269000	1.70975500
C	-2.04678700	-3.83733900	0.62806500
C	-3.10628700	-4.46982700	-0.03939800
H	-5.24509700	-4.72186700	-0.14481500
H	-5.72608300	-3.18754200	1.72950300
H	-1.49561800	-2.62662600	2.33175500
H	-1.01376200	-4.07953400	0.37068400
H	-2.90184800	-5.14218000	-0.87538400
Ge	-3.96263400	-1.50051900	3.64699000
C	-5.83487000	-0.90280900	3.60185600
H	-6.06407100	-0.43436300	2.63315900
H	-6.51607100	-1.75536100	3.74904000
H	-6.01725000	-0.17044400	4.40334700

C	-2.71161100	0.01280200	3.49815100
H	-2.89193500	0.73399800	4.31033400
H	-1.67165700	-0.33757800	3.57397400
H	-2.82510500	0.52819800	2.53372000
C	-3.57522300	-2.48293900	5.31123500
H	-3.69669800	-1.82287300	6.18450200
H	-4.25492000	-3.34214400	5.42061300
H	-2.53857900	-2.85467400	5.29373100
Pd	-1.73162100	-1.19193600	0.04141100
I	-1.24358700	0.88579100	-1.57852900

Zero-point correction= 0.566733 (Hartree/Particle)

Thermal correction to Energy= 0.606970

Thermal correction to Enthalpy= 0.607915

Thermal correction to Gibbs Free Energy= 0.486115

Sum of electronic and zero-point Energies= -4120.488639

Sum of electronic and thermal Energies= -4120.448402

Sum of electronic and thermal Enthalpies= -4120.447458

Sum of electronic and thermal Free Energies= -4120.569257

E(B3LYPD3/Def2TZVPP) = -4122.877046

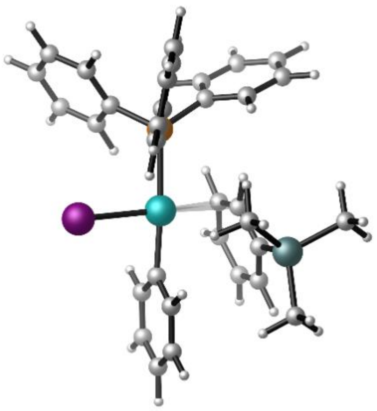

C	-4.56503600	-0.24834200	-0.01525700
C	-5.90277100	-0.25014300	-0.43136300
C	-6.30408900	-1.04169100	-1.51418600
C	-5.35514400	-1.82226600	-2.18242300
C	-4.01746400	-1.82428000	-1.76206300
C	-3.61719000	-1.05646700	-0.65970700
H	-4.27246900	0.38392900	0.82584700
H	-6.63262900	0.37281700	0.09436200
H	-7.34753400	-1.04180200	-1.84043100
H	-5.65287600	-2.43537400	-3.03831800
H	-3.29125800	-2.44062900	-2.29812000
P	0.63873000	-1.27495900	0.78808600
C	1.77051500	-1.33464400	-0.66387300
C	1.36143600	-2.10988600	-1.76456600
C	2.99309200	-0.64897800	-0.72864400
C	2.17284500	-2.21867700	-2.89642200
H	0.39668000	-2.62333300	-1.74188300
C	3.79698700	-0.74761600	-1.86954700
H	3.32324800	-0.02564500	0.10349400
C	3.39294400	-1.53537900	-2.95196000
H	1.84495900	-2.82883800	-3.74155400
H	4.74402500	-0.20373000	-1.91020400
H	4.02407300	-1.61129300	-3.84088800
C	1.13464700	-2.74515300	1.79501100
C	0.86093300	-2.75339600	3.17654600
C	1.66118900	-3.90543900	1.20331800
C	1.10555000	-3.89452000	3.94394900
H	0.46021100	-1.86111800	3.66360400
C	1.90570100	-5.04679900	1.97492600
H	1.88718500	-3.92615600	0.13553400
C	1.62687800	-5.04679800	3.34481100
H	0.88799100	-3.88095800	5.01476100
H	2.31899700	-5.93942700	1.49888100
H	1.81789100	-5.93972900	3.94489700
C	1.19764700	0.12856500	1.83796500
C	2.41672600	0.08354800	2.54164100
C	0.37733400	1.26108600	1.96597300
C	2.81104000	1.15747100	3.34251500
H	3.05632000	-0.79933800	2.47524400
C	0.77349700	2.33412600	2.77141200
H	-0.57468300	1.30452200	1.43607300
C	1.99007800	2.28601100	3.45785800
H	3.76013700	1.11110000	3.88209400
H	0.12353600	3.20753000	2.86485400
H	2.29755500	3.12388800	4.08860600
C	-4.41627900	-4.23437900	0.37450000
C	-4.68552400	-3.36482100	1.44489800
C	-3.65090900	-2.72364000	2.13083800
C	-2.32018400	-2.97269000	1.70975500
C	-2.04678700	-3.83733900	0.62806500
C	-3.10628700	-4.46982700	-0.03939800
H	-5.24509700	-4.72186700	-0.14481500
H	-5.72608300	-3.18754200	1.72950300
H	-1.49561800	-2.62662600	2.33175500
H	-1.01376200	-4.07953400	0.37068400
H	-2.90184800	-5.14218000	-0.87538400
Ge	-3.96263400	-1.50051900	3.64699000
C	-5.83487000	-0.90280900	3.60185600
H	-6.06407100	-0.43436300	2.63315900
H	-6.51607100	-1.75536100	3.74904000
H	-6.01725000	-0.17044400	4.40334700
C	-2.71161100	0.01280200	3.49815100
H	-2.89193500	0.73399800	4.31033400
H	-1.67165700	-0.33757800	3.57397400
H	-2.82510500	0.52819800	2.53372000
C	-3.57522300	-2.48293900	5.31123500
H	-3.69669800	-1.82287300	6.18450200
H	-4.25492000	-3.34214400	5.42061300

H	-2.53857900	-2.85467400	5.29373100
Pd	-1.73162100	-1.19193600	0.04141100
I	-1.24358700	0.88579100	-1.57852900

Zero-point correction = 0.567284 (Hartree/Particle)

Thermal correction to Energy = 0.607220

Thermal correction to Enthalpy = 0.608164

Thermal correction to Gibbs Free Energy= 0.490055

Sum of electronic and zero-point Energies = -4120.481017

Sum of electronic and thermal Energies = -4120.441081

Sum of electronic and thermal Enthalpies = -4120.440137

Sum of electronic and thermal Free Energies = -4120.558246

E(B3LYPD3/Def2TZVPP) = -4122.86568

TS1

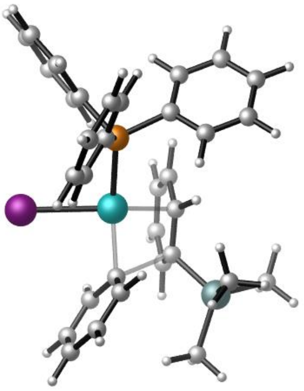

C	-3.82071700	0.15523400	1.23345200
C	-4.68264300	1.21302700	0.91822200
C	-5.57279900	1.09585200	-0.15348200
C	-5.58683200	-0.07788100	-0.92129100
C	-4.71805500	-1.12720100	-0.62002900
C	-3.81483900	-1.01391700	0.44924700
H	-3.14365500	0.25772100	2.08313900
H	-4.66506900	2.12228300	1.52475300
H	-6.26104900	1.91188400	-0.38729400
H	-6.27557300	-0.17165000	-1.76523600
H	-4.72663600	-2.03237400	-1.23038100
P	0.64122300	-1.22778000	0.68426600
C	1.70119800	-1.66486800	-0.75172200
C	1.27113600	-2.73100100	-1.56211300
C	2.89749200	-1.00241400	-1.06562600
C	2.03756000	-3.14132800	-2.65511700
H	0.32688600	-3.23456400	-1.34091100
C	3.65609900	-1.40721900	-2.16945400
H	3.24163200	-0.16405500	-0.45778400
C	3.23165000	-2.47825600	-2.96219600
H	1.69576500	-3.97372400	-3.27510800
H	4.58341000	-0.88094600	-2.40913200
H	3.82742800	-2.79275900	-3.82270600
C	1.24996000	-2.31670300	2.04275500
C	0.76181200	-2.07464600	3.34093300
C	2.10411000	-3.40933700	1.82760900
C	1.12007900	-2.90963900	4.40045400
H	0.10344000	-1.22117200	3.52616900
C	2.45895600	-4.24704200	2.89202800
H	2.49665400	-3.61288100	0.82953000
C	1.96808100	-4.00191300	4.17739200
H	0.73452400	-2.70822900	5.40285900
H	3.12561400	-5.09396100	2.71168000
H	2.24598100	-4.65814400	5.00570000
C	1.18673400	0.43418600	1.26368900
C	2.41355000	0.59466000	1.93571700
C	0.36612500	1.55600800	1.06072100
C	2.81688400	1.85638800	2.37909000
H	3.05433500	-0.26993300	2.12273200
C	0.77184600	2.81720300	1.51021100
H	-0.59050600	1.44496600	0.54776900
C	1.99678000	2.97081900	2.16637200
H	3.77268300	1.96823700	2.89687400
H	0.12330700	3.68175300	1.34832800
H	2.31111800	3.95666200	2.51817100
C	-3.59568500	-4.73361600	-0.09842600
C	-4.26237700	-3.76632300	0.60490500
C	-3.57399400	-2.82224100	1.46524600
C	-2.13020500	-3.00491000	1.60757700
C	-1.47358800	-4.04828700	0.85663300
C	-2.17687700	-4.86727400	0.00552300
H	-4.15457400	-5.43198300	-0.72708100
H	-5.34973100	-3.69345000	0.52068500
H	-1.65007900	-2.68735400	2.53820300
H	-0.40273600	-4.19828000	1.00289400
H	-1.66043500	-5.64476600	-0.56182000
Ge	-4.58810300	-2.42342100	3.15112000
C	-6.38383700	-1.77851500	2.69407800
H	-6.32518500	-0.84458300	2.11777800
H	-6.92553100	-2.53119100	2.10153800
H	-6.95015600	-1.59311000	3.62003400
C	-3.59922300	-1.17319000	4.31022500
H	-3.71276200	-1.49980600	5.35517300
H	-2.52521500	-1.16209900	4.07067300
H	-3.99110900	-0.15075900	4.21822700
C	-4.68376600	-4.17723800	4.04467000
H	-5.23558200	-4.08770800	4.99362500
H	-5.19672800	-4.90871900	3.40211500
H	-3.66975600	-4.54784900	4.26138700
Pd	-1.70611400	-1.37363700	0.28747100
I	-1.49710500	0.16145600	-2.08894000

Zero-point correction = 0.566266 (Hartree/Particle)

Thermal correction to Energy = 0.605393

Thermal correction to Enthalpy = 0.606337

Thermal correction to Gibbs Free Energy= 0.490017

Sum of electronic and zero-point Energies = -4120.440332
Sum of electronic and thermal Energies = -4120.401206
Sum of electronic and thermal Enthalpies = -4120.400261
Sum of electronic and thermal Free Energies = -4120.516581
E(B3LYPD3/Def2TZVPP) = -4122.823923

TS2

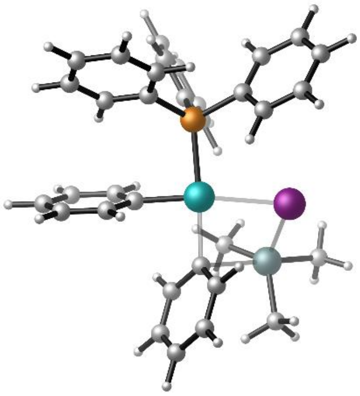

C	-3.43447800	0.65565500	0.44133300
C	-3.56420100	1.81874700	-0.32842700
C	-3.17024500	1.82703100	-1.67158100
C	-2.65155600	0.65739600	-2.23827400
C	-2.52220100	-0.50586400	-1.46652600
C	-2.90563900	-0.52195200	-0.11363000
H	-3.74772400	0.67589500	1.48884500
H	-3.97258800	2.72590100	0.12715400
H	-3.26786000	2.73603400	-2.27106000
H	-2.33844200	0.64806300	-3.28668000
H	-2.09481000	-1.39895200	-1.93046000
I	-2.61928700	-4.74712600	2.10715400
Pd	-2.65533300	-2.18368900	0.98841700
P	-0.36462200	-1.61544600	1.27076700
C	0.61157100	-2.03291300	-0.23122200
C	0.15676700	-3.08509400	-1.04534000
C	1.79920800	-1.36486200	-0.57391700
C	0.87710600	-3.46270200	-2.18165800
H	-0.76940900	-3.60640800	-0.79341000
C	2.51563300	-1.74189900	-1.71393400
H	2.16682700	-0.54555800	0.04714000
C	2.05646600	-2.78984200	-2.51954000
H	0.51053400	-4.27976200	-2.80781400
H	3.43673800	-1.21397900	-1.97294100
H	2.61664900	-3.07997400	-3.41190900
C	0.44619500	-2.55402700	2.63266800
C	-0.24723500	-2.67029400	3.85165800
C	1.70394200	-3.16248100	2.49985300
C	0.31360800	-3.37099600	4.92204300
H	-1.23584900	-2.21607900	3.96072600
C	2.25812200	-3.87335400	3.57008500
H	2.25548400	-3.08790600	1.56084500
C	1.56745100	-3.97724800	4.78161600
H	-0.23414400	-3.45140400	5.86407500
H	3.23575200	-4.34778300	3.45400800
H	2.00316500	-4.53384500	5.61501000
C	0.06771300	0.14270000	1.62429600
C	0.51956600	0.55863000	2.88798400
C	-0.11456800	1.10703900	0.61483400
C	0.78185400	1.91046000	3.13718000
H	0.67526900	-0.16791100	3.68686400
C	0.15698800	2.45334600	0.86586800
H	-0.46983400	0.81008000	-0.37261400
C	0.60272500	2.86116900	2.12827200
H	1.13387300	2.21696000	4.12540200
H	0.01085800	3.18745400	0.06970300
H	0.81076000	3.91612800	2.32369900
C	-6.82210700	-1.91384400	-0.38276000
C	-5.45572600	-2.20901200	-0.41982100
C	-4.72111200	-2.56693400	0.73579600
C	-5.44602700	-2.52344000	1.95324200
C	-6.80855600	-2.20494900	2.01165000
C	-7.50736900	-1.90262700	0.83881700
H	-7.34955700	-1.66223400	-1.30789000
H	-4.94661800	-2.14213000	-1.38307400
H	-4.93881700	-2.76108100	2.89408200
H	-7.32400000	-2.19195400	2.97668100
H	-8.57197000	-1.65561400	0.87502600
Ge	-4.18318200	-5.09562400	0.27893300
C	-6.08354000	-5.29950800	0.76590200
H	-6.71575500	-4.48561800	0.39798300
H	-6.41019800	-6.25663400	0.33188300
H	-6.16683500	-5.35526600	1.85988500
C	-3.75743900	-7.05787700	0.03685800
H	-4.33857400	-7.44064000	-0.82020500
H	-2.68703700	-7.21881400	-0.16864200
H	-4.03618100	-7.64005700	0.93053700
C	-3.56006000	-4.47021900	-1.49023200
H	-4.39593800	-4.58179200	-2.19875500
H	-3.21094800	-3.43345100	-1.50009200
H	-2.74324300	-5.13962200	-1.79742000

Zero-point correction = 0.567662 (Hartree/Particle)
Thermal correction to Energy = 0.605111
Thermal correction to Enthalpy = 0.606056
Thermal correction to Gibbs Free Energy= 0.495805
Sum of electronic and zero-point Energies = -4120.421347
Sum of electronic and thermal Energies = -4120.383898
Sum of electronic and thermal Enthalpies = -4120.382954
Sum of electronic and thermal Free Energies = -4120.493205
E(B3LYPD3/Def2TZVPP) = -4122.810000

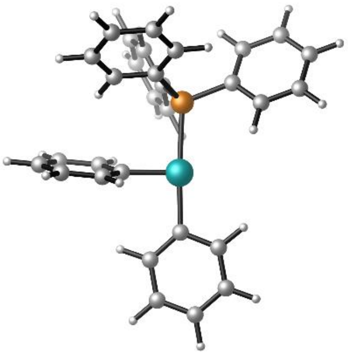

C	-4.89122200	-1.31876400	-0.99308700
C	-6.00293400	-1.17544700	-1.83420900
C	-5.83126400	-0.99312600	-3.21159200
C	-4.53597900	-0.95407800	-3.73948800
C	-3.42812500	-1.09593100	-2.89145400
C	-3.57590400	-1.27940100	-1.49817700
H	-5.05595100	-1.46534200	0.07740200
H	-7.01194500	-1.20620800	-1.41077100
H	-6.69903300	-0.88336900	-3.86799600
H	-4.38536000	-0.81468200	-4.81453300
H	-2.42737100	-1.06017500	-3.34194800
Pd	-1.78691600	-1.37190000	-0.52227900
P	0.38886600	-1.41636000	0.52440400
C	1.76846100	-1.06071000	-0.64166900
C	1.61549000	-1.43846900	-1.98745000
C	2.96574700	-0.44927100	-0.23218000
C	2.64606800	-1.21567100	-2.90627600
H	0.68983500	-1.91531000	-2.32181800
C	3.99170100	-0.22309100	-1.15385400
H	3.09969300	-0.14707800	0.80866600
C	3.83472500	-0.60655700	-2.49095300
H	2.51722500	-1.51548200	-3.94913500
H	4.91831800	0.25453600	-0.82583300
H	4.63853800	-0.42785200	-3.20946400
C	0.86672100	-3.00548800	1.32193100
C	0.13874600	-3.44555800	2.44441500
C	1.88234100	-3.82416700	0.80143500
C	0.43200700	-4.67591600	3.03580200
H	-0.65802700	-2.82734100	2.86147300
C	2.16684600	-5.05983300	1.39381600
H	2.45871500	-3.50082500	-0.06761100
C	1.44448800	-5.48835700	2.51143900
H	-0.13987100	-5.00290900	3.90777600
H	2.96060400	-5.68655000	0.97948600
H	1.66921300	-6.45270300	2.97396000
C	0.58054600	-0.15963200	1.85463800
C	1.37195700	-0.36220100	2.99708100
C	-0.09120500	1.06511400	1.69363100
C	1.48813200	0.64460100	3.96123300
H	1.89715600	-1.30860600	3.14057200
C	0.03161500	2.07166100	2.65477800
H	-0.72257000	1.22582700	0.81486500
C	0.81984300	1.86174600	3.79226900
H	2.10429600	0.47561700	4.84784900
H	-0.49686600	3.01852200	2.51969000
H	0.90990900	2.64551500	4.54850800
C	-3.35706800	-1.77682800	3.42318600
C	-2.84489100	-1.25058700	2.22907900
C	-2.66165000	-2.07583600	1.10575500
C	-3.02973700	-3.42956700	1.19482000
C	-3.53891300	-3.95171000	2.39087000
C	-3.70065200	-3.13051300	3.51219800
H	-3.48154700	-1.12101300	4.28997700
H	-2.56535700	-0.19545100	2.19286000
H	-2.91058900	-4.09206100	0.33446800
H	-3.80702900	-5.01100000	2.44453600
H	-4.09486500	-3.54046000	4.44570100

Zero-point correction= 0.455520 (Hartree/Particle)

Thermal correction to Energy= 0.484316

Thermal correction to Enthalpy= 0.485260

Thermal correction to Gibbs Free Energy= 0.391305

Sum of electronic and zero-point Energies= -1626.241369

Sum of electronic and thermal Energies= -1626.212573

Sum of electronic and thermal Enthalpies= -1626.211629

Sum of electronic and thermal Free Energies= -1626.305583

Zero-point correction = 0.384117 (Hartree/Particle)

E(B3LYPD3/Def2TZVPP) = -1628.104423

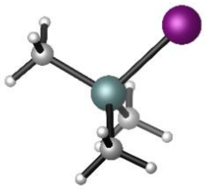

Ge	-0.18045500	-1.02649000	0.10731700
C	0.53509600	-0.01519700	1.62381800
H	0.17047500	1.02172700	1.59829200
H	0.19577000	-0.49260000	2.55741500
H	1.63431300	-0.01619300	1.59974400
C	0.53520900	-2.84544000	-0.01003800
H	0.19782000	-3.41471900	0.87131600
H	0.16886400	-3.34261600	-0.91965600
H	1.63438100	-2.82367500	-0.02372800
C	-2.13382500	-0.95813200	-0.01046300
H	-2.48009400	0.08527500	-0.02333400
H	-2.48018300	-1.46872800	-0.92048400
H	-2.55795600	-1.46674400	0.87050500
I	0.69279400	0.20780900	-2.02980500

Zero-point correction = 0.109521 (Hartree/Particle)

Thermal correction to Energy = 0.119212

Thermal correction to Enthalpy = 0.120157

Thermal correction to Gibbs Free Energy= 0.073159

Sum of electronic and zero-point Energies = -2494.205464

Sum of electronic and thermal Energies = -2494.195772

Sum of electronic and thermal Enthalpies = -2494.194828

Sum of electronic and thermal Free Energies = -2494.241825

E(B3LYPD3/Def2TZVPP) = -2494.731031

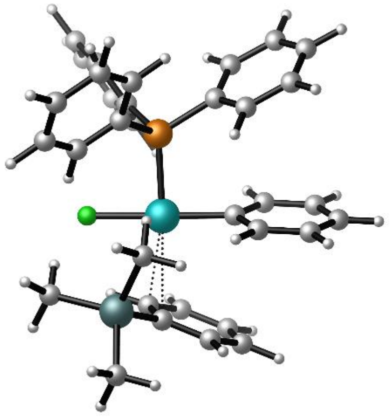

C	-3.14031100	0.48695800	1.89442900
C	-3.64207300	1.75330700	1.56817600
C	-4.03581000	2.03928200	0.25521500
C	-3.92126100	1.04833600	-0.72515000
C	-3.42417700	-0.21918100	-0.39541600
C	-3.03972300	-0.52138400	0.92041200
H	-2.82526500	0.29120100	2.92367300
H	-3.71964000	2.52141600	2.34352700
H	-4.42127400	3.02957400	-0.00202200
H	-4.21768000	1.26052000	-1.75679300
H	-3.33981000	-0.97537300	-1.17744500
Pd	-2.45096800	-2.34526500	1.44481300
P	-0.26553900	-1.73050600	1.27152100
C	0.58736400	-2.73249800	-0.01455700
C	0.25884100	-4.09910400	-0.09305000
C	1.55000500	-2.19730600	-0.88587500
C	0.88186400	-4.91212300	-1.04160700
H	-0.48450500	-4.50212600	0.60039700
C	2.16529200	-3.01911200	-1.83694500
H	1.82599900	-1.14321000	-0.83088900
C	1.83109300	-4.37435900	-1.91943500
H	0.61748000	-5.97107400	-1.09988700
H	2.91013400	-2.59464300	-2.51462100
H	2.31111700	-5.01150800	-2.66653400
C	0.61814200	-2.10716500	2.83558800
C	-0.04898100	-1.93335900	4.05960200
C	1.95998200	-2.52046800	2.83034800
C	0.62135000	-2.16416600	5.26381400
H	-1.09698700	-1.62319800	4.07098100
C	2.62539900	-2.75542100	4.03736700
H	2.48843100	-2.66187600	1.88508900
C	1.95864400	-2.57678300	5.25432700
H	0.09538100	-2.02715400	6.21168300
H	3.66861500	-3.08044300	4.02554900
H	2.48038100	-2.76301800	6.19632900
C	0.15594300	0.01584000	0.90675300
C	0.76429800	0.84247400	1.86367300
C	-0.20202900	0.55479500	-0.34236300
C	1.00936300	2.18977000	1.57463700
H	1.04717700	0.44281600	2.83890700
C	0.05152100	1.89608000	-0.62914200
H	-0.68805800	-0.07336900	-1.09054200
C	0.65429800	2.71874500	0.33098000
H	1.48128200	2.82541100	2.32782800
H	-0.23178800	2.30343100	-1.60256600
H	0.84563900	3.77123500	0.10768800
C	-6.38217800	-1.52448300	0.58345400
C	-5.79787400	-2.41601700	-0.32503400
C	-4.91844900	-3.42201200	0.10353300
C	-4.64900000	-3.51469700	1.49304500
C	-5.22615700	-2.60689200	2.40425200
C	-6.09060500	-1.60742000	1.94715800
H	-7.05742700	-0.74609000	0.21861900
H	-6.03018400	-2.30770900	-1.38877100
H	-4.05001500	-4.34015200	1.88160500
H	-5.00905400	-2.70346300	3.47116800
H	-6.53250700	-0.89801900	2.65037300
Ge	-4.04792600	-4.62906100	-1.20371400
C	-5.43442500	-5.30395100	-2.43142400
H	-5.96345600	-4.46777600	-2.91507300
H	-4.97641900	-5.92530900	-3.21713900
H	-6.17022000	-5.91435400	-1.88500300
C	-3.16206800	-6.08369600	-0.22957200
H	-2.50236000	-6.64616600	-0.90852500
H	-2.56620000	-5.64700000	0.58599500
H	-3.90540500	-6.77664300	0.19493400
C	-2.73230400	-3.57406100	-2.22461200
H	-2.19009400	-4.22253200	-2.93049100
H	-3.23727400	-2.77945100	-2.79535300
H	-2.00411400	-3.11472700	-1.54042100
F	-1.83010400	-4.23109900	2.10350200

Zero-point correction= 0.569166 (Hartree/Particle)

Thermal correction to Energy= 0.608010

Thermal correction to Enthalpy= 0.608955

Thermal correction to Gibbs Free Energy= 0.494493

Sum of electronic and zero-point Energies= -3922.429790

Sum of electronic and thermal Energies= -3922.390945

Sum of electronic and thermal Enthalpies= -3922.390001

Sum of electronic and thermal Free Energies= -3922.504463
E(B3LYPD3/Def2TZVPP) = -3924.930841

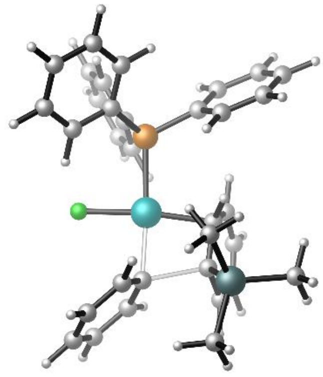

C	-4.17754500	0.14257800	1.05947900
C	-5.03714500	1.07069000	0.46033600
C	-5.60142300	0.80218100	-0.79080900
C	-5.29653400	-0.39960300	-1.44546600
C	-4.44821500	-1.33055100	-0.84353800
C	-3.86515500	-1.06660900	0.40933900
H	-3.73175600	0.38592900	2.02401800
H	-5.26428500	2.00702500	0.97717600
H	-6.28003800	1.52294600	-1.25401700
H	-5.72867300	-0.61459100	-2.42662900
H	-4.23334200	-2.27386000	-1.35110700
P	0.59825400	-1.23672800	0.83609000
C	1.54699300	-1.57510300	-0.70036600
C	0.98995500	-2.45548800	-1.64449900
C	2.80793400	-1.00756500	-0.94576000
C	1.69071100	-2.77158300	-2.81196900
H	0.00437700	-2.89291800	-1.46467200
C	3.50295800	-1.32121000	-2.11762300
H	3.24996400	-0.31813900	-0.22320400
C	2.94736500	-2.20411300	-3.05055200
H	1.25082400	-3.45817200	-3.53940900
H	4.48244600	-0.87314500	-2.30236600
H	3.49265200	-2.44677900	-3.96606000
C	1.25995000	-2.43409200	2.06347900
C	0.80843500	-2.33010500	3.39310300
C	2.11475700	-3.49043100	1.71208400
C	1.20967900	-3.26207000	4.35168900
H	0.14538600	-1.51050300	3.68335700
C	2.51033700	-4.42672100	2.67470000
H	2.47342800	-3.58794000	0.68554800
C	2.05964100	-4.31603300	3.99317200
H	0.85473000	-3.16765900	5.38082700
H	3.17600400	-5.24508800	2.38948200
H	2.36942900	-5.04881600	4.74221800
C	1.20161800	0.39553900	1.43440900
C	2.30725400	0.51791700	2.29398300
C	0.53173500	1.55075300	0.98970200
C	2.73692500	1.78306200	2.70707200
H	2.83472000	-0.37091500	2.64622200
C	0.97110000	2.81112400	1.40622100
H	-0.32175400	1.43246100	0.31222900
C	2.06970400	2.93079200	2.26535200
H	3.59632700	1.87058500	3.37660300
H	0.44851300	3.70668200	1.05970500
H	2.40608000	3.91822000	2.59196400
C	-3.76940600	-4.72564200	0.07254100
C	-4.36925700	-3.71766700	0.77249500
C	-3.60415800	-2.75294900	1.55142100
C	-2.15768200	-3.00083000	1.64821700
C	-1.57305200	-4.08307900	0.87758900
C	-2.34421400	-4.89539800	0.09067000
H	-4.38214700	-5.42907000	-0.49815700
H	-5.45598300	-3.60268600	0.73269700
H	-1.66205800	-2.77739700	2.60047000
H	-0.49721900	-4.25242800	0.95974500
H	-1.88759500	-5.69846200	-0.49258000
Ge	-4.43732100	-2.27602600	3.30851200
C	-6.29580000	-1.69227000	3.06634500
H	-6.74627100	-1.49394800	4.05140300
H	-6.34698400	-0.77771500	2.45959900
H	-6.88063800	-2.48215300	2.57045700
C	-3.30412400	-0.94551500	4.22918500
H	-3.86277800	-0.01625500	4.41466800
H	-2.96827800	-1.35069800	5.19539100
H	-2.41495200	-0.70467100	3.62667100
C	-4.38518700	-3.97248800	4.30862600
H	-4.80464300	-3.83117600	5.31709000
H	-4.96833500	-4.74598400	3.78611400
H	-3.34499000	-4.32019100	4.40581700
Pd	-1.73627800	-1.30724000	0.51448400
F	-1.52171800	0.31388900	-0.78890400

Zero-point correction= 0.567509 (Hartree/Particle)
Thermal correction to Energy= 0.605739
Thermal correction to Enthalpy= 0.606683
Thermal correction to Gibbs Free Energy= 0.494132
Sum of electronic and zero-point Energies= -3922.370649
Sum of electronic and thermal Energies= -3922.332419
Sum of electronic and thermal Enthalpies= -3922.331475
Sum of electronic and thermal Free Energies= -3922.444026
E(B3LYPD3/Def2TZVPP) = -3924.870265

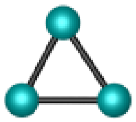

(triplet)

Pd	0.43900700	-2.01151900	1.78009000

Pd	2.18399200	-1.31143400	-0.01562700
Pd	0.11967400	-2.80510700	-0.54307000

Zero-point correction = 0.001161 (Hartree/Particle)

Thermal correction to Energy = 0.005831

Thermal correction to Enthalpy = 0.006775

Thermal correction to Gibbs Free Energy= -0.032457

Sum of electronic and zero-point Energies = -383.764097

Sum of electronic and thermal Energies = -383.759427

Sum of electronic and thermal Enthalpies = -383.758483

Sum of electronic and thermal Free Energies = -383.797715

E(B3LYPD3/Def2TZVPP) = -383.8696309

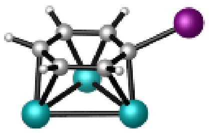

Pd	0.29467400	0.67328100	0.04407500
Pd	0.27429300	-1.51619800	2.16567800
Pd	2.28770400	-1.52456000	-0.31695500
C	2.24444900	1.22907000	0.71279900
C	1.34458300	1.27358900	1.82900500
C	1.35470400	0.22002700	2.83603200
C	2.26544100	-0.87719700	2.71081400
C	3.18883800	-0.94196700	1.58581000
C	3.16423500	0.09423000	0.59069700
H	2.50774300	2.15802700	0.19974500
H	0.92286900	2.24051100	2.12532300
H	0.95298300	0.47480900	3.82275200
H	2.54541800	-1.45040800	3.60157200
H	4.08956500	-1.54722600	1.72009400
I	5.09393000	0.61705100	-0.38595000

Zero-point correction = 0.089141 (Hartree/Particle)

Thermal correction to Energy = 0.101199

Thermal correction to Enthalpy = 0.102143

Thermal correction to Gibbs Free Energy= 0.045050

Sum of electronic and zero-point Energies = -913.008426

Sum of electronic and thermal Energies = -912.996369

Sum of electronic and thermal Enthalpies = -912.995424

Sum of electronic and thermal Free Energies = -913.052517

E(B3LYPD3/Def2TZVPP) = -913.4613999

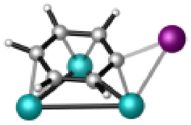

Pd	0.29640200	0.51643100	0.08297200
Pd	0.19264800	-1.42422000	2.28284500
Pd	2.41346100	-1.27012500	-0.69864700
C	2.22156000	1.18106200	0.71034000
C	1.33426000	1.25599200	1.83636000
C	1.36905300	0.24114000	2.87777700
C	2.29858800	-0.84694600	2.76239100
C	3.19352400	-0.93052600	1.64968900
C	3.14346200	0.04719700	0.60948800
H	2.46047900	2.08844500	0.14967000
H	0.89030100	2.22363600	2.09414700
H	0.99132100	0.52642500	3.86629500
H	2.54637600	-1.44561100	3.64503800
H	4.02712500	-1.63293900	1.70162700
I	5.15287100	0.59307800	-0.32849900

Zero-point correction = 0.088760 (Hartree/Particle)

Thermal correction to Energy = 0.100222

Thermal correction to Enthalpy = 0.101166

Thermal correction to Gibbs Free Energy= 0.046113

Sum of electronic and zero-point Energies = -913.007749

Sum of electronic and thermal Energies = -912.996286

Sum of electronic and thermal Enthalpies = -912.995342

Sum of electronic and thermal Free Energies = -913.050395

E(B3LYPD3/Def2TZVPP) = -913.4603917

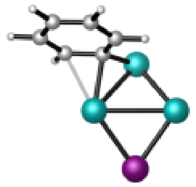

Pd	-1.02179600	-1.21590500	0.34618400
Pd	-3.07113800	-1.33969400	-1.54280400
Pd	-1.90853800	-3.68857100	-0.02712800
C	-3.77791700	-0.80298700	1.30297100
C	-4.91460900	-0.02507400	1.51141900
C	-5.13376300	1.14707500	0.76123300
C	-4.20125200	1.54488500	-0.19273300
C	-3.02924300	0.78375700	-0.41780200
C	-2.81147600	-0.41860300	0.32812600
H	-3.62574500	-1.72009500	1.87859400
H	-5.64916400	-0.33690100	2.25953500
H	-6.03116100	1.74614600	0.93540700
H	-4.34923300	2.47110500	-0.75528000
H	-2.24016400	1.20418500	-1.04929900
I	-2.23697400	-3.62166600	-2.64970100

Zero-point correction = 0.090734 (Hartree/Particle)

Thermal correction to Energy = 0.103258

Thermal correction to Enthalpy = 0.104202

Thermal correction to Gibbs Free Energy= 0.046177

Sum of electronic and zero-point Energies =-913.072860

Sum of electronic and thermal Energies = -913.060337

Sum of electronic and thermal Enthalpies = -913.059393

Sum of electronic and thermal Free Energies = -913.117418

E(B3LYPD3/Def2TZVPP) = -913.5303255

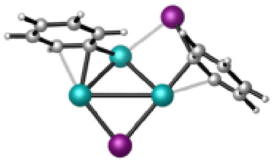

C	2.82523500	-4.20177100	-2.27490100
C	2.95352900	-3.45801300	-1.10665700
C	3.37323700	-2.09663400	-1.19045700
C	3.66830100	-1.51161300	-2.46024900
C	3.53066500	-2.30945500	-3.62491800
C	3.12177900	-3.63512800	-3.53306900
H	2.49018300	-5.24024700	-2.20941600
H	2.75666600	-3.91284300	-0.13444100
H	4.17829500	-0.54820300	-2.52738700
H	3.78319400	-1.87271300	-4.59468900
H	3.03430600	-4.24439100	-4.43562700
I	4.55314000	-1.33869900	0.62847200
Pd	2.35651800	0.31919100	1.04880400
Pd	-0.32419500	0.35113900	1.11475300
Pd	1.70520400	-0.91788200	-1.35580500
C	1.06166000	-2.03469600	2.35454300
C	0.26151600	-2.61511300	3.33262300
C	-0.42834800	-1.82098400	4.27396100
C	-0.30551300	-0.43827400	4.24055700
C	0.50210900	0.19299200	3.25599000
C	1.16771100	-0.61955600	2.28251500
H	1.58592000	-2.65427000	1.62638700
H	0.15983200	-3.70342700	3.36335700
H	-1.04336800	-2.29867000	5.04038300
H	-0.79014800	0.17922200	5.00152200
H	0.75882900	1.24963600	3.38715600
I	-0.60435300	0.58193500	-1.54690900

Zero-point correction = 0.180489 (Hartree/Particle)

Thermal correction to Energy = 0.200866

Thermal correction to Enthalpy = 0.201810

Thermal correction to Gibbs Free Energy= 0.124668

Sum of electronic and zero-point Energies = -1442.307830

Sum of electronic and thermal Energies = -1442.287452

Sum of electronic and thermal Enthalpies = -1442.286508

Sum of electronic and thermal Free Energies = -1442.363651

E(B3LYPD3/Def2TZVPP) = -1443.115731

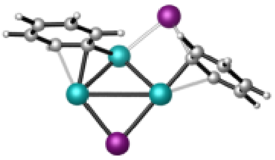

C	2.89827500	-4.26897300	-2.36591100
C	2.93653500	-3.50833100	-1.19907400
C	3.36226900	-2.15055100	-1.27271500
C	3.76806600	-1.58755400	-2.51826600
C	3.71795500	-2.39493900	-3.67735600
C	3.29484600	-3.71997600	-3.60128400
H	2.55518000	-5.30562900	-2.31358800
H	2.66408200	-3.94813800	-0.23824600
H	4.25032800	-0.60886500	-2.55847000
H	4.04265800	-1.97118300	-4.63133200
H	3.27472100	-4.33945200	-4.50092000
I	4.51314100	-1.40034800	0.64797400
Pd	2.35186500	0.25001500	0.96889900
Pd	-0.31040100	0.38535000	1.09788000
Pd	1.70133600	-1.01101700	-1.39776000
C	1.01476400	-2.05072500	2.32444700
C	0.21336000	-2.59881100	3.31958300
C	-0.43174900	-1.77711800	4.26943600
C	-0.26246400	-0.39974800	4.22711100
C	0.54827300	0.19935300	3.22489600
C	1.16680800	-0.64020200	2.24349900
H	1.50526700	-2.69087000	1.59032300
H	0.07577700	-3.68291800	3.35840900
H	-1.04774800	-2.23036700	5.04978000
H	-0.71124600	0.23713900	4.99404100
H	0.84422200	1.24673900	3.34687800
I	-0.58421500	0.55865100	-1.57173500

Zero-point correction = 0.180184 (Hartree/Particle)

Thermal correction to Energy = 0.199870

Thermal correction to Enthalpy = 0.200814

Thermal correction to Gibbs Free Energy= 0.125518

Sum of electronic and zero-point Energies =-1442.308109

Sum of electronic and thermal Energies = -1442.288423

Sum of electronic and thermal Enthalpies = -1442.287479

Sum of electronic and thermal Free Energies = -1442.362775

E(B3LYPD3/Def2TZVPP) = -1443.115676

Int5

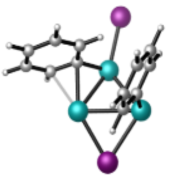

C	1.41638200	-3.94531600	-2.02727900
C	0.97879900	-2.61408600	-1.97854200
C	1.93184900	-1.59380900	-1.87579400
C	3.30059400	-1.88534300	-1.80565000
C	3.72105600	-3.22150500	-1.86468700
C	2.78040900	-4.25183600	-1.96960200

H	0.67664200	-4.74663100	-2.10697200
H	-0.08592400	-2.38556800	-2.01476100
H	4.04307400	-1.09011700	-1.68702600
H	4.78877400	-3.45029200	-1.81398600
H	3.11029200	-5.29301500	-2.00242000
I	4.80330900	1.41851900	1.60170400
Pd	2.37628700	1.00960700	0.62781500
Pd	-0.03603800	-0.18463000	0.54068500
Pd	1.57633100	0.30068900	-1.92783600
C	2.37500700	-1.78870400	1.61076100
C	1.90470100	-2.82103100	2.41120500
C	0.87901500	-2.59632400	3.35589900
C	0.33464900	-1.32869800	3.50426200
C	0.79285900	-0.24922500	2.70039200
C	1.79805100	-0.49374900	1.71812200
H	3.16369600	-1.97323900	0.88167000
H	2.33576300	-3.82012900	2.30445900
H	0.53206900	-3.41905200	3.98581600
H	-0.42134700	-1.13550200	4.26962100
H	0.50279800	0.77020000	2.97220500
I	-1.20719100	0.57031700	-1.73356100

Zero-point correction = 0.182118 (Hartree/Particle)

Thermal correction to Energy = 0.202309

Thermal correction to Enthalpy = 0.203253

Thermal correction to Gibbs Free Energy= 0.125750

Sum of electronic and zero-point Energies = -1442.339557

Sum of electronic and thermal Energies = -1442.319366

Sum of electronic and thermal Enthalpies = -1442.318421

Sum of electronic and thermal Free Energies = -1442.395925

E(B3LYPD3/Def2TZVPP) = -1443.150513

Int1

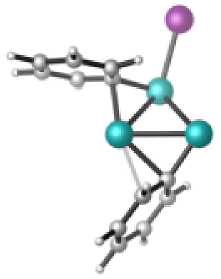

C	1.90028300	-3.56225300	-2.54679800
C	2.63379900	-3.67188000	-1.37377900
C	3.29089600	-2.52445700	-0.84724500
C	3.25242200	-1.29227000	-1.56404300
C	2.49665200	-1.21695900	-2.76792800
C	1.82317000	-2.33212600	-3.24184200
H	1.38064200	-4.43948900	-2.94027600
H	2.69813800	-4.62866500	-0.85079900
H	3.93217000	-0.47701000	-1.30763700
H	2.48139200	-0.27796000	-3.32577300
H	1.25067500	-2.27218400	-4.17025400
I	6.57844700	-3.02624200	-0.27231700
Pd	3.59750300	-0.09817700	1.95571100
Pd	1.82604000	-1.16013700	0.20884700
Pd	4.22474500	-2.69004600	0.83538800
C	1.63090400	-1.77557500	3.31749500
C	0.33808000	-2.21509600	3.59410500
C	-0.77902400	-1.57476400	3.02288400
C	-0.60593500	-0.47908900	2.18484100
C	0.69709800	-0.00510800	1.88519900
C	1.81923900	-0.68741000	2.43275800
H	2.49483100	-2.28086900	3.75679300
H	0.19341300	-3.07409200	4.25433900
H	-1.78524500	-1.92929900	3.25768600
H	-1.47025800	0.05495700	1.78293300
H	0.81105400	0.96172200	1.38315900

Zero-point correction = 0.181331 (Hartree/Particle)

Thermal correction to Energy = 0.199072

Thermal correction to Enthalpy = 0.200016

Thermal correction to Gibbs Free Energy= 0.129649

Sum of electronic and zero-point Energies = -1144.318742

Sum of electronic and thermal Energies = -1144.301001

Sum of electronic and thermal Enthalpies = -1144.300056

Sum of electronic and thermal Free Energies = -1144.370424

E(B3LYPD3/Def2TZVPP) = -1145.118764

Int2

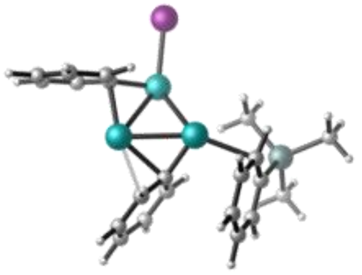

C	-6.29893200	1.06862400	-1.70595400
C	-5.62555000	2.29659400	-1.57195500
C	-5.72277800	3.29242900	-2.54880500
C	-6.53987900	3.02717900	-3.68634300
C	-7.23350400	1.79658500	-3.81374000
C	-7.10588900	0.81678700	-2.81188900
H	-6.19123500	0.30830900	-0.92832600
H	-5.01287400	2.46212600	-0.68222700
H	-6.49049900	3.69498800	-4.55517400
H	-7.78644300	1.57363300	-4.72998500
H	-7.63108400	-0.13526200	-2.91134400
Ge	-4.74903500	5.00851400	-2.40291500
C	-3.73546600	4.99651100	-0.72017700

H	-4.41531500	4.88366400	0.13852600
H	-3.00873600	4.16966800	-0.70644500
H	-3.18768100	5.94480600	-0.60857400
C	-6.04684500	6.48871600	-2.42241700
H	-5.52088200	7.45593900	-2.43600000
H	-6.68323500	6.42250300	-3.31848000
H	-6.68684000	6.45227200	-1.52763600
C	-3.56086600	5.15926900	-3.96484200
H	-3.02246400	6.11948100	-3.93894700
H	-2.82491700	4.34066200	-3.97095900
H	-4.14609100	5.11327600	-4.89658000
C	-10.05925700	1.59947600	0.21546000
C	-9.80203900	2.05877700	-1.10130500
C	-8.93746400	3.17176600	-1.28452000
C	-8.29459100	3.76766000	-0.17166500
C	-8.56593700	3.29253700	1.10747100
C	-9.44884900	2.21162700	1.30355300
H	-10.70740900	0.73145000	0.35687100
H	-10.13539300	1.45715100	-1.95127600
H	-7.60055500	4.59542500	-0.31901100
H	-8.08737100	3.76729300	1.96766700
H	-9.63658800	1.84232100	2.31431500
I	-10.06570400	8.72307800	-3.71394200
Pd	-9.72538200	6.47079200	-2.41157500
Pd	-10.97777900	3.97542400	-1.72836800
Pd	-8.62897100	3.83523500	-3.08417600
C	-12.40429100	6.61028800	-1.24835400
C	-13.73367400	6.22851300	-1.12730500
C	-14.31651700	5.32814000	-2.04931600
C	-13.56645300	4.81760100	-3.09863600
C	-12.19748300	5.17709300	-3.24380700
C	-11.60514300	6.05674000	-2.28862600
H	-11.96735700	7.32108700	-0.54434700
H	-14.33844600	6.63647100	-0.31346900
H	-15.36943700	5.05599500	-1.94617100
H	-14.02312400	4.15932600	-3.84114200
H	-11.67475400	4.92345200	-4.16888600

Zero-point correction = 0.383630 (Hartree/Particle)

Thermal correction to Energy = 0.415315

Thermal correction to Enthalpy = 0.416259

Thermal correction to Gibbs Free Energy= 0.315796

Sum of electronic and zero-point Energies = -3572.118026

Sum of electronic and thermal Energies = -3572.086341

Sum of electronic and thermal Enthalpies = -3572.085396

Sum of electronic and thermal Free Energies = -3572.185860

E(B3LYPD3/Def2TZVPP) = -3573.772416

TS3

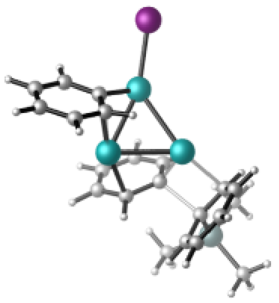

C	-7.19185400	1.35063000	-3.63267200
C	-6.71156100	2.11032000	-2.58640200
C	-6.47739100	3.52582300	-2.70707000
C	-6.77948500	4.12064600	-4.00656000
C	-7.27882100	3.31161300	-5.07072400
C	-7.49622300	1.95249800	-4.87822100
H	-7.33034300	0.27454700	-3.50630300
H	-6.48355400	1.61918400	-1.63674500
H	-6.37596300	5.11035600	-4.24805700
H	-7.46079300	3.76914900	-6.04588400
H	-7.88644300	1.34315200	-5.69617600
Ge	-4.78621200	4.07621100	-1.72687600
C	-5.05008700	3.67329100	0.17344300
H	-5.84898200	4.29674000	0.59878200
H	-5.30557600	2.61306800	0.31854700
H	-4.11357400	3.88045300	0.71406300
C	-4.34720500	5.96306900	-2.05352700
H	-3.25377500	6.06600800	-1.97408200
H	-4.64593700	6.28399100	-3.06275400
H	-4.81332100	6.62499600	-1.31145100
C	-3.40236700	2.92575100	-2.51906100
H	-2.42942700	3.14242100	-2.05098300
H	-3.64633900	1.86495400	-2.35949800
H	-3.32351600	3.11355600	-3.60084100
C	-8.93999300	4.06340900	0.60850700
C	-8.56386400	3.58777700	-0.67227400
C	-7.90522800	4.48249200	-1.58392300
C	-7.59284100	5.79681100	-1.15916800
C	-7.98511100	6.23682900	0.10744900
C	-8.66649900	5.38502600	0.98608900
H	-9.38110000	3.36851000	1.32675900
H	-8.57722500	2.51421800	-0.87470400
H	-7.06716400	6.47973600	-1.82625200
H	-7.76451500	7.26482100	0.40425700
H	-8.96029300	5.73889700	1.97621600
I	-12.26540100	8.36695600	-3.60537900
Pd	-10.88989600	6.42384500	-2.48714400
Pd	-10.62786300	3.90264500	-1.43542200
Pd	-8.77443400	4.64080400	-3.39064100
C	-13.25966400	5.47846000	-1.02936700

C	-14.21247600	4.52659600	-0.68880500
C	-14.29057300	3.29779600	-1.38204700
C	-13.42075100	3.03099800	-2.42858600
C	-12.42999400	3.98253700	-2.80326300
C	-12.32755800	5.20335700	-2.06807100
H	-13.21592000	6.43066200	-0.49749100
H	-14.91202800	4.73448000	0.12513000
H	-15.05735200	2.56941800	-1.10783400
H	-13.51194100	2.10731300	-3.00527400
H	-11.90857600	3.85517100	-3.75645600

Zero-point correction = 0.381568 (Hartree/Particle)

Thermal correction to Energy = 0.412878

Thermal correction to Enthalpy = 0.413822

Thermal correction to Gibbs Free Energy= 0.314537

Sum of electronic and zero-point Energies = -3572.081967

Sum of electronic and thermal Energies = -3572.050657

Sum of electronic and thermal Enthalpies = -3572.049713

Sum of electronic and thermal Free Energies = -3572.148998

E(B3LYPD3/Def2TZVPP) = -3573.731902

Int3

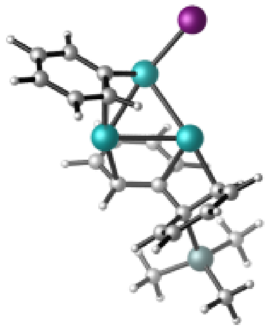

C	-7.49844000	1.71247700	-3.51184700
C	-7.15701500	2.37659500	-2.37210700
C	-6.79361500	3.80967400	-2.35141200
C	-6.95101400	4.50292400	-3.67825900
C	-7.27921300	3.74871000	-4.85268500
C	-7.58707100	2.40810300	-4.76282500
H	-7.70952700	0.64184700	-3.47963400
H	-7.10386600	1.81936700	-1.43442100
H	-6.39077800	5.43352400	-3.83740800
H	-7.29051900	4.25680000	-5.81950400
H	-7.88208500	1.85691800	-5.65898300
Ge	-4.78326300	3.77875200	-1.76125400
C	-4.83263100	3.20450700	0.11369000
H	-5.48558500	3.86927600	0.69911600
H	-5.19769600	2.17092000	0.20345600
H	-3.81514500	3.25641000	0.53113400
C	-4.04546100	5.59039000	-1.93246500
H	-2.95724500	5.51811200	-1.77734400
H	-4.22302300	6.01239700	-2.93250700
H	-4.46046000	6.26543600	-1.17122800
C	-3.86320100	2.51573200	-2.94457800
H	-2.78569100	2.52526800	-2.71894900
H	-4.25042800	1.49712000	-2.79943600
H	-4.00547500	2.80577400	-3.99652600
C	-9.06214700	4.76553500	0.68036800
C	-8.43532200	4.00195700	-0.33540200
C	-7.60484000	4.65109300	-1.32420500
C	-7.40606300	6.05340900	-1.19411800
C	-8.03763700	6.78725000	-0.17403700
C	-8.87916100	6.16204700	0.74258000
H	-9.58377200	4.25178600	1.49131300
H	-8.38081100	2.91759900	-0.21625600
H	-6.69815700	6.57123000	-1.84136300
H	-7.85561600	7.86200600	-0.10766200
H	-9.36201300	6.73797600	1.53436800
I	-11.96316200	7.69842500	-4.23375600
Pd	-11.07465600	6.43884200	-2.11045000
Pd	-10.45570000	3.95675000	-1.21048400
Pd	-8.80987700	5.28190200	-3.23805300
C	-13.42123300	5.07951500	-1.08399800
C	-14.24527300	3.98107200	-0.86339700
C	-14.02780200	2.76540800	-1.54646800
C	-12.99769800	2.65959000	-2.47031900
C	-12.14013700	3.76568700	-2.72448300
C	-12.33194500	4.97063700	-1.98775100
H	-13.60049600	6.01665800	-0.55146900
H	-15.06837000	4.06212100	-0.14859100
H	-14.69037500	1.91578800	-1.36506500
H	-12.85965500	1.74046800	-3.04512800
H	-11.48864500	3.73940600	-3.60234400

Zero-point correction = 0.382617 (Hartree/Particle)

Thermal correction to Energy = 0.414380

Thermal correction to Enthalpy = 0.415324

Thermal correction to Gibbs Free Energy= 0.314279

Sum of electronic and zero-point Energies = -3572.103530

Sum of electronic and thermal Energies = -3572.071767

Sum of electronic and thermal Enthalpies = -3572.070823

Sum of electronic and thermal Free Energies = -3572.171868

E(B3LYPD3/Def2TZVPP) = -3573.752406

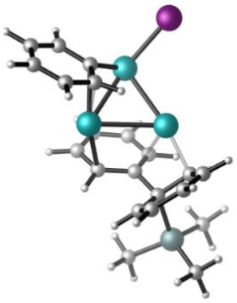

C	-7.45030600	1.69846000	-3.49295900
C	-7.13136200	2.37129600	-2.35106400
C	-6.78693100	3.80765900	-2.33206700
C	-6.93671100	4.49545400	-3.66078200
C	-7.23670400	3.73335000	-4.83756900
C	-7.53292600	2.38893200	-4.74623300
H	-7.64781100	0.62532800	-3.45950800
H	-7.08262000	1.81807600	-1.41084400
H	-6.38734200	5.43400900	-3.81326900
H	-7.23515100	4.23610100	-5.80728400
H	-7.81208300	1.83204500	-5.64395600
Ge	-4.76483100	3.78474900	-1.75584500
C	-4.79883400	3.21126000	0.11929200
H	-5.45241900	3.87233300	0.70808100
H	-5.15602600	2.17526000	0.21215100
H	-3.77877100	3.27108600	0.52935200
C	-4.04033800	5.60095200	-1.93328900
H	-2.95169900	5.53547100	-1.77793600
H	-4.22045000	6.01827200	-2.93478600
H	-4.45945100	6.27567600	-1.17412000
C	-3.84820600	2.52808200	-2.94789300
H	-2.76808900	2.55086700	-2.73603100
H	-4.22194100	1.50573000	-2.79471800
H	-4.00717700	2.81372100	-3.99859200
C	-9.05586500	4.74656200	0.69965800
C	-8.41772100	3.98775000	-0.31359200
C	-7.58394200	4.64022300	-1.29175500
C	-7.39637200	6.04033200	-1.16318400
C	-8.03301900	6.77089800	-0.14625600
C	-8.87626100	6.14178500	0.76735700
H	-9.58814900	4.22860100	1.50087300
H	-8.37172400	2.90213100	-0.20308000
H	-6.70448900	6.56468400	-1.82230800
H	-7.85766500	7.84681000	-0.08016100
H	-9.36583000	6.71541300	1.55669500
I	-11.92657700	7.70262300	-4.24689700
Pd	-11.03351500	6.44486500	-2.12469500
Pd	-10.42870700	3.95899700	-1.22538900
Pd	-8.81410800	5.25394600	-3.30830000
C	-13.38369700	5.09566600	-1.08571100
C	-14.20989400	4.00000100	-0.86041200
C	-13.99939100	2.78265900	-1.54327600
C	-12.97444900	2.67272800	-2.47205600
C	-12.11459300	3.77624200	-2.73161800
C	-12.29846200	4.98239800	-1.99414700
H	-13.55756000	6.03385600	-0.55329200
H	-15.02948300	4.08413100	-0.14193200
H	-14.66373000	1.93531600	-1.35767700
H	-12.84228600	1.75276500	-3.04692000
H	-11.46976000	3.74835500	-3.61436400

Zero-point correction = 0.382596 (Hartree/Particle)

Thermal correction to Energy = 0.413568

Thermal correction to Enthalpy = 0.414513

Thermal correction to Gibbs Free Energy= 0.315230

Sum of electronic and zero-point Energies = -3572.103510

Sum of electronic and thermal Energies = -3572.072538

Sum of electronic and thermal Enthalpies = -3572.071594

Sum of electronic and thermal Free Energies = -3572.170877

E(B3LYPD3/Def2TZVPP) = -3573.752154

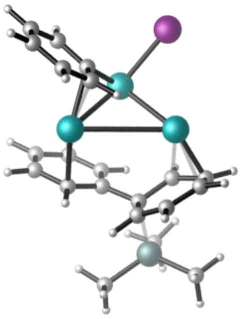

C	-7.38727100	1.82900900	-3.51374500
C	-7.15719600	2.41799300	-2.30726300
C	-6.93324600	3.87207000	-2.17616900
C	-7.05233900	4.61181700	-3.45798700
C	-6.95503700	3.95790000	-4.71934700
C	-7.38965900	2.60874400	-4.73387100
H	-7.55823200	0.75305000	-3.57967700
H	-7.10966600	1.79418400	-1.41408400
H	-6.87672500	5.68965200	-3.43875700
H	-6.63955600	4.48282100	-5.62426900
H	-7.48055700	2.08540400	-5.68935400
Ge	-4.82738900	3.88941000	-1.75861600
C	-4.66294100	2.94560800	-0.04817700
H	-5.34863200	3.37888600	0.69497300
H	-4.88396800	1.87490700	-0.16512700
H	-3.63033600	3.05719600	0.31749800
C	-4.24056600	5.75556700	-1.61745400
H	-3.14271100	5.74927600	-1.52679300
H	-4.51241500	6.33030400	-2.51472600
H	-4.66438300	6.24042800	-0.72763000

C	-3.94051000	2.96985300	-3.24325300
H	-2.87135300	2.86935900	-2.99947800
H	-4.36665000	1.96815600	-3.39390500
H	-4.03602100	3.54891300	-4.17297900
C	-9.05628200	4.47264000	0.99521600
C	-8.36727400	3.81286200	-0.05083700
C	-7.60670900	4.55926800	-0.99996700
C	-7.54246300	5.95009200	-0.83097000
C	-8.21351800	6.59557400	0.21801900
C	-8.98505100	5.87164900	1.12354700
H	-9.56504500	3.87912000	1.75899200
H	-8.29847700	2.72366800	-0.02357100
H	-6.97010600	6.56114300	-1.52845000
H	-8.13371500	7.68111800	0.31131400
H	-9.51326200	6.37457600	1.93591400
I	-11.43425200	7.09521500	-4.84554600
Pd	-10.50027100	5.81845900	-2.74982500
Pd	-10.44726700	3.77672400	-0.99171800
Pd	-9.01122900	4.16370400	-4.36410300
C	-13.06704800	5.39355700	-1.44242100
C	-14.10861600	4.63116900	-0.92690500
C	-14.17098800	3.24024700	-1.15977300
C	-13.19630700	2.61810500	-1.92576300
C	-12.11690300	3.36772300	-2.47306300
C	-12.03411100	4.76490900	-2.19047500
H	-13.03390600	6.47058300	-1.26454100
H	-14.88717900	5.11778600	-0.33359900
H	-15.00449900	2.65993200	-0.75701300
H	-13.26924800	1.55289200	-2.15850600
H	-11.50434100	2.90068000	-3.24807200

Zero-point correction = 0.384262 (Hartree/Particle)

Thermal correction to Energy = 0.415523

Thermal correction to Enthalpy = 0.416468

Thermal correction to Gibbs Free Energy= 0.317717

Sum of electronic and zero-point Energies = -3572.119825

Sum of electronic and thermal Energies = -3572.088564

Sum of electronic and thermal Enthalpies = -3572.087620

Sum of electronic and thermal Free Energies = -3572.186371

E(B3LYPD3/Def2TZVPP) = -3573.771001

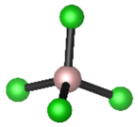

B	-1.62859500	0.20542400	0.00000000
F	-1.16004100	-1.11946800	0.00000000
F	-1.16026700	0.86787600	-1.14758900
F	-1.16026700	0.86787600	1.14758900
F	-3.03390500	0.20568800	0.00000000

Zero-point correction = 0.014414 (Hartree/Particle)

Thermal correction to Energy = 0.018755

Thermal correction to Enthalpy = 0.019699

Thermal correction to Gibbs Free Energy= -0.013172

Sum of electronic and zero-point Energies = -424.276385

Sum of electronic and thermal Energies = -424.272045

Sum of electronic and thermal Enthalpies = -424.271100

Sum of electronic and thermal Free Energies = -424.303972

E(B3LYPD3/Def2TZVPP) = -424.827812

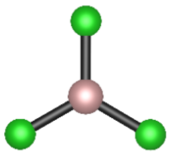

B	-1.26299300	0.20580400	0.00055200
F	-1.26294900	-1.10947400	-0.00063700
F	-1.26291000	0.86239300	-1.13912100
F	-1.26560700	0.86317600	1.13920600

Zero-point correction = 0.012294 (Hartree/Particle)

Thermal correction to Energy = 0.015810

Thermal correction to Enthalpy = 0.016755

Thermal correction to Gibbs Free Energy= -0.013867

Sum of electronic and zero-point Energies = -324.304156

Sum of electronic and thermal Energies = -324.300640

Sum of electronic and thermal Enthalpies = -324.299696

Sum of electronic and thermal Free Energies = -324.330317

E(B3LYPD3/Def2TZVPP) = -324.7172068

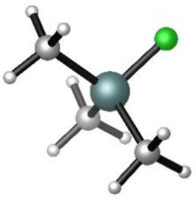

F	-1.28929300	0.68164200	-2.10258800
Ge	-0.82189900	0.32978700	-3.78748800
C	1.12915400	0.28861100	-3.76540300
H	1.48811200	-0.49450400	-3.08040300
H	1.50860200	0.07227500	-4.77651700
H	1.52862500	1.26153300	-3.44104500
C	-1.56436700	1.80410500	-4.82862600
H	-2.65834800	1.84081100	-4.71350800
H	-1.13515200	2.76172800	-4.49684000
H	-1.32766000	1.66334200	-5.89506400
C	-1.64047500	-1.39783200	-4.18084900
H	-2.73420100	-1.33582900	-4.07456100
H	-1.40428400	-1.69548400	-5.21467900
H	-1.25547200	-2.16908700	-3.49653600

Zero-point correction = 0.110608 (Hartree/Particle)

Thermal correction to Energy = 0.119739

Thermal correction to Enthalpy = 0.120683

Thermal correction to Gibbs Free Energy= 0.076877

Sum of electronic and zero-point Energies = -2296.167865

Sum of electronic and thermal Energies = -2296.158734

Sum of electronic and thermal Enthalpies = -2296.157789

Sum of electronic and thermal Free Energies = -2296.201596

E(B3LYPD3/Def2TZVPP) = -2296.813263

Int4

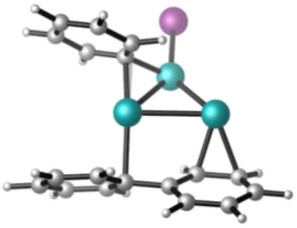

C	-6.30297700	1.09560700	-2.69782500
C	-6.80718100	1.69194500	-1.53002500
C	-7.30902800	3.00229600	-1.54011300
C	-7.26629700	3.73976600	-2.77849000
C	-6.75233200	3.12668900	-3.96085000
C	-6.29295800	1.78879000	-3.90992200
H	-5.90478400	0.07905000	-2.65058400
H	-6.78651000	1.12922400	-0.59502100
H	-7.39672200	4.82439800	-2.75542800
H	-6.54481600	3.73708400	-4.84398300
H	-5.88750800	1.32229300	-4.81069600
C	-8.84253400	3.40268600	1.94396600
C	-8.47702900	2.83754000	0.71066200
C	-7.86510800	3.62904600	-0.30780800
C	-7.70843000	5.02149600	-0.03991800
C	-8.07624700	5.56601300	1.19059800
C	-8.63164700	4.75950600	2.19545000
H	-9.29102300	2.76375500	2.70865500
H	-8.60156100	1.76351900	0.55900500
H	-7.23819400	5.66211800	-0.78740200
H	-7.91684600	6.63203100	1.37062100
H	-8.90793500	5.19230900	3.15951800
I	-12.02730000	7.04334100	-4.72399000
Pd	-10.46069100	5.32153700	-3.46975000
Pd	-9.99280500	3.74831700	-1.18381700
Pd	-9.01237200	3.03962000	-3.84298700
C	-12.17106000	5.72888600	-1.08298400
C	-13.08379000	5.32474600	-0.11298100
C	-13.53214400	3.98853200	-0.05309500
C	-13.06360600	3.05531000	-0.96973700
C	-12.14067800	3.43760700	-1.97999400
C	-11.66802100	4.78626600	-2.02803700
H	-11.84268400	6.76858800	-1.12959000
H	-13.45635500	6.05551600	0.61001400
H	-14.25833500	3.69227700	0.70792000
H	-13.43207900	2.02622400	-0.95141700
H	-11.95171700	2.74767000	-2.80803600

Zero-point correction = 0.272460 (Hartree/Particle)

Thermal correction to Energy = 0.295565

Thermal correction to Enthalpy = 0.296509

Thermal correction to Gibbs Free Energy= 0.213606

Sum of electronic and zero-point Energies = -1375.916726

Sum of electronic and thermal Energies = -1375.893621

Sum of electronic and thermal Enthalpies = -1375.892677

Sum of electronic and thermal Free Energies = -1375.975580

E(B3LYPD3/Def2TZVPP) = -1377.052438

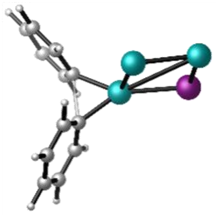

(triplet)

C	-3.35594500	-0.80495000	-0.20728200
C	-4.50218400	-1.57077700	-0.44876600
C	-4.71681100	-2.76286300	0.25254000
C	-3.78005800	-3.18051000	1.20418700
C	-2.63222200	-2.41883200	1.45141000
C	-2.39740900	-1.23753900	0.72732800
H	-3.21527500	0.13165400	-0.75366900
H	-5.23246100	-1.22764800	-1.18643200
H	-5.61487600	-3.35700100	0.06671800
H	-3.94283300	-4.10300500	1.76797100
H	-1.93447200	-2.74954700	2.22264400
C	-2.29513000	2.49583600	2.67736600
C	-2.06543600	1.58845900	1.63696100
C	-1.54621700	0.31001200	1.91099700
C	-1.30818800	-0.05984700	3.24595800
C	-1.53873700	0.85322600	4.28148900
C	-2.02974600	2.13345900	4.00275700
H	-2.69100600	3.48855300	2.44701600
H	-2.29839500	1.88605600	0.61123700
H	-0.95707700	-1.06252000	3.49592300
H	-1.33873900	0.55441300	5.31397900
H	-2.21589400	2.84065700	4.81472000
I	1.62319600	-0.40153800	-1.39081400
Pd	3.05936000	-2.15589000	-0.02827200
Pd	0.99982500	-2.29721900	1.50881600
Pd	-0.52101000	-0.51055600	0.33417300

Zero-point correction = 0.181836 (Hartree/Particle)

Thermal correction to Energy = 0.199160

Thermal correction to Enthalpy = 0.200105

Thermal correction to Gibbs Free Energy=0.129739
Sum of electronic and zero-point Energies = -1144.284210
Sum of electronic and thermal Energies = -1144.266886
Sum of electronic and thermal Enthalpies = -1144.265941
Sum of electronic and thermal Free Energies = -1144.336307
E(B3LYPD3/Def2TZVPP) = -1145.078664

Int2-BF$_4$

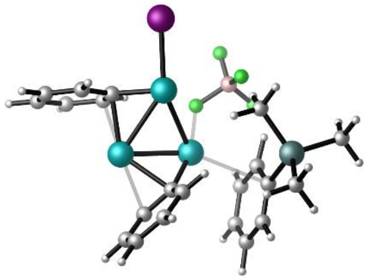

C	-6.40120000	0.90314800	-1.88874900
C	-5.75762000	2.15401900	-1.81951700
C	-5.96493500	3.13545700	-2.79230900
C	-6.84681600	2.83480000	-3.86431700
C	-7.50284000	1.58141100	-3.93723600
C	-7.27337200	0.61429100	-2.93256900
H	-6.21671600	0.15753400	-1.11119100
H	-5.08726500	2.35092700	-0.97831200
H	-6.93777900	3.52185800	-4.70813700
H	-8.08421900	1.33228200	-4.82858700
H	-7.77054700	-0.35651600	-2.99008800
Ge	-5.08176200	4.90103300	-2.74138600
C	-4.22392100	5.12036400	-0.98538800
H	-4.96842800	5.03315700	-0.17876200
H	-3.44620400	4.35663700	-0.82987400
H	-3.75348700	6.11360300	-0.92009300
C	-6.44795300	6.28503400	-3.04446700
H	-5.96790300	7.25245600	-3.25884300
H	-7.08155100	6.00296700	-3.89806600
H	-7.08529200	6.40635400	-2.15470100
C	-3.75602500	4.95598300	-4.19656900
H	-3.26150500	5.93947200	-4.22670300
H	-2.99022100	4.17889600	-4.04897400
H	-4.25079200	4.78265100	-5.16492800
C	-9.95559000	1.91203100	0.80638300
C	-9.88836400	2.01901100	-0.60728000
C	-9.05619600	3.01500000	-1.19359900
C	-8.26650600	3.84450200	-0.35855300
C	-8.35291300	3.71935200	1.02395500
C	-9.19882800	2.75606000	1.60984500
H	-10.57786900	1.13189100	1.25132500
H	-10.34300700	1.23508300	-1.22101000
H	-7.60792700	4.59030000	-0.80118800
H	-7.76019000	4.38194900	1.65999200
H	-9.24407500	2.66292500	2.69738900
I	-10.65553000	8.44035400	-3.81895600
Pd	-10.11306000	6.05133600	-2.85364800
Pd	-11.20862600	3.73498100	-1.47757100
Pd	-9.16492000	3.25205200	-3.12329800
C	-12.61836300	6.38883800	-1.31614500
C	-13.91148500	6.02556500	-0.96346800
C	-14.57879600	4.97307200	-1.63305500
C	-13.94843500	4.28928300	-2.66189100
C	-12.61842300	4.62646300	-3.03856000
C	-11.93721600	5.66950400	-2.33880100
H	-12.11898800	7.21666600	-0.81106300
H	-14.42259300	6.56722900	-0.16342900
H	-15.60295500	4.71707900	-1.35202200
H	-14.47283400	3.50601400	-3.21407700
H	-12.20098500	4.21404700	-3.96107900
B	-9.02996100	4.17143300	-6.29566700
F	-9.82329700	4.64598100	-7.32290500
F	-9.89760300	3.61382100	-5.27186000
F	-8.18007600	3.15105600	-6.72120100
F	-8.29111400	5.20442400	-5.70562100

Zero-point correction= 0.399437 (Hartree/Particle)
Thermal correction to Energy= 0.437087
Thermal correction to Enthalpy= 0.438032
Thermal correction to Gibbs Free Energy= 0.323566
Sum of electronic and zero-point Energies= -3996.432904
Sum of electronic and thermal Energies= -3996.395253
Sum of electronic and thermal Enthalpies= -3996.394309
Sum of electronic and thermal Free Energies= -3996.508775
E(B3LYPD3/Def2TZVPP) = -3998.61803113

TS3-BF$_4$

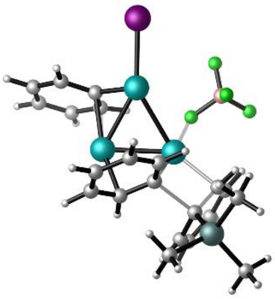

C	-7.12821300	0.84654900	-3.18463700
C	-6.74944000	1.83616900	-2.30604300
C	-6.67001400	3.22358500	-2.69846900
C	-7.04461100	3.52132800	-4.08034700
C	-7.44249500	2.47375600	-4.96530400
C	-7.49107800	1.16372300	-4.52008800
H	-7.14123000	-0.19558600	-2.85673100

H	-6.47345500	1.56470100	-1.28375700
H	-6.75649400	4.48313800	-4.51893200
H	-7.71865300	2.73298400	-5.98887500
H	-7.80294900	0.36809700	-5.20036200
Ge	-4.95985800	4.06327100	-1.98406100
C	-5.02118200	3.96973100	-0.02701100
H	-5.84869700	4.57537800	0.36805300
H	-5.14216500	2.93004900	0.31147400
H	-4.07339300	4.35800400	0.37709500
C	-4.71991800	5.89414200	-2.66179200
H	-3.64283600	6.04971400	-2.82807100
H	-5.23786600	6.04914600	-3.62008900
H	-5.07372500	6.64128100	-1.93850700
C	-3.55164200	2.90382100	-2.71818400
H	-2.56462200	3.27816300	-2.40450700
H	-3.67401600	1.87172100	-2.35799700
H	-3.59546800	2.90409900	-3.81816100
C	-8.99813600	4.11884800	0.62154200
C	-8.66442500	3.47839900	-0.59896200
C	-8.06505300	4.24406300	-1.65962000
C	-7.80072400	5.62224600	-1.43820700
C	-8.15212700	6.22626000	-0.23206600
C	-8.75245200	5.48520300	0.79729800
H	-9.39386300	3.52216200	1.44663700
H	-8.66431100	2.38690200	-0.65011200
H	-7.34791900	6.22196000	-2.22698100
H	-7.96760700	7.29474600	-0.09801100
H	-9.01119600	5.96784300	1.74185600
I	-12.50227700	8.08157000	-3.67921600
Pd	-11.04354700	6.06329500	-2.81932000
Pd	-10.76955100	3.70655400	-1.34840700
Pd	-9.01713900	4.04038600	-3.40383200
C	-13.30101500	5.36713000	-1.02396100
C	-14.25377200	4.48844400	-0.52137400
C	-14.41580100	3.19880200	-1.07476200
C	-13.62444900	2.79324900	-2.13985800
C	-12.63339700	3.66351500	-2.67410300
C	-12.45170400	4.95493600	-2.08844000
H	-13.19555800	6.36827200	-0.60314100
H	-14.88814400	4.80378700	0.31134800
H	-15.18273200	2.53045800	-0.67600400
H	-13.77835200	1.81701400	-2.60638800
H	-12.16464400	3.41701000	-3.63126300
B	-9.91185200	4.80652400	-6.34079000
F	-11.08879200	5.30124500	-6.87458600
F	-10.26168100	3.86664900	-5.28152300
F	-9.16046400	4.11266800	-7.28516500
F	-9.14787200	5.82801600	-5.76110800

Zero-point correction= 0.397123 (Hartree/Particle)

Thermal correction to Energy= 0.434497

Thermal correction to Enthalpy= 0.435441

Thermal correction to Gibbs Free Energy= 0.322072

Sum of electronic and zero-point Energies= -3996.394703

Sum of electronic and thermal Energies= -3996.357330

Sum of electronic and thermal Enthalpies= -3996.356385

Sum of electronic and thermal Free Energies= -3996.469755

E(B3LYPD3/Def2TZVPP) = -3998.57564096

Int3-BF_4

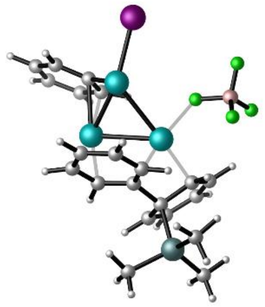

C	-7.40873100	1.11181300	-3.09513700
C	-7.19141000	2.04944900	-2.13080700
C	-6.97507600	3.47718700	-2.44096500
C	-7.21348200	3.83909200	-3.87833800
C	-7.41662700	2.80837700	-4.85660200
C	-7.53073600	1.49438700	-4.47337300
H	-7.49660800	0.05804700	-2.82272000
H	-7.11265600	1.72964900	-1.08970100
H	-6.79147400	4.77610800	-4.25822600
H	-7.52149100	3.10014300	-5.90246900
H	-7.71418000	0.72233200	-5.22478600
Ge	-4.94018900	3.72617800	-1.97041500
C	-4.85444300	3.48695000	-0.02564900
H	-5.57814700	4.15072600	0.47131700
H	-5.06871200	2.44553700	0.25515000
H	-3.84344800	3.74681900	0.32434500
C	-4.37884200	5.53248600	-2.49676900
H	-3.27956500	5.56412800	-2.43290500
H	-4.67140500	5.76576900	-3.53100700
H	-4.78935400	6.29367900	-1.81925600
C	-3.96504600	2.35981700	-2.98192500
H	-2.88353600	2.49901600	-2.83003400
H	-4.24867700	1.35260600	-2.64576100
H	-4.18543200	2.45365700	-4.05586800
C	-9.15186600	4.93507400	0.46072400
C	-8.56875600	4.00428000	-0.43412600
C	-7.81953200	4.46934900	-1.58732000
C	-7.68387900	5.88270100	-1.74712700
C	-8.28107800	6.78111000	-0.84629300
C	-9.02247900	6.32168400	0.23984700

H	-9.59564500	4.57614100	1.39243300
H	-8.46511800	2.96586600	-0.11341700
H	-7.04195200	6.28825000	-2.52857400
H	-8.14911300	7.85336600	-1.00600000
H	-9.47283600	7.02656800	0.94128900
I	-13.02286300	7.63786400	-3.74036300
Pd	-11.39862700	6.04753400	-2.40557600
Pd	-10.66685300	3.79496000	-1.11855100
Pd	-9.10427900	4.56810100	-3.37794000
C	-13.60475600	4.87685000	-0.84379300
C	-14.36453800	3.80983500	-0.37651200
C	-14.19714100	2.51337900	-0.90875500
C	-13.27852600	2.29117800	-1.92517100
C	-12.48436800	3.36001600	-2.42512700
C	-12.62517700	4.65905300	-1.84895900
H	-13.74897400	5.87763900	-0.43216800
H	-15.10015000	3.97994600	0.41420200
H	-14.81192700	1.69050500	-0.53617600
H	-13.18331200	1.30264200	-2.38135500
H	-11.93097700	3.21661700	-3.35788300
B	-9.44011300	5.21794200	-6.50177200
F	-10.30495400	6.09855400	-7.12860200
F	-10.15360300	4.60358800	-5.38877700
F	-9.02829600	4.19899000	-7.35588300
F	-8.33162000	5.89158800	-5.97219900

Zero-point correction= 0.398427 (Hartree/Particle)
Thermal correction to Energy= 0.436225
Thermal correction to Enthalpy= 0.437169
Thermal correction to Gibbs Free Energy= 0.321217
Sum of electronic and zero-point Energies= -3996.412346
Sum of electronic and thermal Energies= -3996.374548
Sum of electronic and thermal Enthalpies= -3996.373604
Sum of electronic and thermal Free Energies= -3996.489556
E(B3LYPD3/Def2TZVPP) = -3998.59475919

8 NMR Spectra

Triethyl(4-fluorophenyl)germane

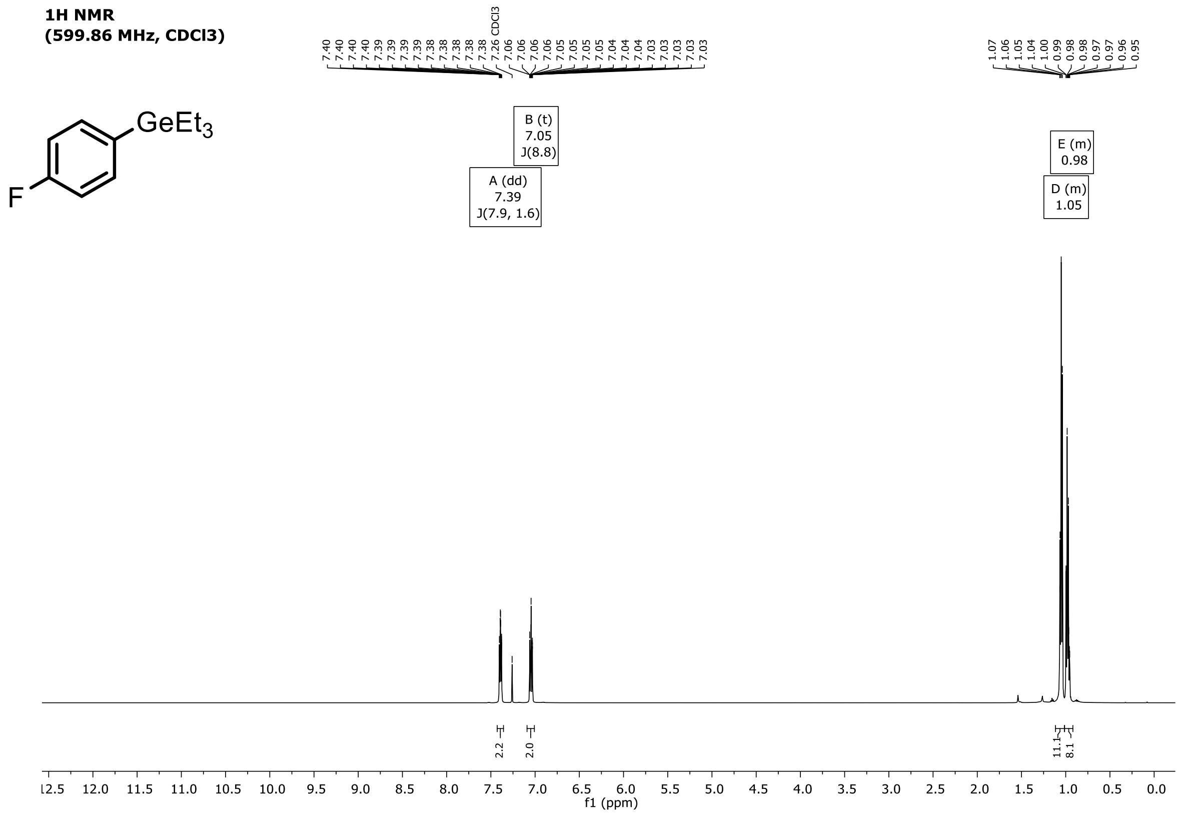

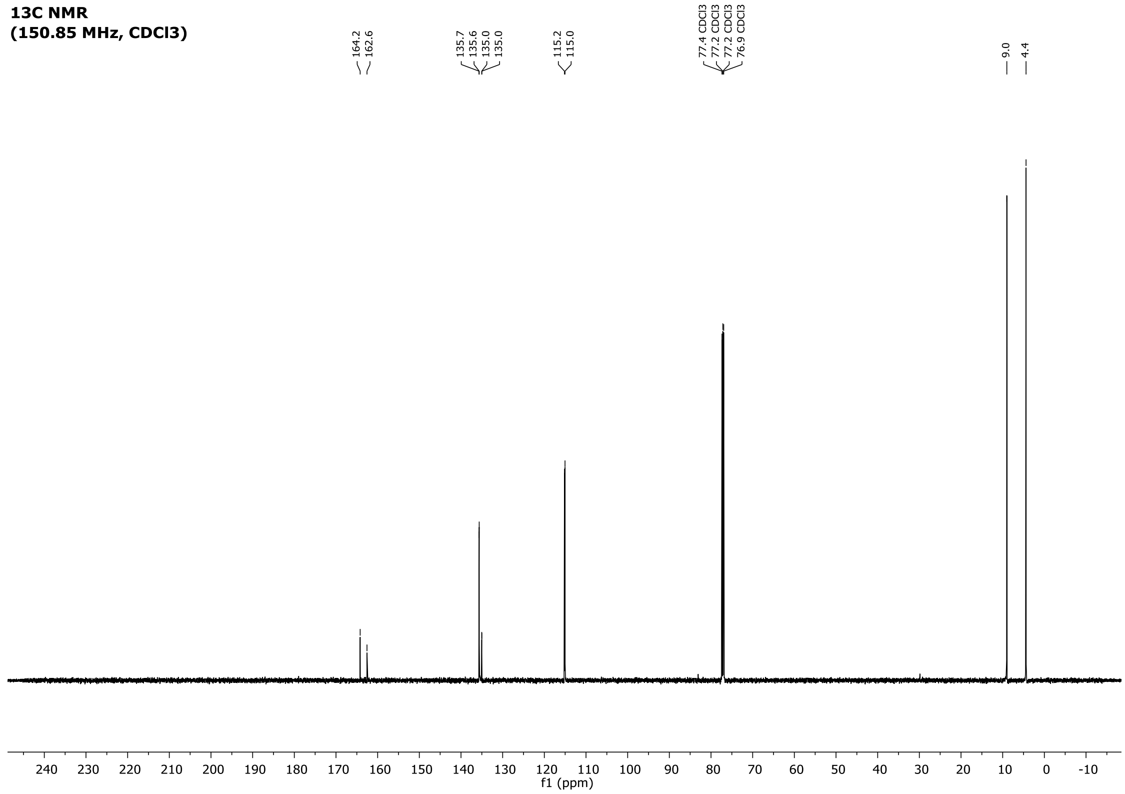

19F NMR
(564.38 MHz, CDCl3)

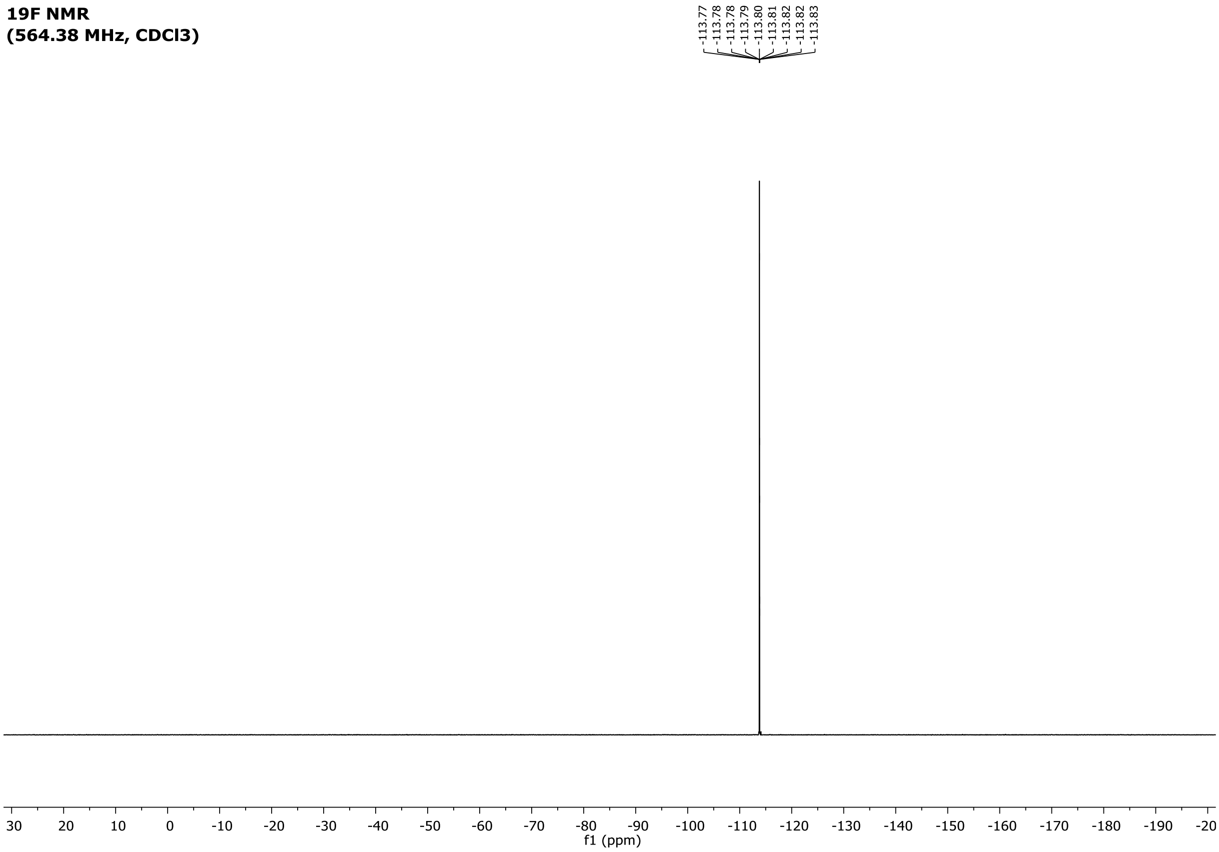

Triethyl(4-chlorophenyl)germane

1H NMR
(599.86 MHz, CDCl3)

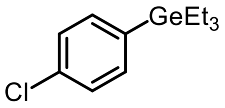

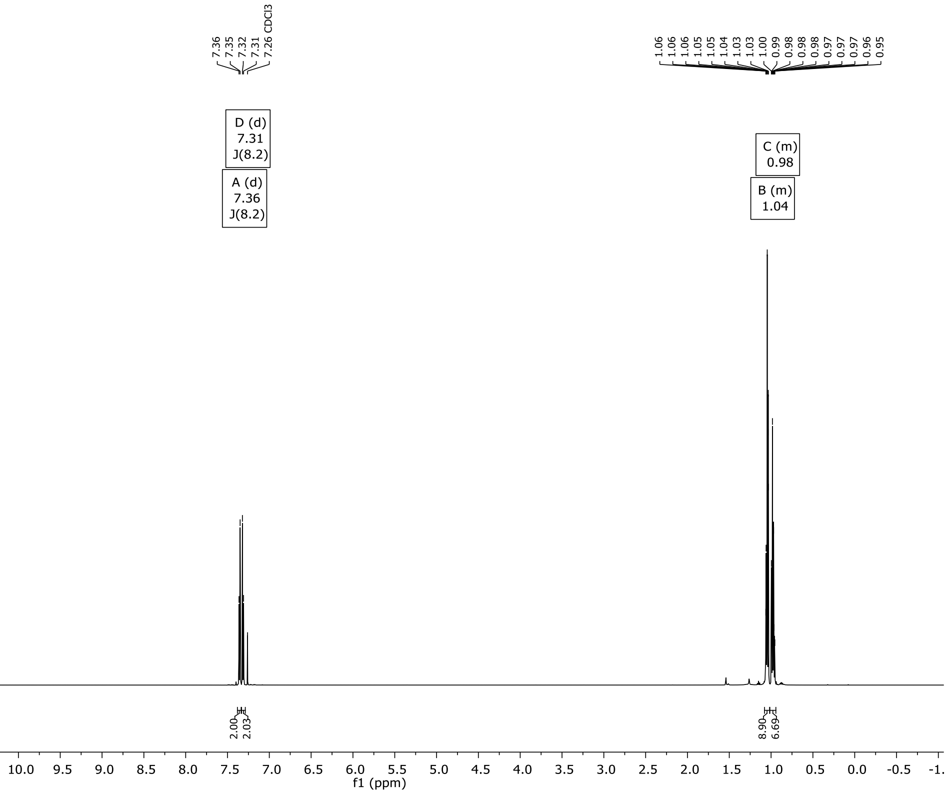

13C NMR
(150.85 MHz, CDCl3)

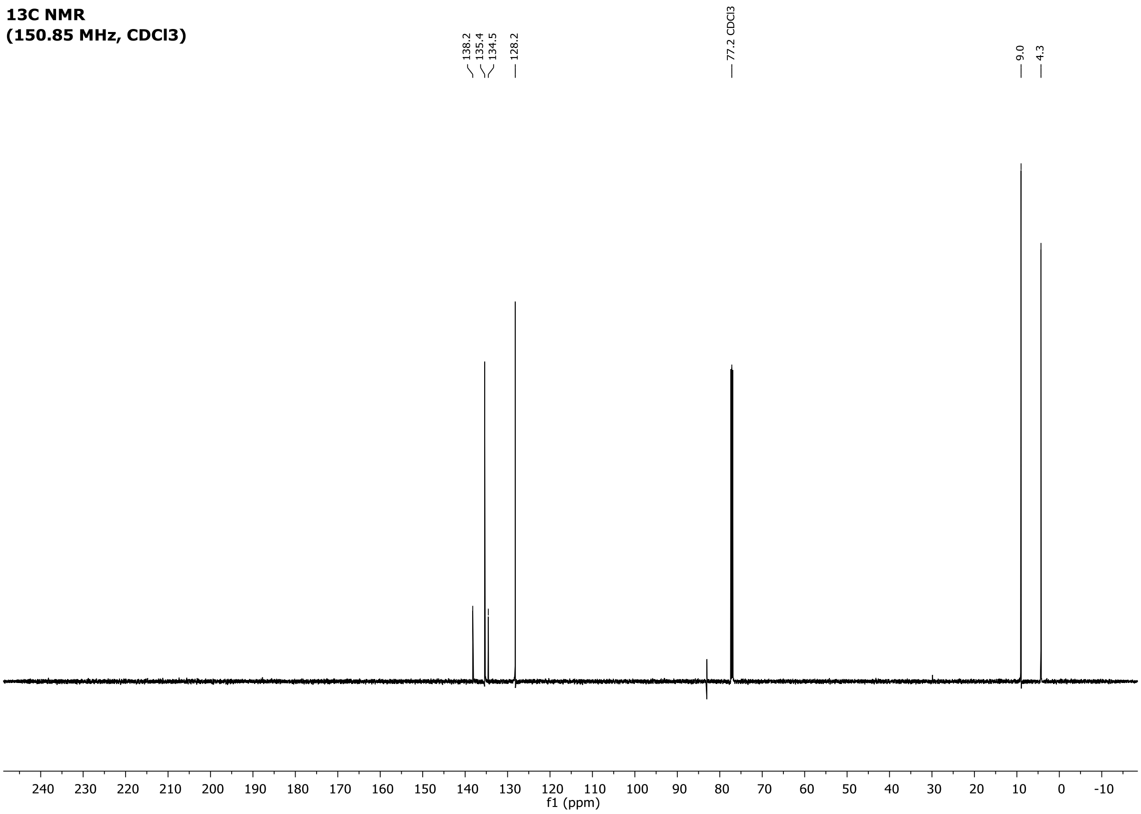

Triethyl(4-methoxyphenyl)germane

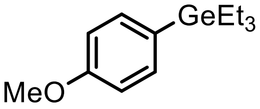

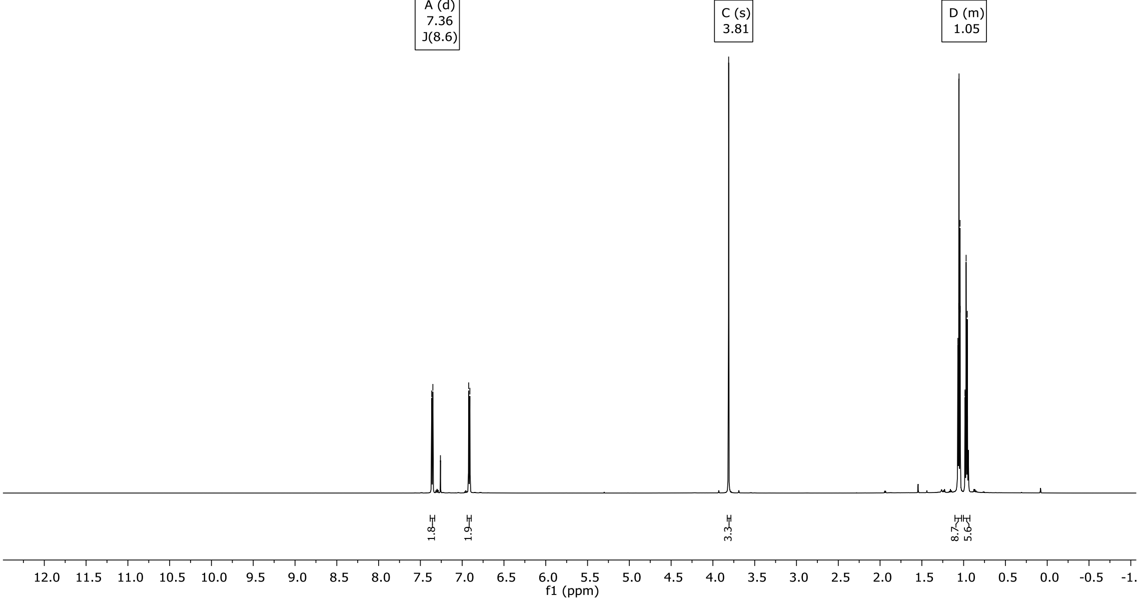

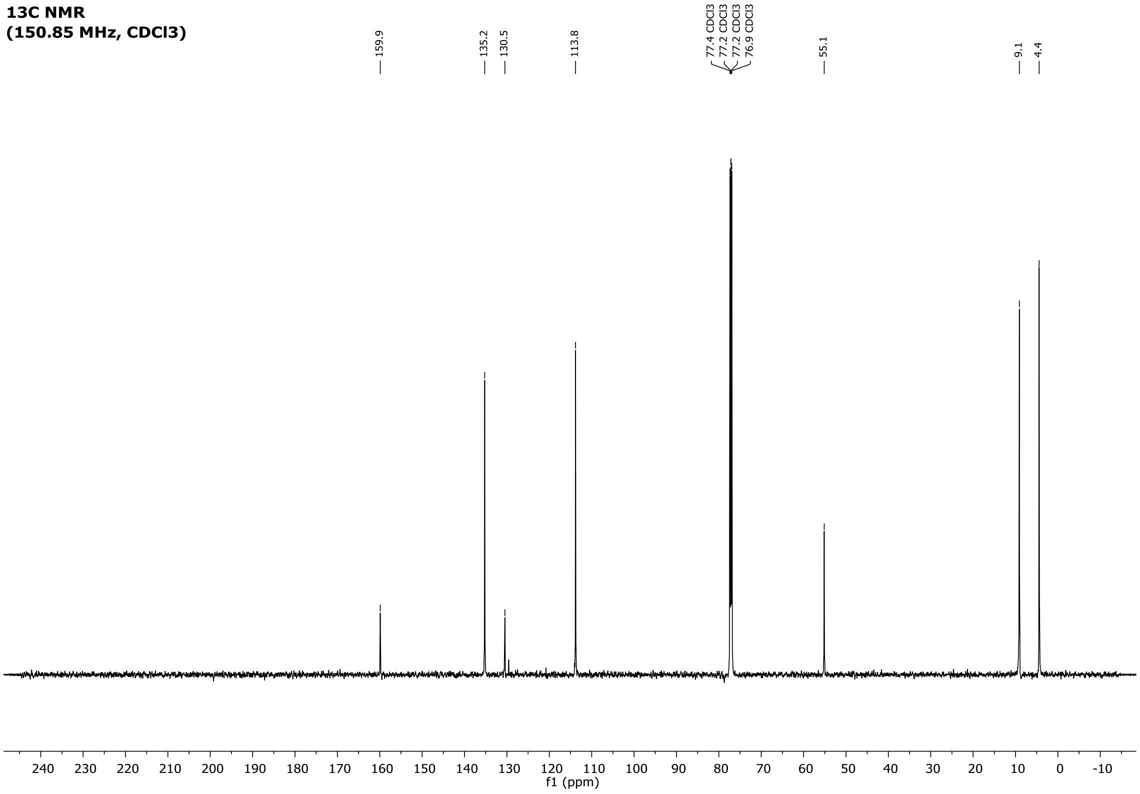

Triethyl(*p*-tolyl)germane

1H NMR
(599.86 MHz, CDCl3)

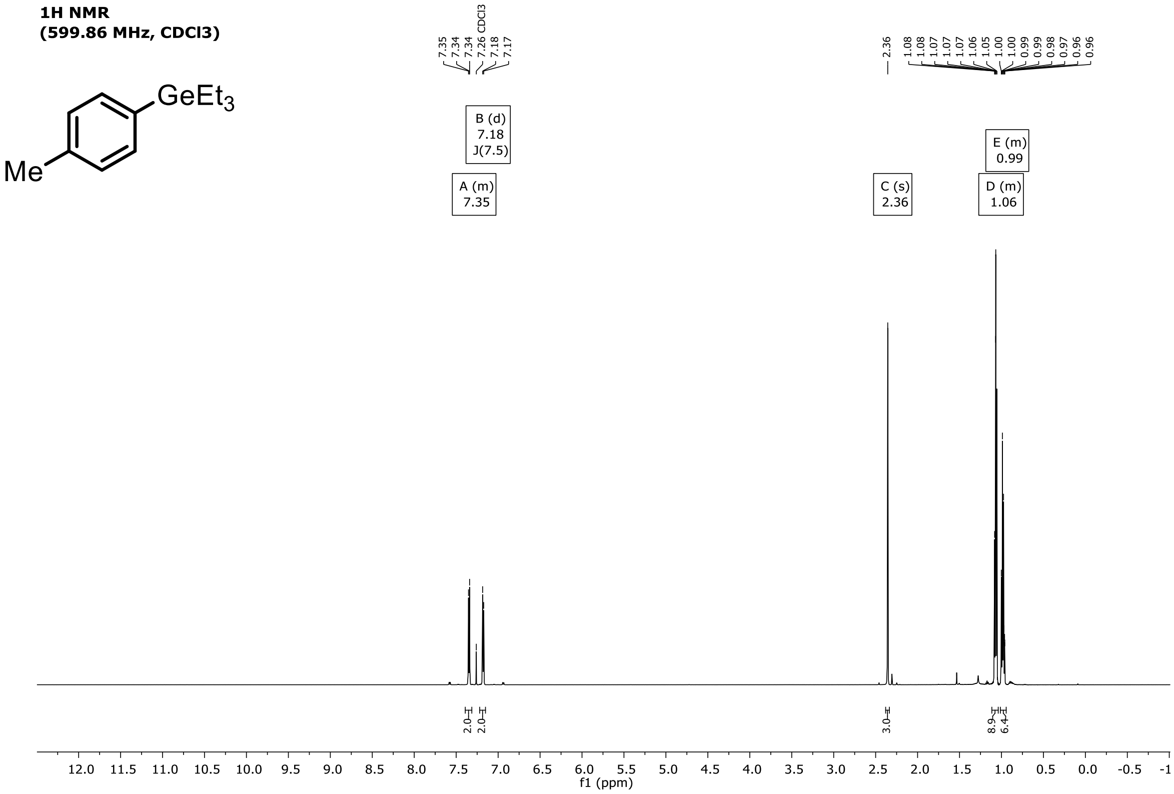

13C NMR
(150.85 MHz, CDCl3)

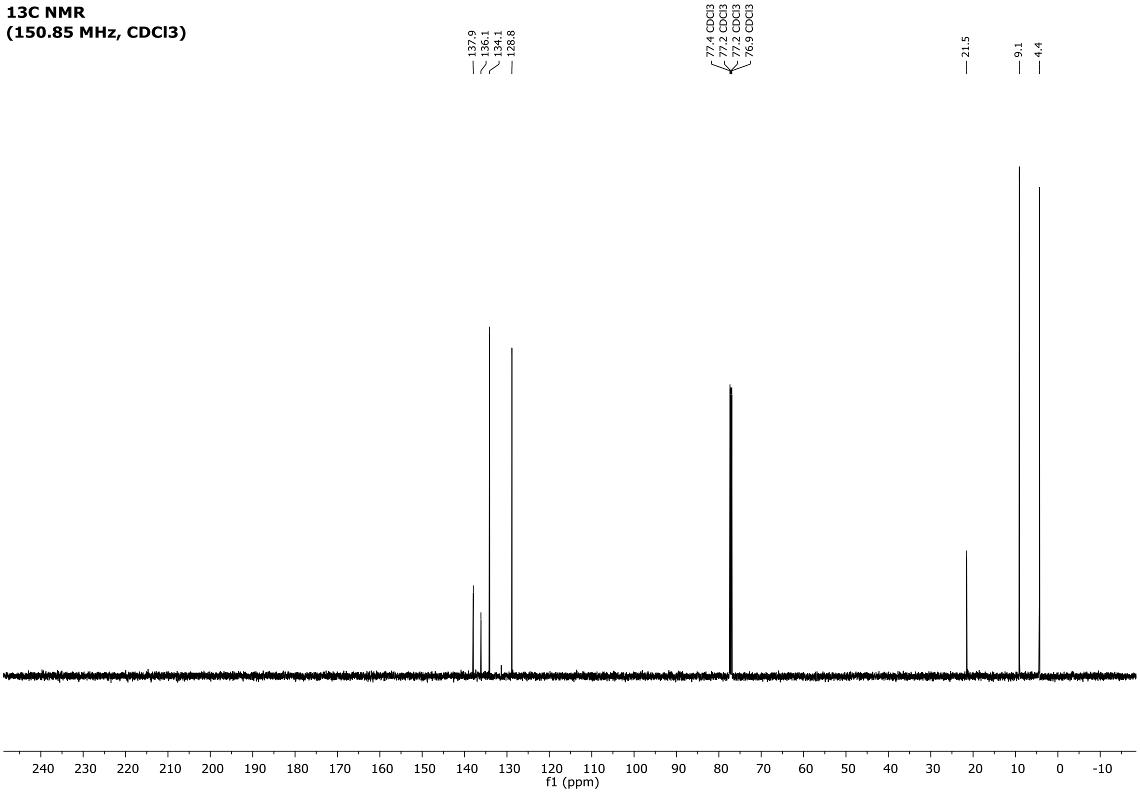

Triethyl(3-fluorophenyl)germane

1H NMR
399.97 MHz, CDCl3

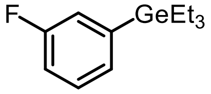

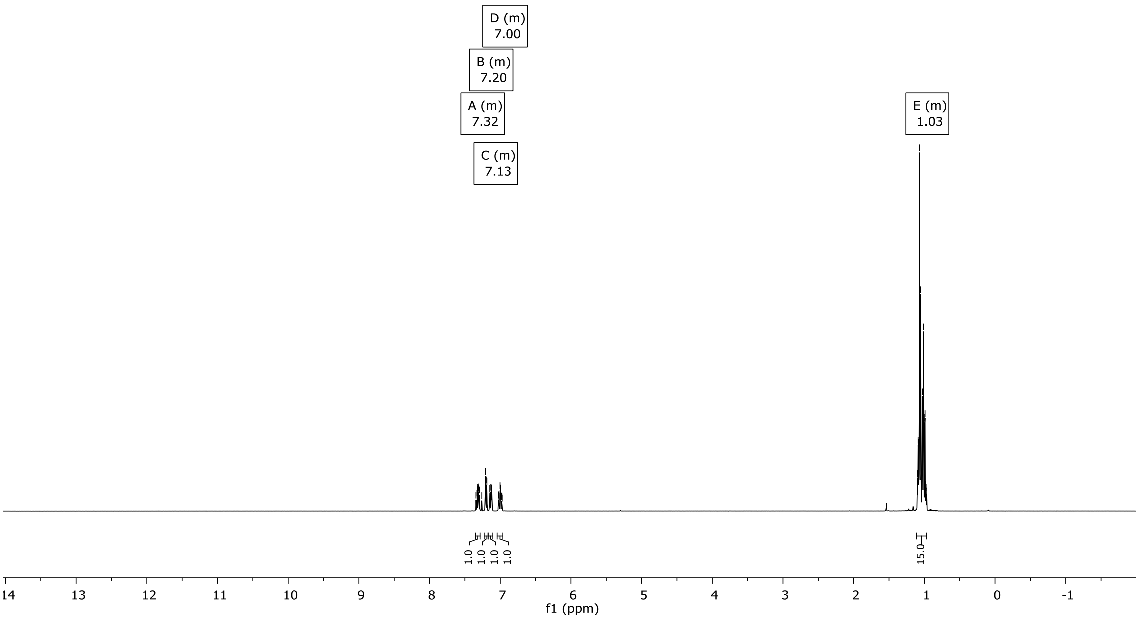

13C NMR
100.58 MHz, CDCl3

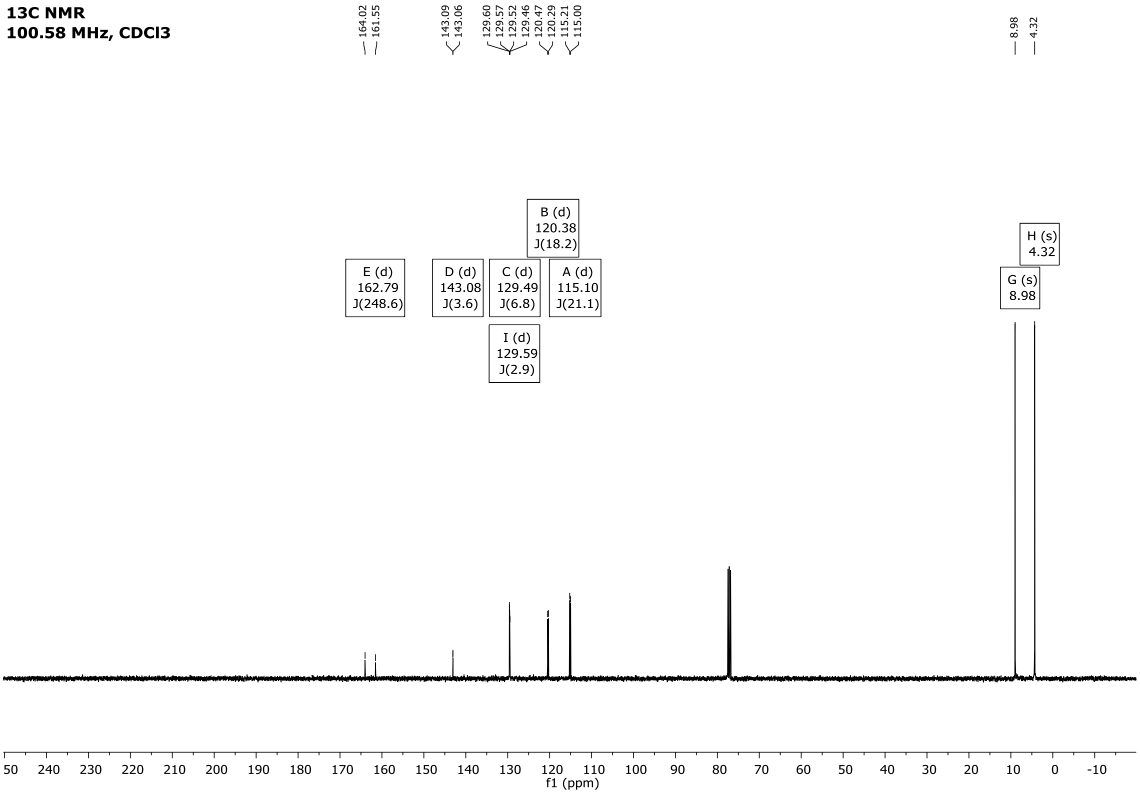

19F NMR
376.33 MHz, CDCl3

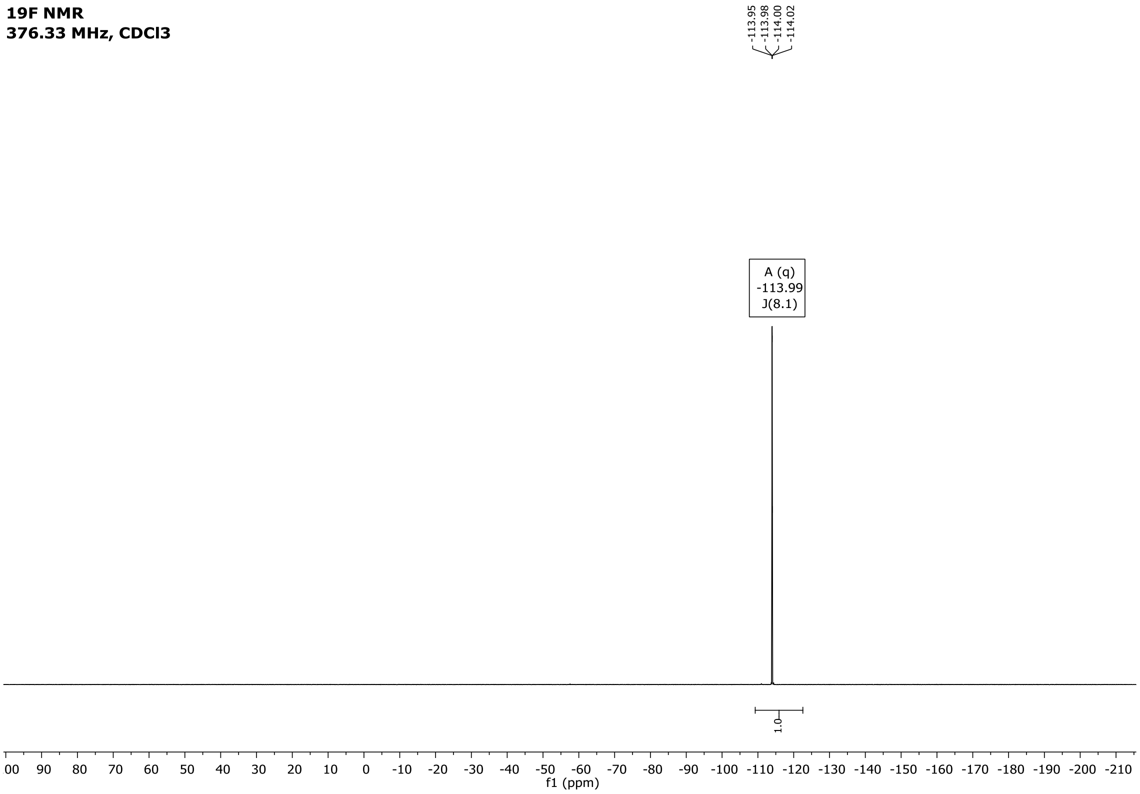

Triethyl(2-fluorophenyl)germane

13C NMR
150.85 MHz, CDCl3

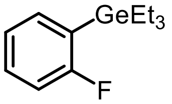

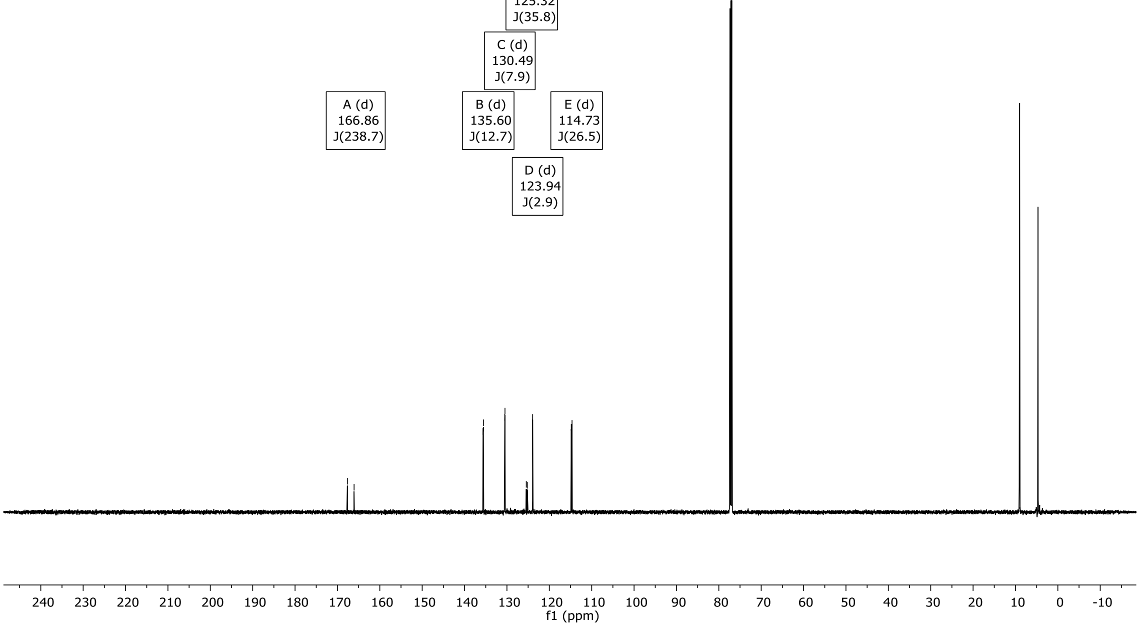

19F NMR
564.40 MHz, CDCl3

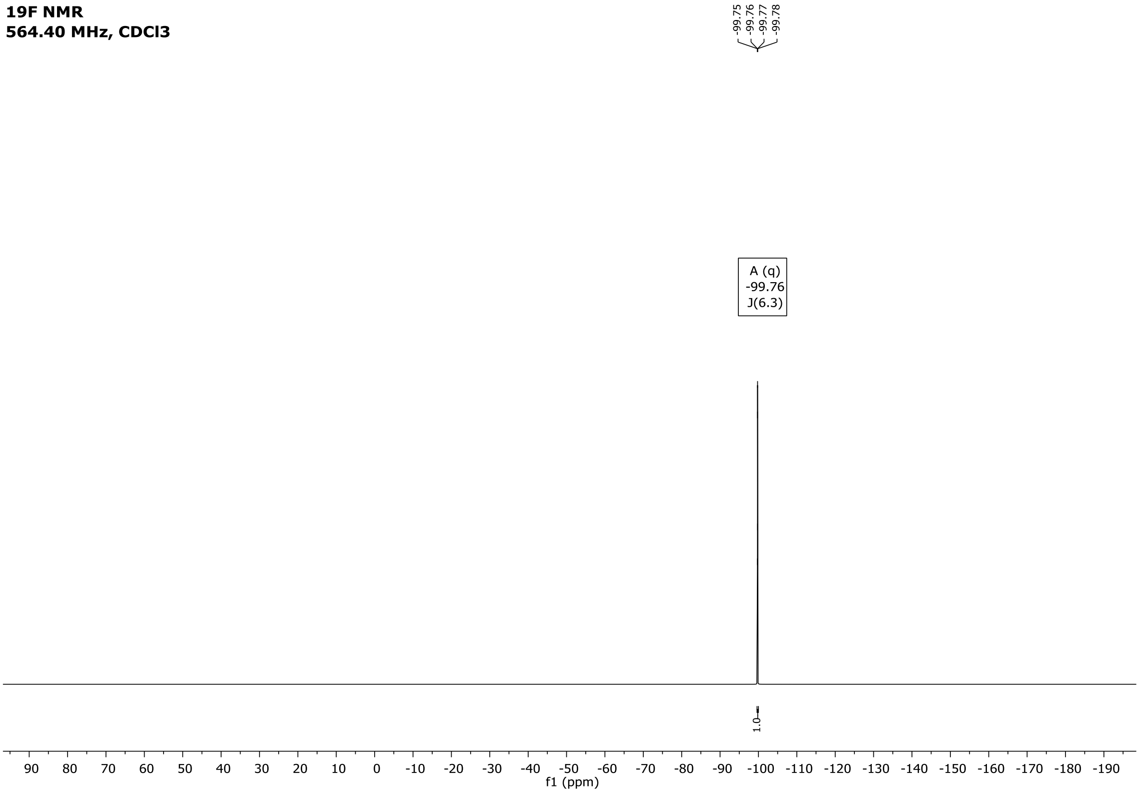

Triethyl(furan-2-yl)germane

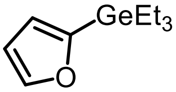

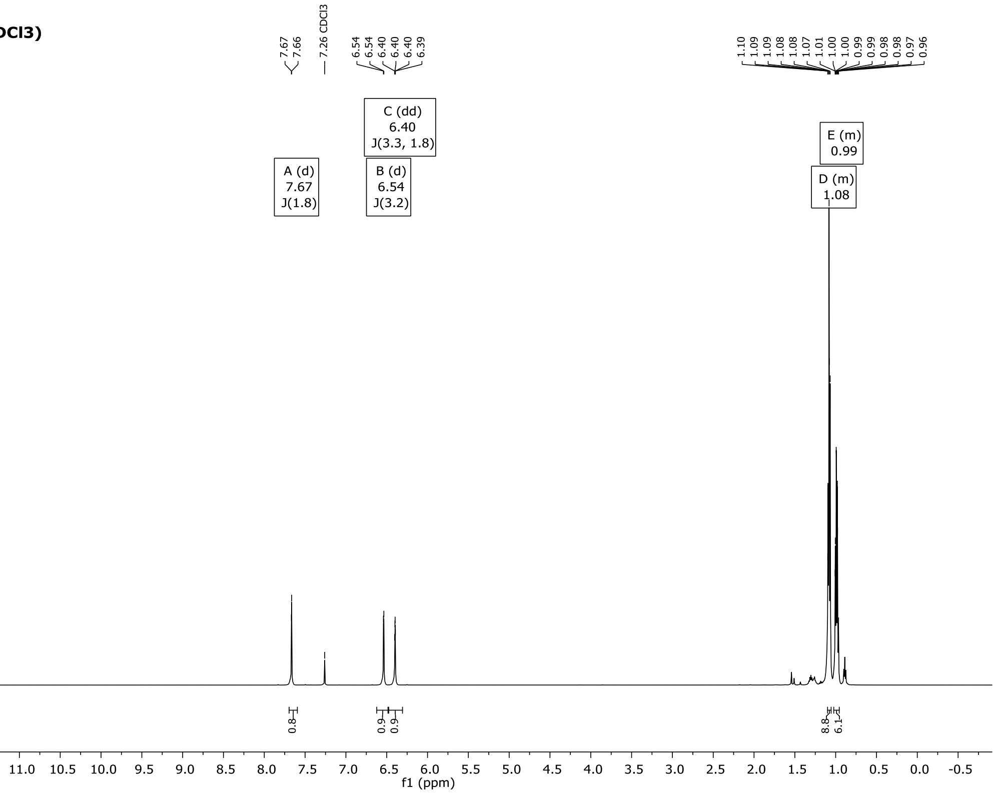

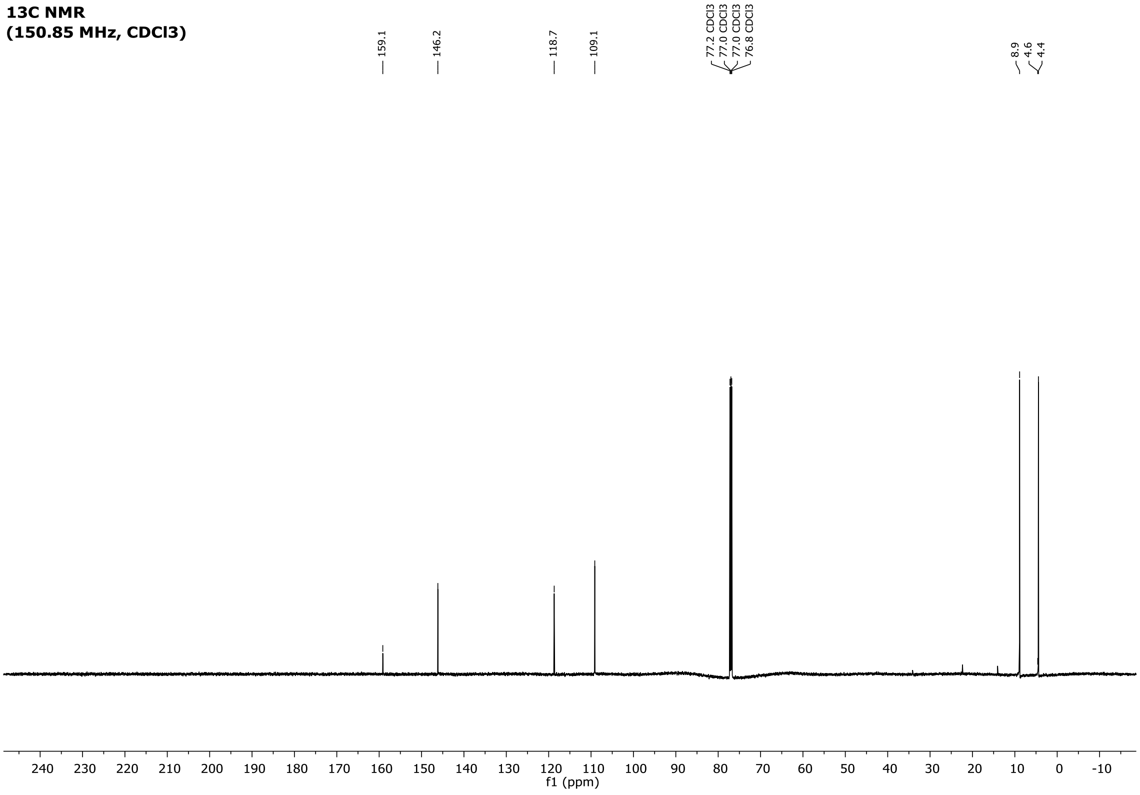

5-Bromo-2-(triethylgermyl)pyridine

1H NMR
(599.86 MHz, CDCl3)

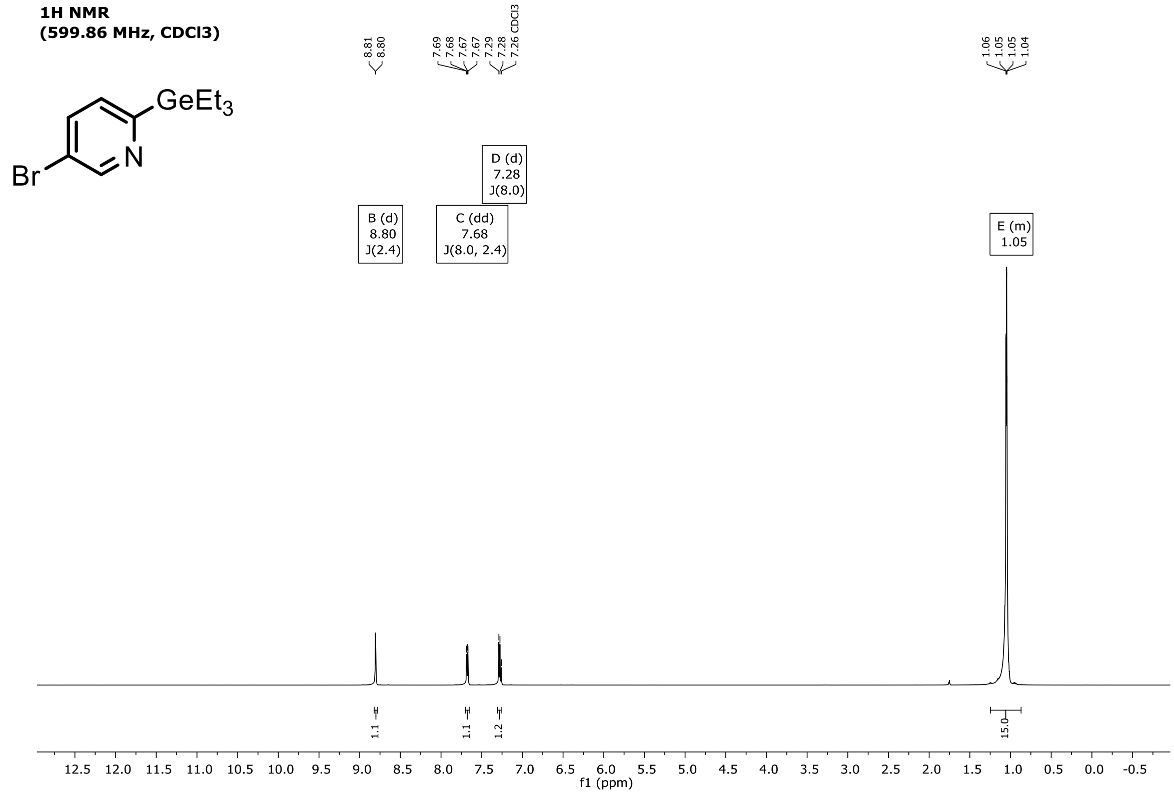

13C NMR
(150.85 MHz, CDCl3)

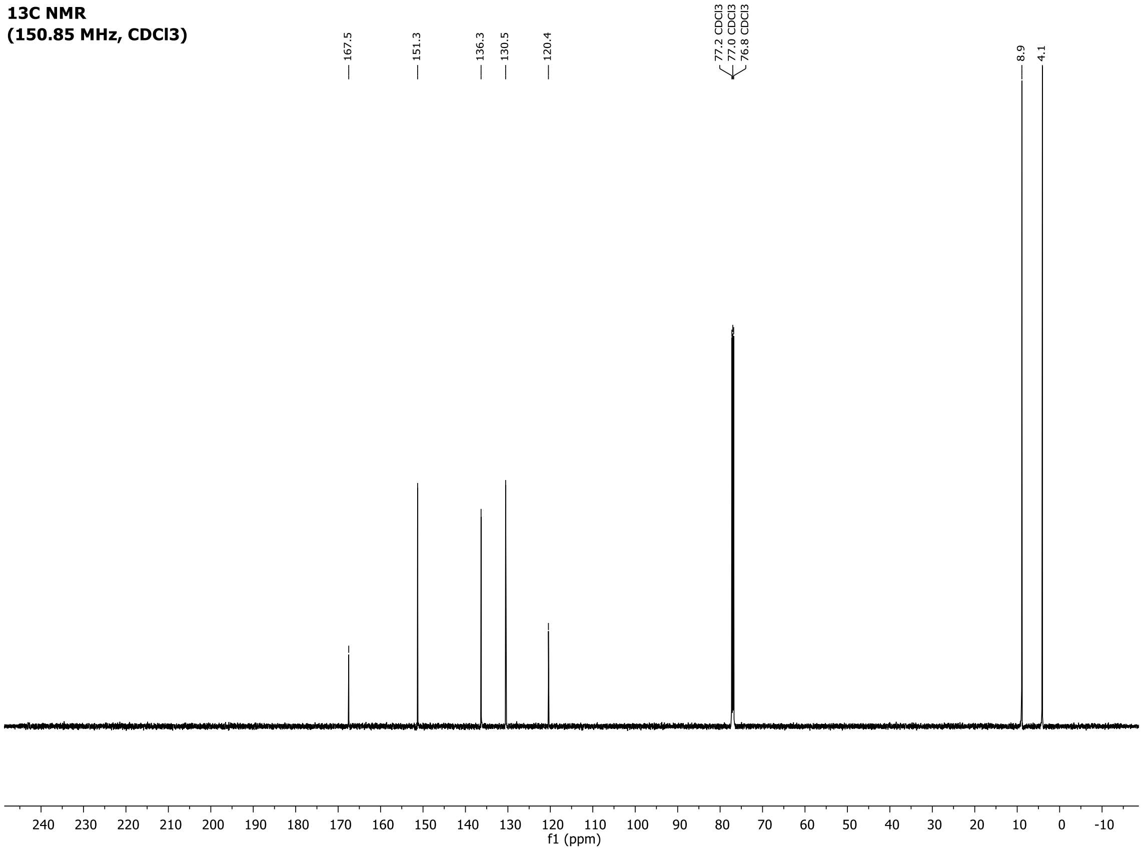

3-Fluoro-2-(triethylgermyl)pyridine

1H NMR
(599.86 MHz, CDCl3)

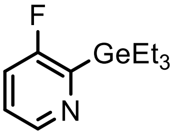

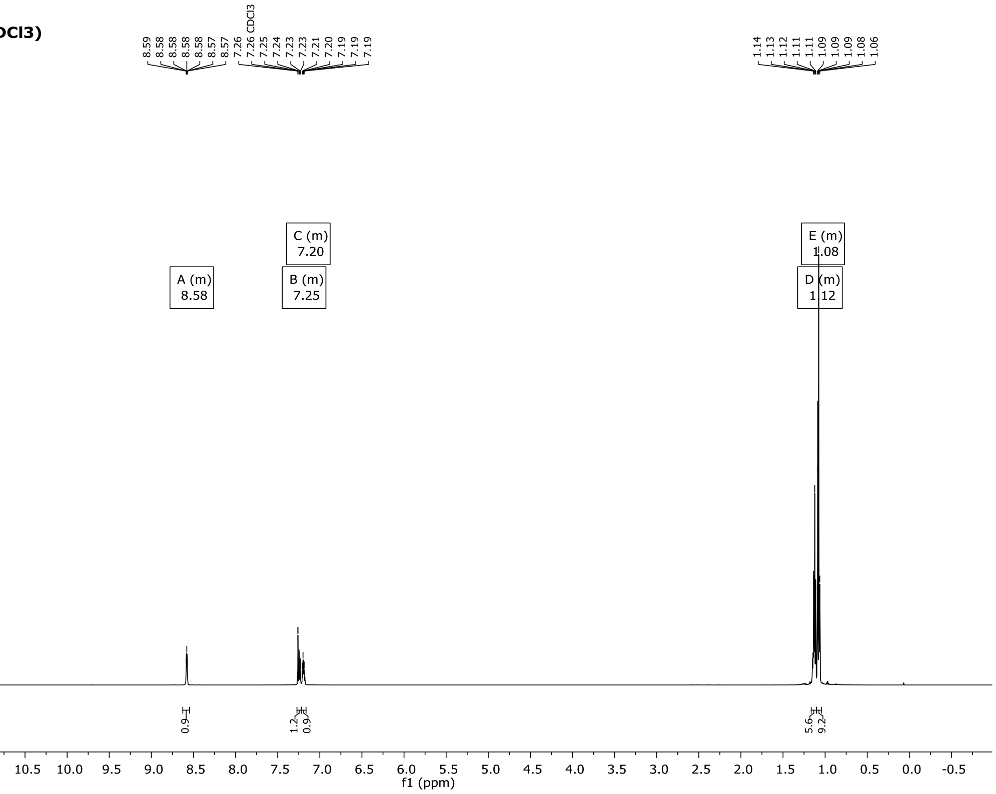

13C NMR
(150.85 MHz, CDCl3)

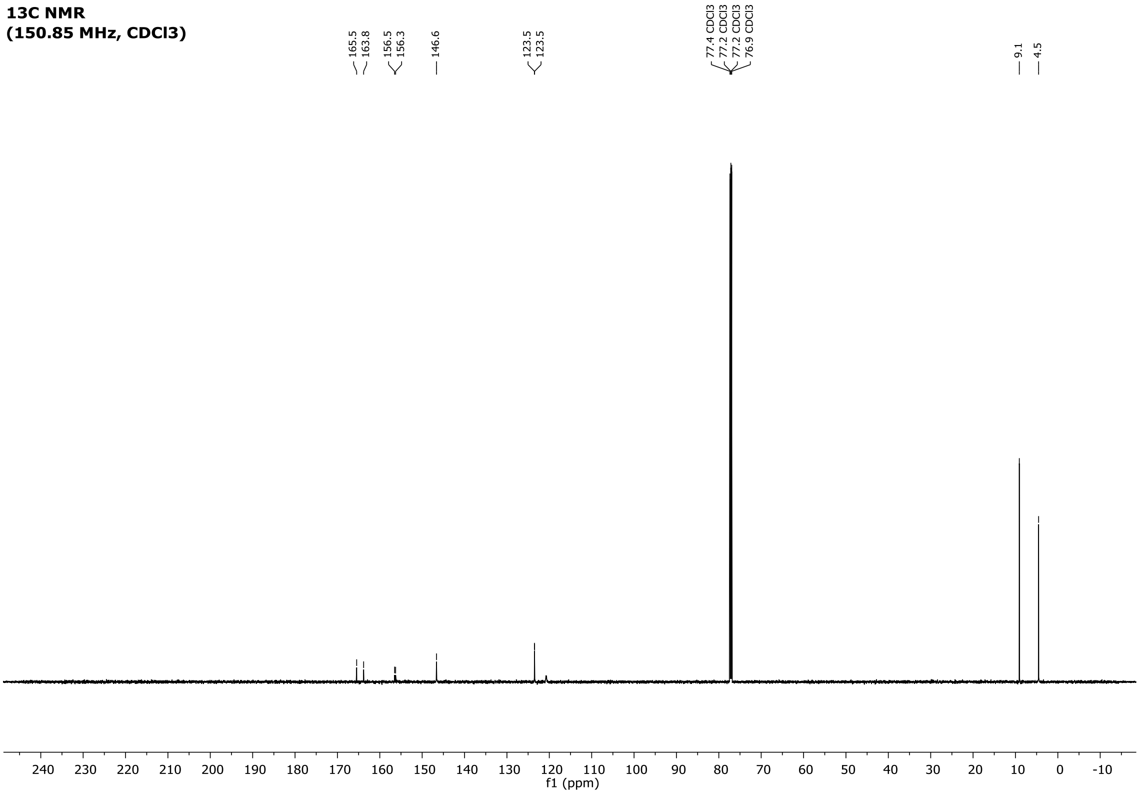

19F NMR
(564.38 MHz, CDCl3)

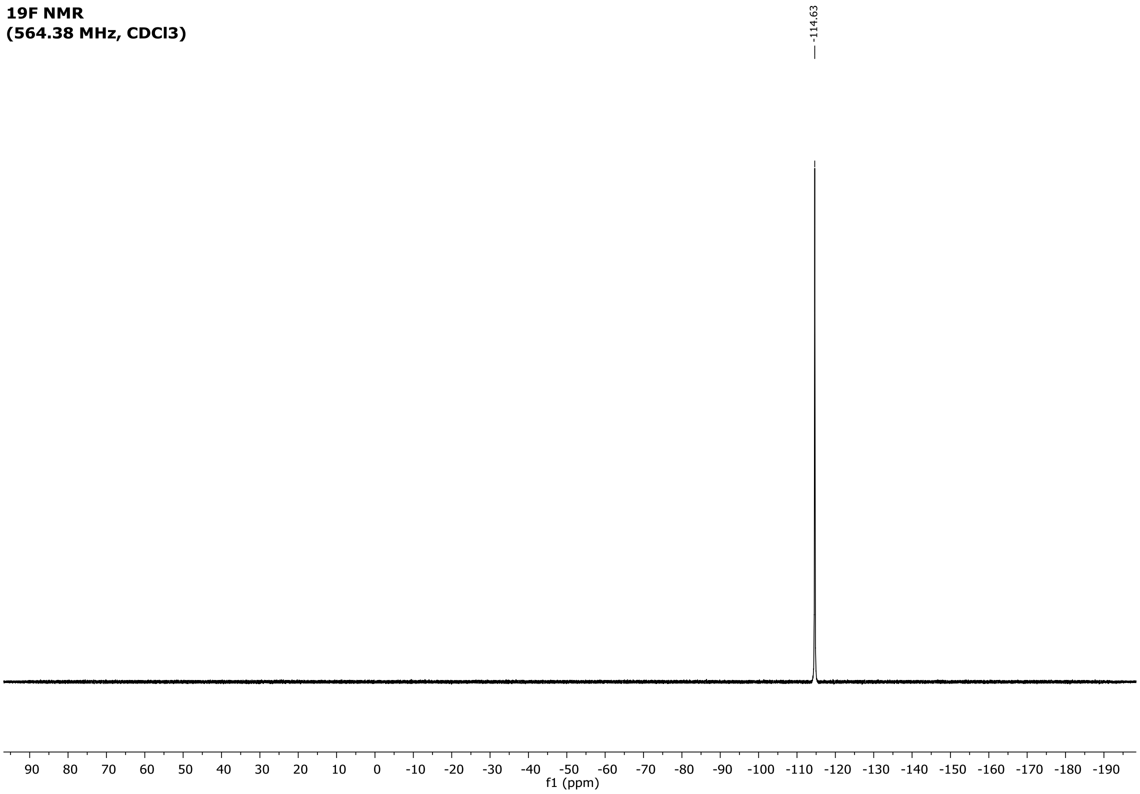

2-(Triethylgermyl)pyridine

1H NMR
(599.86 MHz, CDCl3)

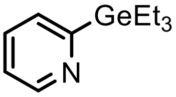

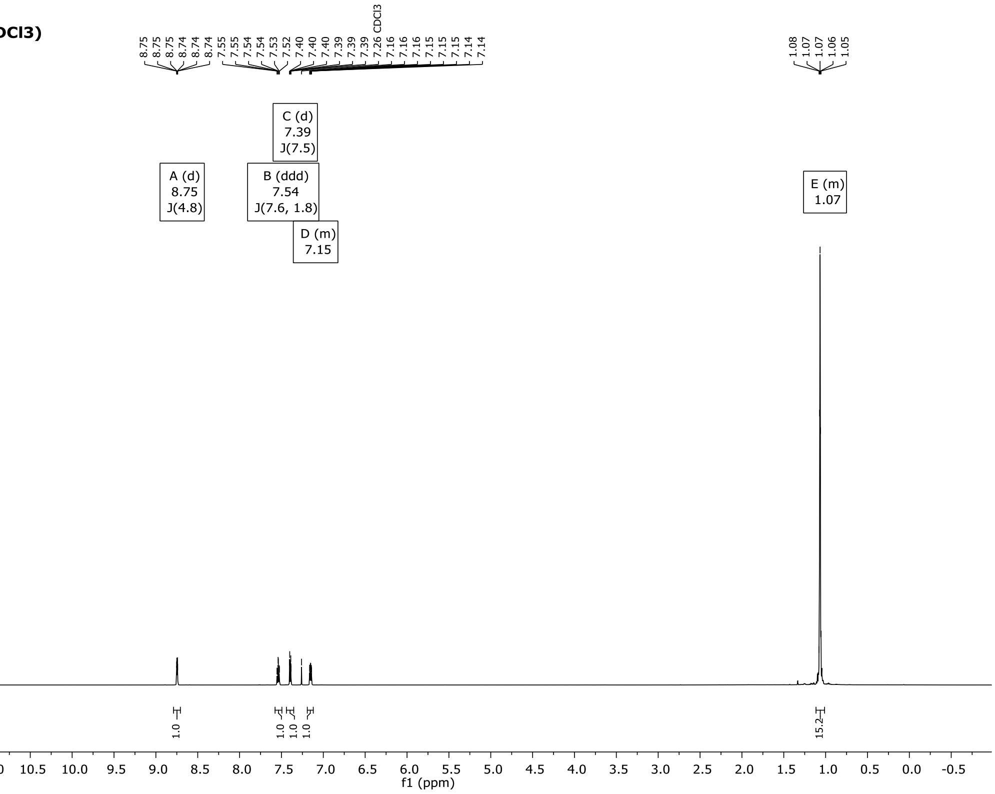

13C NMR
(150.85 MHz, CDCl3)

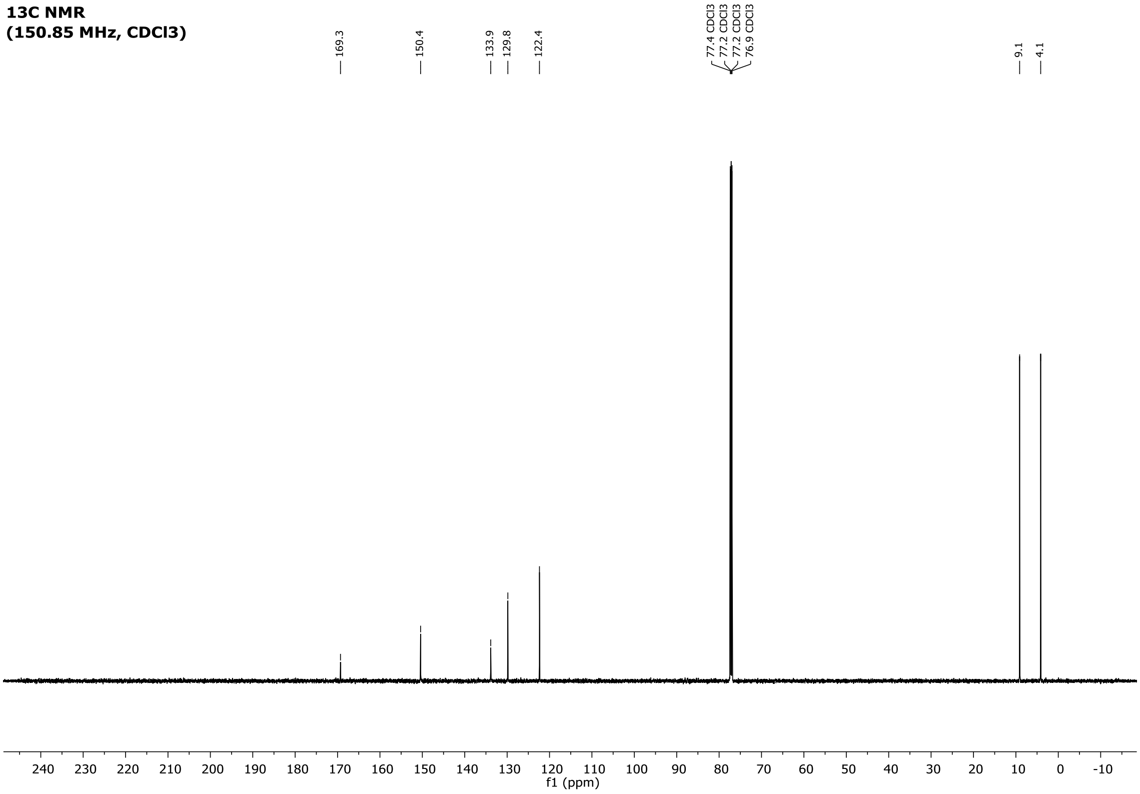

Triethyl(thiophen-2-yl)germane

1H NMR
(599.86 MHz, CDCl3)

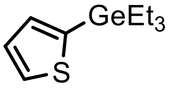

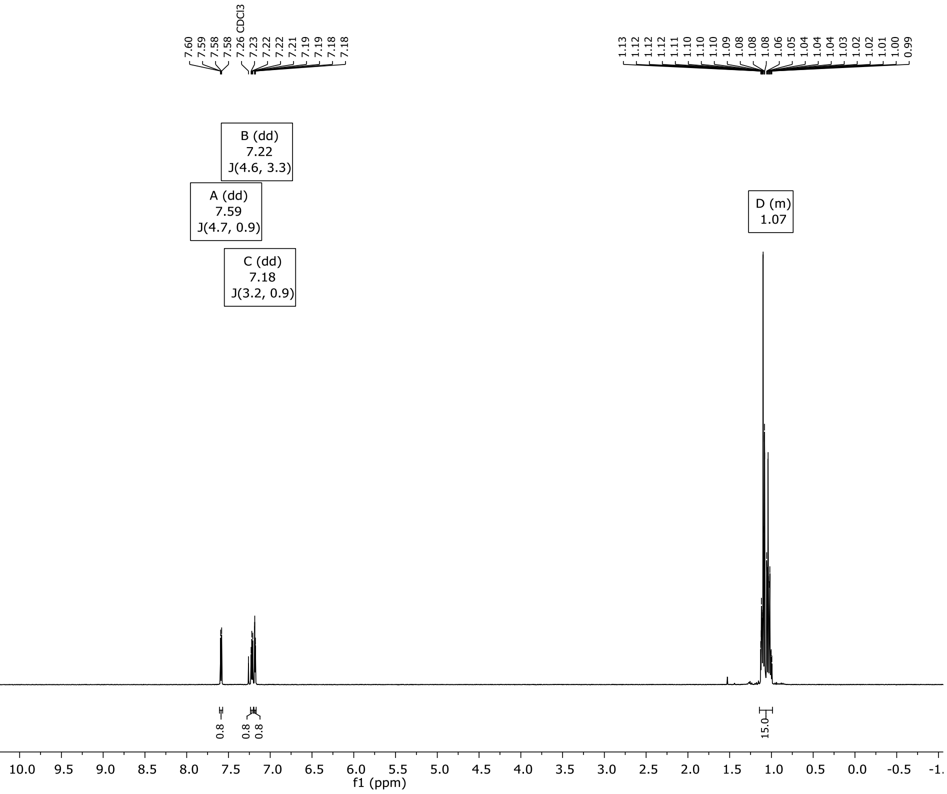

13C NMR
(100.58 MHz, CDCl3)

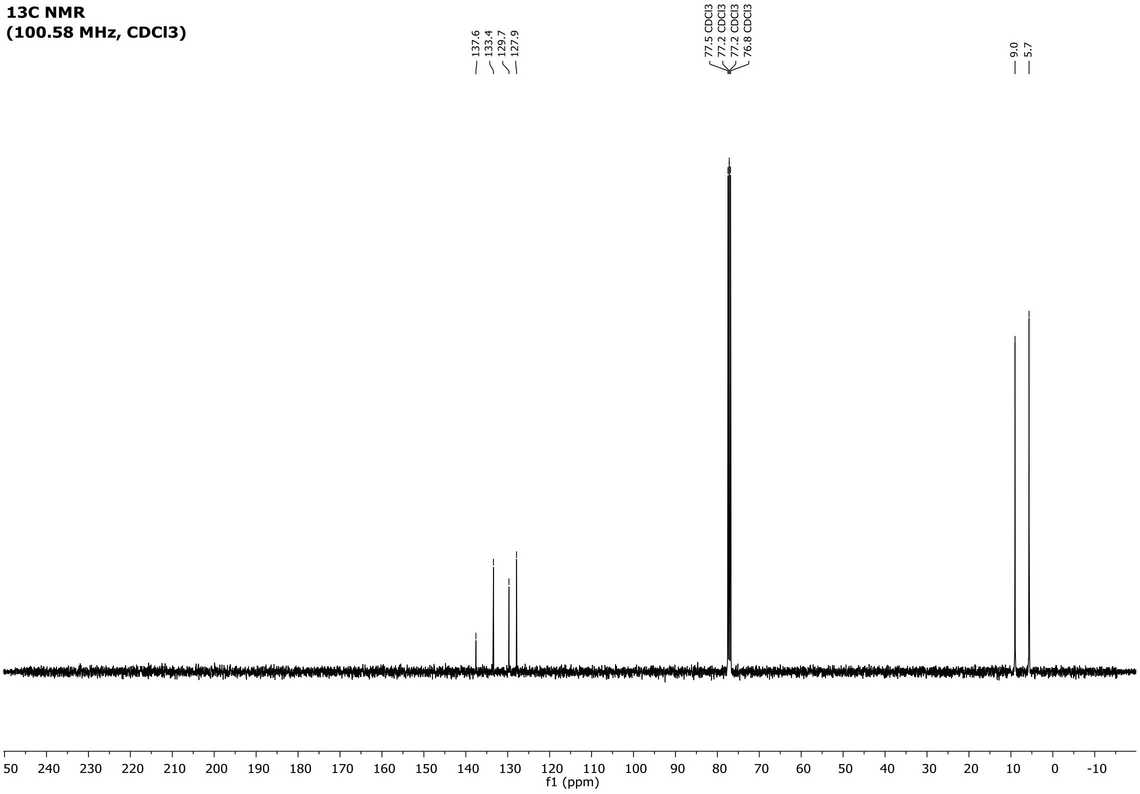

Triethyl(thiophen-3-yl)germane

19F NMR
(599.86 MHz, CDCl3)

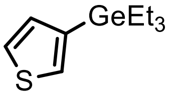

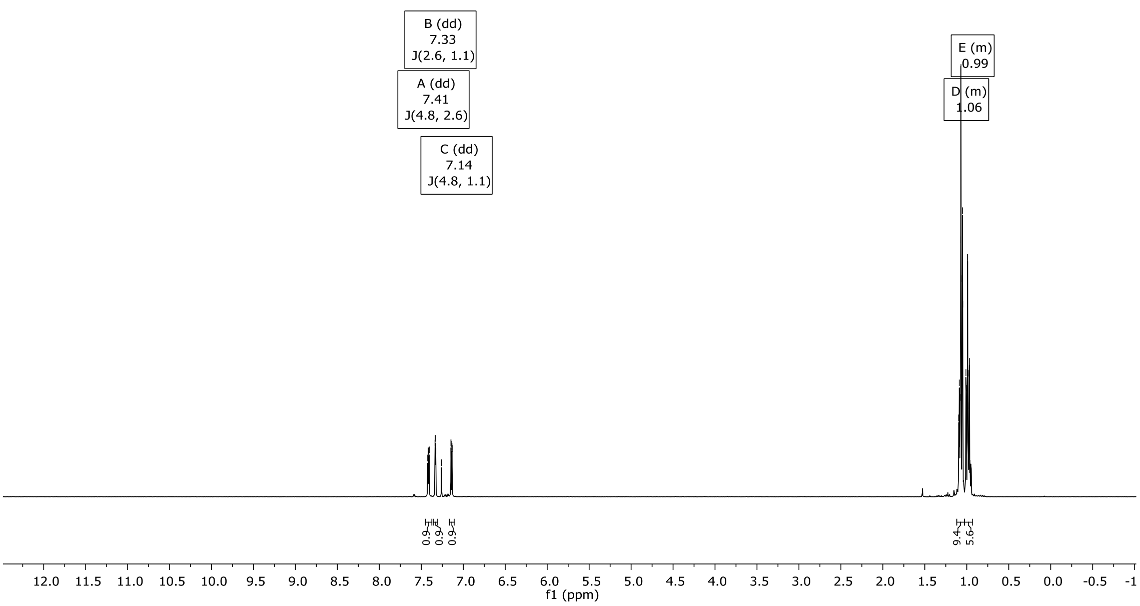

13C NMR
(100.58 MHz, CDCl3)

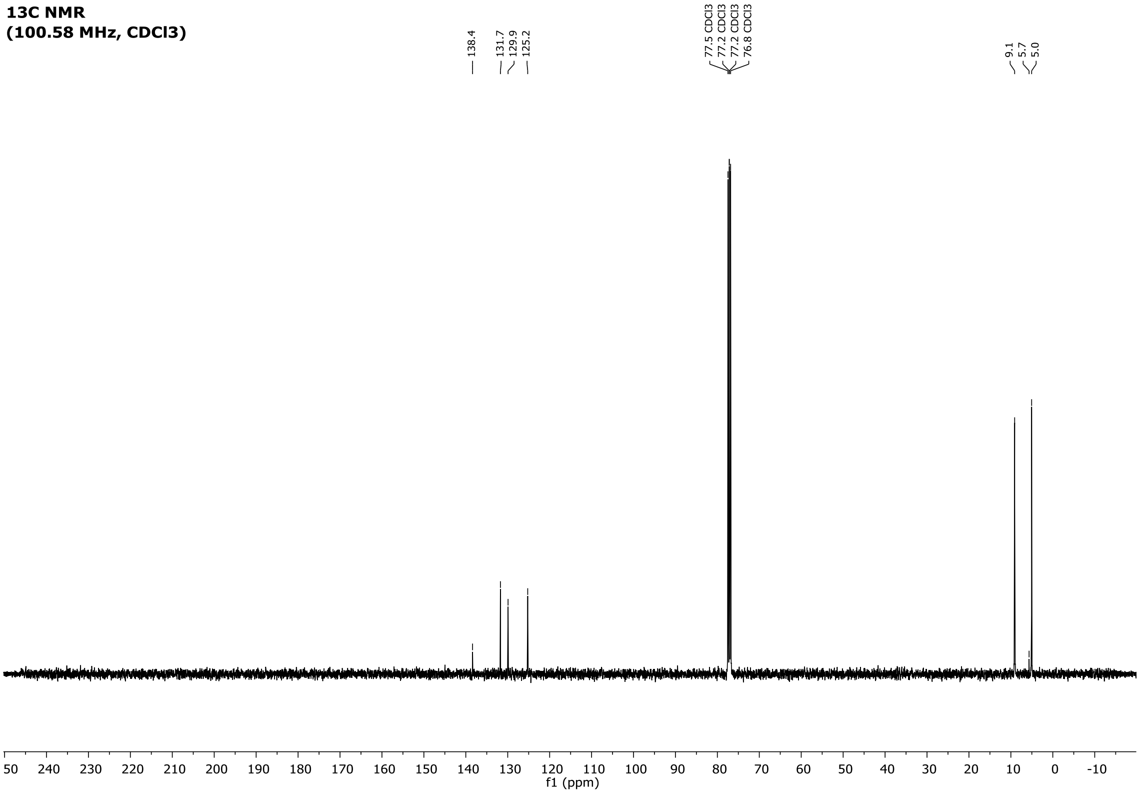

Triethyl(perfluorophenyl)germane

1H NMR
(599.86 MHz, CDCl3)

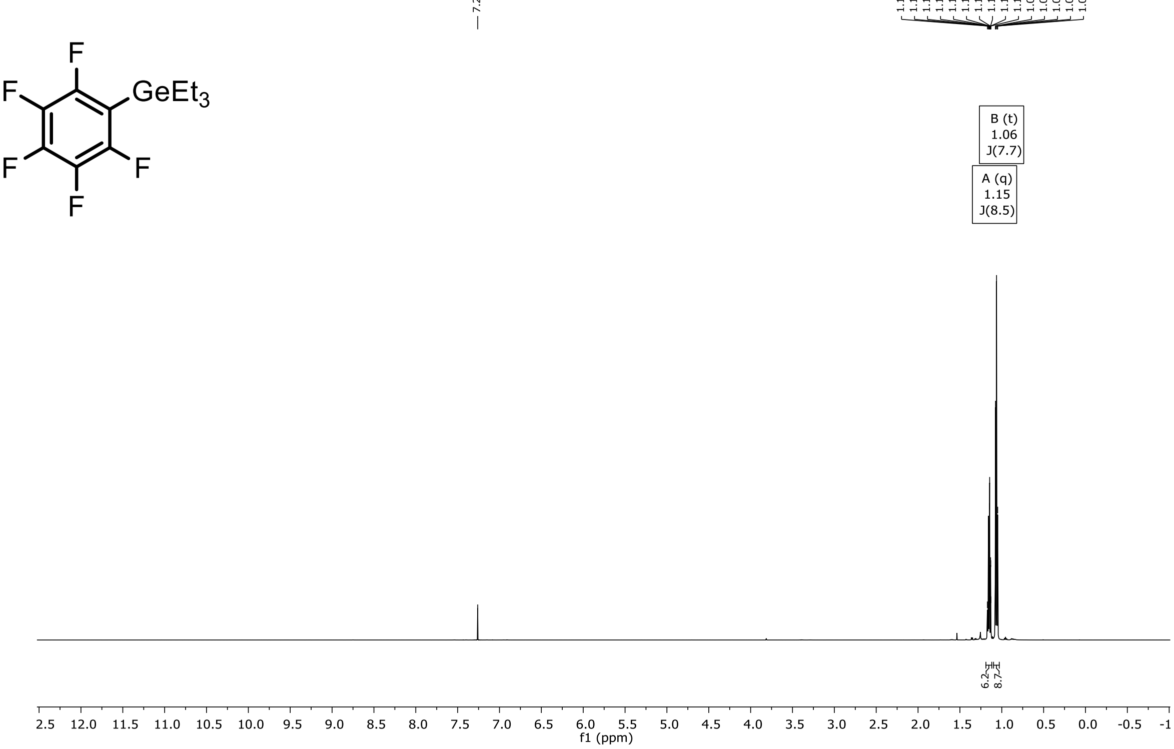

13C NMR
150.85 MHz, CDCl3

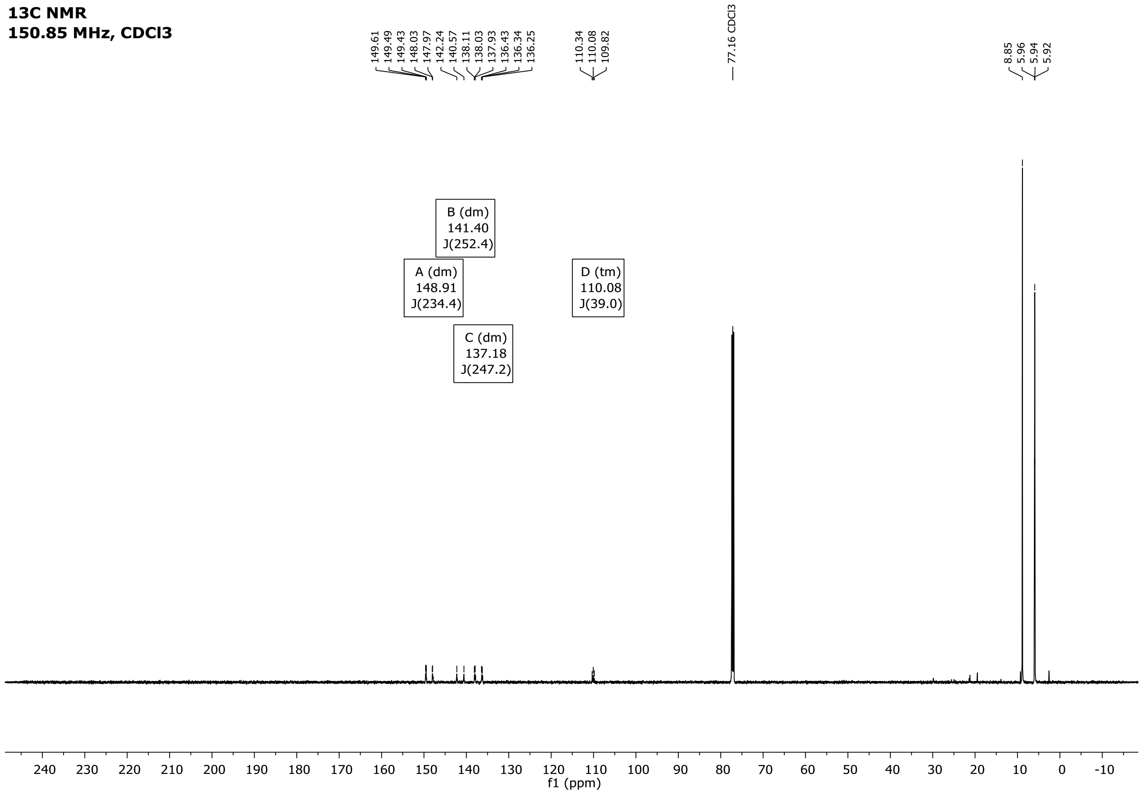

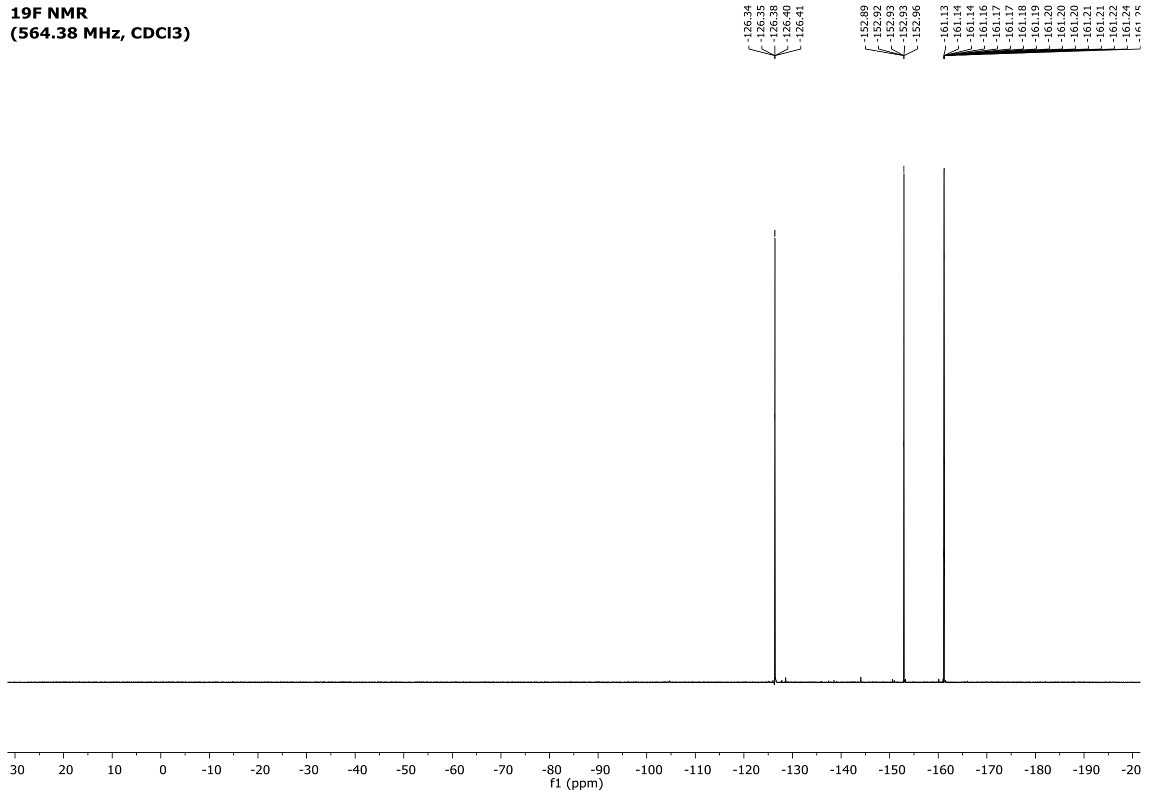
19F NMR
(564.38 MHz, CDCl3)
f1 (ppm)

‘

4-(Triethylgermyl)phenyl trifluoromethanesulfonate

1H NMR
(599.86 MHz, CDCl3)

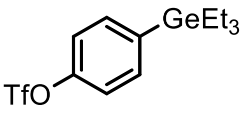

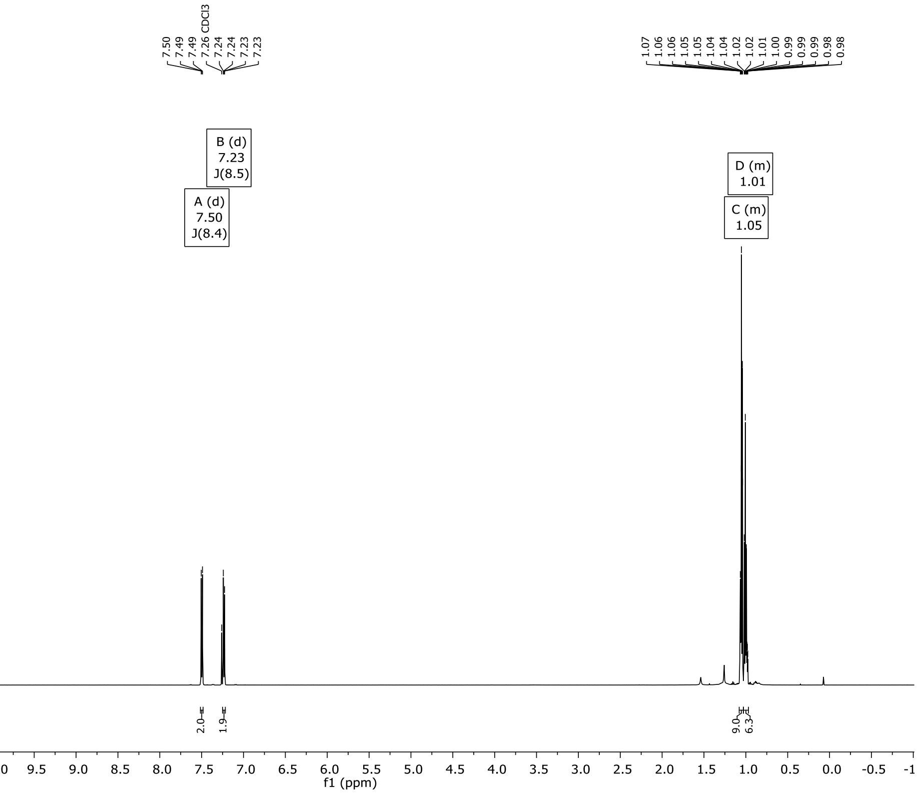

13C NMR
(150.85 MHz, CDCl3)

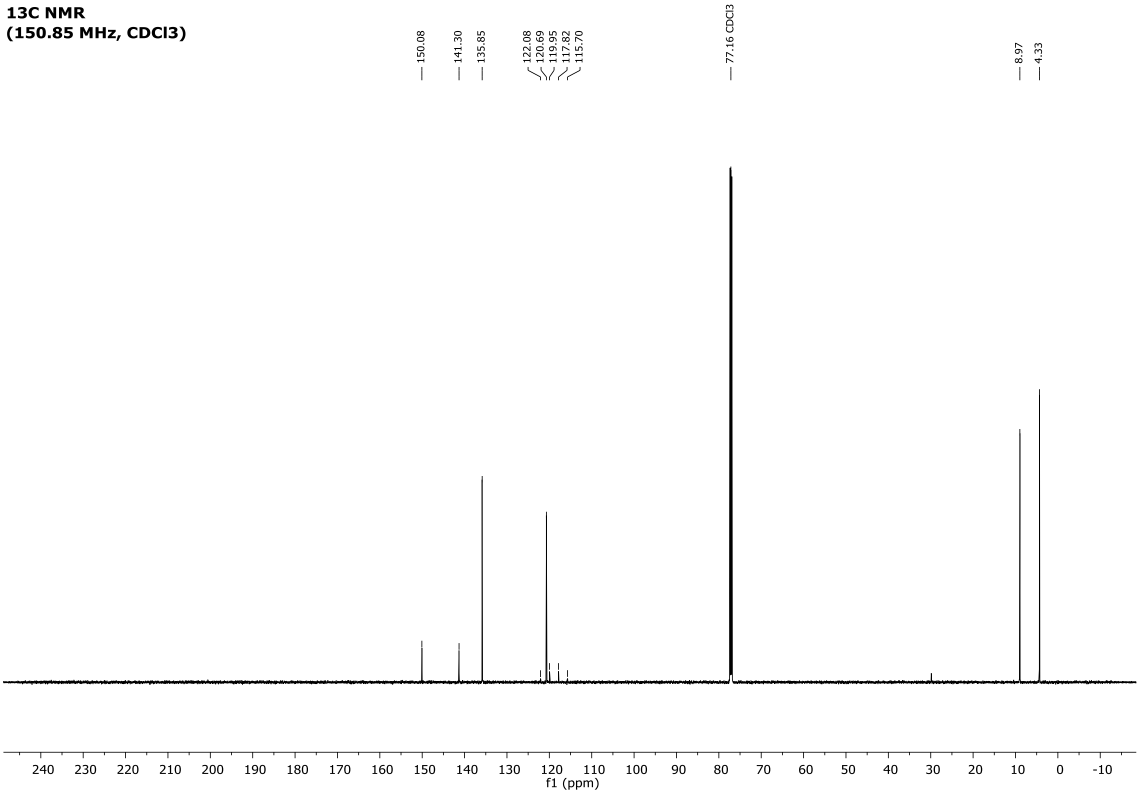

19F NMR
(564.38 MHz, CDCl3)

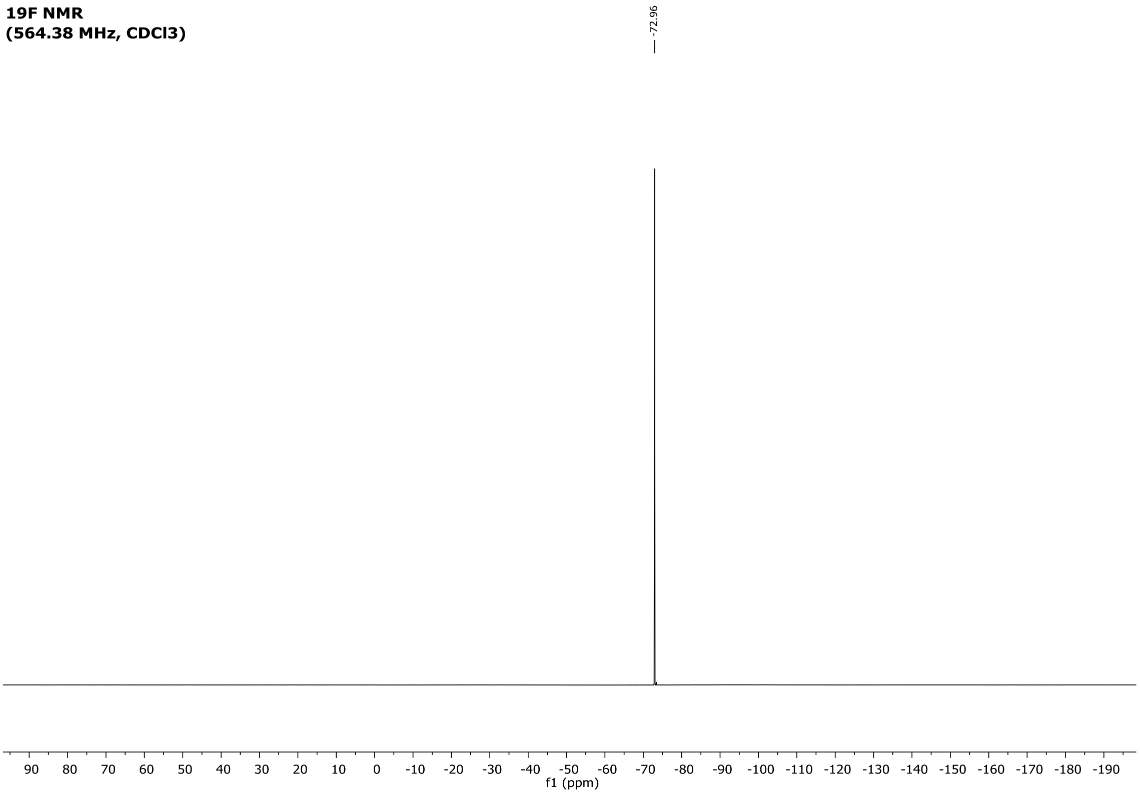

Triethyl(4-iodophenyl)germane

1H NMR
(599.86 MHz, CDCl3)

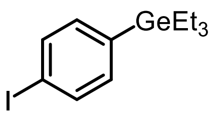

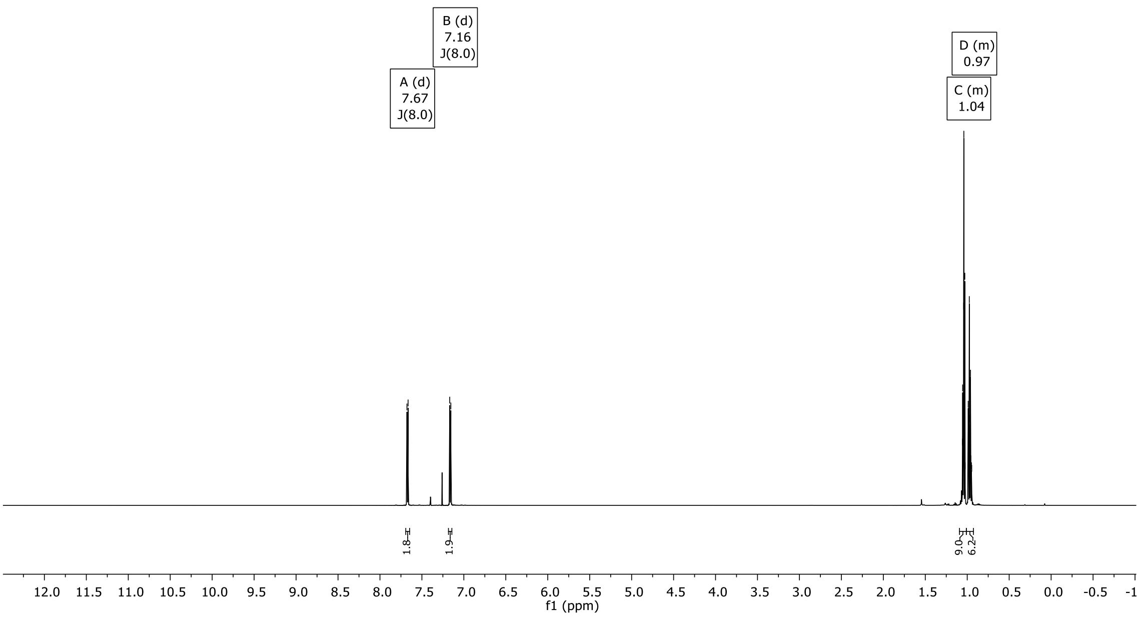

13C NMR
(150.85 MHz, CDCl3)

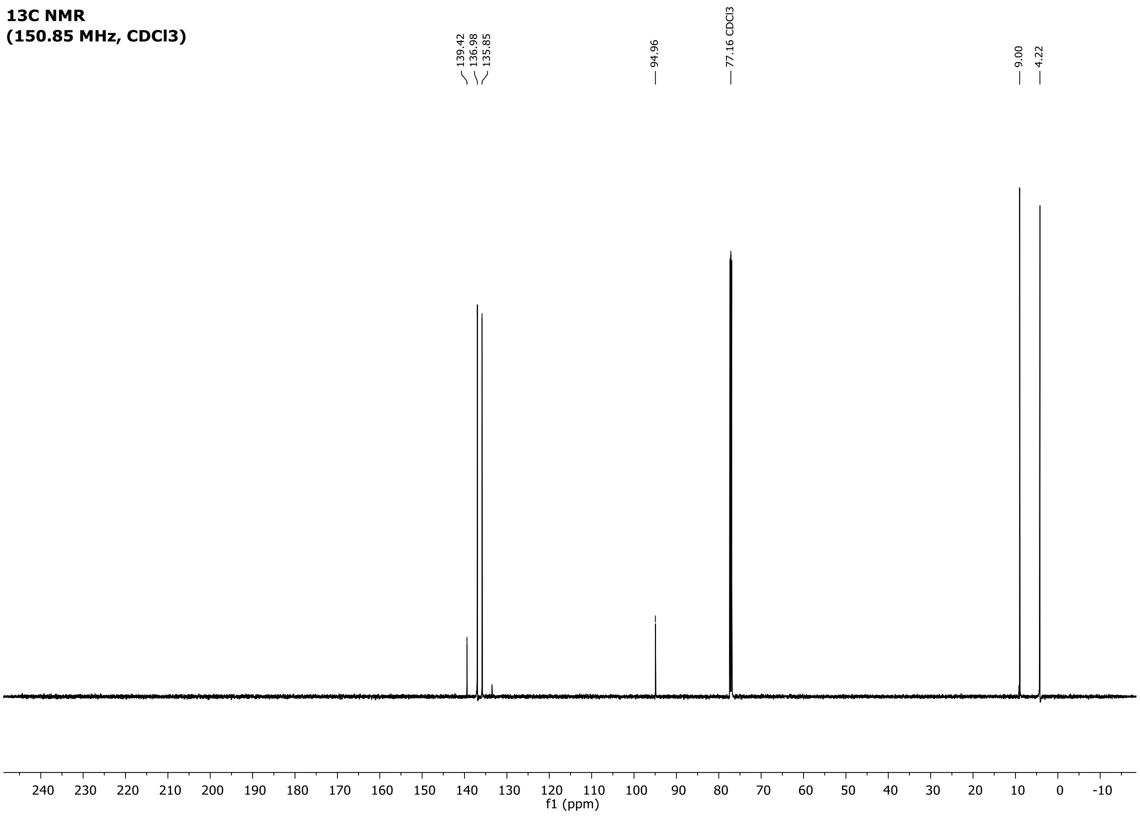

Trimethyl(4-(triethylgermyl)phenyl)silane

1H NMR
(599.86 MHz, CDCl3)

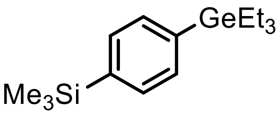

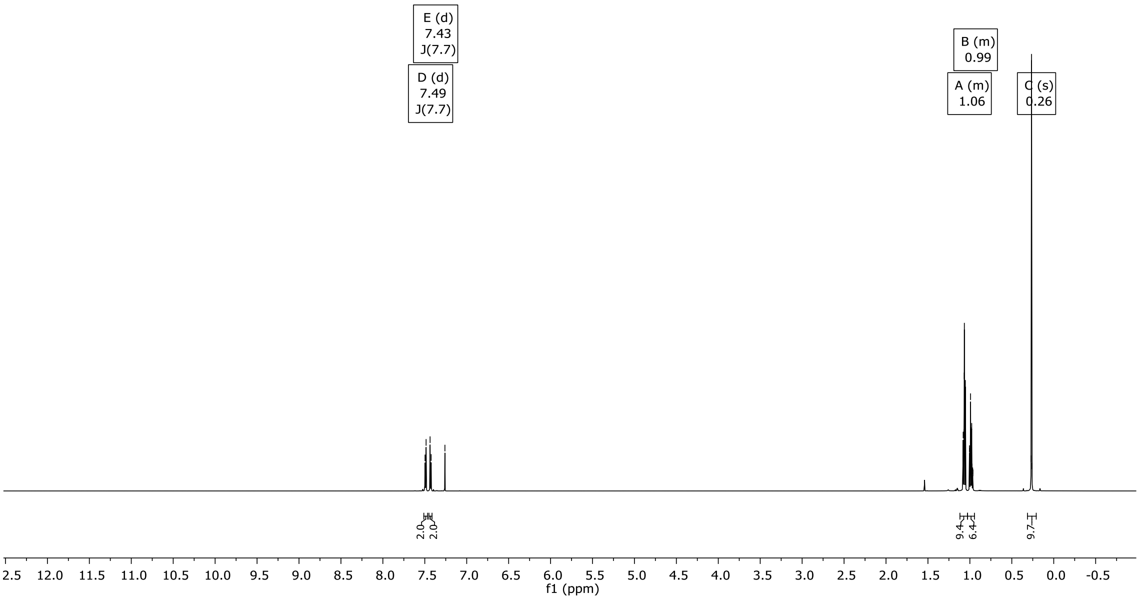

13C NMR
(150.85 MHz, CDCl3)

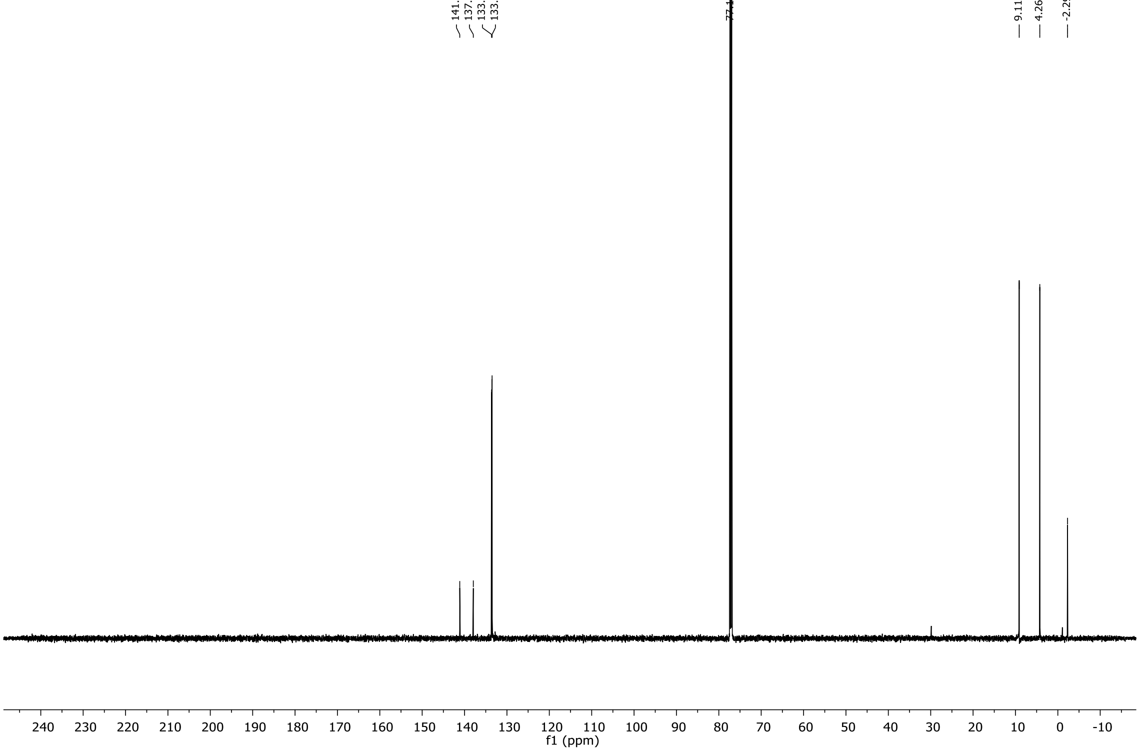

Triethyl(4-(4,4,5,5-tetramethyl-1,3,2-dioxaborolan-2-yl)phenyl)germane

1H NMR
(599.86 MHz, CDCl3)

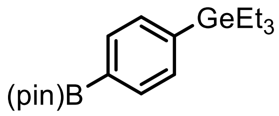

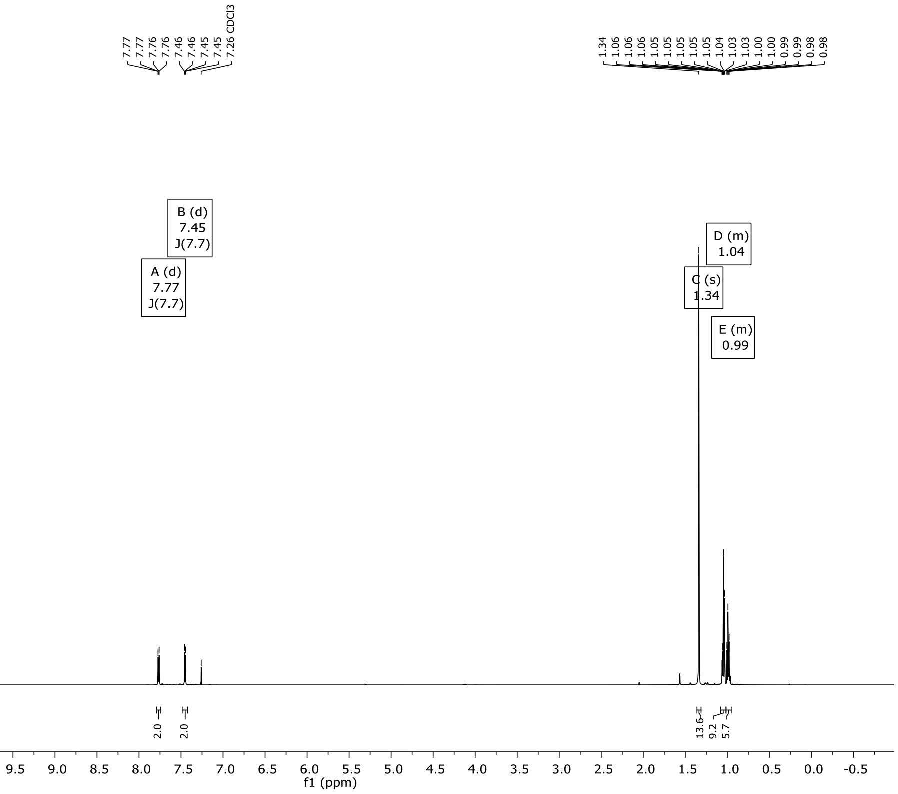

13C NMR
(150.85 MHz, CDCl3)

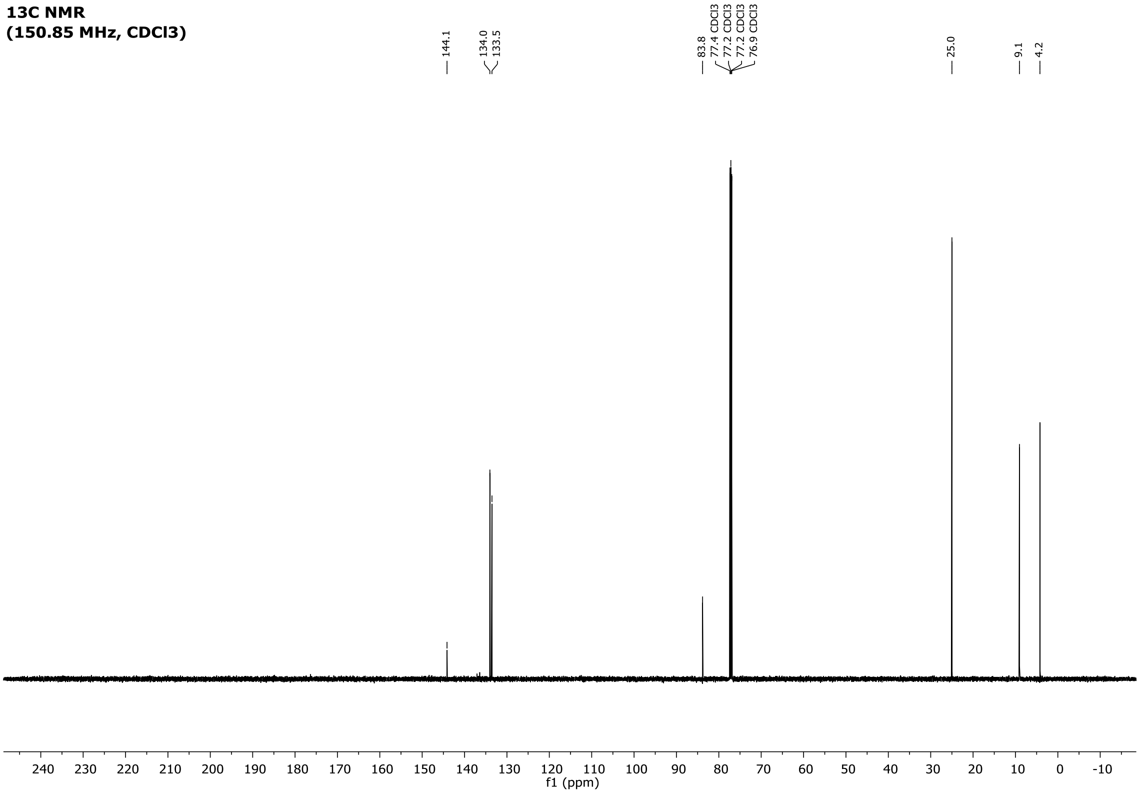

Triethyl(naphthalen-1-yl)germane

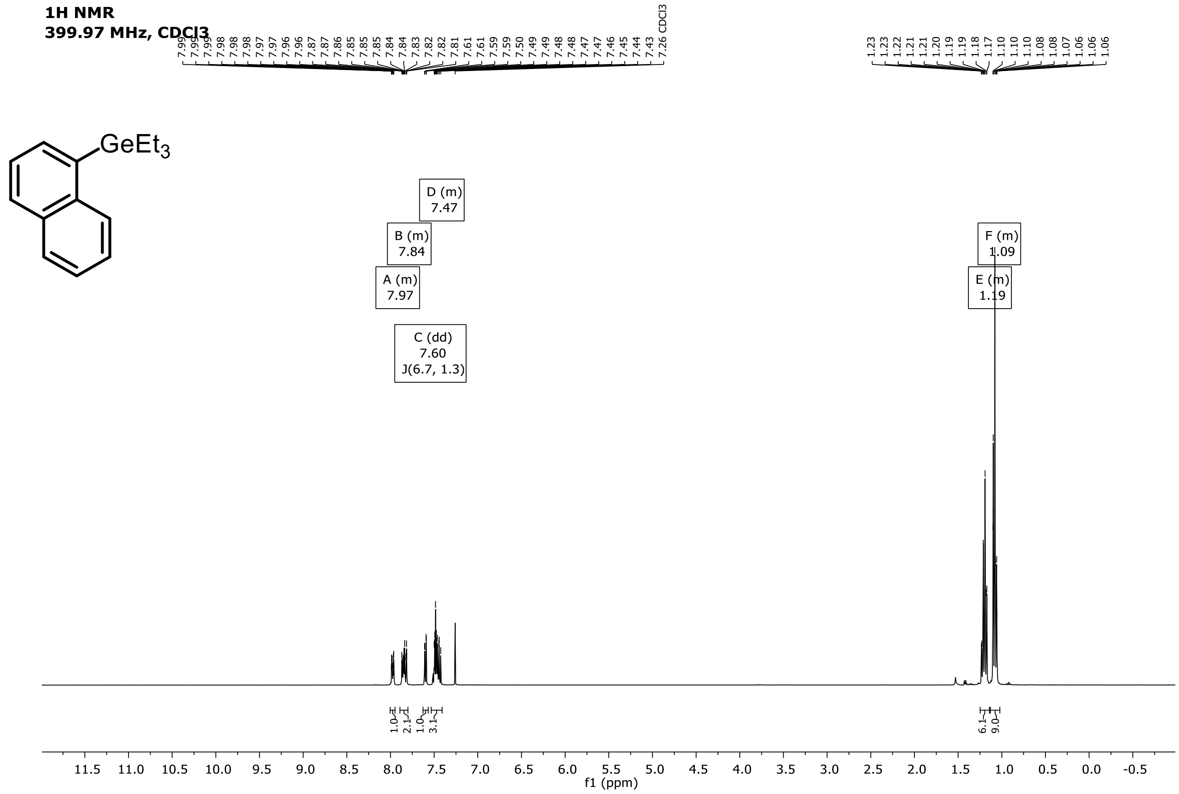

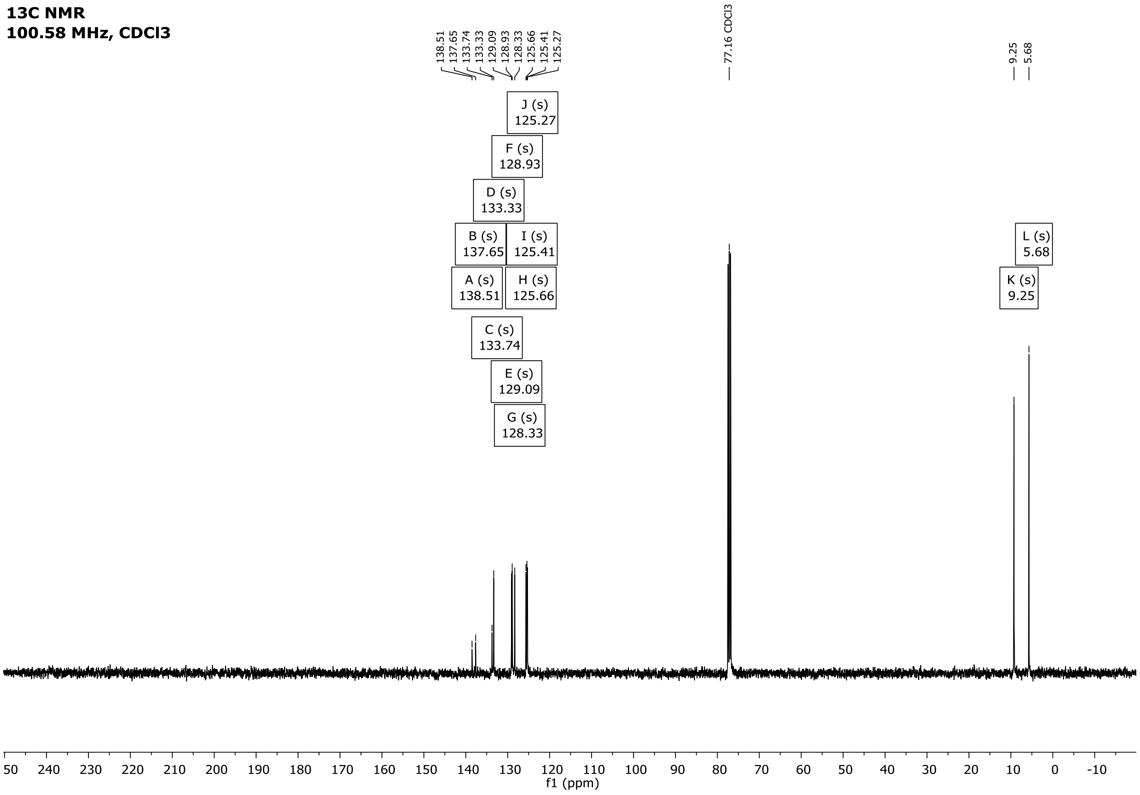

Triethyl(phenanthren-9-yl)germane

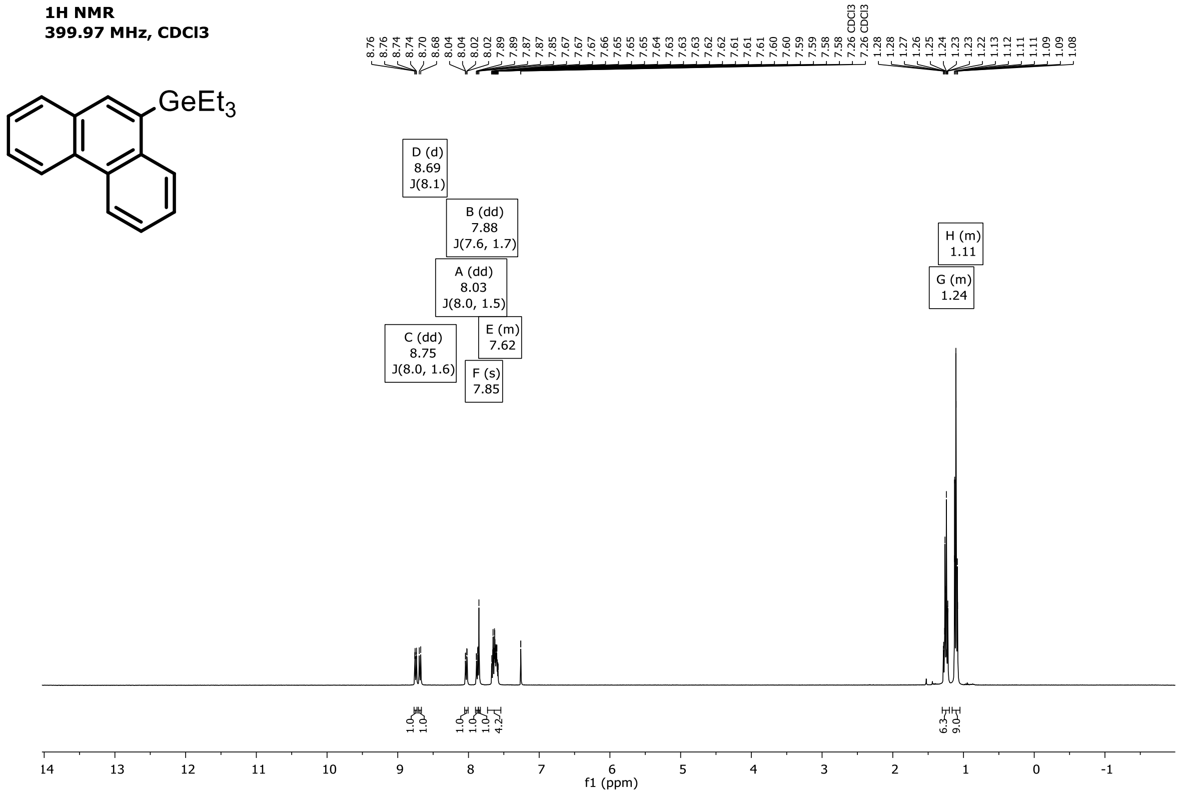

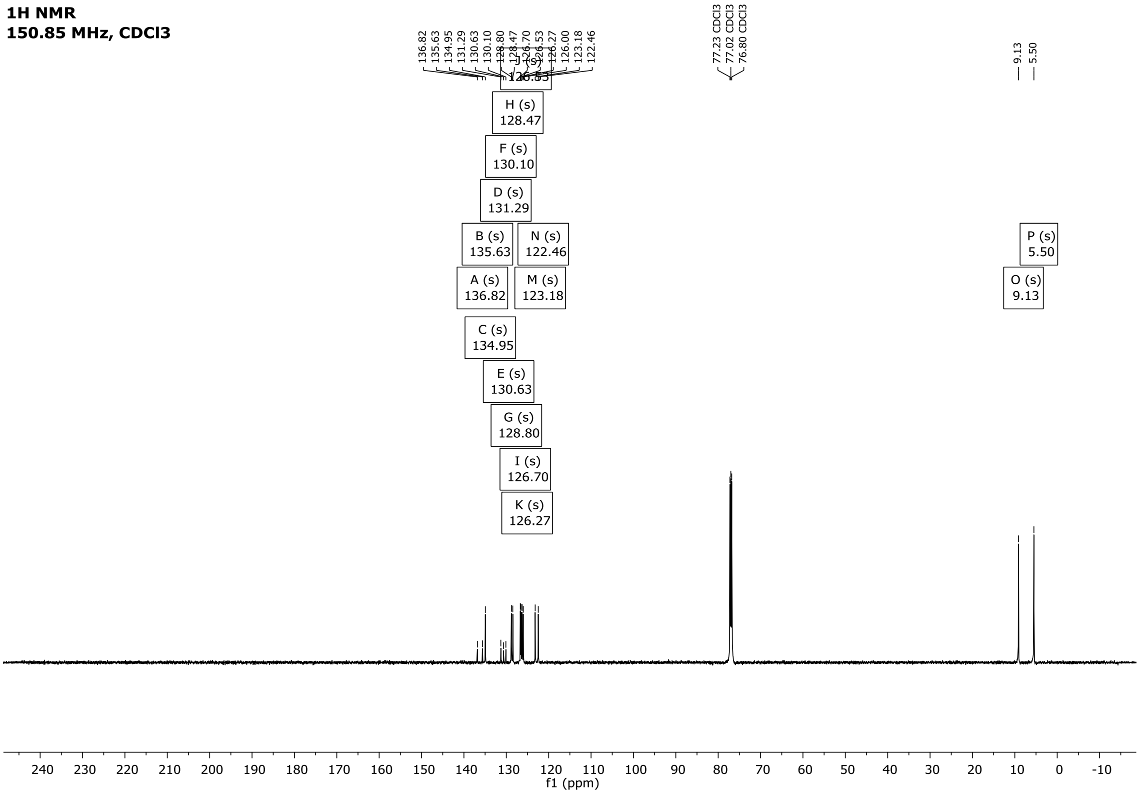

4-(Thiophen-2-yl)-2,3-dihydro-1*H*-inden-1-one

1H NMR
(599.86 MHz, CDCl3)

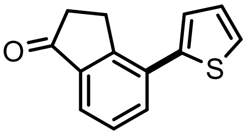

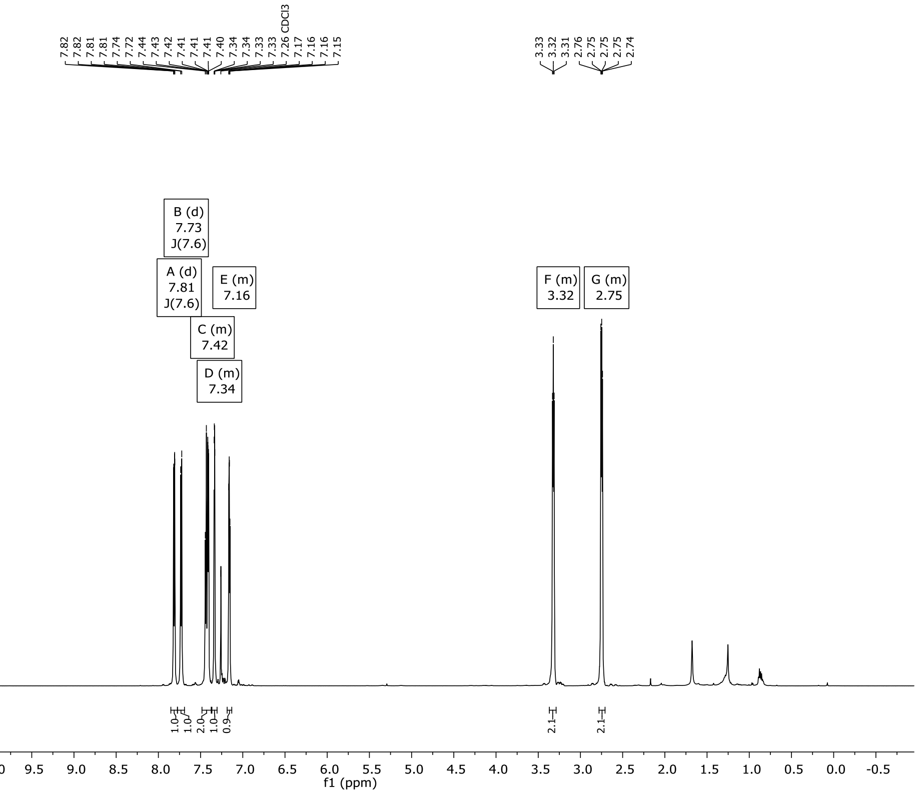

13C NMR
(150.85 MHz, CDCl3)

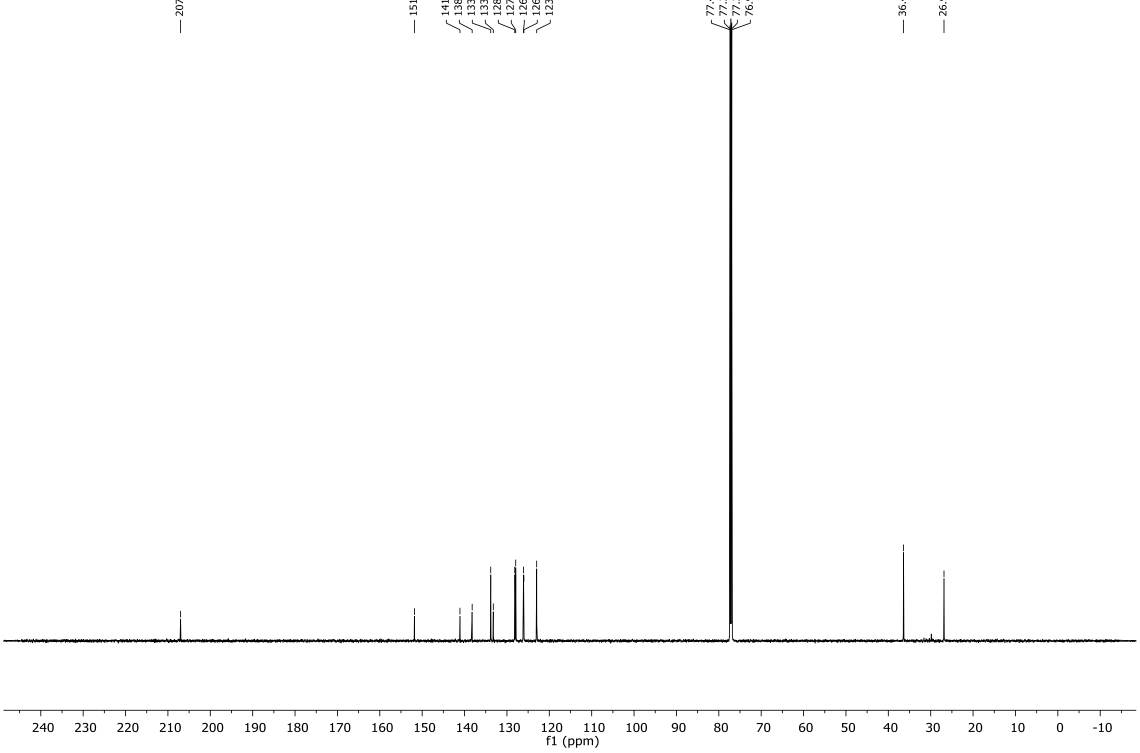

3’-Bromo-4’-fluoro-[1,1'-biphenyl]-4-yl trifluoromethanesulfonate

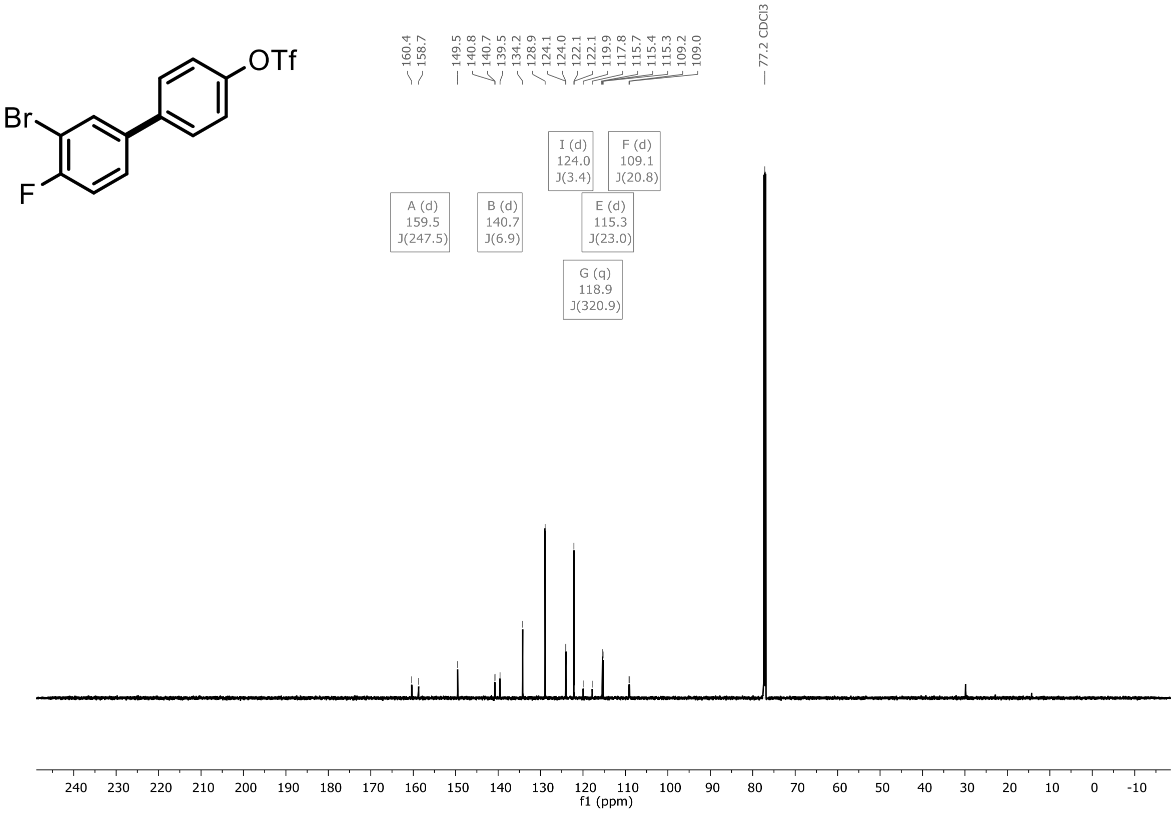

19F NMR
(564.38 MHz, CDCl3)

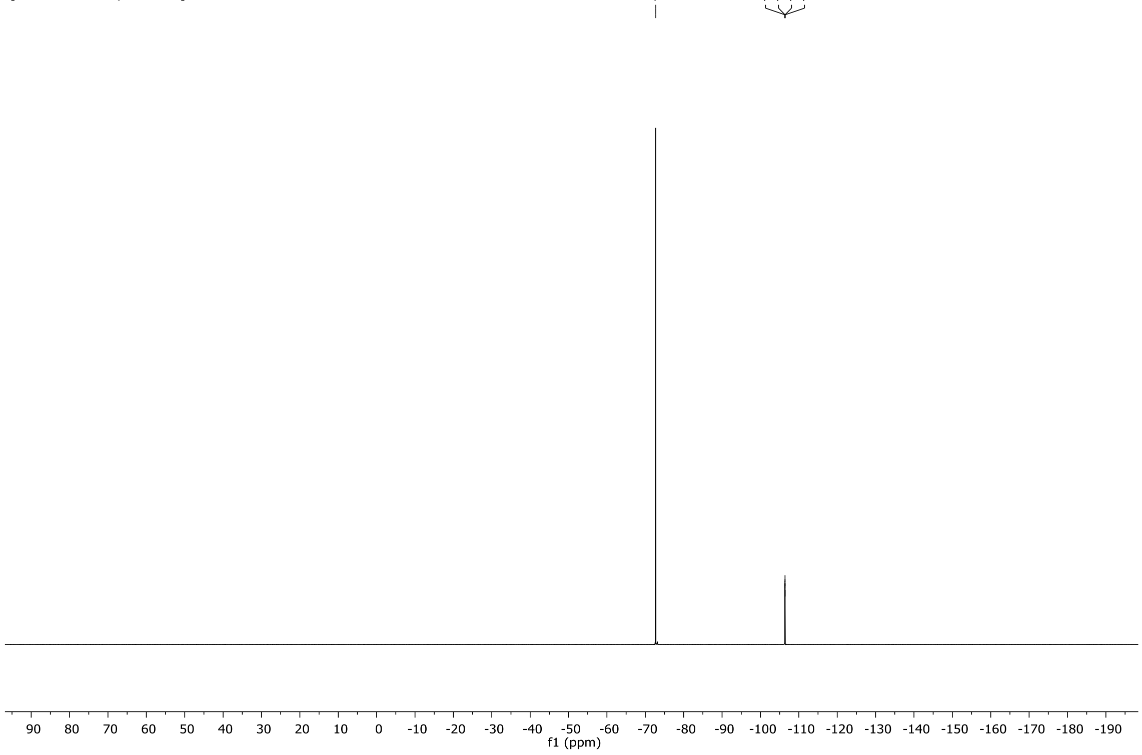

19F NMR
(564.38 MHz, CDCl3)

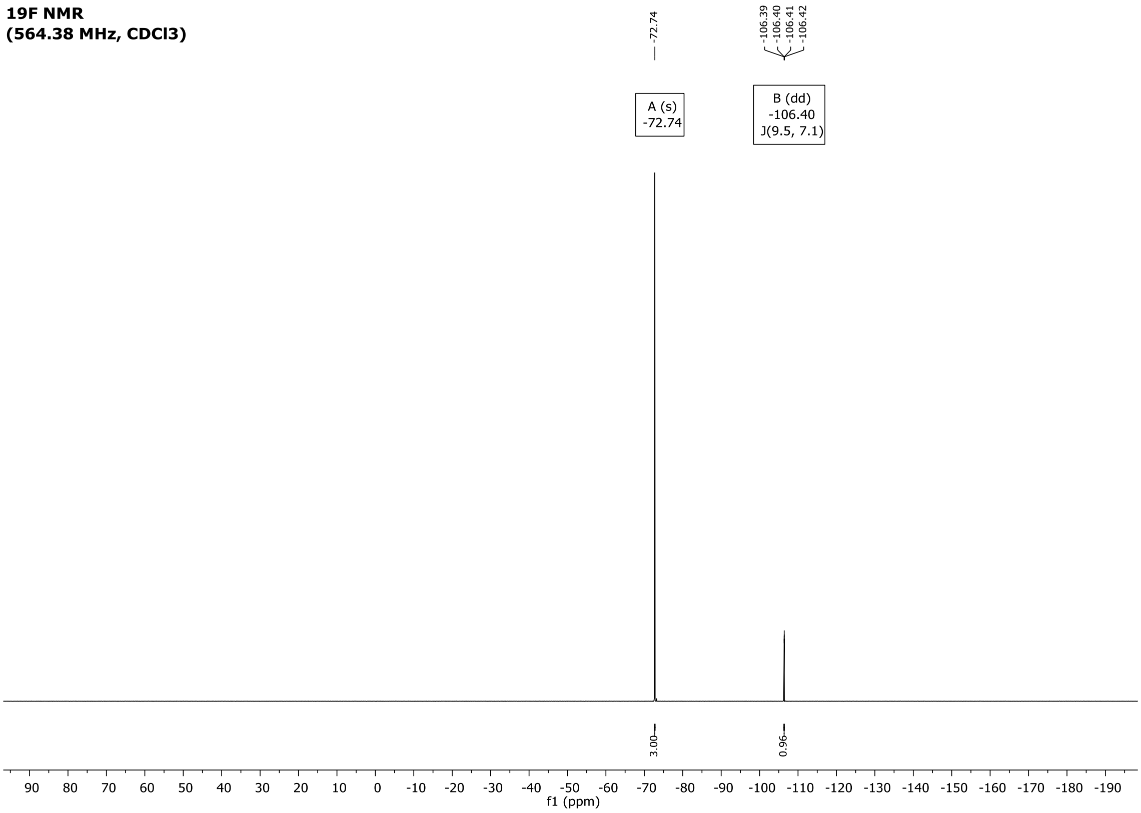

Trimethyl(3'-nitro-[1,1'-biphenyl]-4-yl)silane

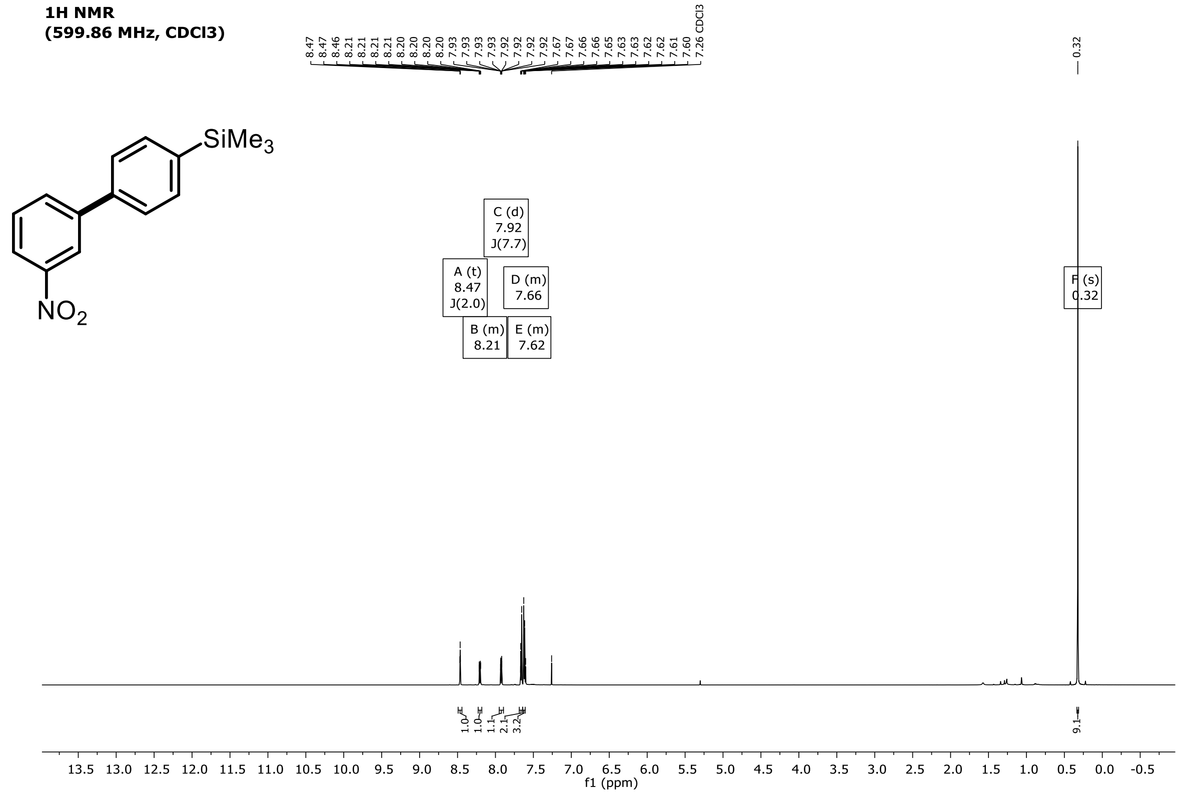

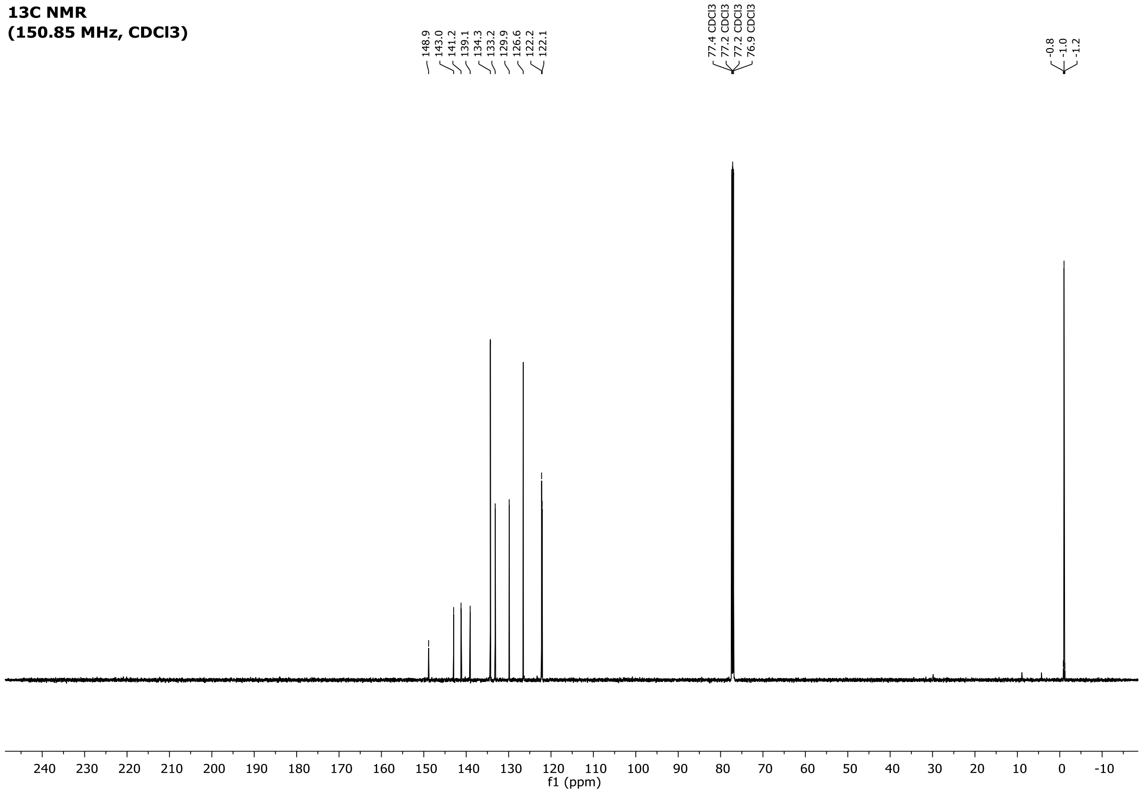

1-(3-Fluorophenyl)naphthalene

1H NMR
599.86 MHz, CDCl3

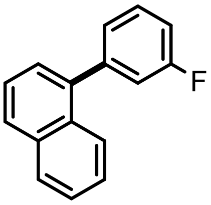

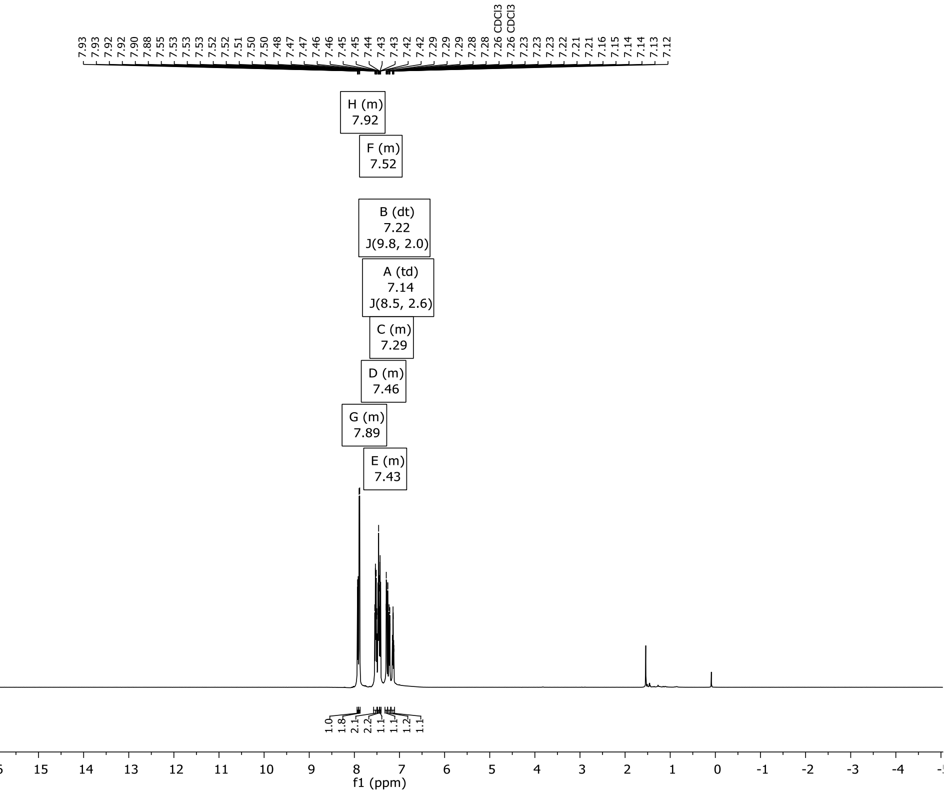

13C NMR
150.85 MHz, CDCl3

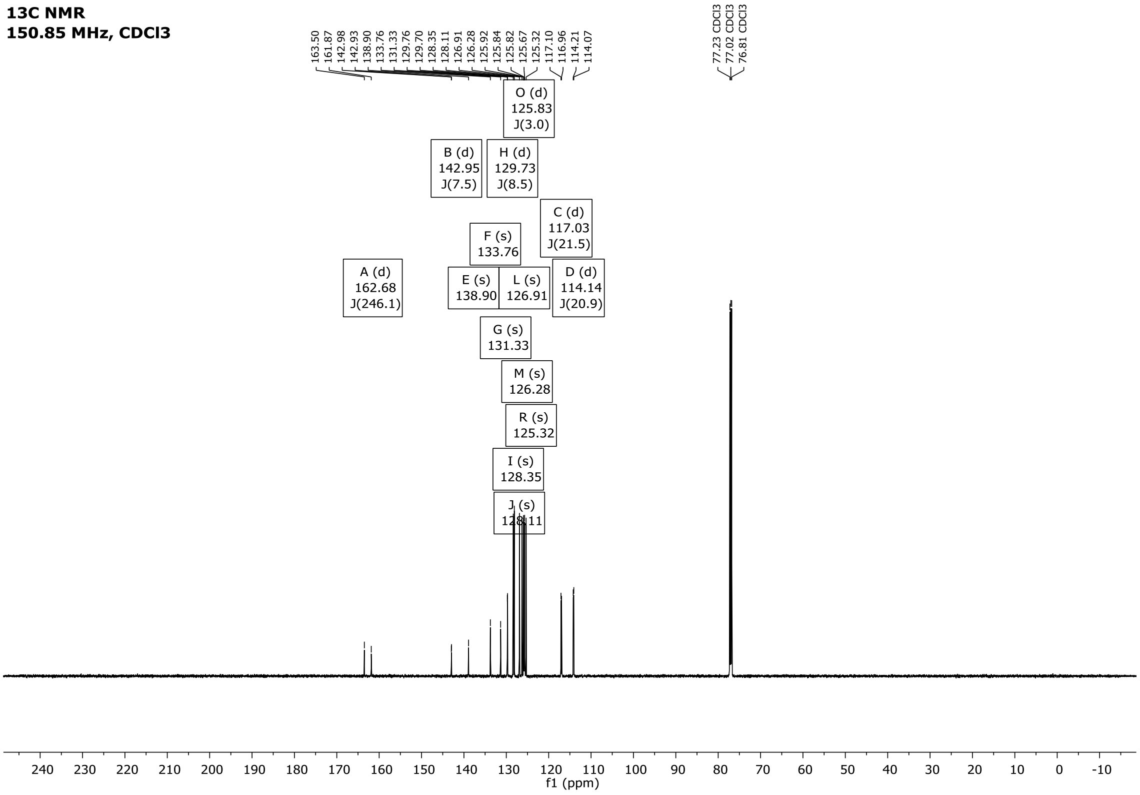

19F NMR
564.40 MHz, CDCl3

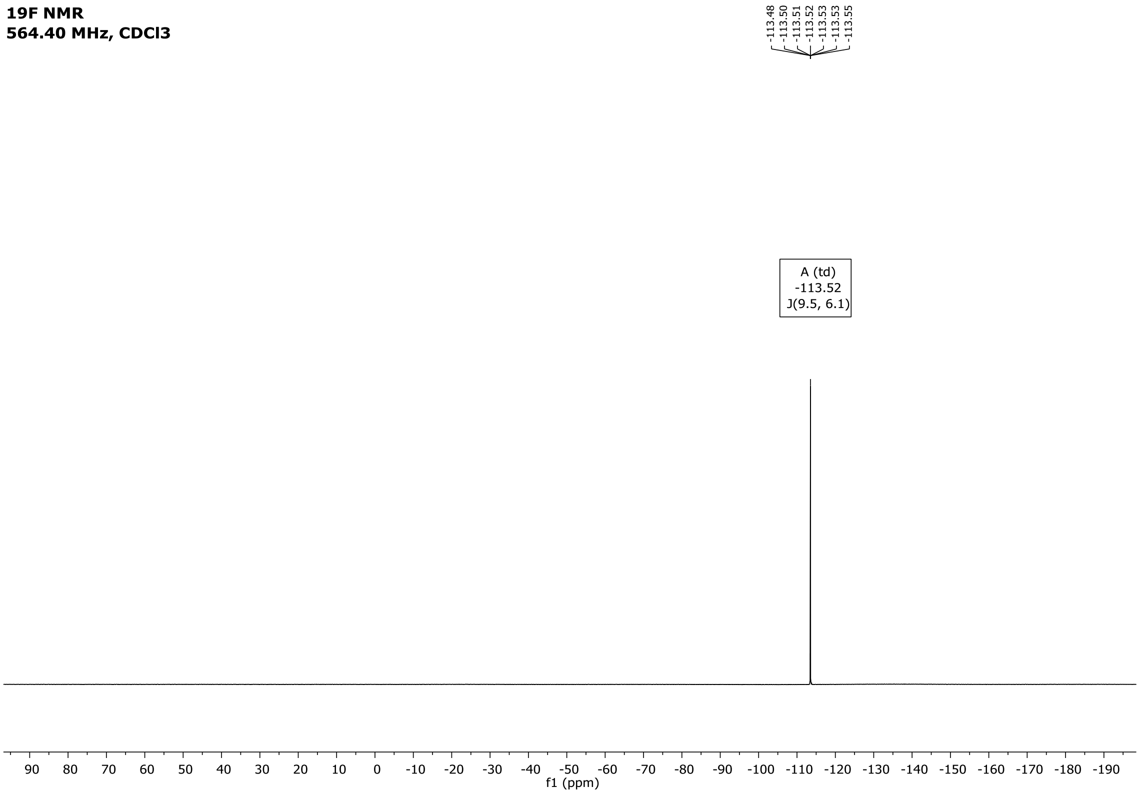

9 References

(1) Bielawski, M.; Aili, D.; Olofsson, B. Regiospecific one-pot synthesis of diaryliodonium tetrafluoroborates from arylboronic acids and aryl iodides. *J. Org. Chem.* **2008**, *73*, 4602.

(2) Beaud, R.; Phipps, R. J.; Gaunt, M. J. Enantioselective Cu-catalyzed arylation of secondary phosphine oxides with diaryliodonium salts toward the synthesis of P-chiral phosphines. *J. Am. Chem. Soc* **2016**, *138*, 13183.

(3) Petros, A. M.; Dinges, J.; Augeri, D. J.; Baumeister, S. A.; Betebenner, D. A.; Bures, M. G.; Elmore, S. W.; Hajduk, P. J.; Joseph, M. K.; Landis, S. K.; Nettesheim, D. G.; Rosenberg, S. H.; Shen, W.; Thomas, S.; Wang, X.; Zanze, I.; Zhang, H.; Fesik, S. W. Discovery of a potent inhibitor of the antiapoptotic protein Bcl-xL from NMR and parallel synthesis. *J. Med. Chem.* **2006**, *49*, 656.

(4) Wei, J.; Liu, K. M.; Duan, X. F. Cobalt-catalyzed biaryl couplings via C-F bond activation in the absence of phosphine or NHC ligands. *J. Org. Chem.* **2017**, *82*, 1291.

(5) Bright, T. V.; Dalton, F.; Elder, V. L.; Murphy, C. D.; O'Connor, N. K.; Sandford, G. A convenient chemical-microbial method for developing fluorinated pharmaceuticals. *Org. Biomol. Chem.* **2013**, *11*, 1135.

(6) Ackermann, L.; Born, R.; Spatz, J. H.; Meyer, D. Efficient aryl-(hetero)aryl coupling by activation of C-Cl and C-F bonds using nickel complexes of air-stable phosphine oxides. *Angew Chem Int Edit* **2005**, *44*, 7216.

(7) Dai, C.; Fu, G. C. The first general method for palladium-catalyzed Negishi cross-coupling of aryl and vinyl chlorides: Use of commercially available Pd(P(t-Bu)3)2 as a catalyst. *J. Am. Chem. Soc* **2001**, *123*, 2719.

(8) Keaveney, S. T.; Schoenebeck, F. Palladium-catalyzed decarbonylative trifluoromethylation of acid fluorides. *Angew. Chem. Int. Ed.* **2018**, *57*, 4073.

(9) Shi, S.; Meng, G.; Szostak, M. Synthesis of biaryls through nickel-catalyzed Suzuki–Miyaura coupling of amides by carbon–nitrogen bond cleavage. *Angew. Chem. Int. Ed.* **2016**, *55*, 6959.

(10) Zoller, J.; Fabry, D. C.; Rueping, M. Unexpected dual role of titanium dioxide in the visible light heterogeneous catalyzed C-H arylation of heteroarenes. *ACS Catalysis* **2015**, *5*, 3900.

(11) Thompson, A. M.; Sutherland, H. S.; Palmer, B. D.; Kmentova, I.; Blaser, A.; Franzblau, S. G.; Wan, B. J.; Wang, Y. H.; Ma, Z. K.; Denny, W. A. Synthesis and structure-activity relationships of varied ether linker analogues of the antitubercular drug (6S)-2-nitro-6-{[4-(trifluoromethoxy)benzyl]oxy}-6,7-dihydro-5H-imidazo[2,1-b][1,3]oxazine (PA-824). *J. Med. Chem.* **2011**, *54*, 6563.

(12) Hackenberger, D.; Weber, P.; Blakemore, D. C.; Goossen, L. J. Synthesis of 3-substituted 2-arylpyridines via Cu/Pd-catalyzed decarboxylative cross-coupling of picolinic acids with (hetero)aryl halides. *J. Org. Chem.* **2017**, *82*, 3917.

(13) Shen, Y.; Chen, J. X.; Liu, M. C.; Ding, J. C.; Gao, W. X.; Huang, X. B.; Wu, H. Y. Copper-catalyzed direct C-H arylation of pyridine N-oxides with arylboronic esters: one-pot synthesis of 2-arylpyridines. *Chem. Commun.* **2014**, *50*, 4292.

(14) Liang, Y. F.; Steinbock, R.; Yang, L.; Ackermann, L. Continuous visible-light photoflow approach for a manganese-catalyzed (het)arene C-H arylation. *Angew. Chem. Int. Ed.* **2018**, *57*, 10625.

(15) Liu, T. P.; Zhao, X. M.; Shen, Q. L.; Lu, L. General and highly efficient fluorinated-N-heterocyclic carbene-based catalysts for the palladium-catalyzed Suzuki-Miyaura reaction. *Tetrahedron* **2012**, *68*, 6535.

(16) Luo, Z. J.; Zhao, H. Y.; Zhang, X. Highly selective Pd-catalyzed direct C-F bond arylation of polyfluoroarenes. *Org. Lett.* **2018**, *20*, 2543.

(17) Hofer, M.; Genoux, A.; Kumar, R.; Nevado, C. Gold-catalyzed direct oxidative arylation with boron coupling partners. *Angew. Chem. Int. Ed.* **2016**, *56*, 1021.

(18) Minard, C.; Palacio, C.; Cariou, K.; Dodd, R. H. Selective Suzuki-Miyaura monocouplings with symmetrical dibromoarenes and aryl ditriflates for the one-pot synthesis of unsymmetrical triaryls. *Eur. J. Org. Chem.* **2014**, *2014*, 2942.

(19) Cornella, J.; Righi, M.; Larrosa, I. Carboxylic acids as traceless directing groups for formal meta-selective direct arylation. *Angew. Chem. Int. Ed.* **2011**, *50*, 9429.

(20) Novikov, R. A.; Tarasova, A. V.; Denisov, D. A.; Borisov, D. D.; Korolev, V. A.; Timofeev, V. P.; Tomilov, Y. V. [4+2] Annulation of donor-acceptor cyclopropanes with acetylenes using 1,2-zwitterionic reactivity. *J. Org. Chem.* **2017**, *82*, 2724.
(21) Hanley, P. S.; Ober, M. S.; Krasovskiy, A. L.; Whiteker, G. T.; Kruper, W. J. Nickel- and palladium-catalyzed coupling of aryl fluorosulfonates with aryl boronic acids enabled by sulfuryl fluoride. *ACS Catalysis* **2015**, *5*, 5041.
(22) Gehrtz, P. H.; Geiger, V.; Schmidt, T.; Srsan, L.; Fleischer, I. Cross-coupling of chloro(hetero)arenes with thiolates employing a Ni(0)-precatalyst. *Org. Lett.* **2019**, *21*, 50.
(23) Garcia-Cuadrado, D.; de Mendoza, P.; Braga, A. A. C.; Maseras, F.; Echavarren, A. M. Proton-abstraction mechanism in the palladium-catalyzed intramolecular arylation: Substituent effects. *J. Am. Chem. Soc* **2007**, *129*, 6880.
(24) Guo, P. F.; Joo, J. M.; Rakshit, S.; Sames, D. C-H Arylation of pyridines: High regioselectivity as a consequence of the electronic character of C-H bonds and heteroarene ring. *J. Am. Chem. Soc* **2011**, *133*, 16338.
(25) Zhou, Q. Z.; Zhang, B.; Su, L. J.; Jiang, T. S.; Chen, R. E.; Du, T. Q.; Ye, Y. Y.; Shen, J. F.; Dai, G. L.; Han, D. M.; Jiang, H. J. Palladium-catalyzed highly regioselective 2-arylation of 2,x-dibromopyridines and its application in the efficient synthesis of a 1713-HSD1 inhibitor. *Tetrahedron* **2013**, *69*, 10996.
(26) Ren, G. R.; Cui, X. L.; Wu, Y. J. Efficient synthesis of biaryls through the Kumada reaction catalyzed by carbene adducts of cyclopalladated ferrocenylimine. *Eur. J. Org. Chem.* **2010**, 2372.
(27) Liu, W. Y.; Xiong, D. Y.; Huo, P.; Mei, G. Q. Highly active palladium catalysts with bisacetylacetonate ligands for Suzuki-Miyaura cross-coupling reactions in mild conditions. *Chem. Lett.* **2017**, *46*, 1550.
(28) Yadav, M. R.; Nagaoka, M.; Kashihara, M.; Zhong, R. L.; Miyazaki, T.; Sakaki, S.; Nakao, Y. The Suzuki-Miyaura coupling of nitroarenes. *J. Am. Chem. Soc* **2017**, *139*, 9423.
(29) Ochiai, H.; Uetake, Y.; Niwa, T.; Hosoya, T. Rhodium-catalyzed decarbonylative borylation of aromatic thioesters for facile diversification of aromatic carboxylic acids. *Angew. Chem. Int. Ed.* **2017**, *56*, 2482.
(30) Qiu, D.; Wang, S. A.; Tang, S. B.; Meng, H.; Jin, L.; Mo, F. Y.; Zhang, Y.; Wang, J. B. Synthesis of trimethylstannyl arylboronate compounds by sandmeyer-type transformations and their applications in chemoselective cross-coupling reactions. *J. Org. Chem.* **2014**, *79*, 1979.
(31) Goodson, F. E.; Wallow, T. I.; Novak, B. M. Mechanistic studies on the aryl-aryl interchange reaction of $ArPdL_2I$ (L = triarylphosphine) complexes. *J. Am. Chem. Soc* **1997**, *119*, 12441.
(32) (a) Pilon, M. C.; Grushin, V. V. Synthesis and characterization of organopalladium complexes containing a fluoro ligand. *Organometallics* **1998**, *17*, 1774. (b) Fraser, S. L.; Antipin, M. Y.; Khroustalyov, V. N.; Grushin, V. V. Molecular fluoro palladium complexes. *J. Am. Chem. Soc* **1997**, *119*, 4769.
(33) Fitton, P.; Rick, E. A. The addition of aryl halides to tetrakis(triphenylphosphine)palladium(0). *J. Organomet. Chem.* **1971**, *28*, 287.
(34) Grushin, V. V.; Alper, H. Alkali-induced disproportionation of palladium(II) tertiary phosphine complexes, [L2PdCl2], to LO and palladium(O). Key intermediates in the biphasic carbonylation of ArX catalyzed by [L2PdCl2]. *Organometallics* **1993**, *12*, 1890.
(35) Takemoto, S.; Grushin, V. V. Nucleophile-catalyzed, facile, and highly selective C–H activation of fluoroform with Pd(II). *J. Am. Chem. Soc* **2013**, *135*, 16837.
(36) Frisch, M. J.; Trucks, G. W.; Schlegel, H. B.; Scuseria, G. E.; Robb, M. A.; Cheeseman, J. R.; Scalmani, G.; Barone, V.; Petersson, G. A.; Nakatsuji, H.; Li, X.; Caricato, M.; Marenich, A. V.; Bloino, J.; Janesko, B. G.; Gomperts, R.; Mennucci, B.; Hratchian, H. P.; Ortiz, J. V.; Izmaylov, A. F.; Sonnenberg, J. L.; Williams; Ding, F.; Lipparini, F.; Egidi, F.; Goings, J.; Peng, B.; Petrone, A.; Henderson, T.; Ranasinghe, D.; Zakrzewski, V. G.; Gao, J.; Rega, N.; Zheng, G.; Liang, W.; Hada, M.; Ehara, M.; Toyota, K.; Fukuda, R.; Hasegawa, J.; Ishida, M.; Nakajima, T.; Honda, Y.; Kitao, O.; Nakai, H.; Vreven, T.; Throssell, K.; Montgomery Jr., J. A.; Peralta, J. E.; Ogliaro, F.; Bearpark, M. J.; Heyd, J. J.; Brothers, E. N.; Kudin, K. N.; Staroverov, V. N.; Keith, T. A.; Kobayashi, R.; Normand, J.; Raghavachari, K.; Rendell, A. P.; Burant, J. C.; Iyengar, S. S.; Tomasi, J.; Cossi, M.; Millam, J. M.;

Klene, M.; Adamo, C.; Cammi, R.; Ochterski, J. W.; Martin, R. L.; Morokuma, K.; Farkas, O.; Foresman, J. B.; Fox, D. J. Gaussian 16 Rev. B.01. **2016**.
(37) Legault, C. Y.; Version 1.0b ed.; Université de Sherbrooke: 2009.
